# Unlocking Lewis acid–free metalation of O–nucleophiles in HFIP

**DOI:** 10.1038/s42004-026-02079-3

**Published:** 2026-06-02

**Authors:** Kacper Łyczek, Martyna Markwitz, Maciej Janicki, Krzysztof Kuciński

**Affiliations:** 1 Faculty of Chemistry, Adam Mickiewicz University, Uniwersytetu Poznanskiego 8, 61-614 Poznan, Poland; 2 Faculty of Biology, Adam Mickiewicz University, Uniwersytetu Poznanskiego 6, 61-614 Poznan, Poland

**Keywords:** Synthetic chemistry methodology, Reaction mechanisms

## Abstract

Allylsilanes are primarily used in allylation reactions that form new C–C bonds. Although they can also serve as silylating reagents to generate Si–O bonds, such transformations have generally required Brønsted or Lewis acid catalysis. Herein, we describe an acid-free method to access silyl ethers, siloxanes, and borasiloxanes via silylation or borylation. Hexafluoroisopropanol enables this reactivity, allowing a wide range of alcohols and silanols to undergo efficient conversion under mild conditions.

**Figure:**
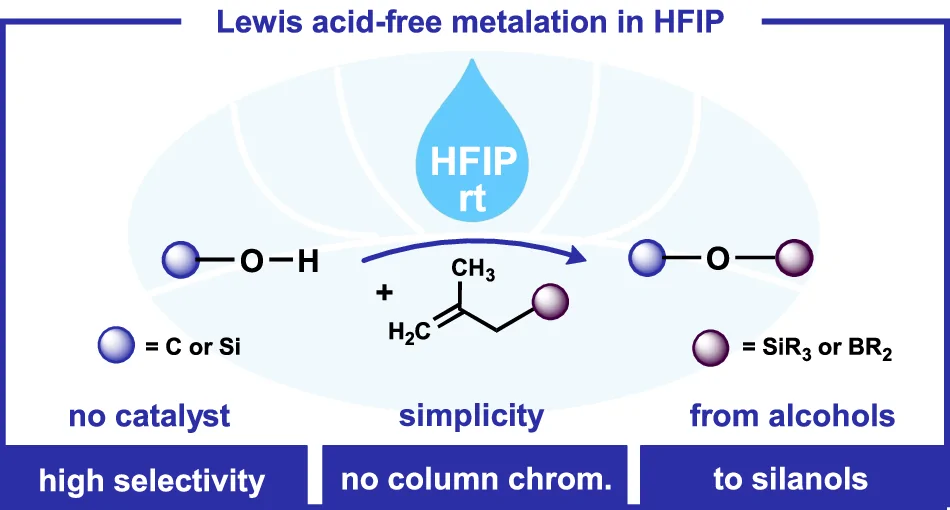

## Abstract (web summary)

Allylsilanes are typically used in C–C bond formation, but their potential as silylating agents for Si–O bond creation has been limited by the need for acid catalysis. Here, the authors introduce an acid-free method using hexafluoroisopropanol to efficiently convert alcohols and silanols into silyl ethers, siloxanes, and borasiloxanes.

## Introduction

In recent years, the so-called *methyl magic effect* has attracted considerable attention and has been documented in numerous studies, particularly in the synthesis of biologically active compounds^1,2^. By analogy, the term *“magic solvent”* can be aptly applied to hexafluoroisopropanol (HFIP)^3–10^. Over the past decade, several research groups, including those of Baeza^11–13^, Lebœuf^14–17^, Donohoe^18,19^, and others^20–27^, have substantially expanded the use of this solvent in organic synthesis, ranging from acid-mediated reactions^5^ to organocatalytic^28,29^ and photocatalytic processes^30,31^. This behavior arises from the unique physicochemical properties of HFIP, including strong hydrogen-bond donation, low nucleophilicity, a high dielectric constant, and exceptional stabilization of cationic intermediates^10,32^. However, these advantages should be balanced against considerations of sustainability and environmental impact when evaluating the broader use of HFIP.

**Fig. 1:**
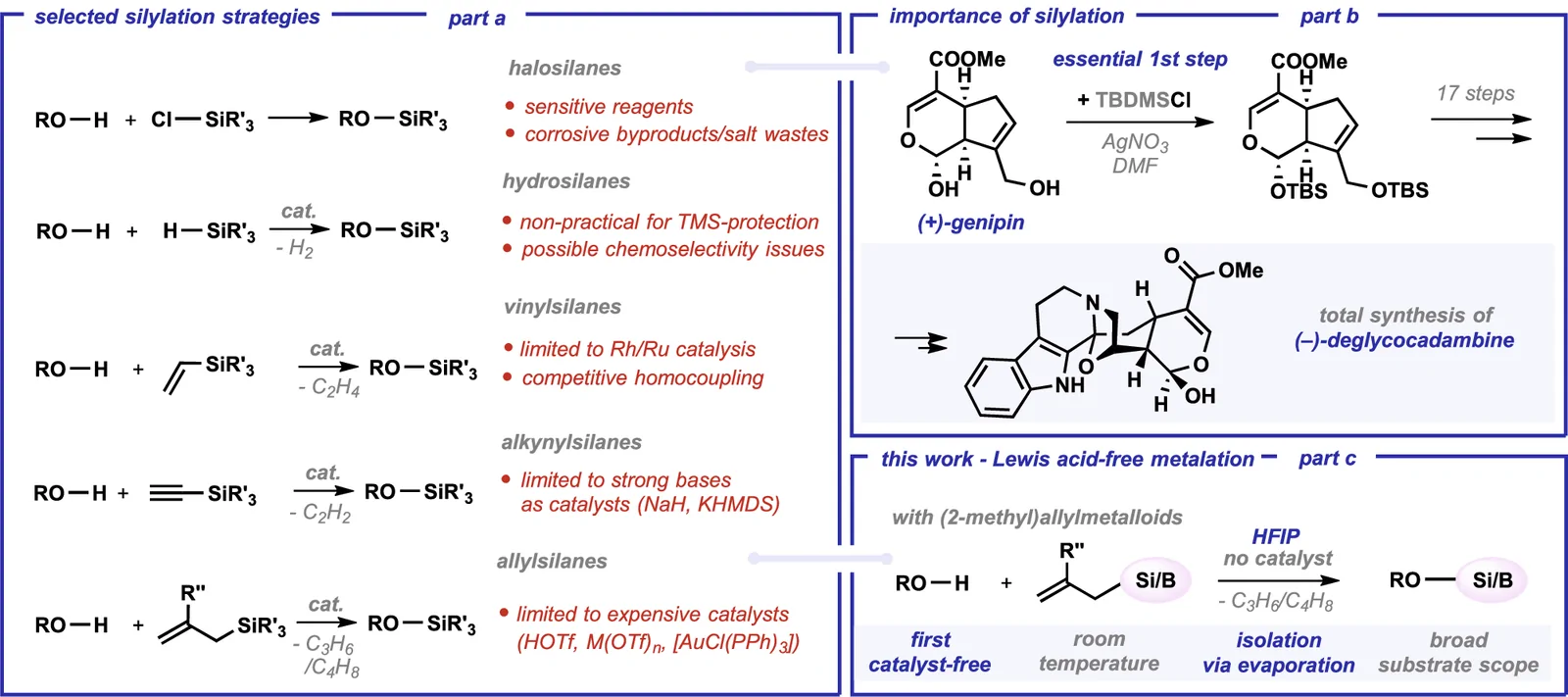
Context of the investigation. The figure summarizes the background of silylation chemistry and the concept of the HFIP-enabled catalyst-free strategy developed in this work. **a** Selected catalytic strategies for the synthesis of silyl ethers from O–nucleophiles and organosilicon reagents. **b** Representative application of silylation in total synthesis. **c** This work: HFIP-enabled Lewis acid–free metalation of O–nucleophiles with allylsilanes.

Silylation represents a fundamental strategy in organic synthesis, widely employed for the protection of hydroxyl groups and the introduction of silicon-based functionality (Fig. 1)^33–36^. Over decades, it has evolved into a cornerstone transformation enabling construction of complex molecules. While conventional halosilanes are effective^37^, they often require harsh conditions and generate halogenated corrosive byproducts or stoichiometric salt wastes, which can limit substrate scope and large-scale synthesis. Another class of reagents encompasses hydrosilanes^38–41^, various carbosilanes^42–45^, and others^46–49^, all of which offer distinct advantages and limitations and generally depend on a catalyst for effective transformation. In this context, 2-methylallylsilanes emerge as a compelling alternative, offering mild, halogen-free transformations and easy isolation procedure. The presence of a *β*-methyl substituent additionally stabilizes reactive intermediates^50^, enhancing selectivity and reaction control. Nonetheless, the broader application of these reagents remains restricted, as their effective activation generally necessitates strong Lewis or Brønsted acids^51–56^.

Very recently, we reported the functionalization of secondary benzylic alcohols with various thiols, allylsilanes, activated arenes, and indoles, in which HFIP was crucial for additional stabilization of the generated secondary benzylic cations^57^. Building on our ongoing interest in organosilicon chemistry, we aimed to extend the scope of HFIP-enabled transformations to include a Lewis acid-free silylation of *O*–nucleophiles with allylsilanes. So far, the HFIP/organosilane combination has generally referred to the use of organosilicon compounds as reductants^58,59^ (see exceptions^60,61^). Thus, we set out to investigate the use of hexafluoroisopropanol in silylation in detail. Initially, we hypothesized that this magic solvent could facilitate the process, eliminating the need for acidic conditions. As a result, we developed a highly efficient method for silyl ethers, siloxanes, and borasiloxanes synthesis.

## Results and discussion

**Fig. 2:**
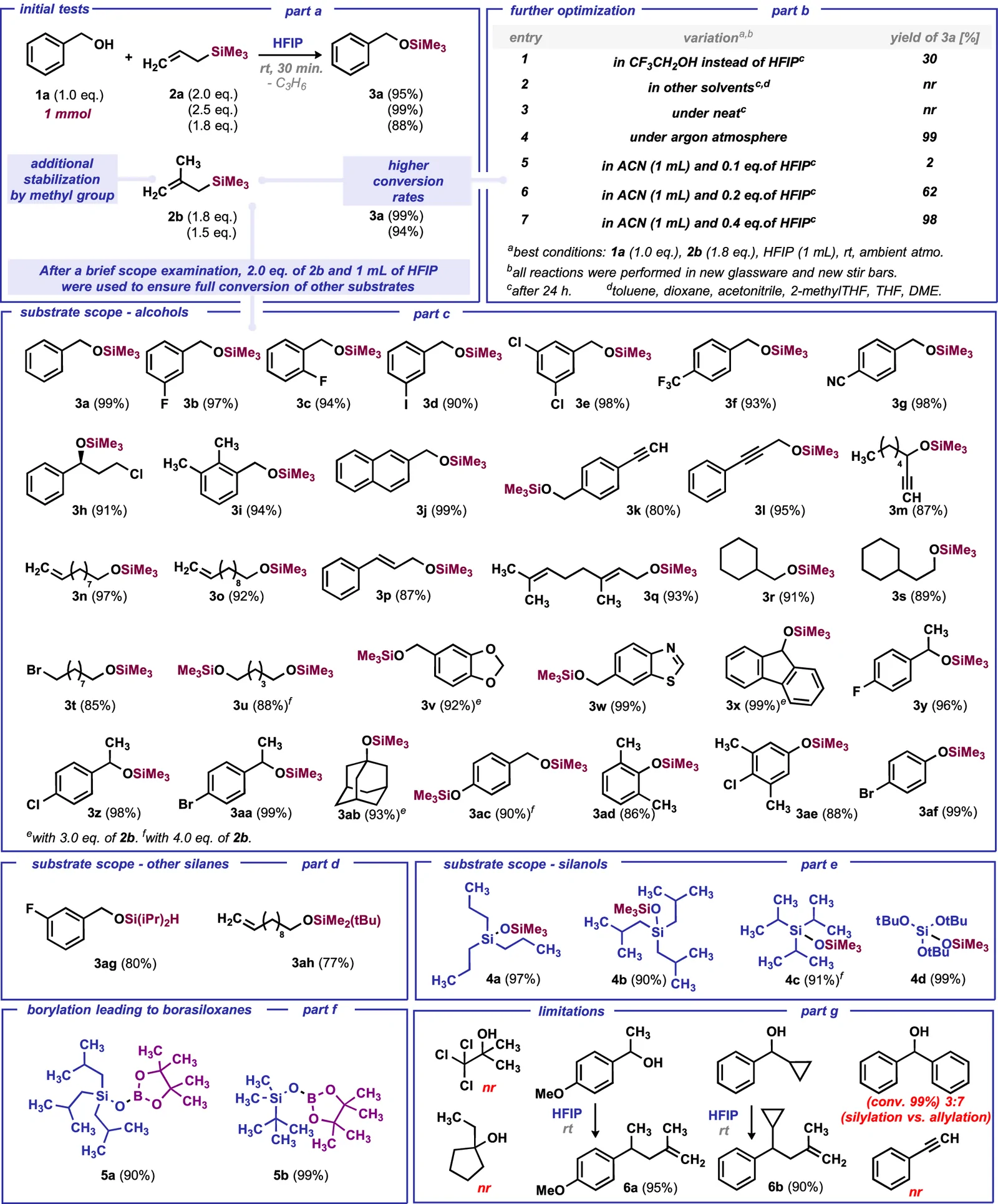
Optimization studies and substrate scope. The figure summarizes the optimization studies, substrate scope, and extensions of the HFIP-mediated catalyst-free silylation protocol. **a** Initial optimization studies with allyltrimethylsilane (**2a**) and 2-methylallyltrimethylsilane (**2b**). **b** Further optimization studies including solvent screening, atmosphere effects, and HFIP loading. **c** Substrate scope for alcohols and phenols. **d** Scope of allylsilanes. **e** Synthesis of siloxanes from silanols. **f** Synthesis of borasiloxanes using 2-methylallyl pinacolborane. **g** Limitations.

We started our studies by choosing allyltrimethylsilane (**2a**) as a model silylating agent in combination with benzyl alcohol (**1a**), in the presence of HFIP as the solvent (Fig. 2a). The initial experiment proved effective, affording 88% conversion with 1.8 eq. of **2a**. Increasing the loading of the silylating reagent improved the conversion to 99% when 2.5 eq. of **2a** were employed. On this basis, we evaluated an alternative commercially available allylsilane, 2-methylallyltrimethylsilane (**2b**), which bears an additional methyl substituent expected to enhance stabilization of the developing carbocation. Gratifyingly, the use of 1.8 eq. of **2b** resulted in quantitative (99%) conversion of **1a** to **3a**, and 2-methylallyltrimethylsilane was therefore selected for subsequent studies. Nevertheless, allyltrimethylsilane can also be considered an effective silylating reagent, although a larger excess is required to achieve high conversion. Three additional parameters were evaluated in subsequent screening (Fig. 2b): solvent identity (entries 1–3), solvent volume (entries 5–7), and reaction atmosphere (entry 4). Notably, all optimization and substrate scope studies were performed using new stir bars and vials to avoid contamination. Initially, the solvent most closely related to HFIP, namely trifluoroethanol, was evaluated. In this case, only 30% conversion of **1a** to **3a** was observed after 24 h. Other solvents were then examined (e.g., toluene, dioxane, acetonitrile, 2-methyltetrahydrofuran, THF and DME); however, no reaction was observed in any of these cases. We also investigated whether HFIP could be employed in catalytic amounts. Notably, the use of 20 mol% HFIP in acetonitrile afforded 62% conversion, whereas doubling the HFIP loading resulted in 99% conversion to **3a**.

Nevertheless, for subsequent studies, we selected to use the initial 1 mL of HFIP, owing to the limited solubility of several substrates. These results unequivocally demonstrate the essential role of HFIP in the reaction system, as it is the only medium that enables the transformation to proceed in the absence of either Brønsted or Lewis acids, which have thus far been considered indispensable when employing allylsilanes. To assess the generality of the HFIP-mediated silylation, we examined the coupling of a series of alcohols featuring different functional groups and steric properties (Fig. 2c). Coupling reactions of **2b** with primary benzyl alcohols bearing either electron-withdrawing (**1b**–**1h**) or electron-donating substituents (**1i**–**1j**) proceeded efficiently, affording yields of 90–99%. Importantly, the position of substituents on the aromatic ring (*ortho*, *meta*, or *para*) had any significant effect on the efficiency of the reaction. Next, primary and secondary alcohols containing a triple bond (**1k**–**1m**) were evaluated, as such functionalities can affect chemoselectivity in reactions using other silylating reagents, where competitive processes such as hydrosilylation with hydrosilanes or C–H silylation with hydrosilanes or carbosilanes may occur. These reactions demonstrated that the developed method is highly selective, leading exclusively to the formation of silyl ethers (**3k** to **3m**) in high yields. It is worth noting that the system was also evaluated in the context of C–H silylation of phenylacetylene (Fig. 2g). Although discussed as a limitation, this feature can also be advantageous, since hydrosilanes, alkynylsilanes, and trifluorotrimethylsilane can silylate both O–H and C–H groups. Next, similarly as before, the influence of carbon–carbon double bonds on the reaction outcome was examined. This is particularly relevant when comparing with methods employing hydrosilanes and vinylsilanes, which are often significantly less selective for such substrates. In this case as well, the HFIP-mediated system leaves the unsaturated bond intact, affording the corresponding products **3n**–**3q** in yields of up to 97%. Subsequently, aliphatic alcohols bearing cyclic or halogen substituents were also found to be highly reactive, affording products **3r**–**3t** in 85–91% yields. Gratifyingly, pentane-1,5-diol was readily incorporated into this protocol (**3u**, 88% yield). Encouraged by these results, we next explored heterocyclic alcohols as biorelevant scaffolds, each of which afforded the desired products with very good efficiency (**3v**–**3w**, 92–99% yields). Next, we were pleased to find that other secondary alcohols were also efficiently silylated under the standard conditions, affording products **3x**–**3aa** in excellent yields (96–99%). Notably, secondary benzyl alcohols bearing electron-donating substituents were unsuitable, as additional stabilization of the benzyl carbocation partially led to competing allylation products (**6a**–**6b**, Fig. 2g). For tertiary alcohols, the outcome was strongly influenced by steric factors. For example, 1-adamantanol underwent the transformation with high efficiency (**3ab**, 93%), whereas sterically hindered substrates such as 1,1,1-trichloro-2-methyl-2-propanol and 1-ethyl-1-hydroxycyclopentane were completely unreactive after 24h (Fig. 2g). Next, we sought to obtain silylated phenols. Using our reaction system (Fig. 2c), four trimethylsilylated variants were afforded (**3ac**–**3af**, 86–99% yield). Variation of the silyl coupling partner demonstrated that the transformation remained effective with alternative 2-methylallylsilanes (Fig. 2d). Coupling of **1b** with **2c** proceeded in slightly diminished yield (**3ag**, 80%), as did the reaction of **1o** with **2d** (**3ah**, 77%). Next, we examined whether our reaction manifold could be applied to the synthesis of siloxanes (Fig. 2e). Gratifyingly, a range of silanols were efficiently silylated under the standard conditions, affording siloxanes **4a**–**4d** in good yields. Encouragingly, this strategy could be further extended to the synthesis of borasiloxanes (Fig. 2f)^62,63^. To this end, 2-methylallyl pinacolborane was prepared and subsequently employed as the coupling partner with two silanols, enabling the formation of borasiloxanes **5a** and **5b** in very good yields (90% and 99%, respectively).

**Fig. 3:**
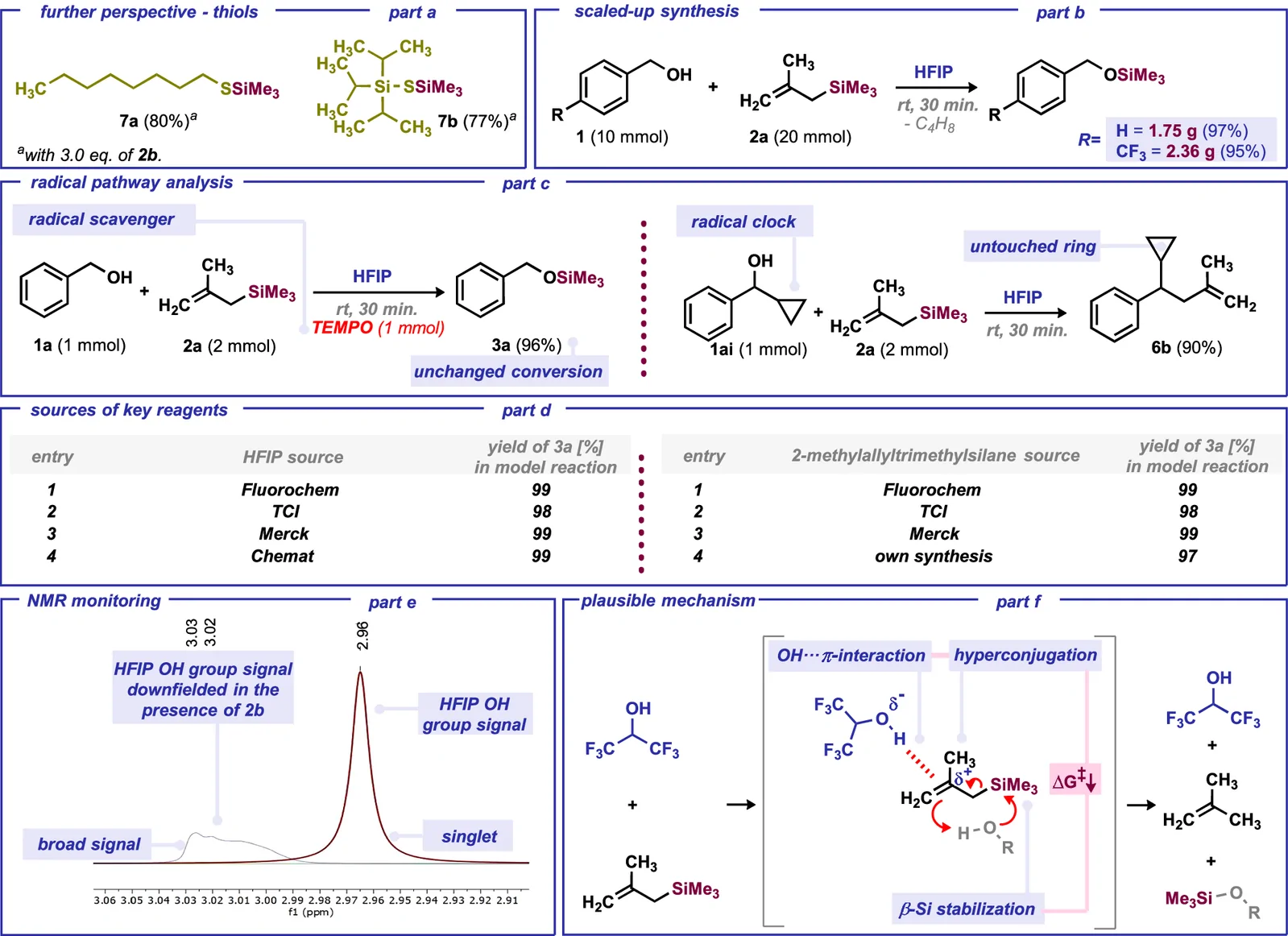
Applications and mechanistic considerations. The figure presents additional applications of the methodology together with preliminary mechanistic investigations. **a** Extension of the methodology to S–silylation. **b** Scale-up experiments. **c** Radical pathway analysis using TEMPO and a radical clock substrate. **d** Evaluation of reagent sources. **e** NMR monitoring of interactions between HFIP and 2-methyl-allyltrimethylsilane. **f** Proposed mechanistic rationale involving O–H···π interactions and β-silicon stabilization.

To obtain additional insights into the reaction, including mechanistic aspects, we conducted preliminary experiments (Fig. 3). We initially explored the feasibility of *S*–silylation (Fig. 3a), which is considerably more challenging than the corresponding *O*–silylation. To our surprise, two thiosilanes (**7a** and **7b**) were successfully obtained, the latter being an unsymmetrical disilathiane. Furthermore, we successfully demonstrated the scalability of our method by conducting the synthesis of **3a** and **3f** on a 10-fold larger scale (Fig. 3b). Subsequently, we investigated the possible involvement of radical intermediates. As previously demonstrated in the scope studies (Fig. 2g), *α*-cyclopropylbenzyl alcohol undergoes the 2-methylallylation reaction to afford product **6b**.

This transformation simultaneously serves as a radical clock experiment (Fig. 3c). No byproducts indicative of cyclopropane ring opening were detected in the reaction mixture. Taken together with the absence of any effect upon addition of a radical scavenger (TEMPO), these observations rule out the involvement of free radical species. A key aspect of catalyst-free transformations concerns the origin of the reagents involved in the reaction. We found that both HFIP and 2-methylallyltrimethylsilane exhibited comparable (high) reactivity regardless of their source. Finally, we investigated the behaviour of the individual reagents in the presence of HFIP using NMR spectroscopy (Fig. 3e and Supplementary Information: Mechanistic Studies NMR). Additional NMR spectra and mechanistic experiments are provided in the Supplementary Information. For a mixture of HFIP and 3-fluorobenzyl alcohol in CDCl₃, a pronounced shift of the OH proton was observed (sole HFIP: singlet at 2.96 ppm; mixture: multiplet at 3.44 ppm). The OH signal of the benzyl alcohol was also shifted downfield (2.04 ppm *vs*. 2.21 ppm). These observations are consistent with the formation of a hydrogen-bonded complex, in which HFIP acts as the hydrogen-bond donor (exhibiting the stronger shift), while the benzyl alcohol predominantly serves as the hydrogen-bond acceptor. Nevertheless, we believe that the dominant role in the reaction outcome is played by pre-activation of the electrophile (**2b**) in the presence of HFIP. In this case, the ¹H NMR experiment indicates weak but detectable interactions between HFIP and 2-methylallyltrimethylsilane, manifested by broadening and a slight downfield shift of the (HFIP) OH signal (from 2.96 to 3.02 ppm). This observation may be indicative of an HFIP···*π* interaction, potentially associated with hyperconjugative effects of the methyl group and the *β*-silicon effect, which together could stabilize the developing *β*-carbocation character and thereby lower the activation barrier for nucleophilic attack by an alcohol. To probe the plausibility of an O–H···π interaction between HFIP and 2-methylallyltrimethylsilane, the system was examined using preliminary DFT calculations (Supplementary Information: Preliminary computational studies and Supplementary Data 1). The BSSE-corrected interaction energy was found to be -7.82 kcal mol⁻¹ (-32.7 kJ mol⁻¹). In combination with the short H···π distance and the nearly linear O–H···π arrangement, these data are consistent with a stabilizing O–H···π contact in the studied system. Altogether, these factors may explain why no external catalyst is required. Importantly, the above-mentioned ¹H and ¹⁹F NMR experiments on a mixture of hexafluoroisopropanol and 2-methylallyltrimethylsilane showed no evidence for significant formation of an *O*-silylated HFIP species (Supplementary Information: Mechanistic Studies NMR). Next, a mixture of HFIP (1 mL) and 2-methylallyltrimethylsilane (1 mmol) was also stirred for an extended period of time and subsequently analyzed by GC–MS. Based on mass spectral data, the formation of silylated HFIP can be inferred, along with volatile byproducts arising from degradation of **2b**, including the corresponding symmetrical hexamethyldisiloxane and trimethyl(2,2,4-trimethylpent-4-en-1-yl)silane, consistent with literature reports (Smit) as a homocoupling product of **2b**.^26^ Importantly, when benzyl alcohol (**1a**) was added to such a mixture in the absence of **2b**, formation of **3a** was observed only in trace amounts (<1%). This result indicates that even if *O*-silylated HFIP is formed, it does not act as an efficient silyl donor under the reaction conditions. Consistent with the low nucleophilicity of HFIP, any such species is unlikely to accumulate to a level sufficient to account for the observed reactivity.

## Methods

### General information

2-Methylallyltrimethylsilane (**2b**) was obtained from multiple suppliers (Fluorochem, TCI, Merck, own synthesis^64^) and used as received, without further purification. Allyltrimethylsilane (**2a**) was obtained from multiple suppliers (TCI and Merck) and used as received, without further purification. Diisopropyl(2-methylallyl)silane (**2c**) and *tert*-butyldimethyl(2-methylallyl)silane (**2 d**) were synthesized in accordance with previous literature^65,66^. Hexafluoroisopropanol was sourced from Fluorochem, TCI, Merck, or Chemat and used as received without additional purification or drying. Other solvents employed during reaction optimization were purchased from Honeywell, dried over calcium hydride (CaH₂), and purified by distillation prior to use. Alcohols, silanols, and thiols were obtained from commercial suppliers (Sigma-Aldrich, Ambeed, Fisher, Acros, Fluorochem, Angene, and TCI) and used as received. 2-Methylallylpinacolborane was synthesized in accordance with the literature^67^. Reaction progress – primarily the conversion of alcohols – was monitored by gas chromatography (GC) using a Bruker Scion 460-GC and an Agilent 5977B GC/MSD equipped with an Agilent 8860 GC system. The structures of products were determined by NMR spectroscopy and mass spectrometry (GC-MS). The ^1^H NMR (400 MHz), ^13^C NMR (101 MHz), ^29^Si NMR (79 MHz), ^19^F NMR (377 MHz), and ^11^B (128 MHz) spectra were recorded on Bruker Avance III HD NanoBay spectrometer, using chloroform-d (CDCl_3_) or benzene-d6 (C_6_D_6_) as the solvent. Deuterated solvent was purchased from Sigma Aldrich (Merck) (CDCl_3_ 99.8 atom% D) and used as received. High resolution mass spectra (HRMS) were obtained using Impact HD mass spectrometer (Q-TOF type instrument equipped with electrospray ion source; Bruker Daltonics, Germany). The sample solutions (DCM:MeOH) were infused into the ESI source by a syringe pump (direct inlet) at the flow rate of 3 µL/min. The instrument was operated under the following optimized settings: end plate voltage 500 V; capillary voltage 4.2 kV; nebulizer pressure 0.3 bar; dry gas (nitrogen) temperature 200 °C; dry gas flow rate 4 L/min. The spectrometer was previously calibrated with the standard tune mixture.

### General synthetic procedures

All procedures can be found in Supporting Information File. Here, we present two example procedures. Additional experimental procedures, characterization data, NMR spectra, and mechanistic studies are provided in the Supplementary Information.

### Synthesis of compounds 3a-3t

To a 5 mL vial equipped with a magnetic stirring bar, alcohol (**1a**-**1t**, 1 mmol) was added. Then, 2-methylallyltrimethylsilane (**2b**, 2 mmol, 0.256 g), and HFIP (1 mL) have been placed in a vial. In most cases, the reaction required stirring for a period of 30 minutes. After this time, HFIP and an excess of **2b** were evaporated under reduced pressure to give desired products. The pure products **3a**-**3t** were identified by ^1^H NMR, ^13^C NMR, ^29^Si NMR, ^19^F NMR spectroscopy, and mass spectrometry.

### Synthesis of compound 3 u

To a 5 mL vial equipped with a magnetic stirring bar, pentane-1,5-diol (**1 u**, 1 mmol, 0.104 g) was added. Then, 2-methylallyltrimethylsilane (**2b**, 4 mmol, 0.512 g), and HFIP (1 mL) have been placed in a vial. The reaction required stirring for a period of 30 minutes. After this time, HFIP and an excess of **2b** were evaporated under reduced pressure to give desired product. The pure product **3 u** was identified by ^1^H NMR, ^13^C NMR, ^29^Si NMR spectroscopy, and mass spectrometry.

## Conclusions

In conclusion, we have developed a catalyst-free strategy for the silylation of alcohols, silanols, and thiols without the need for any Lewis or Brønsted acid. The distinctive hydrogen-bond-donating ability of hexafluoroisopropanol, together with its capacity to stabilize cationic intermediates, underpins this transformation. This enables highly efficient activation of 2-methylallyltrimethylsilane, further supported by intrinsic features of this reagent, namely the *β*-silicon effect and hyperconjugation by the methyl group. The combined contribution of these effects lowers the activation barrier, thereby allowing the reaction to proceed in the absence of a catalyst. Importantly, this catalyst-free method is user-friendly and broadly functional-group tolerant, enabling its immediate application to a range of synthetically important nucleophiles.

### Supporting information

Supporting Information is available free of charge on the Communications Chemistry website. Experimental section, characterization of the obtained products, their spectra, and the authors have cited additional references within the Supporting Information^64–89^.

## Supplementary information


Transparent Peer Review file
Supporting Information
Description of Additional Supplementary Files
Supplementary Data for Preliminary Calculations


## Data Availability

The data underlying this study are available in the published article, its Supplementary Information, and Supplementary Data 1. Supplementary Data 1 contains preliminary computational data in .XYZ format. Once the manuscript is available online, all NMR data related to this work will be deposited in our university’s open-access repository.
