# Supplementary material for: Unlocking Lewis acid–free metalation of O–nucleophiles in HFIP
Source: Commun Chem. 2026 Jun 2;9:288. doi: 10.1038/s42004-026-02079-3 (PMC13526861; doi:10.1038/s42004-026-02079-3)
Supplement: Supplementary file 1 [file 42004_2026_2079_MOESM1_ESM.pdf]

nature portfolio

Peer Review File

Unlocking Lewis Acid–Free Metalation of O–Nucleophiles in HFIP

Corresponding Author: Dr Krzysztof Kuciński

This manuscript has been previously reviewed at another journal. This document only contains information relating to versions considered at Communications Chemistry.

This file contains all reviewer reports in order by version, followed by all author rebuttals in order by version.

Version 0:

Reviewer comments:

Reviewer #1

(Remarks to the Author)
The authors have adequately adressed my comments and concerns, so that the manuscript is now suitable for publication in Communications Chemistry in its present form.

Open Access This Peer Review File is licensed under a Creative Commons Attribution 4.0 International License, which permits use, sharing, adaptation, distribution and reproduction in any medium or format, as long as you give appropriate credit to the original author(s) and the source, provide a link to the Creative Commons license, and indicate if changes were made.
In cases where reviewers are anonymous, credit should be given to 'Anonymous Referee' and the source.
The images or other third party material in this Peer Review File are included in the article's Creative Commons license, unless indicated otherwise in a credit line to the material. If material is not included in the article's Creative Commons license and your intended use is not permitted by statutory regulation or exceeds the permitted use, you will need to obtain

permission directly from the copyright holder.
To view a copy of this license, visit https://creativecommons.org/licenses/by/4.0/
