# Supplementary material for: Unlocking Lewis acid–free metalation of O–nucleophiles in HFIP
Source: Commun Chem. 2026 Jun 2;9:288. doi: 10.1038/s42004-026-02079-3 (PMC13526861; doi:10.1038/s42004-026-02079-3)
Supplement: Supplementary file 2 [file 42004_2026_2079_MOESM2_ESM.pdf]

Supporting Information

Unlocking Lewis Acid–Free Metalation of O–Nucleophiles in HFIP

Kacper Łyczek,[1] Martyna Markwitz,[1] Maciej Janicki[2] and Krzysztof Kuciński*,[1]

[1] Faculty of Chemistry, Adam Mickiewicz University, Poznań, Uniwersytetu Poznańskiego St. 8, 61-614 Poznań, Poland.

[2] Faculty of Biology, Adam Mickiewicz University, Poznań, Uniwersytetu Poznańskiego St. 6, 61-614 Poznań, Poland.

*email: kucinski.k@amu.edu.pl

Twitter account: @SiRCH_Kucina (KK)

https://www.kucinskilab.com/

ORCID iDs:

0009-0006-1549-499X (Kacper Łyczek, kaclyc@st.amu.edu.pl)

0009-0005-3251-6858 (Martyna Markwitz, marmar80@st.amu.edu.pl)

0000-0001-7800-2792 (Maciej Janicki, mjanicki@amu.edu.pl)

0000-0002-9339-6395 (Krzysztof Kuciński, kucinski.k@amu.edu.pl)

Content

GENERAL INFORMATION 9
GENERAL SYNTHETIC PROCEDURES 10
Synthesis of compounds **3a-3t** 10
Synthesis of compound **3u** 10
Synthesis of compound **3v** 10
Synthesis of compound **3w** 10
Synthesis of compound **3x** 10
Synthesis of compound **3y** 10
Synthesis of compounds **3z-3aa** 11
Synthesis of compound **3ab** 11
Synthesis of compound **3ac** 11
Synthesis of compounds **3ad-3af** 11
Synthesis of compound **3ag** 11
Synthesis of compound **3ah** 11
Synthesis of compound **4a, 4b,** and **4d** 11
Synthesis of compound **4c** 12
Synthesis of compound **5a** and **5b** 12
Synthesis of compound **6a** and **6b** 12
Synthesis of compound **7a** and **7b** 12
Scaled-up synthesis of **3a** 12
Scaled-up synthesis of **3f** 12
Experiment in the presence of radical scavenger 13
CHARACTERIZATION DATA FOR ALL PRODUCTS 14
(Benzyloxy)trimethylsilane (**3a**) 14
((3-Fluorobenzyl)oxy)trimethylsilane (**3b**) 14
((2-Fluorobenzyl)oxy)trimethylsilane (**3c**) 14
((3-Iodobenzyl)oxy)trimethylsilane (**3d**) 15
((3,5-Dichlorobenzyl)oxy)trimethylsilane (**3e**) 15
Trimethyl((4-(trifluoromethyl)benzyl)oxy)silane (**3f**) 15
4-(((Trimethylsilyl)oxy)methyl)benzonitrile (**3g**) 15
(*S*)-(3-Chloro-1-phenylpropoxy)trimethylsilane (**3h**) 16
((2,3-Dimethylbenzyl)oxy)trimethylsilane (**3i**) 16
Trimethyl(naphthalen-2-ylmethoxy)silane (**3j**) 16
((4-Ethynylbenzyl)oxy)trimethylsilane (**3k**) 17
Trimethyl((3-phenylprop-2-yn-1-yl)oxy)silane (**3l**) 17
Trimethyl(oct-1-yn-3-yloxy)silane (**3m**) 17
(Dec-9-en-1-yloxy)trimethylsilane (**3n**) 17

Trimethyl(undec-10-en-1-yloxy)silane (**3o**) 18
(Cinnamyloxy)trimethylsilane (**3p**) 18
(*E*)-((3,7-dimethylocta-2,6-dien-1-yl)oxy)trimethylsilane (**3q**) 18
(Cyclohexylmethoxy)trimethylsilane (**3r**) 19
(2-Cyclohexylethoxy)trimethylsilane (**3s**) 19
((9-Bromononyl)oxy)trimethylsilane (**3t**) 19
2,2,10,10-Tetramethyl-3,9-dioxa-2,10-disilaundecane (**3u**) 19
(Benzo[d][1,3]dioxol-5-ylmethoxy)trimethylsilane (**3v**) 20
6-(((Trimethylsilyl)oxy)methyl)benzo[d]thiazole (**3w**) 20
((9H-Fluoren-9-yl)oxy)trimethylsilane (**3x**) 20
(1-(4-Fluorophenyl)ethoxy)trimethylsilane (**3y**) 21
(1-(4-Chlorophenyl)ethoxy)trimethylsilane (**3z**) 21
(1-(4-Bromophenyl)ethoxy)trimethylsilane (**3aa**) 21
1-Trimethylsiloxyadamantane (**3ab**) 21
Bis(trimethylsilyl) 4-methylolphenol (**3ac**) 22
(2,6-Dimethylphenoxy)trimethylsilane (**3ad**) 22
4-Chloro-3,5-dimethylphenoxytrimethylsilane (**3ae**) 22
(4-Bromophenoxy)trimethylsilane (**3af**) 23
((3-Fluorobenzyl)oxy)diisopropylsilane (**3ag**) 23
tert-Butyldimethyl(undec-10-en-1-yloxy)silane (**3ah**) 23
1,1,1-Trimethyl-3,3,3-tripropyldisiloxane (**4a**) 24
1,1,1-Triisobutyl-3,3,3-trimethyldisiloxane (**4b**) 24
1,1,1-Triisopropyl-3,3,3-trimethyldisiloxane (**4c**) 24
Tri-*tert*-butyl (trimethylsilyl) silicate (**4d**) 25
Triisobutyl((4,4,5,5-tetramethyl-1,3,2-dioxaborolan-2-yl)oxy)silane (**5a**) 25
tert-Butyldimethyl((4,4,5,5-tetramethyl-1,3,2-dioxaborolan-2-yl)oxy)silane (**5b**) 25
1-Methoxy-4-(4-methylpent-4-en-2-yl)benzene (**6a**) 26
(1-Cyclopropyl-3-methylbut-3-en-1-yl)benzene (**6b**) 26
Trimethyl(octylthio)silane (**7a**) 26
1,1,1-Triisopropyl-3,3,3-trimethyldisilathiane (**7b**) 26
SPECTRA FOR ALL PRODUCTS 28
(Benzyloxy)trimethylsilane (**3a**) 29
Figure S1. ^{1}H NMR (400 MHz, Chloroform-d, 25°C) of (benzyloxy)trimethylsilane (**3a**) 29
Figure S2. ^{13}C{^{1}H} NMR (101 MHz, Chloroform-d, 25°C) of (benzyloxy)trimethylsilane (**3a**). 29
Figure S3. ^{29}Si NMR (79 MHz, Chloroform-d, 25°C) of (benzyloxy)trimethylsilane (**3a**) 30
((3-Fluorobenzyl)oxy)trimethylsilane (**3b**) 31
Figure S4. ^{1}H NMR (400 MHz, Chloroform-d, 25°C) of ((3-fluorobenzyl)oxy)trimethylsilane (**3b**). 31
Figure S5. ^{13}C{^{1}H} NMR (101 MHz, Chloroform-d, 25°C) of ((3-fluorobenzyl)oxy)trimethylsilane (**3b**). 31
Figure S6. ^{19}F NMR (377 MHz, Chloroform-d, 25°C) of ((3-fluorobenzyl)oxy)trimethylsilane (**3b**). 32
Figure S7. ^{29}Si NMR (79 MHz, Chloroform-d, 25°C) of ((3-fluorobenzyl)oxy)trimethylsilane (**3b**). 32

((2-Fluorobenzyl)oxy)trimethylsilane (**3c**) 33
Figure S8. ^{1}H NMR (400 MHz, Chloroform-d, 25°C) of ((2-fluorobenzyl)oxy)trimethylsilane (**3c**)........ 33
Figure S9. ^{13}C NMR (101 MHz, Chloroform-d, 25°C) of ((2-fluorobenzyl)oxy)trimethylsilane (**3c**). 33
Figure S10. ^{19}F NMR (377 MHz, Chloroform-d, 25°C) of ((2-fluorobenzyl)oxy)trimethylsilane (**3c**). 34
Figure S11. ^{29}Si NMR (79 MHz, Chloroform-d, 25°C) of ((2-fluorobenzyl)oxy)trimethylsilane (**3c**). 34
((3-Iodobenzyl)oxy)trimethylsilane (**3d**) 35
Figure S12. ^{1}H NMR (400 MHz, Chloroform-d, 25°C) of ((3-iodobenzyl)oxy)trimethylsilane (**3d**). 35
Figure S13. ^{13}C NMR (101 MHz, Chloroform-d, 25°C) of ((3-iodobenzyl)oxy)trimethylsilane (**3d**). 35
Figure S14. ^{29}Si NMR (79 MHz, Chloroform-d, 25°C) of ((3-iodobenzyl)oxy)trimethylsilane (3d). 36
((3,5-Dichlorobenzyl)oxy)trimethylsilane (**3e**) 37
Figure S15. ^{1}H NMR (400 MHz, Chloroform-d, 25°C) of ((3,5-dichlorobenzyl)oxy)trimethylsilane (**3e**). 37
Figure S16. ^{13}C NMR (101 MHz, Chloroform-d, 25°C) of ((3,5-dichlorobenzyl)oxy)trimethylsilane (**3e**)........ 37
Figure S17. ^{29}Si NMR (79 MHz, Chloroform-d, 25°C) of ((3,5-dichlorobenzyl)oxy)trimethylsilane (**3e**). 38
Trimethyl((4-(trifluoromethyl)benzyl)oxy)silane (**3f**) 39
Figure S18. ^{1}H NMR (400 MHz, Chloroform-d, 25°C) of trimethyl((4-(trifluoromethyl)benzyl)oxy)silane (**3f**)........ 39
Figure S19. ^{13}C NMR (101 MHz, Chloroform-d, 25°C) of trimethyl((4-(trifluoromethyl)benzyl)oxy)silane (**3f**). 39
Figure S20. ^{19}F NMR (377 MHz, Chloroform-d, 25°C) of trimethyl((4-(trifluoromethyl)benzyl)oxy)silane (**3f**)........ 40
Figure S21. ^{29}Si NMR (79 MHz, Chloroform-d, 25°C) of trimethyl((4-(trifluoromethyl)benzyl)oxy)silane (**3f**)........ 40
4-(((Trimethylsilyl)oxy)methyl)benzonitrile (**3g**)........ 41
Figure S22. ^{1}H NMR (400 MHz, Chloroform-d, 25°C) of 4-(((trimethylsilyl)oxy)methyl)benzonitrile (**3g**)........ 41
Figure S23. ^{13}C NMR (101 MHz, Chloroform-d, 25°C) of 4-(((trimethylsilyl)oxy)methyl)benzonitrile (**3g**) 41
Figure S24. ^{19}F NMR (377 MHz, Chloroform-d, 25°C) of 4-(((trimethylsilyl)oxy)methyl)benzonitrile (**3g**)........ 42
Figure S25. ^{29}Si NMR (79 MHz, Chloroform-d, 25°C) of 4-(((trimethylsilyl)oxy)methyl)benzonitrile (**3g**)........ 42
(*S*)-(3-Chloro-1-phenylpropoxy)trimethylsilane (**3h**) 43
Figure S26. ^{1}H NMR (400 MHz, Chloroform-d, 25°C) of (*S*)-(3-chloro-1-phenylpropoxy)trimethylsilane (**3h**). 43
Figure S27. ^{13}C NMR (101 MHz, Chloroform-d, 25°C) of (*S*)-(3-chloro-1-phenylpropoxy)trimethylsilane (**3h**)........ 43
Figure S28. ^{29}Si NMR (79 MHz, Chloroform-d, 25°C) of (*S*)-(3-chloro-1-phenylpropoxy)trimethylsilane (**3h**). 44
((2,3-Dimethylbenzyl)oxy)trimethylsilane (**3i**) 45
Figure S29. ^{1}H NMR (400 MHz, Chloroform-d, 25°C) of ((2,3-dimethylbenzyl)oxy)trimethylsilane (**3i**). 45
Figure S30. ^{13}C NMR (101 MHz, Chloroform-d, 25°C) of ((2,3-dimethylbenzyl)oxy)trimethylsilane (**3i**)........ 45
Figure S31. ^{29}Si NMR (79 MHz, Chloroform-d, 25°C) of ((2,3-dimethylbenzyl)oxy)trimethylsilane (**3i**). 46
Trimethyl(naphthalen-2-ylmethoxy)silane (**3j**) 47
Figure S32. ^{1}H NMR (400 MHz, Chloroform-d, 25°C) of trimethyl(naphthalen-2-ylmethoxy)silane (**3j**)........ 47
Figure S33. ^{13}C NMR (101 MHz, Chloroform-d, 25°C) of trimethyl(naphthalen-2-ylmethoxy)silane (**3j**). 47
Figure S34. ^{29}Si NMR (79 MHz, Chloroform-d, 25°C) of trimethyl(naphthalen-2-ylmethoxy)silane (**3j**)........ 48
((4-Ethynylbenzyl)oxy)trimethylsilane (**3k**)........ 49
Figure S35. ^{1}H NMR (400 MHz, Chloroform-d, 25°C) of ((4-ethynylbenzyl)oxy)trimethylsilane (**3k**)........ 49
Figure S36. ^{13}C NMR (101 MHz, Chloroform-d, 25°C) of ((4-ethynylbenzyl)oxy)trimethylsilane (**3k**). 49
Figure S37. ^{29}Si NMR (79 MHz, Chloroform-d, 25°C) of ((4-ethynylbenzyl)oxy)trimethylsilane (**3k**)........ 50
Trimethyl((3-phenylprop-2-yn-1-yl)oxy)silane (**3l**)........ 51
Figure S38. ^{1}H NMR (400 MHz, Chloroform-d, 25°C) of trimethyl((3-phenylprop-2-yn-1-yl)oxy)silane (**3l**). 51
Figure S39. ^{13}C NMR (101 MHz, Chloroform-d, 25°C) of trimethyl((3-phenylprop-2-yn-1-yl)oxy)silane (**3l**). 51
Figure S40. ^{29}Si NMR (79 MHz, Chloroform-d, 25°C) of trimethyl((3-phenylprop-2-yn-1-yl)oxy)silane (**3l**)........ 52
Trimethyl(oct-1-yn-3-yloxy)silane (**3m**)........ 53
Figure S41. ^{1}H NMR (400 MHz, Chloroform-d, 25°C) of trimethyl(oct-1-yn-3-yloxy)silane (**3m**). 53

Figure S42. ^{13}C NMR (101 MHz, Chloroform-d, 25°C) of trimethyl(oct-1-yn-3-yloxy)silane (**3m**). 53
Figure S43. ^{29}Si NMR (79 MHz, Chloroform-d, 25°C) of trimethyl(oct-1-yn-3-yloxy)silane (**3m**). 54
(Dec-9-en-1-yloxy)trimethylsilane (**3n**) 55
Figure S44. ^{1}H NMR (400 MHz, Chloroform-d, 25°C) of (dec-9-en-1-yloxy)trimethylsilane (**3n**). 55
Figure S45. ^{13}C NMR (101 MHz, Chloroform-d, 25°C) of (dec-9-en-1-yloxy)trimethylsilane (**3n**). 55
Figure S46. ^{29}Si NMR (79 MHz, Chloroform-d, 25°C) of (dec-9-en-1-yloxy)trimethylsilane (**3n**). 56
Trimethyl(undec-10-en-1-yloxy)silane (**3o**) 57
Figure S47. ^{1}H NMR (400 MHz, Chloroform-d, 25°C) of trimethyl(undec-10-en-1-yloxy)silane (**3o**). 57
Figure S48. ^{13}C NMR (101 MHz, Chloroform-d, 25°C) of trimethyl(undec-10-en-1-yloxy)silane (**3o**). 57
Figure S49. ^{29}Si NMR (79 MHz, Chloroform-d, 25°C) of trimethyl(undec-10-en-1-yloxy)silane (**3o**). 58
(Cinnamyloxy)trimethylsilane (**3p**) 59
Figure S50. ^{1}H NMR (400 MHz, Chloroform-d, 25°C) of (cinnamyloxy)trimethylsilane (**3p**). 59
Figure S51. ^{13}C NMR (101 MHz, Chloroform-d, 25°C) of (cinnamyloxy)trimethylsilane (**3p**). 59
Figure S52. ^{29}Si NMR (79 MHz, Chloroform-d, 25°C) of (cinnamyloxy)trimethylsilane (**3p**). 60
(*E*)-((3,7-dimethylocta-2,6-dien-1-yl)oxy)trimethylsilane (**3q**) 61
Figure S53. ^{1}H NMR (400 MHz, Chloroform-d, 25°C) of (*E*)-((3,7-dimethylocta-2,6-dien-1-yl)oxy)trimethylsilane (**3q**). 61
Figure S54. ^{13}C NMR (101 MHz, Chloroform-d, 25°C) of (*E*)-((3,7-dimethylocta-2,6-dien-1-yl)oxy)trimethylsilane (**3q**). 61
Figure S55. ^{29}Si NMR (79 MHz, Chloroform-d, 25°C) of (*E*)-((3,7-dimethylocta-2,6-dien-1-yl)oxy)trimethylsilane (**3q**). 62
(Cyclohexylmethoxy)trimethylsilane (**3r**) 63
Figure S56. ^{1}H NMR (400 MHz, Chloroform-d, 25°C) of (cyclohexylmethoxy)trimethylsilane (**3r**). 63
Figure S57. ^{13}C NMR (101 MHz, Chloroform-d, 25°C) of (cyclohexylmethoxy)trimethylsilane (**3r**). 63
Figure S58. ^{29}Si NMR (79 MHz, Chloroform-d, 25°C) of (cyclohexylmethoxy)trimethylsilane (**3r**). 64
(2-Cyclohexylethoxy)trimethylsilane (**3s**) 65
Figure S59. ^{1}H NMR (400 MHz, Chloroform-d, 25°C) of (2-cyclohexylethoxy)trimethylsilane (**3s**). 65
Figure S60. ^{13}C NMR (101 MHz, Chloroform-d, 25°C) of (2-cyclohexylethoxy)trimethylsilane (**3s**). 65
Figure S61. ^{29}Si NMR (79 MHz, Chloroform-d, 25°C) of (2-cyclohexylethoxy)trimethylsilane (**3s**). 66
((9-Bromononyl)oxy)trimethylsilane (**3t**) 67
Figure S62. ^{1}H NMR (400 MHz, Chloroform-d, 25°C) of ((9-bromononyl)oxy)trimethylsilane (**3t**). 67
Figure S63. ^{13}C NMR (101 MHz, Chloroform-d, 25°C) of ((9-bromononyl)oxy)trimethylsilane (**3t**). 67
Figure S64. ^{29}Si NMR (79 MHz, Chloroform-d, 25°C) of ((9-bromononyl)oxy)trimethylsilane (**3t**). 68
2,2,10,10-Tetramethyl-3,9-dioxa-2,10-disilaundecane (**3u**) 69
Figure S65. ^{1}H NMR (400 MHz, Chloroform-d, 25°C) of 2,2,10,10-tetramethyl-3,9-dioxa-2,10-disilaundecane (**3u**). 69
Figure S66. ^{13}C NMR (101 MHz, Chloroform-d, 25°C) of 2,2,10,10-tetramethyl-3,9-dioxa-2,10-disilaundecane (**3u**). 69
Figure S67. ^{29}Si NMR (79 MHz, Chloroform-d, 25°C) of 2,2,10,10-tetramethyl-3,9-dioxa-2,10-disilaundecane (**3u**). 70
(Benzo[d][1,3]dioxol-5-ylmethoxy)trimethylsilane (**3v**) 71
Figure S68. ^{1}H NMR (400 MHz, Chloroform-d, 25°C) of (benzo[*d*][1,3]dioxol-5-ylmethoxy)trimethylsilane (**3v**). 71
Figure S69. ^{13}C NMR (101 MHz, Chloroform-d, 25°C) of (benzo[*d*][1,3]dioxol-5-ylmethoxy)trimethylsilane (**3v**). 71
Figure S70. ^{29}Si NMR (79 MHz, Chloroform-d, 25°C) of (benzo[*d*][1,3]dioxol-5-ylmethoxy)trimethylsilane (**3v**). 72
6-(((Trimethylsilyl)oxy)methyl)benzo[d]thiazole (**3w**) 73
Figure S71. ^{1}H NMR (400 MHz, Chloroform-d, 25°C) of 6-(((trimethylsilyl)oxy)methyl)benzo[*d*]thiazole (**3w**). 73
Figure S72. ^{13}C NMR (101 MHz, Chloroform-d, 25°C) of 6-(((trimethylsilyl)oxy)methyl)benzo[*d*]thiazole (**3w**). 73
Figure S73. ^{29}Si NMR (79 MHz, Chloroform-d, 25°C) of 6-(((trimethylsilyl)oxy)methyl)benzo[*d*]thiazole (**3w**). 74
((*9H*-Fluoren-9-yl)oxy)trimethylsilane (**3x**) 75
Figure S74. ^{1}H NMR (400 MHz, Chloroform-d, 25°C) of ((*9H*-fluoren-9-yl)oxy)trimethylsilane (**3x**). 75
Figure S75. ^{13}C NMR (101 MHz, Chloroform-d, 25°C) of ((*9H*-fluoren-9-yl)oxy)trimethylsilane (**3x**). 75

Figure S76. ^{29}Si NMR (79 MHz, Chloroform-d, 25°C) of ((*9H*-fluoren-9-yl)oxy)trimethylsilane (**3x**). 76
(1-(4-Fluorophenyl)ethoxy)trimethylsilane (**3y**) 77
Figure S77. ^{1}H NMR (400 MHz, Chloroform-d, 25°C) of (1-(4-fluorophenyl)ethoxy)trimethylsilane (**3y**). 77
Figure S78. ^{13}C NMR (101 MHz, Chloroform-d, 25°C) of (1-(4-fluorophenyl)ethoxy)trimethylsilane (**3y**). 77
Figure S79. ^{19}F NMR (377 MHz, Chloroform-d, 25°C) of (1-(4-fluorophenyl)ethoxy)trimethylsilane (**3y**). 78
Figure S80. ^{29}Si NMR (79 MHz, Chloroform-d, 25°C) of (1-(4-fluorophenyl)ethoxy)trimethylsilane (**3y**). 78
(1-(4-Chlorophenyl)ethoxy)trimethylsilane (**3z**) 79
Figure S81. ^{1}H NMR (400 MHz, Chloroform-d, 25°C) of (1-(4-chlorophenyl)ethoxy)trimethylsilane (**3z**). 79
Figure S82. ^{13}C NMR (101 MHz, Chloroform-d, 25°C) of (1-(4-chlorophenyl)ethoxy)trimethylsilane (**3z**). 79
Figure S83. ^{29}Si NMR (79 MHz, Chloroform-d, 25°C) of (1-(4-chlorophenyl)ethoxy)trimethylsilane (**3z**). 80
(1-(4-Bromophenyl)ethoxy)trimethylsilane (**3aa**) 81
Figure S84. ^{1}H NMR (400 MHz, Chloroform-d, 25°C) of (1-(4-bromophenyl)ethoxy)trimethylsilane (**3aa**). 81
Figure S85. ^{13}C NMR (101 MHz, Chloroform-d, 25°C) of (1-(4-bromophenyl)ethoxy)trimethylsilane (**3aa**). 81
Figure S86. ^{29}Si NMR (79 MHz, Chloroform-d, 25°C) of (1-(4-bromophenyl)ethoxy)trimethylsilane (**3aa**). 82
1-Trimethylsiloxyadamantane (**3ab**) 83
Figure S87. ^{1}H NMR (400 MHz, Chloroform-d, 25°C) of 1-trimethylsiloxyadamantane (**3ab**). 83
Figure S88. ^{13}C NMR (101 MHz, Chloroform-d, 25°C) of 1-trimethylsiloxyadamantane (**3ab**). 83
Figure S89. ^{29}Si NMR (79 MHz, Chloroform-d, 25°C) of 1-trimethylsiloxyadamantane (**3ab**). 84
Bis(trimethylsilyl) 4-methylolphenol (**3ac**) 85
Figure S90. ^{1}H NMR (400 MHz, Chloroform-d, 25°C) of bis(trimethylsilyl) 4-methylolphenol (**3ac**). 85
Figure S91. ^{13}C NMR (101 MHz, Chloroform-d, 25°C) of bis(trimethylsilyl) 4-methylolphenol (**3ac**). 85
Figure S92. ^{29}Si NMR (79 MHz, Chloroform-d, 25°C) of bis(trimethylsilyl) 4-methylolphenol (**3ac**). 86
(2,6-Dimethylphenoxy)trimethylsilane (**3ad**) 87
Figure S93. ^{1}H NMR (400 MHz, Chloroform-d, 25°C) of (2,6-dimethylphenoxy)trimethylsilane (**3ad**). 87
Figure S94. ^{13}C NMR (101 MHz, Chloroform-d, 25°C) of (2,6-dimethylphenoxy)trimethylsilane (**3ad**). 87
Figure S95. ^{29}Si NMR (79 MHz, Chloroform-d, 25°C) of (2,6-dimethylphenoxy)trimethylsilane (**3ad**). 88
4-Chloro-3,5-dimethylphenoxytrimethylsilane (**3ae**) 89
Figure S96. ^{1}H NMR (400 MHz, Chloroform-d, 25°C) of 4-chloro-3,5-dimethylphenoxytrimethylsilane (**3ae**). 89
Figure S97. ^{13}C NMR (101 MHz, Chloroform-d, 25°C) of 4-chloro-3,5-dimethylphenoxytrimethylsilane (**3ae**). 89
Figure S98. ^{29}Si NMR (79 MHz, Chloroform-d, 25°C) of 4-chloro-3,5-dimethylphenoxytrimethylsilane (**3ae**). 90
(4-Bromophenoxy)trimethylsilane (**3af**) 91
Figure S99. ^{1}H NMR (400 MHz, Chloroform-d, 25°C) of (4-bromophenoxy)trimethylsilane (**3af**). 91
Figure S100. ^{13}C NMR (101 MHz, Chloroform-d, 25°C) of (4-bromophenoxy)trimethylsilane (**3af**). 91
Figure S101. ^{29}Si NMR (79 MHz, Chloroform-d, 25°C) of (4-bromophenoxy)trimethylsilane (**3af**). 92
((3-Fluorobenzyl)oxy)diisopropylsilane (**3ag**) 93
Figure S101. ^{1}H NMR (400 MHz, Chloroform-d, 25°C) of ((3-fluorobenzyl)oxy)diisopropylsilane (**3ag**). 93
Figure S102. ^{13}C NMR (101 MHz, Chloroform-d, 25°C) of ((3-fluorobenzyl)oxy)diisopropylsilane (**3ag**). 93
Figure S103. ^{19}F NMR (377 MHz, Chloroform-d, 25°C) of ((3-fluorobenzyl)oxy)diisopropylsilane (**3ag**). 94
Figure S104. ^{29}Si NMR (79 MHz, Chloroform-d, 25°C) of ((3-fluorobenzyl)oxy)diisopropylsilane (**3ag**). 94
tert-Butyldimethyl(undec-10-en-1-yloxy)silane (**3ah**) 95
Figure S105. ^{1}H NMR (400 MHz, Chloroform-d, 25°C) of *tert*-butyldimethyl(undec-10-en-1-yloxy)silane (**3ah**). 95
Figure S106. ^{13}C NMR (101 MHz, Chloroform-d, 25°C) of *tert*-butyldimethyl(undec-10-en-1-yloxy)silane (**3ah**). 95
Figure S107. ^{29}Si NMR (101 MHz, Chloroform-d, 25°C) of *tert*-butyldimethyl(undec-10-en-1-yloxy)silane (**3ah**). 96
1,1,1-Trimethyl-3,3,3-tripropyldisiloxane (**4a**) 97
Figure S108. ^{1}H NMR (400 MHz, Chloroform-d, 25°C) of 1,1,1-trimethyl-3,3,3-tripropyldisiloxane (**4a**). 97

Figure S109. ^{13}C NMR (101 MHz, Chloroform-d, 25°C) of 1,1,1-trimethyl-3,3,3-tripropyldisiloxane (**4a**). 97
Figure S110. ^{29}Si NMR (79 MHz, Chloroform-d, 25°C) of 1,1,1-trimethyl-3,3,3-tripropyldisiloxane (**4a**). 98
1,1,1-Triisobutyl-3,3,3-trimethyldisiloxane (**4b**) 99
Figure S111. ^{1}H NMR (400 MHz, Chloroform-d, 25°C) of 1,1,1-triisobutyl-3,3,3-trimethyldisiloxane (**4b**). 99
Figure S112. ^{13}C NMR (101 MHz, Chloroform-d, 25°C) of 1,1,1-triisobutyl-3,3,3-trimethyldisiloxane (**4b**). 99
Figure S113. ^{29}Si NMR (79 MHz, Chloroform-d, 25°C) of 1,1,1-triisobutyl-3,3,3-trimethyldisiloxane (**4b**). 100
1,1,1-Triisopropyl-3,3,3-trimethyldisiloxane (**4c**) 101
Figure S114. ^{1}H NMR (400 MHz, Chloroform-d, 25°C) of 1,1,1-triisopropyl-3,3,3-trimethyldisiloxane (**4c**). 101
Figure S115. ^{13}C NMR (101 MHz, Chloroform-d, 25°C) of 1,1,1-triisopropyl-3,3,3-trimethyldisiloxane (**4c**). 101
Figure S116. ^{29}Si NMR (79 MHz, Chloroform-d, 25°C) of 1,1,1-triisopropyl-3,3,3-trimethyldisiloxane (**4c**). 102
Tri-*tert*-butyl (trimethylsilyl) silicate (**4d**) 103
Figure S117. ^{1}H NMR (400 MHz, Chloroform-d, 25°C) of tri-tert-butyl (trimethylsilyl) silicate (**4d**). 103
Figure S118. ^{13}C NMR (101 MHz, Chloroform-d, 25°C) of tri-tert-butyl (trimethylsilyl) silicate (**4d**). 103
Figure S119. ^{1}H NMR (400 MHz, Chloroform-d, 25°C) of tri-tert-butyl (trimethylsilyl) silicate (**4d**). 104
Triisobutyl((4,4,5,5-tetramethyl-1,3,2-dioxaborolan-2-yl)oxy)silane (**5a**) 105
Figure S120. ^{1}H NMR (400 MHz, Chloroform-d, 25°C) of triisobutyl((4,4,5,5-tetramethyl-1,3,2-dioxaborolan-2-yl)oxy)silane (**5a**). 105
Figure S121. ^{13}C NMR (101 MHz, Chloroform-d, 25°C) of triisobutyl((4,4,5,5-tetramethyl-1,3,2-dioxaborolan-2-yl)oxy)silane (**5a**). 105
Figure S122. ^{11}B NMR (128 MHz, Chloroform-d, 25°C) of triisobutyl((4,4,5,5-tetramethyl-1,3,2-dioxaborolan-2-yl)oxy)silane (**5a**). 106
Figure S123. ^{29}Si NMR (79 MHz, Chloroform-d, 25°C) of triisobutyl((4,4,5,5-tetramethyl-1,3,2-dioxaborolan-2-yl)oxy)silane (**5a**). 106
tert-Butyldimethyl((4,4,5,5-tetramethyl-1,3,2-dioxaborolan-2-yl)oxy)silane (**5b**) 107
Figure S124. ^{1}H NMR (400 MHz, Chloroform-d, 25°C) of *tert*-butyldimethyl((4,4,5,5-tetramethyl-1,3,2-dioxaborolan-2-yl)oxy)silane (**5b**). 107
Figure S125. ^{13}C NMR (101 MHz, Chloroform-d, 25°C) of *tert*-butyldimethyl((4,4,5,5-tetramethyl-1,3,2-dioxaborolan-2-yl)oxy)silane (**5b**). 107
Figure S126. ^{11}B NMR (128 MHz, Chloroform-d, 25°C) of *tert*-butyldimethyl((4,4,5,5-tetramethyl-1,3,2-dioxaborolan-2-yl)oxy)silane (**5b**). 108
Figure S127. ^{29}Si NMR (79 MHz, Chloroform-d, 25°C) of *tert*-butyldimethyl((4,4,5,5-tetramethyl-1,3,2-dioxaborolan-2-yl)oxy)silane (**5b**). 108
1-Methoxy-4-(4-methylpent-4-en-2-yl)benzene (**6a**) 109
Figure S128. ^{1}H NMR (400 MHz, Chloroform-d, 25°C) of 1-methoxy-4-(4-methylpent-4-en-2-yl)benzene (**6a**). 109
Figure S129. ^{13}C NMR (101 MHz, Chloroform-d, 25°C) of 1-methoxy-4-(4-methylpent-4-en-2-yl)benzene (**6a**). 109
(1-Cyclopropyl-3-methylbut-3-en-1-yl)benzene (**6b**) 110
Figure S130. ^{1}H NMR (400 MHz, Chloroform-d, 25°C) of (1-cyclopropyl-3-methylbut-3-en-1-yl)benzene (**6b**). 110
Figure S131. ^{13}C NMR (101 MHz, Chloroform-d, 25°C) of (1-cyclopropyl-3-methylbut-3-en-1-yl)benzene (**6b**). 110
Trimethyl(octylthio)silane (**7a**) 111
Figure S132. ^{1}H NMR (400 MHz, Chloroform-d, 25°C) of trimethyl(octylthio)silane (**7a**). 111
Figure S133. ^{13}C NMR (101 MHz, Chloroform-d, 25°C) of trimethyl(octylthio)silane (**7a**). 111
Figure S134. ^{29}Si NMR (79 MHz, Chloroform-d, 25°C) of trimethyl(octylthio)silane (**7a**). 112
1,1,1-Triisopropyl-3,3,3-trimethyldisilathiane (**7b**) 113
Figure S135. ^{1}H NMR (400 MHz, Chloroform-d, 25°C) of 1,1,1-triisopropyl-3,3,3-trimethyldisilathiane (**7b**). 113
Figure S136. ^{1}H NMR (400 MHz, Chloroform-d, 25°C) of 1,1,1-triisopropyl-3,3,3-trimethyldisilathiane (**7b**). 113
Figure S137. ^{29}Si NMR (79 MHz, Chloroform-d, 25°C) of 1,1,1-triisopropyl-3,3,3-trimethyldisilathiane (**7b**). 114
MECHANISTIC STUDIES NMR 115
Hexafluoroisopropanol 116

Figure S137. ^{1}H NMR (400 MHz, Chloroform-d, 25°C) of HFIP. 116
Figure S138. ^{19}F NMR (377 MHz, Chloroform-d, 25°C) of HFIP. 116
3-Fluorobenzyl alcohol (**1b**) 117
Figure S139. ^{1}H NMR (400 MHz, Chloroform-d, 25°C) of 3-fluorobenzyl alcohol (**1b**). 117
2-Methylallyltrimethylsilane (**2b**) 118
Figure S140. ^{1}H NMR (400 MHz, Chloroform-d, 25°C) of 2-methylallyltrimethylsilane (**2b**). 118
HFIP + **1b** (normal) 118
Figure S141. ^{1}H NMR (400 MHz, Chloroform-d, 25°C) of mixture of HFIP and 3-fluorobenzyl alcohol (**1b**). 118
HFIP + **2b** (normal) 119
Figure S142. ^{19}F NMR (377 MHz, Chloroform-d, 25°C) of mixture of HFIP and 2-methylallyltrimethylsilane (**2b**). 119
HFIP + **1b** (stacked) 120
Figure S143. Stacked ^{1}H NMR (400 MHz, Chloroform-d, 25°C) of mixture of HFIP and 3-fluorobenzyl alcohol (**1b**). 120
HFIP + **2b** (stacked) 120
Figure S144. Stacked ^{1}H NMR (400 MHz, Chloroform-d, 25°C) of mixture of HFIP and 2-methylallyltrimethylsilane (**2b**). 120
Preliminary computational studies 121
References 122

GENERAL INFORMATION

2-Methylallyltrimethylsilane (**2b**) was obtained from multiple suppliers (Fluorochem, TCI, Merck, own synthesis[1]) and used as received, without further purification. Allyltrimethylsilane (**2a**) was obtained from multiple suppliers (TCI and Merck) and used as received, without further purification. Diisopropyl(2-methylallyl)silane (**2c**) and *tert*-butyldimethyl(2-methylallyl)silane (**2d**) were synthesized in accordance with previous literature.[2,3] Hexafluoroisopropanol was sourced from Fluorochem, TCI, Merck, or Chemat and used as received without additional purification or drying. Other solvents employed during reaction optimization were purchased from Honeywell, dried over calcium hydride (CaH_2), and purified by distillation prior to use. Alcohols, silanols, and thiols were obtained from commercial suppliers (Sigma-Aldrich, Ambeed, Fisher, Acros, Fluorochem, Angene, and TCI) and used as received. 2-Methylallylpinacolborane was synthesized in accordance with the literature.[4] Reaction progress – primarily the conversion of alcohols – was monitored by gas chromatography (GC) using a Bruker Scion 460-GC and an Agilent 5977B GC/MSD equipped with an Agilent 8860 GC system. The structures of products were determined by NMR spectroscopy, mass spectrometry (GC-MS). The ^{1}H NMR (400 MHz), ^{13}C NMR (101 MHz), ^{29}Si NMR (79 MHz), ^{19}F NMR (377 MHz), and ^{11}B (128 MHz) spectra were recorded on Bruker Avance III HD NanoBay spectrometer, using chloroform-d ($CDCl_3$) or benzene-d6 (C_6D_6) as the solvent. Deuterated solvent was purchased from Sigma Aldrich (Merck) ($CDCl_3$ 99.8 atom% D) and used as received. High resolution mass spectra (HRMS) were obtained using Impact HD mass spectrometer (Q-TOF type instrument equipped with electrospray ion source; Bruker Daltonics, Germany). The sample solutions (DCM:MeOH) were infused into the ESI source by a syringe pump (direct inlet) at the flow rate of 3 µL/min. The instrument was operated under the following optimized settings: end plate voltage 500 V; capillary voltage 4.2 kV; nebulizer pressure 0.3 bar; dry gas (nitrogen) temperature 200 °C; dry gas flow rate 4 L/min. The spectrometer was previously calibrated with the standard tune mixture.

GENERAL SYNTHETIC PROCEDURES

Synthesis of compounds **3a-3t**

To a 5 mL vial equipped with a magnetic stirring bar, alcohol (**1a**-**1t**, 1 mmol) was added. Then, 2-methylallyltrimethylsilane (**2b**, 2 mmol, 0.256 g), and HFIP (1 mL) have been placed in a vial. In most cases, the reaction required stirring for a period of 30 minutes.

After this time, HFIP and an excess of **2b** were evaporated under reduced pressure to give desired products. The pure products **3a**-**3t** were identified by ^{1}H NMR, ^{13}C NMR, ^{29}Si NMR, ^{19}F NMR spectroscopy, and mass spectrometry.

Synthesis of compound **3u**

To a 5 mL vial equipped with a magnetic stirring bar, pentane-1,5-diol (**1u**, 1 mmol, 0.104 g) was added. Then, 2-methylallyltrimethylsilane (**2b**, 4 mmol, 0.512 g), and HFIP (1 mL) have been placed in a vial. The reaction required stirring for a period of 30 minutes.

After this time, HFIP and an excess of **2b** were evaporated under reduced pressure to give desired product. The pure product **3u** was identified by ^{1}H NMR, ^{13}C NMR, ^{29}Si NMR spectroscopy, and mass spectrometry.

Synthesis of compound **3v**

To a 5 mL vial equipped with a magnetic stirring bar, piperonylalcohol (**1v**, 1 mmol, 0.152 g) was added. Then, 2-methylallyltrimethylsilane (**2b**, 3 mmol, 0.384 g), and HFIP (1 mL) have been placed in a vial. The reaction required stirring for a period of 30 minutes.

After this time, HFIP and an excess of **2b** were evaporated under reduced pressure to give desired product. The pure product **3v** was identified by ^{1}H NMR, ^{13}C NMR, ^{29}Si NMR spectroscopy, and mass spectrometry.

Synthesis of compound **3w**

To a 5 mL vial equipped with a magnetic stirring bar, alcohol (**1w**, 1 mmol) was added. Then, 2-methylallyltrimethylsilane (**2b**, 2 mmol, 0.256 g), and HFIP (1 mL) have been placed in a vial. In most cases, the reaction required stirring for a period of 30 minutes.

After this time, HFIP and an excess of **2b** were evaporated under reduced pressure to give desired product. The pure product **3w** was identified by ^{1}H NMR, ^{13}C NMR, ^{29}Si NMR, ^{19}F NMR spectroscopy, and mass spectrometry.

Synthesis of compound **3x**

To a 5 mL vial equipped with a magnetic stirring bar, 9H-fluoren-9-ol (**1x**, 1 mmol, 0.182 g) was added. Then, 2-methylallyltrimethylsilane (**2b**, 3 mmol, 0.384 g), and HFIP (1 mL) have been placed in a vial. The reaction required stirring for a period of 30 minutes.

After this time, HFIP and an excess of **2b** were evaporated under reduced pressure to give desired product. The pure product **3x** was identified by ^{1}H NMR, ^{13}C NMR, ^{29}Si NMR spectroscopy, and mass spectrometry.

Synthesis of compound **3y**

To a 5 mL vial equipped with a magnetic stirring bar, 1-(4-fluorophenyl)ethan-1-ol (**1y**, 1 mmol, 0.140 g) was added. Then, 2-methylallyltrimethylsilane (**2b**, 2 mmol, 0.256 g), and HFIP (1 mL) have been placed in a vial. The reaction required stirring for a period of 30 minutes.

After this time, HFIP and an excess of **2b** were evaporated under reduced pressure to give desired product. The pure product **3y** was identified by ^{1}H NMR, ^{13}C NMR, ^{29}Si NMR, ^{19}F NMR spectroscopy, and mass spectrometry.

Synthesis of compounds **3z-3aa**

To a 5 mL vial equipped with a magnetic stirring bar, alcohol (**1z** or **1aa**, 1 mmol) was added. Then, 2-methylallyltrimethylsilane (**2b**, 2 mmol, 0.256 g), and HFIP (1 mL) have been placed in a vial. The reaction required stirring for a period of 30 minutes.

After this time, HFIP and an excess of **2b** were evaporated under reduced pressure to give desired products. The pure products **3z-3aa** were identified by ^{1}H NMR, ^{13}C NMR, ^{29}Si NMR spectroscopy, and mass spectrometry.

Synthesis of compound **3ab**

To a 5 mL vial equipped with a magnetic stirring bar, 1-adamantol (**1ab**, 1 mmol, 0.152 g) was added. Then, 2-methylallyltrimethylsilane (**2b**, 3 mmol, 0.384 g), and HFIP (1 mL) have been placed in a vial. The reaction required stirring for a period of 30 minutes.

After this time, HFIP and an excess of **2b** were evaporated under reduced pressure to give desired product. The pure product **3ab** was identified by ^{1}H NMR, ^{13}C NMR, ^{29}Si NMR spectroscopy, and mass spectrometry.

Synthesis of compound **3ac**

To a 5 mL vial equipped with a magnetic stirring bar, 4-hydroxybenzyl alcohol (**1ac**, 1 mmol, 0.124 g) was added. Then, 2-methylallyltrimethylsilane (**2b**, 4 mmol, 0.512 g), and HFIP (1 mL) have been placed in a vial. The reaction required stirring for a period of 30 minutes.

After this time, HFIP and an excess of **2b** were evaporated under reduced pressure to give desired product. The pure product **3ac** was identified by ^{1}H NMR, ^{13}C NMR, ^{29}Si NMR spectroscopy, and mass spectrometry.

Synthesis of compounds **3ad-3af**

To a 5 mL vial equipped with a magnetic stirring bar, alcohol (**1ad-1af**, 1 mmol) was added. Then, 2-methylallyltrimethylsilane (**2b**, 2 mmol, 0.256 g), and HFIP (1 mL) have been placed in a vial. The reaction required stirring for a period of 30 minutes.

After this time, HFIP and an excess of **2b** were evaporated under reduced pressure to give desired products. The pure products **3ad-3af** were identified by ^{1}H NMR, ^{13}C NMR, ^{29}Si NMR spectroscopy, and mass spectrometry.

Synthesis of compound **3ag**

To a 5 mL vial equipped with a magnetic stirring bar, 3-fluorobenzyl alcohol (**1b**, 1 mmol, 0.126 g) was added. Then, diisopropyl(2-methylallyl)silane (**2c**, 2 mmol, 0.340 g), and HFIP (1 mL) have been placed in a vial. The reaction required stirring for a period of 60 minutes.

After this time, HFIP and an excess of **2c** were evaporated under reduced pressure to give desired product. The pure product **3ag** was identified by ^{1}H NMR, ^{13}C NMR, ^{29}Si NMR, ^{19}F NMR spectroscopy, and mass spectrometry.

Synthesis of compound **3ah**

To a 5 mL vial equipped with a magnetic stirring bar, undecane-1-ol (**1o**, 1 mmol, 0.172 g) was added. Then, *tert*-butyldimethyl(2-methylallyl)silane (**2d**, 2 mmol, 0.340 g), and HFIP (1 mL) have been placed in a vial. The reaction required stirring for a period of 60 minutes.

After this time, HFIP and an excess of **2d** were evaporated under reduced pressure to give desired product. The pure product **3ah** was identified by ^{1}H NMR, ^{13}C NMR, ^{29}Si NMR spectroscopy, and mass spectrometry.

Synthesis of compound **4a, 4b,** and **4d**

To a 5 mL vial equipped with a magnetic stirring bar, silanol (**1ah, 1ai, or 1ak**, 1 mmol) was added. Then, 2-methylallyltrimethylsilane (**2b**, 2 mmol, 0.256 g), and HFIP (1 mL) have been placed in a vial. The reaction required stirring for a period of 30 minutes.

After this time, HFIP and an excess of **2b** were evaporated under reduced pressure to give desired products. The pure products **4a, 4b** or **4d** were identified by ^{1}H NMR, ^{13}C NMR, ^{29}Si NMR spectroscopy, and mass spectrometry.

Synthesis of compound **4c**

To a 5 mL vial equipped with a magnetic stirring bar, triisopropylsilanol (**1aj**, 1 mmol, 0.158 g) was added. Then, 2-methylallyltrimethylsilane (**2b**, 4 mmol, 0.512 g), and HFIP (1 mL) have been placed in a vial. The reaction required stirring for a period of 30 minutes.

After this time, HFIP and an excess of **2b** were evaporated under reduced pressure to give desired product. The pure product **4c** was identified by ^{1}H NMR, ^{13}C NMR, ^{29}Si NMR spectroscopy, and mass spectrometry.

Synthesis of compound **5a** and **5b**

To a 5 mL vial equipped with a magnetic stirring bar, silanol (**1ai** or **1ak**, 1 mmol) was added. Then, 2-methylallylpinacolborane (**2e**, 2 mmol, 0.364 g), and HFIP (1 mL) have been placed in a vial. The reaction required stirring for a period of 60 minutes.

After this time, HFIP and an excess of **2e** were evaporated under reduced pressure to give desired products. The pure products **5a** and **5b** were identified by ^{1}H NMR, ^{13}C NMR, ^{29}Si NMR spectroscopy, and mass spectrometry.

Synthesis of compound **6a** and **6b**

To a 5 mL vial equipped with a magnetic stirring bar, alcohol (**1al** or **1am**, 1 mmol) was added. Then, 2-methylallyltrimethylsilane (**2b**, 2 mmol, 0.256 g), and HFIP (1 mL) have been placed in a vial. The reaction required stirring for a period of 30 minutes.

After this time, HFIP and an excess of **2b** were evaporated under reduced pressure to give desired products. The pure products **6a** and **6b** were identified by ^{1}H NMR, ^{13}C NMR, ^{29}Si NMR spectroscopy, and mass spectrometry.

Synthesis of compound **7a** and **7b**

To a 5 mL vial equipped with a magnetic stirring bar, thiol (**1an** or **1ao**, 1 mmol) was added. Then, 2-methylallyltrimethylsilane (**2b**, 3 mmol, 0.384 g), and HFIP (1 mL) have been placed in a vial. The reaction required stirring for a period of 60 minutes.

After this time, HFIP and an excess of **2b** were evaporated under reduced pressure to give desired products. The pure products **7a** and **7b** were identified by ^{1}H NMR, ^{13}C NMR, ^{29}Si NMR spectroscopy, and mass spectrometry.

Scaled-up synthesis of **3a**

To a 20 mL vial equipped with a magnetic stirring bar, benzyl alcohol (**1a**, 10 mmol, 1.08 g) was added. Then, 2-methylallyltrimethylsilane (**2b**, 20 mmol, 2.56 g), and HFIP (10 mL) have been placed in a vial. The reaction required stirring for a period of 30 minutes.

After this time, HFIP and an excess of **2b** were evaporated under reduced pressure to give desired product. The pure product **3a** was obtained in 97% yield (1.75 g)

Scaled-up synthesis of **3f**

To a 20 mL vial equipped with a magnetic stirring bar, benzyl alcohol (**1f**, 10 mmol, 1.76 g) was added. Then, 2-methylallyltrimethylsilane (**2b**, 20 mmol, 2.56 g), and HFIP (10 mL) have been placed in a vial. The reaction required stirring for a period of 30 minutes.

After this time, HFIP and an excess of **2b** were evaporated under reduced pressure to give desired product. The pure product **3f** was obtained in 95% yield (2.36 g).

Experiment in the presence of radical scavenger

To a 5 mL vial equipped with a magnetic stirring bar, TEMPO (1 mmol, 0.156 g) was added. Then, benzyl alcohol (**1a**, 1 mmol, 0.108 g), 2-methylallyltrimethylsilane (**2b**, 2 mmol, 0.256 g), and HFIP (1 mL) have been placed in a vial. The reaction required stirring for a period of 30 minutes. The conversion of **1a** to **3a** was 96%.

CHARACTERIZATION DATA FOR ALL PRODUCTS

(Benzyloxy)trimethylsilane (**3a**)

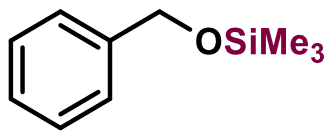

(Benzyloxy)trimethylsilane was obtained in 96% yield as an oil (0.178 g). The title compound was known in the literature, and all spectroscopic data are in agreement.[5]

^{1}H NMR: (400 MHz, $CDCl_3$) δ 7.23 (d, *J* = 4.6 Hz, 4H), 7.18 – 7.12 (m, 1H), 4.60 (s, 2H), 0.06 (s, 9H).

^{13}C{^{1}H} NMR: (101 MHz, $CDCl_3$) δ 141.1, 128.4, 127.3, 126.7, 64.8, -0.3.

^{29}Si NMR: (79 MHz, $CDCl_3$) δ 19.2.

EI-MS m/z (rel. int.): 180 ([M]$^+$, 15%), 165 (100), 135 (75), 91 (95).

((3-Fluorobenzyl)oxy)trimethylsilane (**3b**)

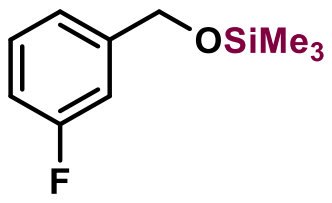

((3-Fluorobenzyl)oxy)trimethylsilane was obtained in 97% yield as an oil (0.192 g). The title compound is a new compound.

^{1}H NMR: (400 MHz, $CDCl_3$) δ 7.26 – 7.19 (m, 1H), 7.07 – 6.98 (m, 2H), 6.93 – 6.83 (m, 1H), 4.64 (s, 2H), 0.12 (s, 9H).

^{13}C{^{1}H} NMR: (101 MHz, $CDCl_3$) δ 163.5 (d, *J* = 245.3 Hz), 144.3 (d, *J* = 7.1 Hz), 130.2 (d, *J* = 8.1 Hz), 122.2 (d, *J* = 2.9 Hz), 114.3 (d, *J* = 21.2 Hz), 113.7 (d, *J* = 21.9 Hz), 64.3, -0.0.

^{19}F NMR: (377 MHz, $CDCl_3$) δ -113.5.

^{29}Si NMR: (79 MHz, $CDCl_3$) δ 19.7.

EI-MS m/z (rel. int.): 198 ([M]$^+$, 5%), 183 (80), 153 (50), 109 (100).

HRMS (ESI-TOF) m/z: [M + Na]$^+$ Calcd for $[C_{10}H_{15}FNaOSi]^+$ 221.0768; Found 221.0762.

((2-Fluorobenzyl)oxy)trimethylsilane (**3c**)

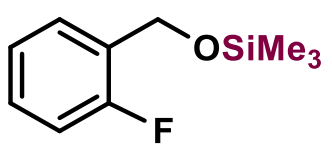

((2-Fluorobenzyl)oxy)trimethylsilane was obtained in 94% yield as an oil (0.186 g). The title compound was known in the literature, and all spectroscopic data are in agreement.[6]

^{1}H NMR: (400 MHz, $CDCl_3$) δ 7.51 – 7.42 (m, 1H), 7.26 – 7.20 (m, 1H), 7.18 – 7.09 (m, 1H), 7.07 – 6.96 (m, 1H), 4.77 (s, 2H), 0.17 (s, 9H).

^{13}C{^{1}H} NMR: (101 MHz, $CDCl_3$) δ 160.48 (d, *J* = 245.6 Hz), 129.28 (d, *J* = 4.7 Hz), 129.08 (d, *J* = 8.0 Hz), 128.51 (d, *J* = 14.5 Hz), 124.47 (d, *J* = 3.6 Hz), 115.34 (d, *J* = 21.3 Hz), 68.4, -0.0.

^{19}F NMR: (377 MHz, $CDCl_3$) δ -122.9

^{29}Si NMR: (79 MHz, $CDCl_3$) δ 19.8.

EI-MS m/z (rel. int.): 183 ([M-15]$^+$, 80%), 167 (60), 109 (80), 77 (100).

((3-Iodobenzyl)oxy)trimethylsilane (**3d**)

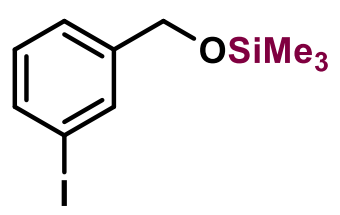

((3-Iodobenzyl)oxy)trimethylsilane was obtained in 90% yield as an oil (0.275 g). The title compound is a new product. (known synthesis but with no characterization).[7]

^{1}H NMR: (400 MHz, $CDCl_3$) δ 7.71 – 7.67 (m, 1H), 7.63 – 7.55 (m, 1H), 7.30 – 7.26 (m, 1H), 7.06 (t, J = 7.7 Hz, 1H), 4.64 (s, 2H), 0.17 (s, 9H).

^{13}C{^{1}H} NMR: (101 MHz, $CDCl_3$) δ 143.56, 136.23, 135.54, 130.15, 125.70, 94.47, 63.82, -0.29.

^{29}Si NMR: (79 MHz, $CDCl_3$) δ 19.8.

EI-MS m/z (rel. int.): 306 ($[M]^+$; 20%), 291 (95), 217 (100), 90 (85).

HRMS (ESI-TOF) m/z: $[M + Na]^+$ Calcd for $[C_{10}H_{15}INaOSi]^+$ 328.9829; Found 328.9823.

((3,5-Dichlorobenzyl)oxy)trimethylsilane (**3e**)

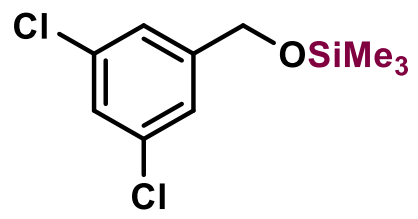

((3,5-Dichlorobenzyl)oxy)trimethylsilane was obtained in 98% yield as an oil (0.243 g). The title compound is a new product. (known synthesis but with no characterization).[8]

^{1}H NMR: (400 MHz, $CDCl_3$) δ 7.24 (t, J = 2.0 Hz, 1H), 7.23 – 7.16 (m, 2H), 4.63 (s, 2H), 0.17 (s, 9H).

^{13}C{^{1}H} NMR: (101 MHz, $CDCl_3$) δ 145.1, 135.3, 127.6, 125.1, 63.7, -0.1.

^{29}Si NMR: (79 MHz, $CDCl_3$) δ 20.4.

EI-MS m/z (rel. int.): 248 ($[M]^+$; 5%), 239 (100), 203 (70), 158 (80).

HRMS (ESI-TOF) m/z: $[M + Na]^+$ Calcd for $[C_{10}H_{14}Cl_2NaOSi]^+$ 271.0083; Found 271.0090.

Trimethyl((4-(trifluoromethyl)benzyl)oxy)silane (**3f**)

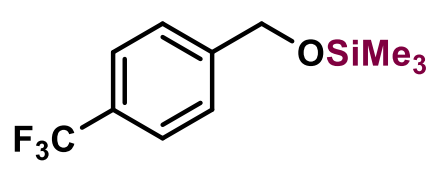

Trimethyl((4-(trifluoromethyl)benzyl)oxy)silane was obtained in 93% yield as an oil (0.231 g). The title compound was known in the literature, and all spectroscopic data are in agreement.[9]

^{1}H NMR: (400 MHz, $CDCl_3$) δ 7.59 (d, J = 8.1 Hz, 2H), 7.44 (d, J = 7.8 Hz, 2H), 4.75 (s, 2H), 0.18 (s, 9H).

^{13}C{^{1}H} NMR: (101 MHz, $CDCl_3$) δ 145.3, 129.45 (q, J = 32.3 Hz), 128.48, 126.54, 125.78, 125.37 (q, J = 3.8 Hz), 124.42 (q, J = 271.8 Hz), 120.4, 64.0, -0.3.

^{19}F NMR: (377 MHz, $CDCl_3$) δ -62.4.

^{29}Si NMR: (79 MHz, $CDCl_3$) δ 20.0.

EI-MS m/z (rel. int.): 233 ($[M]^+$; 100%), 203 (60), 159 (60), 109 (50).

4-(((Trimethylsilyl)oxy)methyl)benzonitrile (**3g**)

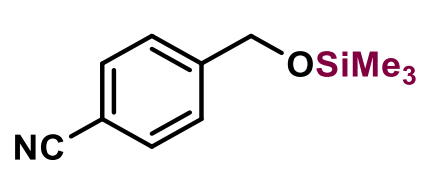

4-(((Trimethylsilyl)oxy)methyl)benzonitrile was obtained in 98% yield as a pale-yellow oil (0.201 g). The title compound was known in the literature, and all spectroscopic data are in agreement.[9]

^{1}H NMR: (400 MHz, $CDCl_3$) δ 7.61 (d, J = 8.3 Hz, 2H), 7.42 (d, J = 8.8 Hz, 2H), 4.74 (s, 2H), 0.17 (s, 9H).

^{13}C{^{1}H} NMR: (101 MHz, $CDCl_3$) δ 146.8, 132.2, 126.7, 119.1, 110.8, 63.8, -0.4.

^{29}Si NMR: (79 MHz, $CDCl_3$) δ 20.5.

EI-MS m/z (rel. int.): 190 ([M]$^+$; 100%), 160 (60), 116 (75), 89 (30).

(*S*)-(3-Chloro-1-phenylpropoxy)trimethylsilane (**3h**)

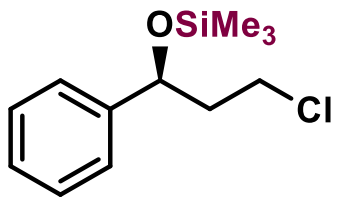

(*S*)-(3-Chloro-1-phenylpropoxy)trimethylsilane was obtained in 91% yield as an oil (0.220 g). The title compound was known in the literature, and all spectroscopic data are in agreement.

^{1}H NMR: (400 MHz, $CDCl_3$) δ 7.31 – 7.24 (m, 4H), 7.24 – 7.15 (m, 1H), 4.84 (dd, J = 8.7, 4.0 Hz, 1H), 3.75 – 3.56 (m, 1H), 3.56 – 3.42 (m, 1H), 2.13 – 2.01 (m, 1H), 2.02 – 1.89 (m, 1H), -0.00 (s, 9H).

^{13}C{^{1}H} NMR: (101 MHz, $CDCl_3$) δ 144.6, 128.4, 127.4, 125.9, 71.6, 43.4, 42.0, 0.2.

^{29}Si NMR: (79 MHz, $CDCl_3$) δ 18.5.

EI-MS m/z (rel. int.): 227 ([M-CH_3]$^+$; 1%), 179 (100), 93 (30), 73 (75)

((2,3-Dimethylbenzyl)oxy)trimethylsilane (**3i**)

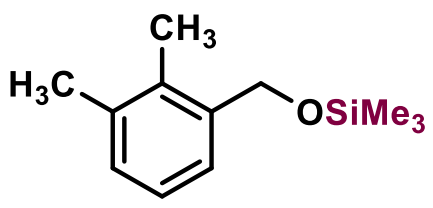

((2,3-Dimethylbenzyl)oxy)trimethylsilane was obtained in 94% yield as an oil (0.196 g). The title compound is a new product.

^{1}H NMR: (400 MHz, $CDCl_3$) δ 7.33 – 7.19 (m, 1H), 7.14 – 7.05 (m, 2H), 4.86 – 4.63 (m, 2H), 2.33 – 2.29 (m, 3H), 2.22 (d, J = 3.3 Hz, 3H), 0.19 (s, 9H).

^{13}C{^{1}H} NMR: (101 MHz, $CDCl_3$) δ 138.6, 136.9, 134.5, 129.9, 129.3, 125.5, 63.7, 20.4, 14.5, -0.3.

^{29}Si NMR: (79 MHz, $CDCl_3$) δ 19.0.

EI-MS m/z (rel. int.): 208 (M+; 5%), 118 (100), 91 (30), 73 (30).

HRMS (ESI-TOF) m/z: [M + Na]$^+$ Calcd for [$C_{12}H_{20}NaOSi$]$^+$ 231.1176; Found 231.1177.

Trimethyl(naphthalen-2-ylmethoxy)silane (**3j**)

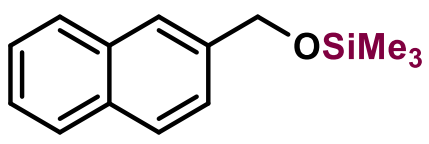

Trimethyl(naphthalen-2-ylmethoxy)silane was obtained in 99% yield as an oil (0.228 g). The title compound was known in the literature, and all spectroscopic data are in agreement.[9]

^{1}H NMR: (400 MHz, $CDCl_3$) δ 7.89 – 7.75 (m, 4H), 7.57 – 7.40 (m, 3H), 4.88 (s, 2H), 0.21 (s, 9H).

^{13}C{^{1}H} NMR: (101 MHz, $CDCl_3$) δ 138.6, 133.5, 132.9, 128.1, 128.0, 127.8, 126.1, 125.7, 125.1, 125.0, 65.0, -0.2.

^{29}Si NMR: (79 MHz, $CDCl_3$) δ 19.4.

EI-MS m/z (rel. int.): 230 ([M]+; 25%), 215 (25), 141 (100), 115 (30).

((4-Ethynylbenzyl)oxy)trimethylsilane (**3k**)

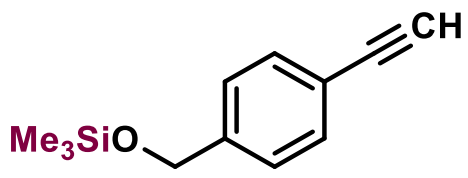

((4-Ethynylbenzyl)oxy)trimethylsilane was obtained in 80% yield as an oil (0.163 g). The title compound is a new product. (known use but with no characterization).[10]

^{1}H NMR: (400 MHz, $CDCl_3$) δ 7.47 (d, *J* = 8.2 Hz, 2H), 7.29 (dd, *J* = 7.9, 0.8 Hz, 2H), 4.70 (s, 2H), 3.05 (s, 1H), 0.16 (s, 9H).

^{13}C{^{1}H} NMR: (101 MHz, $CDCl_3$) δ 142.1, 132.2, 126.4, 120.9, 83.9, 77.0, 64.3, -0.3.

^{29}Si NMR: (79 MHz, $CDCl_3$) δ 19.6.

EI-MS m/z (rel. int.): 204 ([M]+; 5%), 189 (60), 159 (20), 115 (100).

HRMS (ESI-TOF) m/z: [M + Na]+ Calcd for $[C_{12}H_{16}NaOSi]^+$ 227.0863; Found 227.0869.

Trimethyl((3-phenylprop-2-yn-1-yl)oxy)silane (**3l**)

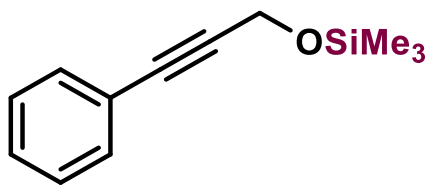

Trimethyl((3-phenylprop-2-yn-1-yl)oxy)silane was obtained in 95% yield as an oil (0.194 g). The title compound was known in the literature, and all spectroscopic data are in agreement.[9]

^{1}H NMR: (400 MHz, $CDCl_3$) δ 7.49 – 7.41 (m, 2H), 7.36 – 7.28 (m, 3H), 4.52 (s, 2H), 0.23 (s, 9H).

^{13}C{^{1}H} NMR: (101 MHz, $CDCl_3$) δ 131.8, 128.4, 128.4, 123.0, 87.7, 85.1, 51.8, -0.1.

^{29}Si NMR: (79 MHz, $CDCl_3$) δ 21.4.

EI-MS m/z (rel. int.): 204 ([M]+; 10%), 159 (100), 130 (45), 115 (80).

Trimethyl(oct-1-yn-3-yloxy)silane (**3m**)

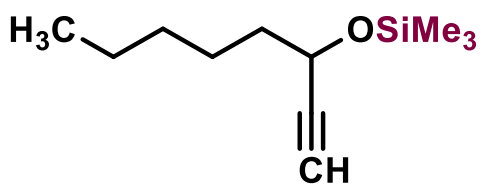

Trimethyl(oct-1-yn-3-yloxy)silane was obtained in 87% yield as an oil (0.172 g). The title compound was known in the literature, and all spectroscopic data are in agreement.[11]

^{1}H NMR: (400 MHz, $CDCl_3$) δ 4.32 (td, *J* = 6.6, 2.1 Hz, 1H), 2.38 (d, *J* = 2.1 Hz, 1H), 1.72 – 1.59 (m, 2H), 1.44 – 1.37 (m, 2H), 1.36 – 1.24 (m, 4H), 0.89 (t, *J* = 6.9 Hz, 3H), 0.17 (s, 9H).

^{13}C{^{1}H} NMR: (101 MHz, $CDCl_3$) δ 85.7, 72.2, 62.7, 38.6, 31.6, 25.0, 22.7, 14.1, 0.2.

^{29}Si NMR: (79 MHz, $CDCl_3$) δ 19.5.

EI-MS m/z (rel. int.): 183 ([M-CH_3]+; 5%), 127 (100), 113 (25), 73 (70).

(Dec-9-en-1-yloxy)trimethylsilane (**3n**)

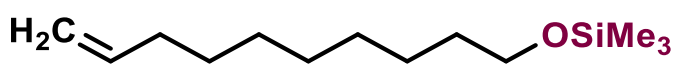

(Dec-9-en-1-yloxy)trimethylsilane was obtained in 97% yield as an oil (0.221 g). The title compound was known in the literature, and all spectroscopic data are in agreement.[5]

^{1}H NMR: (400 MHz, $CDCl_3$) δ 5.88 – 5.73 (m, 1H), 5.06 – 4.82 (m, 2H), 3.56 (t, *J* = 6.7 Hz, 2H), 2.22 – 1.89 (m, 2H), 1.51 (d, *J* = 6.8 Hz, 2H), 1.45 – 1.35 (m, 2H), 1.29 (s, 8H), 0.11 (s, 9H).

^{13}C{^{1}H} NMR: (101 MHz, $CDCl_3$) δ 139.4, 114.3, 62.9, 34.0, 32.9, 29.6, 29.5, 29.2, 29.1, 26.0, -0.3.

^{29}Si NMR: (79 MHz, $CDCl_3$) δ 17.0.

EI-MS m/z (rel. int.): 213 ($[M-CH_3]^+$; 15%), 103 (15), 75 (100), 55 (15).

Trimethyl(undec-10-en-1-yloxy)silane (**3o**)

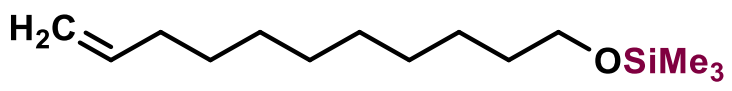

Trimethyl(undec-10-en-1-yloxy)silane was obtained in 92% yield as an oil (0.223 g). The title compound was known in the literature, and all spectroscopic data are in agreement.[12]

^{1}H NMR: (400 MHz, $CDCl_3$) δ 6.00 – 5.59 (m, 1H), 5.23 – 4.85 (m, 2H), 3.56 (t, *J* = 6.7 Hz, 2H), 2.14 – 1.91 (m, 2H), 1.63 – 1.49 (m, 2H), 1.46 – 1.33 (m, 2H), 1.28 (s, 10H), 0.11 (s, 9H).

^{13}C{^{1}H} NMR: (101 MHz, $CDCl_3$) δ 139.4, 114.2, 62.9, 34.0, 32.9, 29.7, 29.6, 29.3, 29.1, 26.0, -0.3.

^{29}Si NMR: (79 MHz, $CDCl_3$) δ 16.9.

EI-MS m/z (rel. int.): 227 ($[M-CH_3]^+$; 10%), 103 (10), 75 (100), 55 (20).

(Cinnamyloxy)trimethylsilane (**3p**)

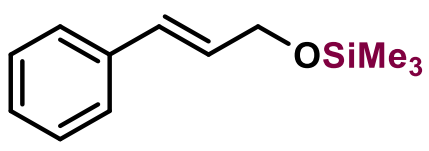

(Cinnamyloxy)trimethylsilane was obtained in 87% yield as an oil (0.179 g). The title compound was known in the literature, and all spectroscopic data are in agreement.[9]

^{1}H NMR: (400 MHz, $CDCl_3$) δ 7.42 – 7.37 (m, 2H), 7.35 – 7.29 (m, 2H), 7.26 – 7.19 (m, 1H), 6.59 (dt, *J* = 15.8, 1.8 Hz, 1H), 6.30 (dt, *J* = 15.8, 5.4 Hz, 1H), 4.33 (dd, *J* = 5.4, 1.7 Hz, 2H), 0.18 (s, 9H).

^{13}C{^{1}H} NMR: (101 MHz, $CDCl_3$) δ 137.1, 130.3, 128.9, 128.6, 127.5, 126.6, 63.6, -0.2.

^{29}Si NMR: (79 MHz, $CDCl_3$) δ 19.1.

EI-MS m/z (rel. int.): 206 ($[M]^+$; 55%), 191 (50), 117 (100), 73 (50).

(*E*)-((3,7-dimethylocta-2,6-dien-1-yl)oxy)trimethylsilane (**3q**)

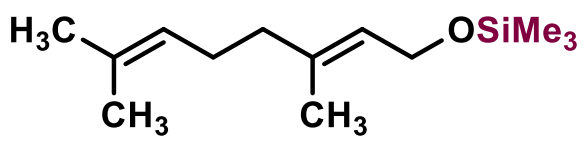

(*E*)-((3,7-dimethylocta-2,6-dien-1-yl)oxy)trimethylsilane was obtained in 93% yield as an oil (0.210 g). The title compound was known in the literature, and all spectroscopic data are in agreement.[13]

^{1}H NMR: (400 MHz, $CDCl_3$) δ 5.39 – 5.23 (m, 1H), 5.13 – 5.01 (m, 1H), 4.16 (d, *J* = 6.6 Hz, 2H), 2.14 – 2.05 (m, 2H), 2.04 – 1.96 (m, 2H), 1.68 (d, *J* = 1.4 Hz, 3H), 1.64 (d, *J* = 1.3 Hz, 3H), 1.60 (d, *J* = 1.3 Hz, 3H), 0.13 (s, 9H).

^{13}C{^{1}H} NMR: (101 MHz, $CDCl_3$) δ 137.8, 131.7, 124.2, 124.0, 59.7, 39.7, 26.5, 25.8, 17.8, 16.4, -0.1.

^{29}Si NMR: (79 MHz, $CDCl_3$) δ 17.9.

EI-MS m/z (rel. int.): 134 (1%), 119 (30), 93 (70), 75 (100).

(Cyclohexylmethoxy)trimethylsilane (**3r**)

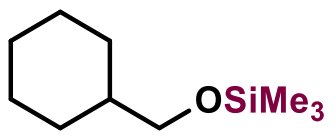

(Cyclohexylmethoxy)trimethylsilane was obtained in 91% yield as an oil (0.169 g). The title compound was known in the literature, and all spectroscopic data are in agreement.[14]

^{1}H NMR: (400 MHz, $CDCl_3$) δ 3.35 (d, *J* = 6.6 Hz, 2H), 1.85 – 1.61 (m, 5H), 1.52 – 1.39 (m, 1H), 1.30 – 1.11 (m, 3H), 0.93 – 0.80 (m, 2H), 0.10 (s, 9H).

^{13}C{^{1}H} NMR: (101 MHz, $CDCl_3$) δ 68.6, 40.4, 29.9, 26.9, 26.1, -0.3.

^{29}Si NMR: (79 MHz, $CDCl_3$) δ 17.0.

EI-MS m/z (rel. int.): 171 ($[M-CH_3]^+$; 95%), 103 (25), 75 (100), 55 (20).

(2-Cyclohexylethoxy)trimethylsilane (**3s**)

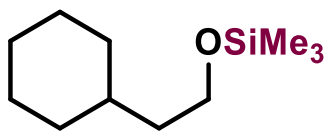

(2-Cyclohexylethoxy)trimethylsilane was obtained in 89% yield as an oil (0.178 g). The title compound is a new product. (known synthesis but with no characterization).[15]

^{1}H NMR: (400 MHz, $CDCl_3$) δ 3.60 (t, *J* = 6.9 Hz, 2H), 1.74 – 1.63 (m, 4H), 1.50 – 1.37 (m, 2H), 1.29 – 1.08 (m, 4H), 1.02 – 0.82 (m, 2H), 0.10 (s, 9H).

^{13}C{^{1}H} NMR: (101 MHz, $CDCl_3$) δ 60.7, 40.5, 34.4, 33.5, 26.8, 26.5, -0.3.

^{29}Si NMR: (79 MHz, $CDCl_3$) δ 16.9.

EI-MS m/z (rel. int.): 185 ($[M-CH_3]^+$; 40%), 109 (100), 67 (40), 55 (20).

HRMS (ESI-TOF) m/z: $[M + Na]^+$ Calcd for $[C_{11}H_{24}NaOSi]^+$ 223.1489; Found 223.1489.

((9-Bromononyl)oxy)trimethylsilane (**3t**)

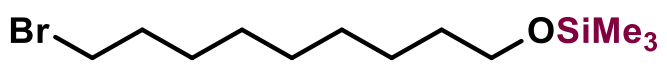

((9-Bromononyl)oxy)trimethylsilane was obtained in 85% yield as an oil (0.250 g). The title compound was known in the literature, and all spectroscopic data are in agreement.[16]

^{1}H NMR: (400 MHz, $CDCl_3$) δ 3.56 (t, *J* = 6.7 Hz, 2H), 3.39 (t, *J* = 6.9 Hz, 2H), 1.84 (dt, *J* = 14.5, 7.0 Hz, 2H), 1.59 – 1.48 (m, 2H), 1.47 – 1.37 (m, 2H), 1.29 (s, 8H), 0.10 (s, 9H).

^{13}C{^{1}H} NMR: (101 MHz, $CDCl_3$) δ 62.8, 34.1, 33.0, 32.8, 29.5, 29.5, 28.8, 28.3, 25.9, -0.3.

^{29}Si NMR: (79 MHz, $CDCl_3$) δ 17.0.

EI-MS m/z (rel. int.): 169 (10%), 83 (50), 69 (100), 55 (50).

2,2,10,10-Tetramethyl-3,9-dioxa-2,10-disilaundecane (**3u**)

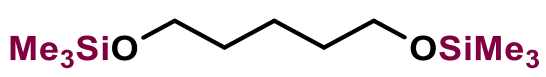

2,2,10,10-Tetramethyl-3,9-dioxa-2,10-disilaundecane was obtained in 88% yield as an oil (0.218 g). The title compound was known in the literature, and all spectroscopic data are in agreement.[17]

^{1}H NMR: (400 MHz, $CDCl_3$) δ 3.57 (t, *J* = 6.7 Hz, 4H), 1.71 – 1.47 (m, 4H), 1.40 – 1.28 (m, 2H), 0.10 (s, 9H).

$^{13}C\{^1H\}$ NMR: (101 MHz, $CDCl_3$) δ 62.7, 32.6, 22.3, -0.3.

^{29}Si NMR: (79 MHz, $CDCl_3$) δ 17.1.

EI-MS m/z (rel. int.): 177 (5%), 147 (100), 143 (30), 69 (70).

(Benzo[d][1,3]dioxol-5-ylmethoxy)trimethylsilane (**3v**)

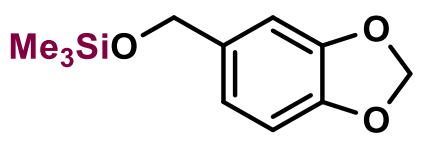

(Benzo[d][1,3]dioxol-5-ylmethoxy)trimethylsilane was obtained in 92% yield as an oil (0.206 g). The title compound was known in the literature, and all spectroscopic data are in agreement.[18]

^1H NMR: (400 MHz, $CDCl_3$) δ 6.84 (s, 1H), 6.78 – 6.75 (m, 2H), 5.94 (s, 2H), 4.59 (s, 2H), 0.16 (s, 9H).

$^{13}C\{^1H\}$ NMR: (101 MHz, $CDCl_3$) δ 147.8, 146.8, 135.1, 120.0, 108.1, 107.7, 101.0, 64.7, -0.2.

^{29}Si NMR: (79 MHz, $CDCl_3$) δ 19.0.

EI-MS m/z (rel. int.): 224 ($[M]^+$; 25%), 209 (10), 135 (100), 77 (10).

6-(((Trimethylsilyl)oxy)methyl)benzo[d]thiazole (**3w**)

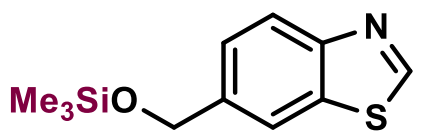

6-(((Trimethylsilyl)oxy)methyl)benzo[d]thiazole was obtained in 99% yield as an oil (0.235 g). The title compound is a new product.

^1H NMR: (400 MHz, $CDCl_3$) δ 8.95 (d, *J* = 1.1 Hz, 1H), 8.08 (d, *J* = 8.4 Hz, 1H), 7.98 – 7.93 (m, 1H), 7.57 – 7.41 (m, 1H), 4.84 (d, *J* = 1.0 Hz, 2H), 0.19 (s, 9H).

$^{13}C\{^1H\}$ NMR: (101 MHz, $CDCl_3$) δ 153.8, 152.6, 139.0, 134.0, 125.1, 123.4, 119.5, 64.4, -0.3.

^{29}Si NMR: (79 MHz, $CDCl_3$) δ 19.7.

EI-MS m/z (rel. int.): 237 ($[M]^+$; 15%), 222 (60), 192 (15), 148 (100).

HRMS (ESI-TOF) m/z: $[M + H]^+$ Calcd for $[C_{11}H_{16}NOSSi]^+$ 238.0716; Found 238.0714.

((9H-Fluoren-9-yl)oxy)trimethylsilane (**3x**)

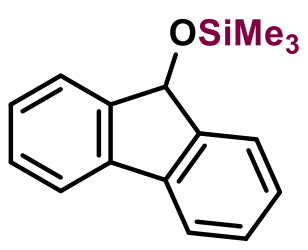

((9H-Fluoren-9-yl)oxy)trimethylsilane was obtained in 99% yield as an oil (0.251 g). The title compound was known in the literature, and all spectroscopic data are in agreement.[9]

^1H NMR: (400 MHz, $CDCl_3$) δ 7.71 – 7.61 (m, 2H), 7.58 – 7.49 (m, 2H), 7.43 – 7.35 (m, 2H), 7.33 – 7.28 (m, 2H), 5.70 (s, 1H), 0.21 (s, 9H).

$^{13}C\{^1H\}$ NMR: (101 MHz, $CDCl_3$) δ 146.0, 140.1, 128.8, 127.7, 125.2, 120.0, 75.6, 1.1.

^{29}Si NMR: (79 MHz, $CDCl_3$) δ 19.5.

EI-MS m/z (rel. int.): 254 ($[M]^+$; 30%), 239 (5), 165 (100), 73 (25).

(1-(4-Fluorophenyl)ethoxy)trimethylsilane (**3y**)

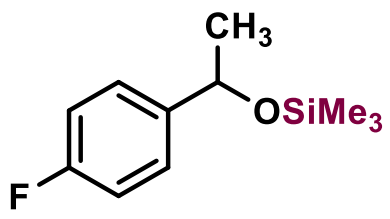

(1-(4-Fluorophenyl)ethoxy)trimethylsilane was obtained in 96% yield as an oil (0.204 g). The title compound was known in the literature, and all spectroscopic data are in agreement.[19]

^{1}H NMR: (400 MHz, $CDCl_3$) δ 7.36 – 7.11 (m, 1H), 7.01 – 6.88 (m, 2H), 6.86 – 6.72 (m, 1H), 4.75 (dd, *J* = 6.4, 2.1 Hz, 1H), 1.32 (dd, *J* = 6.4, 2.2 Hz, 3H), -0.00 (s, 9H).

^{13}C{^{1}H} NMR: (101 MHz, $CDCl_3$) δ 162.75 (d, *J* = 245.1 Hz), 149.20 (d, *J* = 6.8 Hz), 129.42 (d, *J* = 8.2 Hz), 120.68 (d, *J* = 2.7 Hz), 113.43 (d, *J* = 21.3 Hz), 112.09 (d, *J* = 21.9 Hz), 69.8, 26.6, -0.1.

^{19}F NMR: (377 MHz, $CDCl_3$) δ -113.5.

^{29}Si NMR: (79 MHz, $CDCl_3$) δ 17.2.

EI-MS m/z (rel. int.): 197 ($[M-CH_3]^+$; 70%), 122 (25), 103 (25), 75 (100).

(1-(4-Chlorophenyl)ethoxy)trimethylsilane (**3z**)

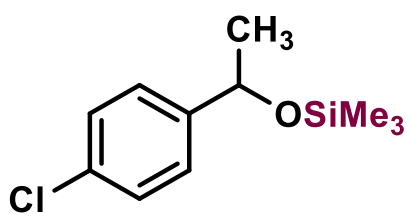

(1-(4-Chlorophenyl)ethoxy)trimethylsilane was obtained in 98% yield as an oil (0.223 g). The title compound was known in the literature, and all spectroscopic data are in agreement.[20]

^{1}H NMR: (400 MHz, $CDCl_3$) δ 7.51 – 7.11 (m, 4H), 4.83 (q, *J* = 6.4 Hz, 1H), 1.40 (d, *J* = 6.4 Hz, 3H), 0.08 (s, 9H).

^{13}C{^{1}H} NMR: (101 MHz, $CDCl_3$) δ 145.2, 132.6, 128.4, 126.9, 70.1, 27.0, 0.2.

^{29}Si NMR: (79 MHz, $CDCl_3$) δ 17.1.

EI-MS m/z (rel. int.): 213 ($[M-CH_3]^+$; 70%), 138 (20), 103 (40), 75 (100).

(1-(4-Bromophenyl)ethoxy)trimethylsilane (**3aa**)

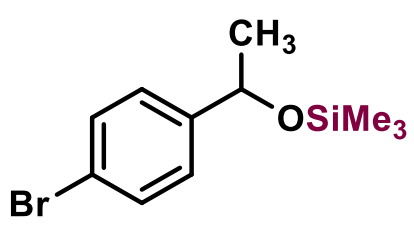

(1-(4-Bromophenyl)ethoxy)trimethylsilane was obtained in 99% yield as an oil (0.269 g). The title compound was known in the literature, and all spectroscopic data are in agreement.[19]

^{1}H NMR: (400 MHz, $CDCl_3$) δ 7.47 – 7.41 (m, 2H), 7.24 – 7.18 (m, 2H), 4.81 (q, *J* = 6.4 Hz, 1H), 1.40 (d, *J* = 6.4 Hz, 3H), 0.08 (s, 9H).

^{13}C{^{1}H} NMR: (101 MHz, $CDCl_3$) δ 145.4, 131.1, 126.9, 120.3, 69.8, 26.7, -0.1.

^{29}Si NMR: (79 MHz, $CDCl_3$) δ 17.2.

EI-MS m/z (rel. int.): 257 ($[M-CH_3]^+$; 30%), 181 (20), 103 (30), 75 (100).

1-Trimethylsiloxyadamantane (**3ab**)

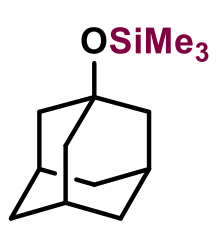

1-Trimethylsiloxyadamantane was obtained in 93% yield as an oil (0.208 g). The title compound was known in the literature, and all spectroscopic data are in agreement.[9]

^{1}H NMR: (400 MHz, $CDCl_3$) δ 2.13 – 2.07 (m, 3H), 1.76 (d, J = 3.5 Hz, 6H), 1.64 – 1.55 (m, 6H), 0.12 (s, 9H).

^{13}C{^{1}H} NMR: (101 MHz, $CDCl_3$) δ 71.6, 46.2, 36.4, 31.1, 3.1.

^{29}Si NMR: (79 MHz, $CDCl_3$) δ 7.7.

EI-MS m/z (rel. int.): 224 ([M]$^+$; 20%), 209 (90), 167 (85), 135 (100).

Bis(trimethylsilyl) 4-methylolphenol (**3ac**)

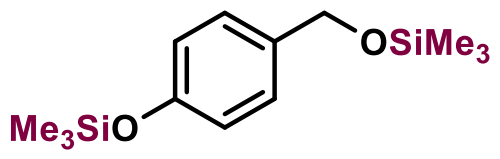

Bis(trimethylsilyl) 4-methylolphenol was obtained in 90% yield as an oil (0.241 g). The title compound was known in the literature, and all spectroscopic data are in agreement.[21]

^{1}H NMR: (400 MHz, $CDCl_3$) δ 7.23 – 7.17 (m, 2H), 6.81 (d, J = 8.5 Hz, 2H), 4.63 (s, 2H), 0.26 (s, 9H), 0.15 (s, 9H).

^{13}C{^{1}H} NMR: (101 MHz, $CDCl_3$) δ 154.5, 134.0, 128.2, 120.1, 64.6, 0.3, -0.2.

^{29}Si NMR: (79 MHz, $CDCl_3$) δ 19.5, 18.7.

EI-MS m/z (rel. int.): 268 ([M]$^+$; 35%), 253 (30), 179 (100), 73 (65).

(2,6-Dimethylphenoxy)trimethylsilane (**3ad**)

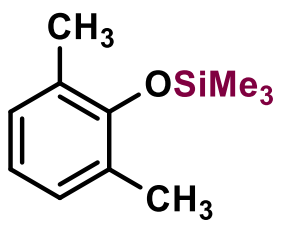

(2,6-Dimethylphenoxy)trimethylsilane was obtained in 86% yield as a pale-yellow oil (0.167 g). The title compound was known in the literature, and all spectroscopic data are in agreement.[5]

^{1}H NMR: (400 MHz, $CDCl_3$) δ 7.03 – 6.92 (m, 2H), 6.80 (t, J = 7.4 Hz, 1H), 2.21 (d, J = 1.0 Hz, 6H), 0.26 (d, J = 1.0 Hz, 9H).

^{13}C{^{1}H} NMR: (101 MHz, $CDCl_3$) δ 128.7, 128.6, 125.4, 121.4, 31.7, 17.8, 1.1.

^{29}Si NMR: (79 MHz, $CDCl_3$) δ 18.4.

EI-MS m/z (rel. int.): 194 ([M]$^+$; 75%), 179 (80), 105 (100), 73 (50).

4-Chloro-3,5-dimethylphenoxytrimethylsilane (**3ae**)

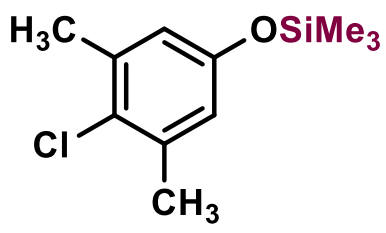

4-Chloro-3,5-dimethylphenoxytrimethylsilane was obtained in 88% yield as a pale-yellow oil (0.201 g). The title compound is a new product. (known synthesis but with no NMR characterization).

^{1}H NMR: (400 MHz, $CDCl_3$) δ 6.71 – 6.41 (m, 2H), 2.33 (d, J = 0.9 Hz, 6H), 0.26 (d, J = 1.2 Hz, 9H).

^{13}C{^{1}H} NMR: (101 MHz, $CDCl_3$) δ 153.1, 137.2, 127.2, 120.1, 20.9, 0.3.

^{29}Si NMR: (79 MHz, $CDCl_3$) δ 19.7.

EI-MS m/z (rel. int.): 228 ([M]$^+$; 60%), 213 (100), 93 (30), 73 (45).

HRMS (ESI-TOF) m/z: $[M + H]^+$ Calcd for $[C_{11}H_{18}ClOSi]^+$ 229.0810; Found 229.0809.

(4-Bromophenoxy)trimethylsilane (**3af**)

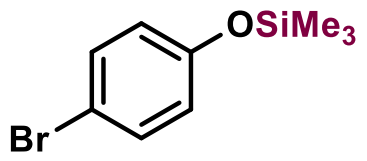

(4-Bromophenoxy)trimethylsilane was obtained in 99% yield as a pale-yellow oil (0.241 g). The title compound was known in the literature, and all spectroscopic data are in agreement.[5]

^{1}H NMR: (400 MHz, $CDCl_3$) δ 7.34 (d, *J* = 8.8 Hz, 2H), 6.73 (d, *J* = 8.8 Hz, 2H), 0.27 (s, 9H).

^{13}C{^{1}H} NMR: (101 MHz, $CDCl_3$) δ 154.5, 132.5, 122.0, 114.0, 0.3.

^{29}Si NMR: (79 MHz, $CDCl_3$) δ 20.5.

EI-MS m/z (rel. int.): 243 ($[M]^+$; 70%), 229 (100), 150 (65), 73 (80).

((3-Fluorobenzyl)oxy)diisopropylsilane (**3ag**)

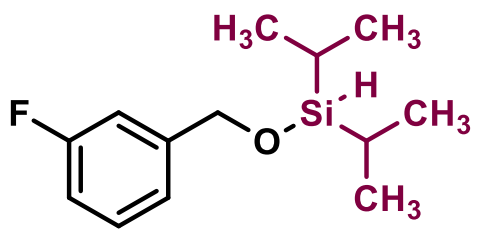

((3-Fluorobenzyl)oxy)diisopropylsilane was obtained in 80% yield as an oil (0.192 g). The title compound is a new product.

^{1}H NMR: (400 MHz, $CDCl_3$) δ 7.29 (d, *J* = 5.8 Hz, 1H), 7.12 – 7.03 (m, 2H), 6.94 (td, *J* = 8.5, 2.7 Hz, 1H), 4.80 (s, 2H), 4.25 (s, 1H), 1.07 (d, *J* = 5.3 Hz, 14H).

^{13}C{^{1}H} NMR: (101 MHz, $CDCl_3$) δ 162.83 (d, *J* = 245.3 Hz), 143.47 (d, *J* = 6.9 Hz), 129.52 (d, *J* = 8.1 Hz), 121.37 (d, *J* = 2.9 Hz), 113.67 (d, *J* = 21.3 Hz), 112.91 (d, *J* = 22.1 Hz), 17.3, 17.1, 12.3.

^{19}F NMR: (377 MHz, $CDCl_3$) δ -113.5.

^{29}Si NMR: (79 MHz, $CDCl_3$) δ 17.1.

EI-MS m/z (rel. int.): 197 ($[M\text{-}iPr]^+$; 35%), 139 (20), 109 (100), 91 (50).

HRMS (ESI-TOF) m/z: $[M + Na]^+$ Calcd for $[C_{13}H_{21}FNaOSi]^+$ 263.1238; Found 263.1233.

tert-Butyldimethyl(undec-10-en-1-yloxy)silane (**3ah**)

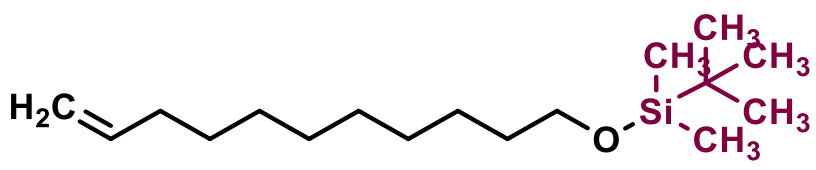

tert-Butyldimethyl(undec-10-en-1-yloxy)silane was obtained in 77% yield as an oil (0.219 g). The title compound was known in the literature, and all spectroscopic data are in agreement.[22]

^{1}H NMR: (400 MHz, $CDCl_3$) δ 6.03 – 5.74 (m, 1H), 5.12 – 4.88 (m, 2H), 3.60 (s, 2H), 2.12 – 1.97 (m, 2H), 1.51 (t, *J* = 6.7 Hz, 2H), 1.38 (t, *J* = 6.9 Hz, 4H), 1.28 (s, 10H), 0.90 (s, 9H), 0.05 (s, 6H).

^{13}C{^{1}H} NMR: (101 MHz, $CDCl_3$) δ 139.1, 113.9, 63.2, 33.7, 32.7, 29.4, 29.3, 29.3, 29.0, 28.8, 25.8, 25.6, 18.2, -5.4.

^{29}Si NMR: (79 MHz, $CDCl_3$) δ 18.3.

EI-MS m/z (rel. int.): 227 ($[M\text{-}tBu]^+$; 5%), 101 (5), 75 (100), 55 (15).

1,1,1-Trimethyl-3,3,3-tripropyldisiloxane (**4a**)

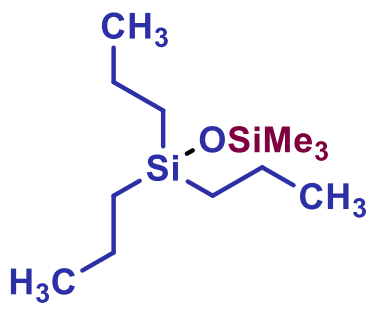

1,1,1-Trimethyl-3,3,3-tripropyldisiloxane was obtained in 97% yield as an oil (0.239 g). The title compound was known in the literature, and all spectroscopic data are in agreement.[20]

^{1}H NMR: (400 MHz, $CDCl_3$) δ 1.45 – 1.28 (m, 6H), 0.95 (t, *J* = 7.3 Hz, 9H), 0.58 – 0.46 (m, 6H), 0.06 (s, 9H).

^{13}C{^{1}H} NMR: (101 MHz, $CDCl_3$) δ 18.6, 18.5, 17.0, 2.1.

^{29}Si NMR: (79 MHz, $CDCl_3$) δ 6.3, 6.1.

EI-MS m/z (rel. int.): 203 ($[M-Pr]^+$; 30%), 161 (65), 119 (100), 73 (25).

1,1,1-Triisobutyl-3,3,3-trimethyldisiloxane (**4b**)

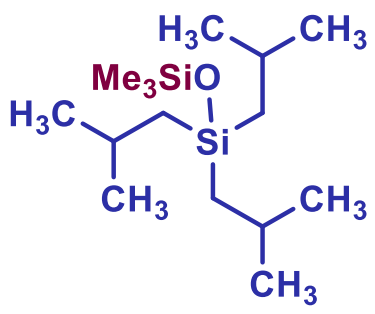

1,1,1-Triisobutyl-3,3,3-trimethyldisiloxanewas obtained in 90% yield as an oil (0.259 g). The title compound was known in the literature, and all spectroscopic data are in agreement.[5]

^{1}H NMR: (400 MHz, $CDCl_3$) δ 1.80 (dt, *J* = 13.3, 6.7 Hz, 3H), 0.94 (d, *J* = 6.5 Hz, 18H), 0.54 (d, *J* = 6.9 Hz, 6H), 0.07 (s, 9H).

^{13}C{^{1}H} NMR: (101 MHz, $CDCl_3$) δ 27.5, 26.3, 24.2, 2.0.

^{29}Si NMR: (79 MHz, $CDCl_3$) δ 5.6, 4.8.

EI-MS m/z (rel. int.): 231 ($[M-iBu]^+$; 40%), 175 (85), 119 (100), 73 (15).

1,1,1-Triisopropyl-3,3,3-trimethyldisiloxane (**4c**)

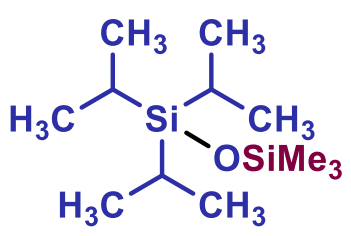

1,1,1-Triisopropyl-3,3,3-trimethyldisiloxane obtained in 91% yield as an oil (0.224 g). The title compound was known in the literature, and all spectroscopic data are in agreement.[20]

^{1}H NMR: (400 MHz, $CDCl_3$) δ 1.05 – 0.93 (m, 21H), 0.10 (s, 9H).

^{13}C{^{1}H} NMR: (101 MHz, $CDCl_3$) δ 18.0, 12.8, 2.2.

^{29}Si NMR: (79 MHz, $CDCl_3$) δ 6.5, 5.1.

EI-MS m/z (rel. int.): 231 ($[M-CH_3]^+$; 5%), 203 (100), 161 (45), 133 (90).

Tri-*tert*-butyl (trimethylsilyl) silicate (**4d**)

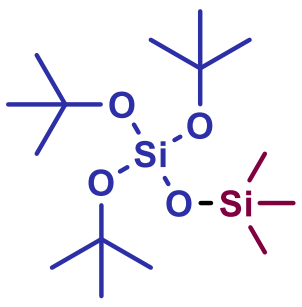

Tri-*tert*-butyl (trimethylsilyl) silicate obtained in 99% yield as an oil (0.333 g). The title compound was known in the literature, and all spectroscopic data are in agreement.[20]

^{1}H NMR: (400 MHz, $CDCl_3$) δ 1.28 (s, 27H), 0.12 (s, 9H).

^{13}C{^{1}H} NMR: (101 MHz, $CDCl_3$) δ 72.6, 31.8, 2.1.

^{29}Si NMR: (79 MHz, $CDCl_3$) δ 8.0, -99.5.

EI-MS m/z (rel. int.): 321 ($[M-CH_3]^+$; 40%), 209 (80), 153 (95), 57 (100).

Triisobutyl((4,4,5,5-tetramethyl-1,3,2-dioxaborolan-2-yl)oxy)silane (**5a**)

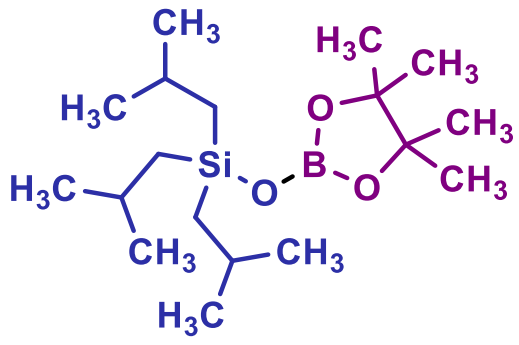

Triisobutyl((4,4,5,5-tetramethyl-1,3,2-dioxaborolan-2-yl)oxy)silane obtained in 90% yield as an oil (0.308 g). The title compound was known in the literature, and all spectroscopic data are in agreement.[23]

^{1}H NMR: (400 MHz, $CDCl_3$) δ 1.85 (dt, *J* = 13.3, 6.7 Hz, 3H), 1.22 (s, 12H), 0.95 (d, *J* = 6.6 Hz, 18H), 0.65 (d, *J* = 6.9 Hz, 6H).

^{13}C{^{1}H} NMR: (101 MHz, $CDCl_3$) δ 82.1, 26.5, 26.5, 24.8, 24.3.

^{11}B NMR: (128 MHz, $CDCl_3$) δ 20.6.

^{29}Si NMR: (79 MHz, $CDCl_3$) δ 13.0.

EI-MS m/z (rel. int.): 285 ([M-iBu]$^+$; 10%), 203 (30), 147 (40), 83 (100).

tert-Butyldimethyl((4,4,5,5-tetramethyl-1,3,2-dioxaborolan-2-yl)oxy)silane (**5b**)

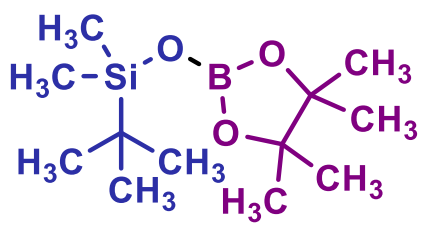

tert-Butyldimethyl((4,4,5,5-tetramethyl-1,3,2-dioxaborolan-2-yl)oxy)silane obtained in 99% yield as an oil (0.255 g). The title compound was known in the literature, and all spectroscopic data are in agreement.[24]

^{1}H NMR: (400 MHz, $CDCl_3$) δ 1.22 (s, 12H), 0.89 (s, 9H), 0.12 (s, 6H).

^{13}C{^{1}H} NMR: (101 MHz, $CDCl_3$) δ 82.2, 25.7, 24.7, 18.1, -3.7.

^{11}B NMR: (128 MHz, $CDCl_3$) δ 20.6.

^{29}Si NMR: (79 MHz, $CDCl_3$) δ 18.0.

EI-MS m/z (rel. int.): 243 ($[M-CH_3]^+$; 5%), 201 (30), 119 (30), 83 (100).

1-Methoxy-4-(4-methylpent-4-en-2-yl)benzene (**6a**)

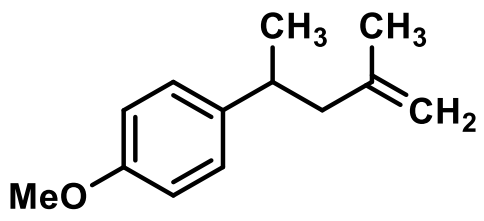

1-Methoxy-4-(4-methylpent-4-en-2-yl)benzene obtained in 95% yield as an oil (0.181 g). The title compound was known in the literature, and all spectroscopic data are in agreement.[25]

^{1}H NMR: (400 MHz, CDCl$_3$) δ 7.24 – 7.07 (m, 2H), 6.98 – 6.76 (m, 2H), 4.93 – 4.48 (m, 2H), 3.80 (s, 3H), 2.90 (q, *J* = 7.0 Hz, 1H), 2.48 – 2.05 (m, 2H), 1.71 (d, *J* = 1.1 Hz, 3H), 1.22 (dd, *J* = 7.0, 1.5 Hz, 3H).

^{13}C{^{1}H} NMR: (101 MHz, CDCl$_3$) δ 157.9, 144.3, 139.7, 127.9, 113.8, 112.1, 55.3, 47.2, 37.1, 22.4, 22.0.

EI-MS m/z (rel. int.): 190 ([M]$^+$; 5%), 135 (100), 105 (15), 91 (20).

(1-Cyclopropyl-3-methylbut-3-en-1-yl)benzene (**6b**)

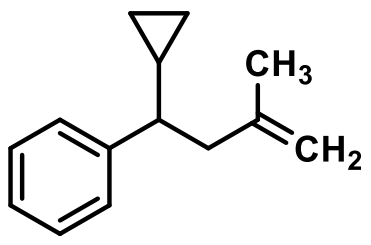

(1-Cyclopropyl-3-methylbut-3-en-1-yl)benzene obtained in 90% yield as an oil (0.167 g). The title compound was known in the literature, and all spectroscopic data are in agreement.[25]

^{1}H NMR: (400 MHz, CDCl$_3$) δ 7.40 – 7.26 (m, 2H), 7.24 – 7.11 (m, 2H), 4.90 – 4.47 (m, 2H), 2.62 – 2.41 (m, 2H), 2.05 (td, *J* = 9.0, 6.2 Hz, 1H), 1.65 (s, 3H), 1.07 – 0.95 (m, 1H), 0.64 – 0.53 (m, 1H), 0.42 – 0.33 (m, 1H), 0.28 – 0.18 (m, 1H), 0.16 – 0.05 (m, 1H).

^{13}C{^{1}H} NMR: (101 MHz, CDCl$_3$) δ 145.4, 144.0, 128.2, 127.7, 126.1, 112.2, 49.4, 45.0, 22.7, 18.1, 5.7, 3.8.

EI-MS m/z (rel. int.): 158 (1%), 131 (100), 115 (20), 91 (45).

Trimethyl(octylthio)silane (**7a**)

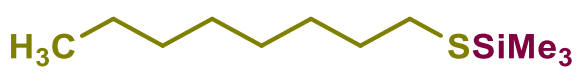

Trimethyl(octylthio)silane obtained in 80% yield as an oil (0.174 g). The title compound was known in the literature, and all spectroscopic data are in agreement.[26]

^{1}H NMR: (400 MHz, CDCl$_3$) δ 2.51 – 2.44 (m, 2H), 1.64 – 1.53 (m, 2H), 1.31 – 1.22 (m, 10H), 0.91 – 0.83 (m, 3H), 0.30 (s, 9H).

^{13}C{^{1}H} NMR: (101 MHz, CDCl$_3$) δ 33.5, 32.3, 29.7, 29.6, 29.3, 26.9, 23.1, 14.5, 1.4.

^{29}Si NMR: (79 MHz, CDCl$_3$) δ 15.7.

EI-MS m/z (rel. int.): 218 ([M]$^+$, 30%), 203 (100), 91 (90), 73 (90).

1,1,1-Triisopropyl-3,3,3-trimethyldisilathiane (**7b**)

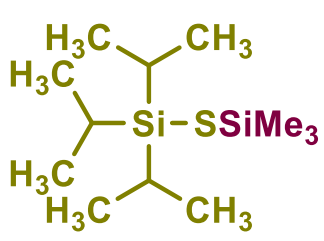

1,1,1-Triisopropyl-3,3,3-trimethyldisilathiane obtained in 77% yield as an oil (0.202 g). The title compound was known in the literature, and all spectroscopic data are in agreement.

^{1}H NMR: (400 MHz, CDCl$_3$) δ 1.34 – 0.95 (m, 21H), 0.37 (s, 9H).

^{13}C{^{1}H} NMR: (101 MHz, CDCl$_3$) δ 18.9, 14.7, 4.7.

^{29}Si NMR: (79 MHz, $CDCl_3$) δ 26.4, 14.6.

EI-MS m/z (rel. int.): 219 ($[M-iPr]^+$, 70%), 177 (65), 73 (100).

SPECTRA FOR ALL PRODUCTS

(Benzyloxy)trimethylsilane (**3a**)

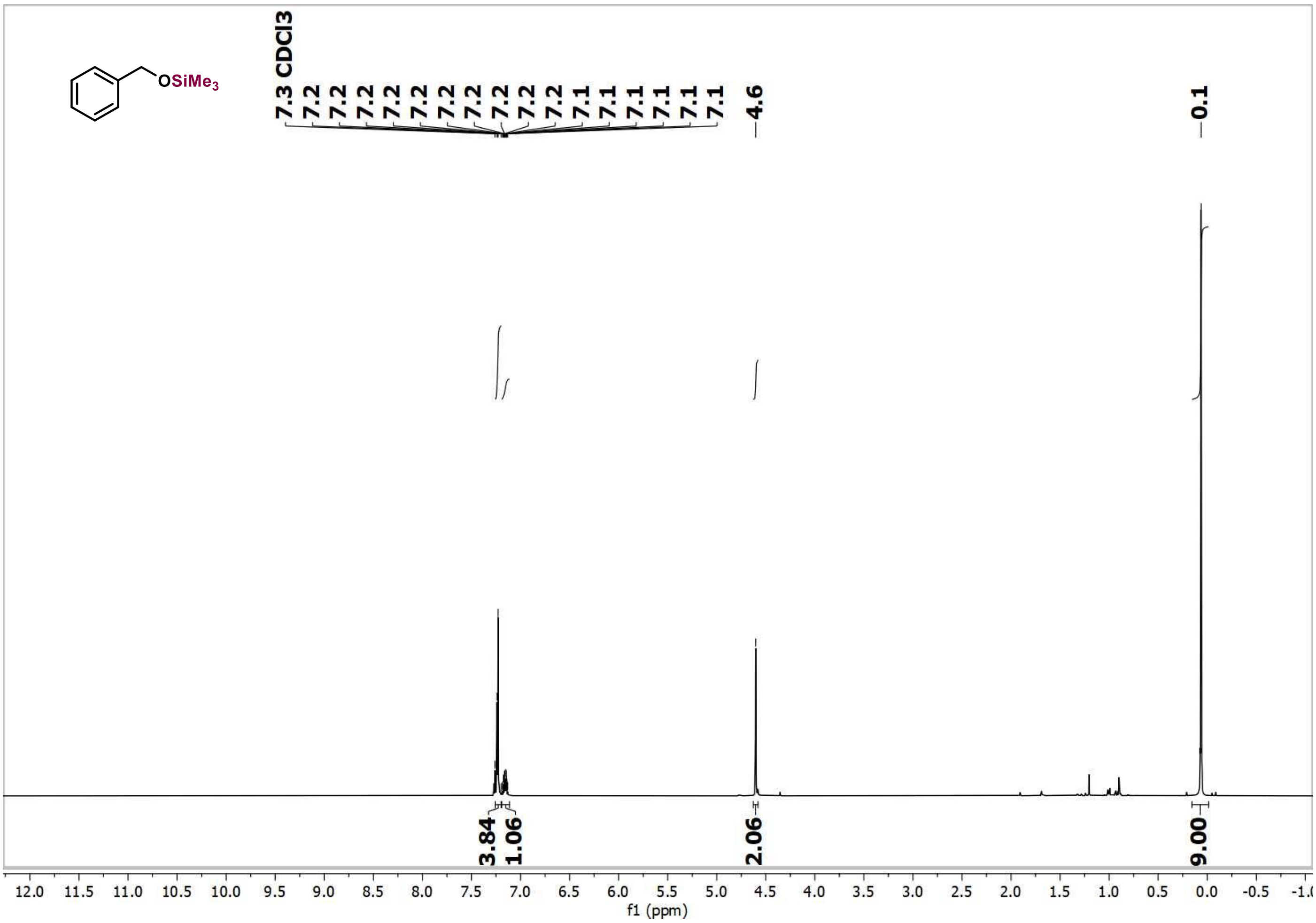

Figure S1. ^{1}H NMR (400 MHz, Chloroform-d, 25°C) of (benzyloxy)trimethylsilane (**3a**).

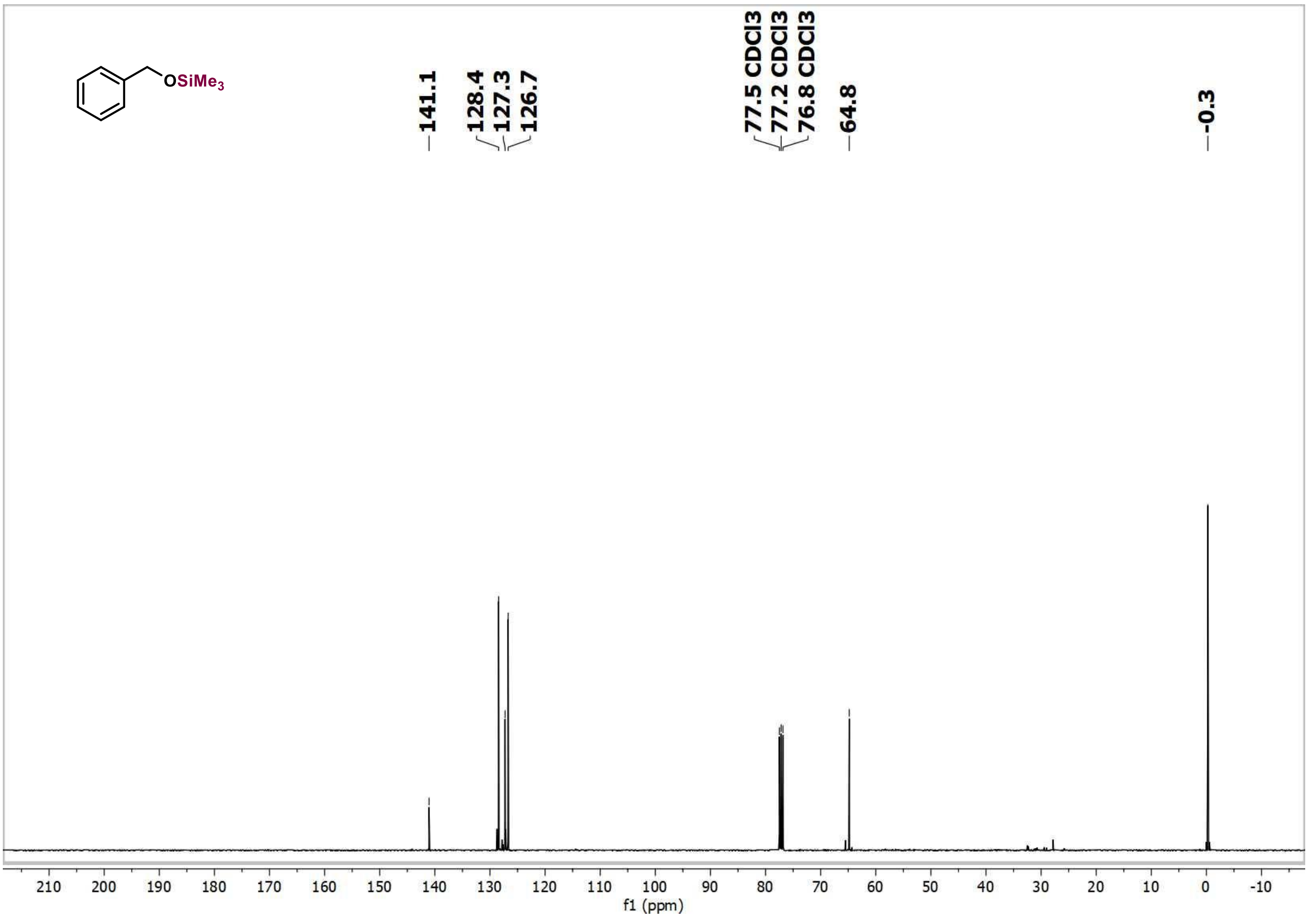

Figure S2. $^{13}C\{^{1}H\}$ NMR (101 MHz, Chloroform-d, 25°C) of (benzyloxy)trimethylsilane (**3a**).

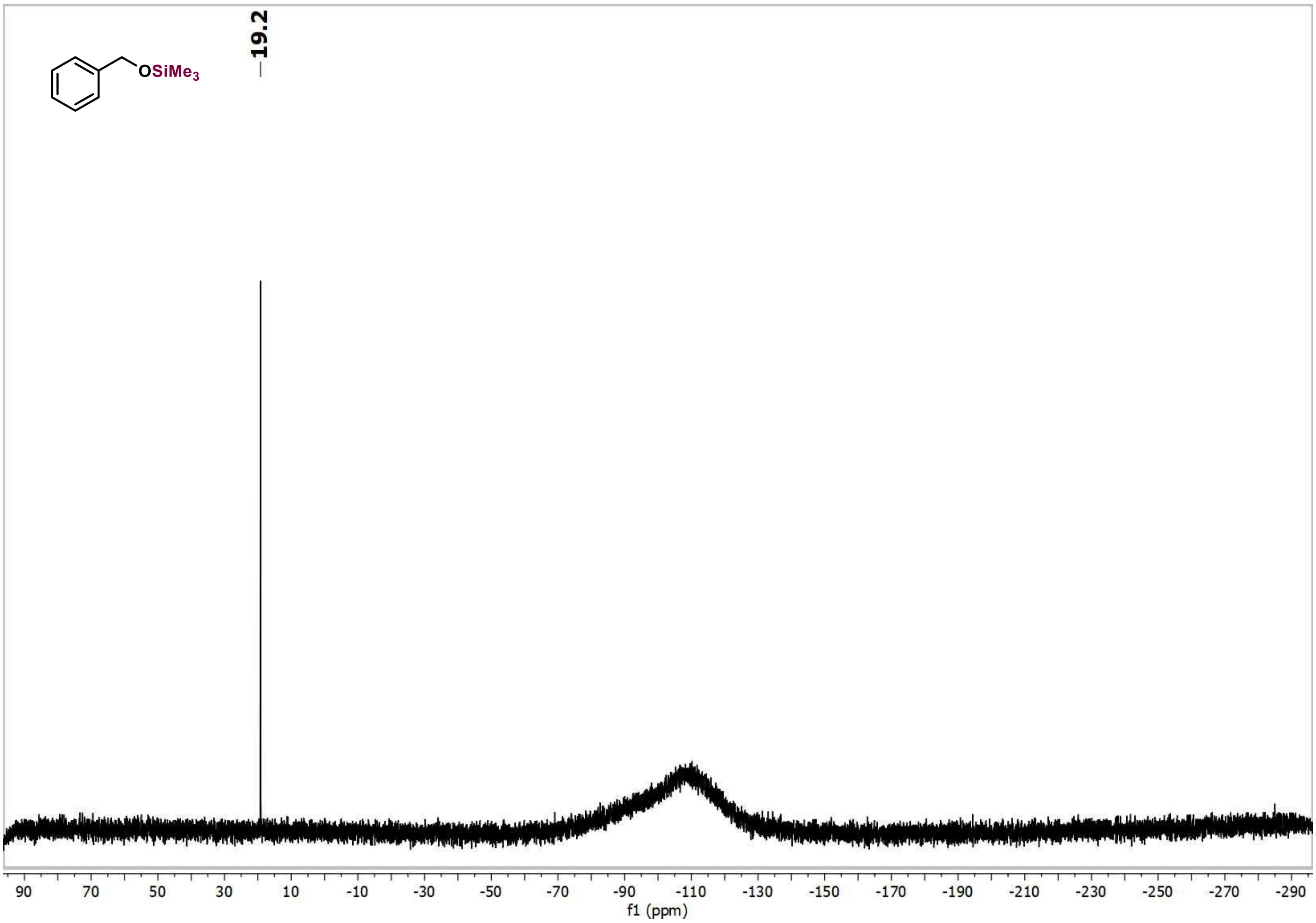

Figure S3. ^{29}Si NMR (79 MHz, Chloroform-d, 25°C) of (benzyloxy)trimethylsilane (**3a**).

((3-Fluorobenzyl)oxy)trimethylsilane (**3b**)

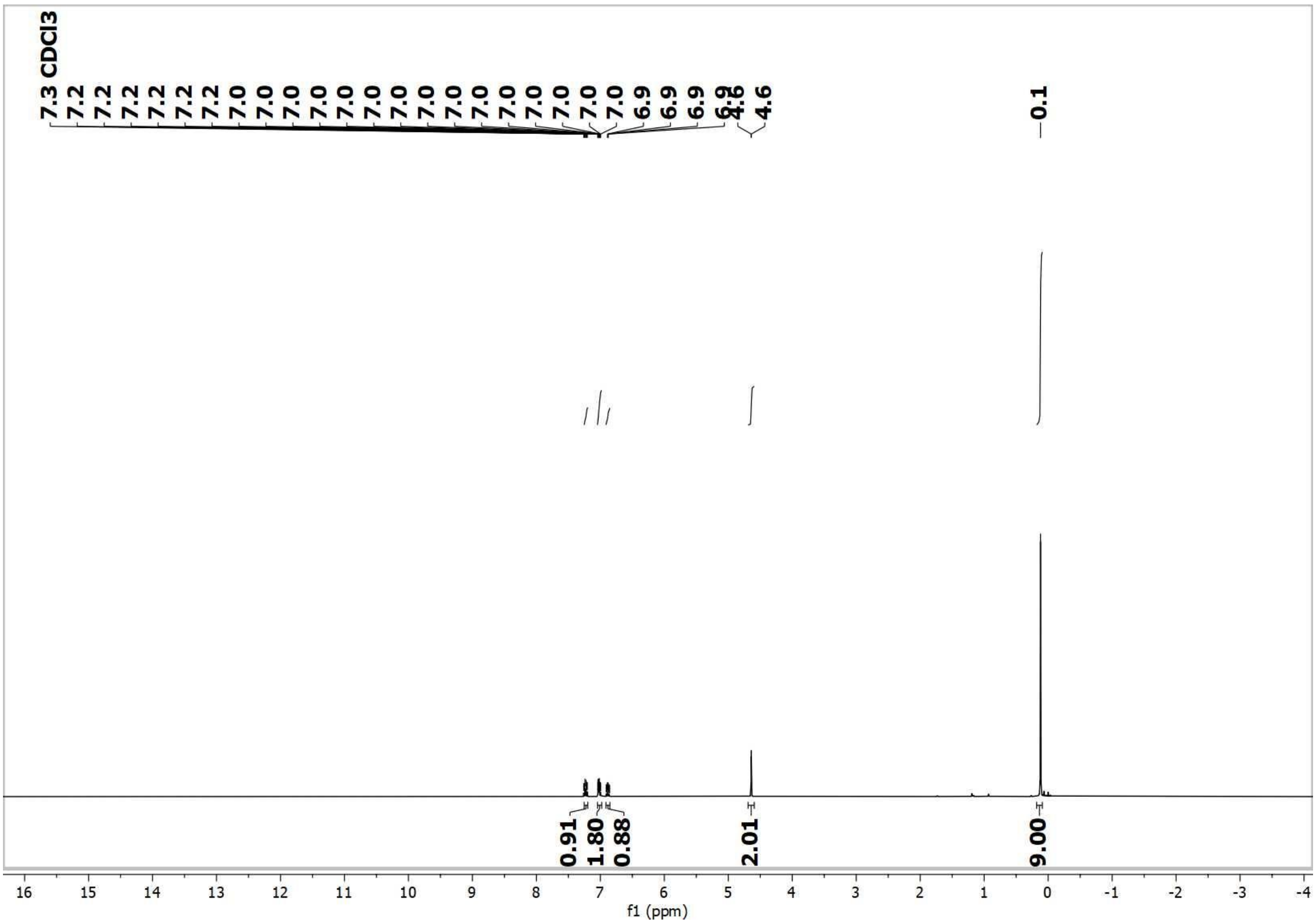

Figure S4. ^{1}H NMR (400 MHz, Chloroform-d, 25°C) of ((3-fluorobenzyl)oxy)trimethylsilane (**3b**).

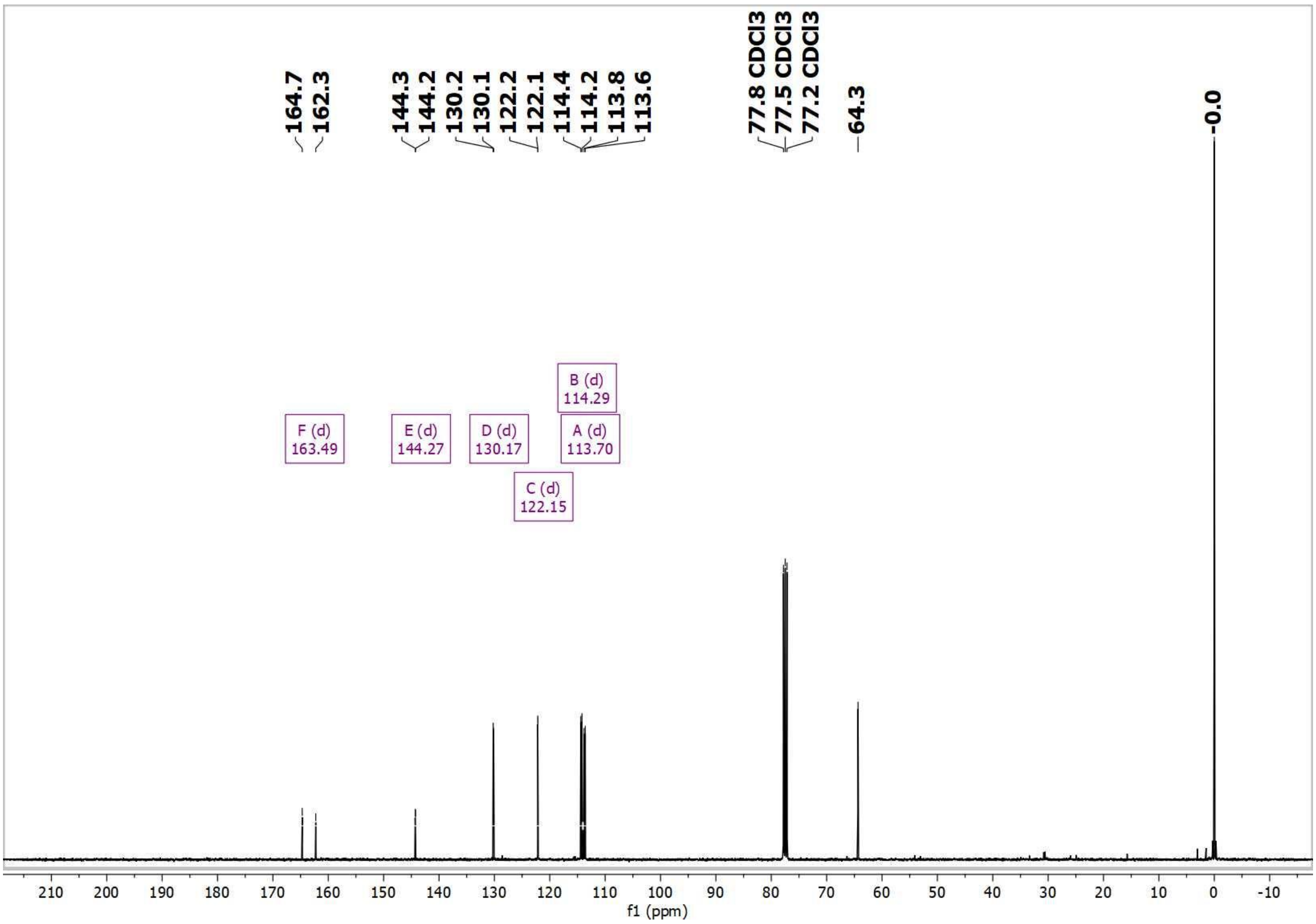

Figure S5. ^{13}C{^{1}H} NMR (101 MHz, Chloroform-d, 25°C) of ((3-fluorobenzyl)oxy)trimethylsilane (**3b**).

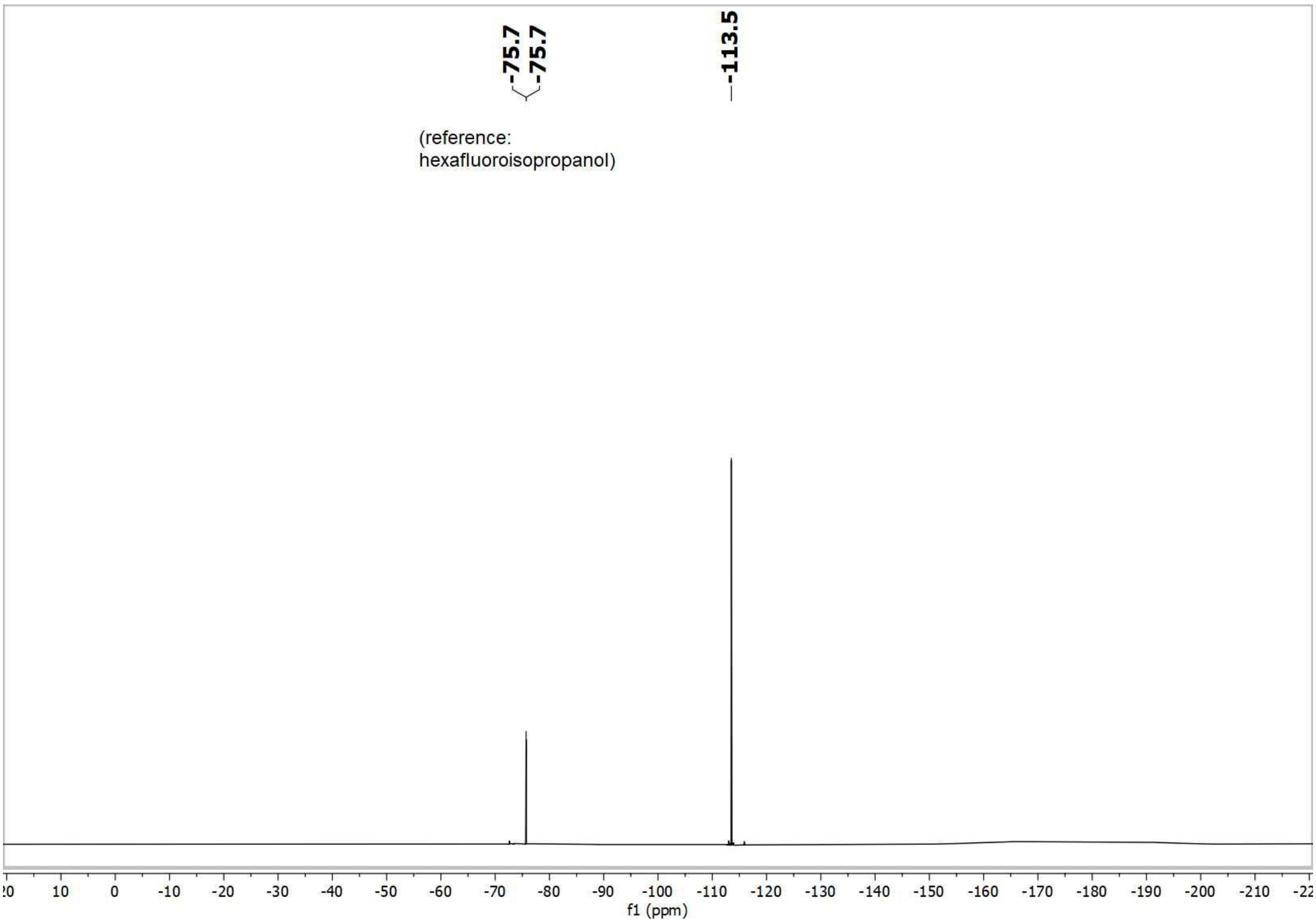

Figure S6. ^{19}F NMR (377 MHz, Chloroform-d, 25°C) of ((3-fluorobenzyl)oxy)trimethylsilane (**3b**).

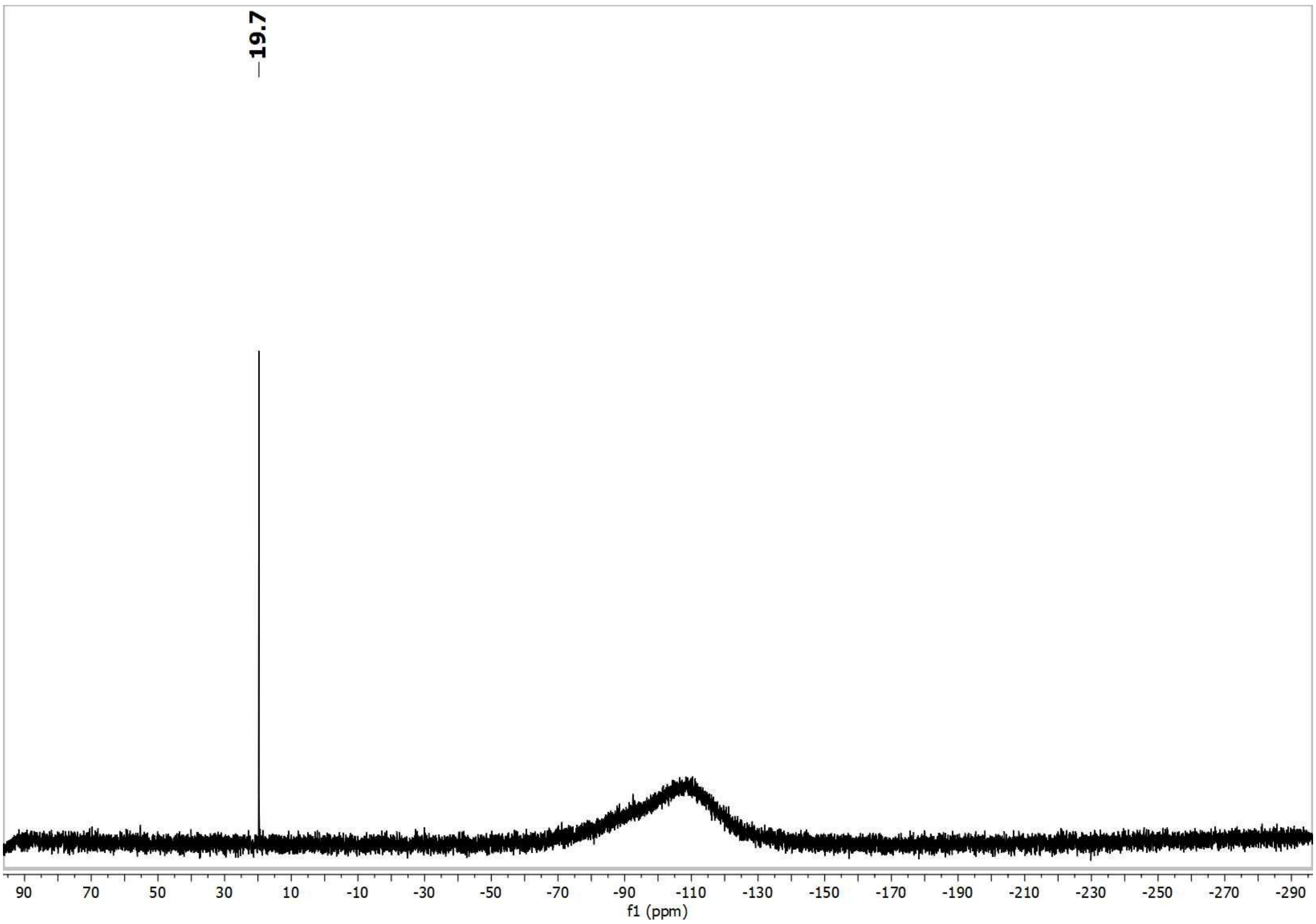

Figure S7. ^{29}Si NMR (79 MHz, Chloroform-d, 25°C) of ((3-fluorobenzyl)oxy)trimethylsilane (**3b**).

((2-Fluorobenzyl)oxy)trimethylsilane (**3c**)

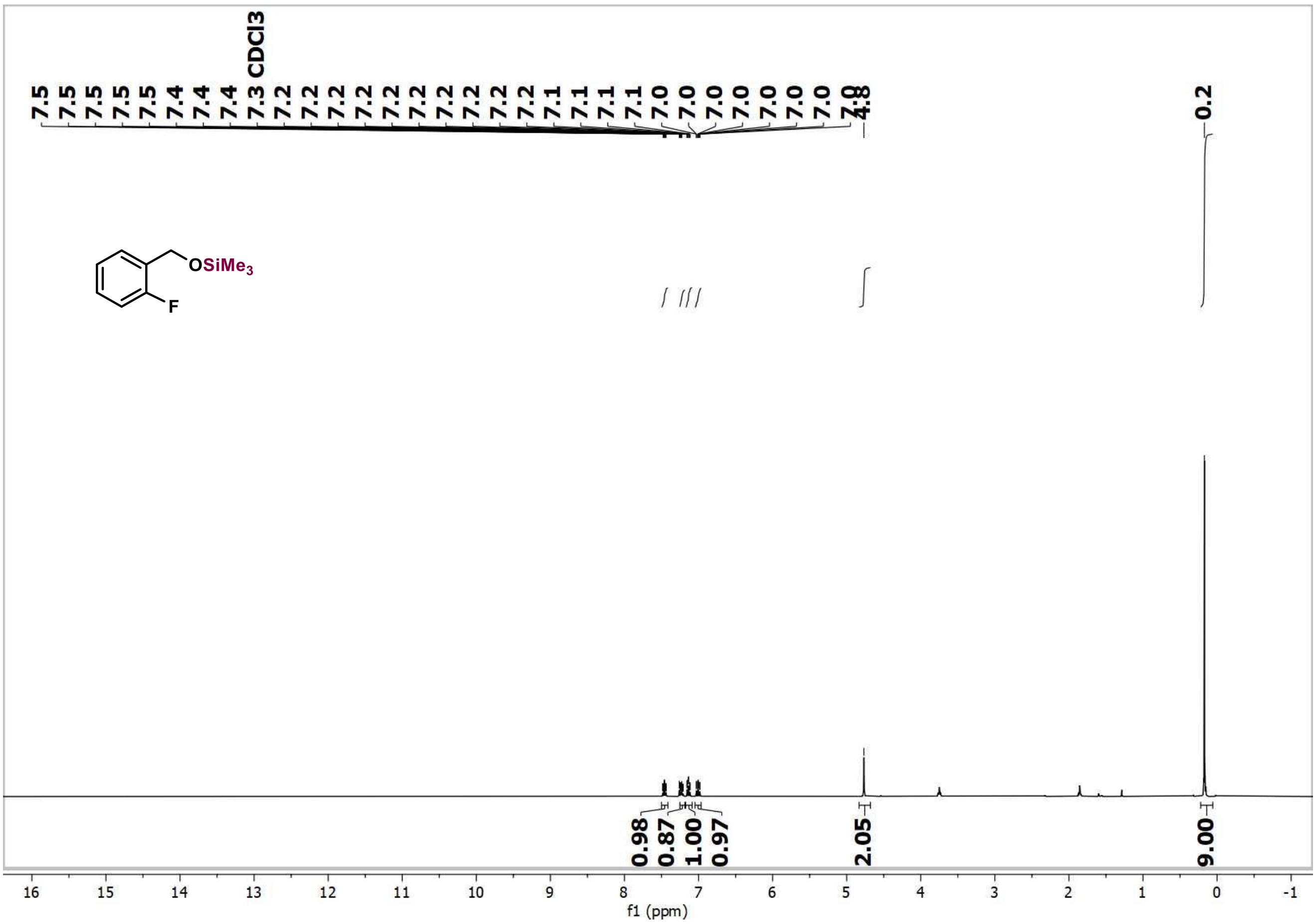

Figure S8. ^{1}H NMR (400 MHz, Chloroform-d, 25°C) of ((2-fluorobenzyl)oxy)trimethylsilane (**3c**).

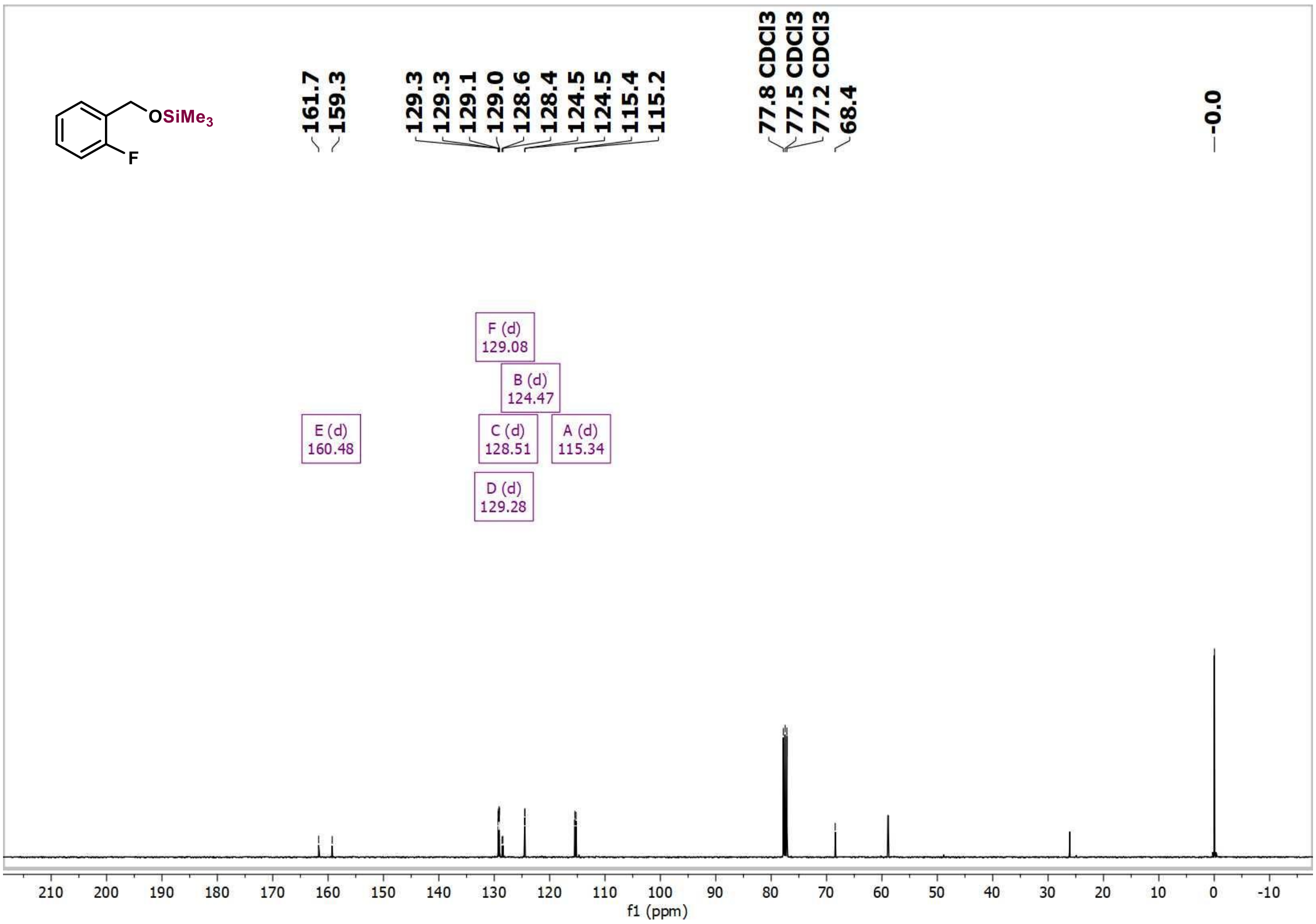

Figure S9. ^{13}C NMR (101 MHz, Chloroform-d, 25°C) of ((2-fluorobenzyl)oxy)trimethylsilane (**3c**).

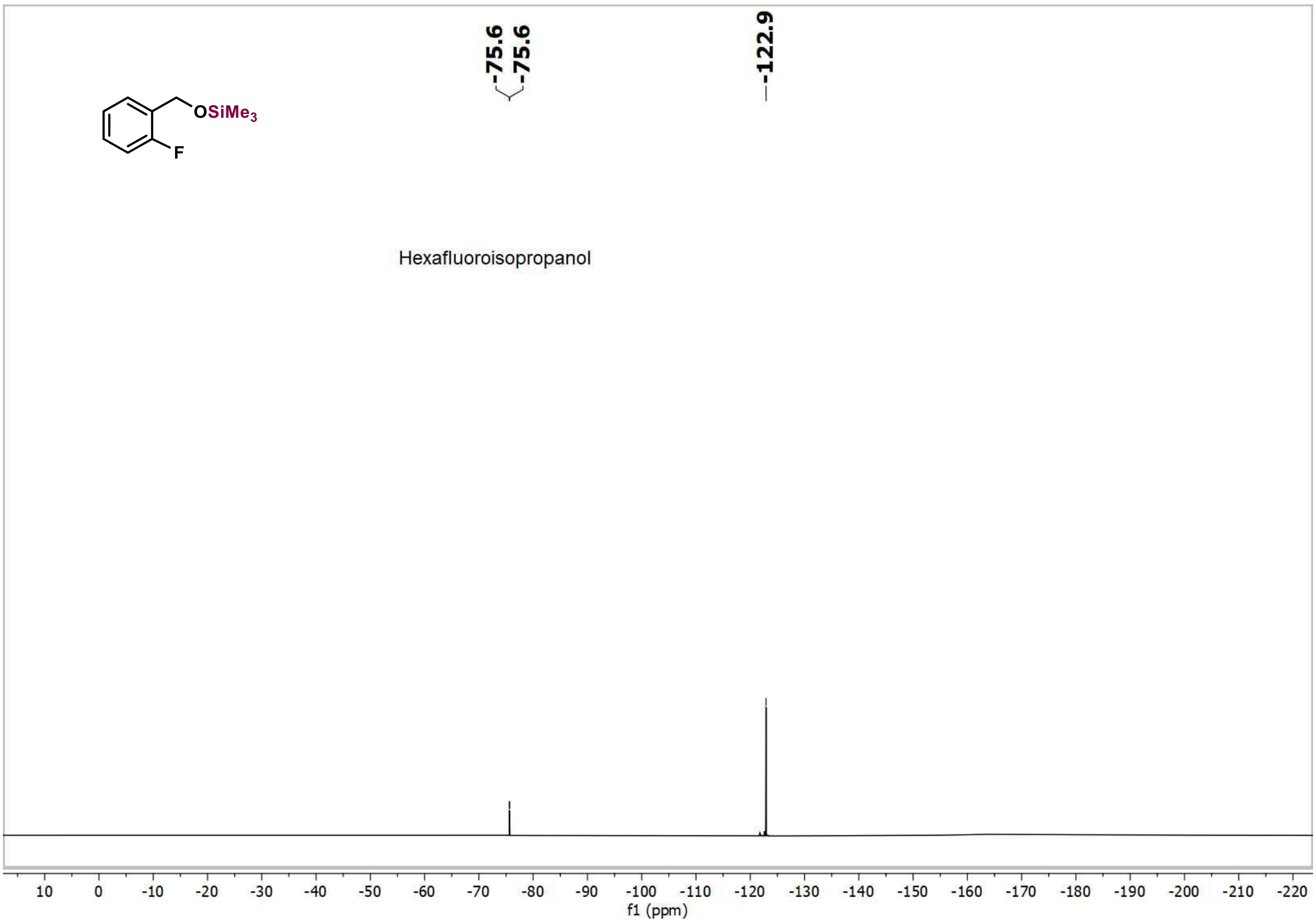

Figure S10. ^{19}F NMR (377 MHz, Chloroform-d, 25°C) of ((2-fluorobenzyl)oxy)trimethylsilane (**3c**).

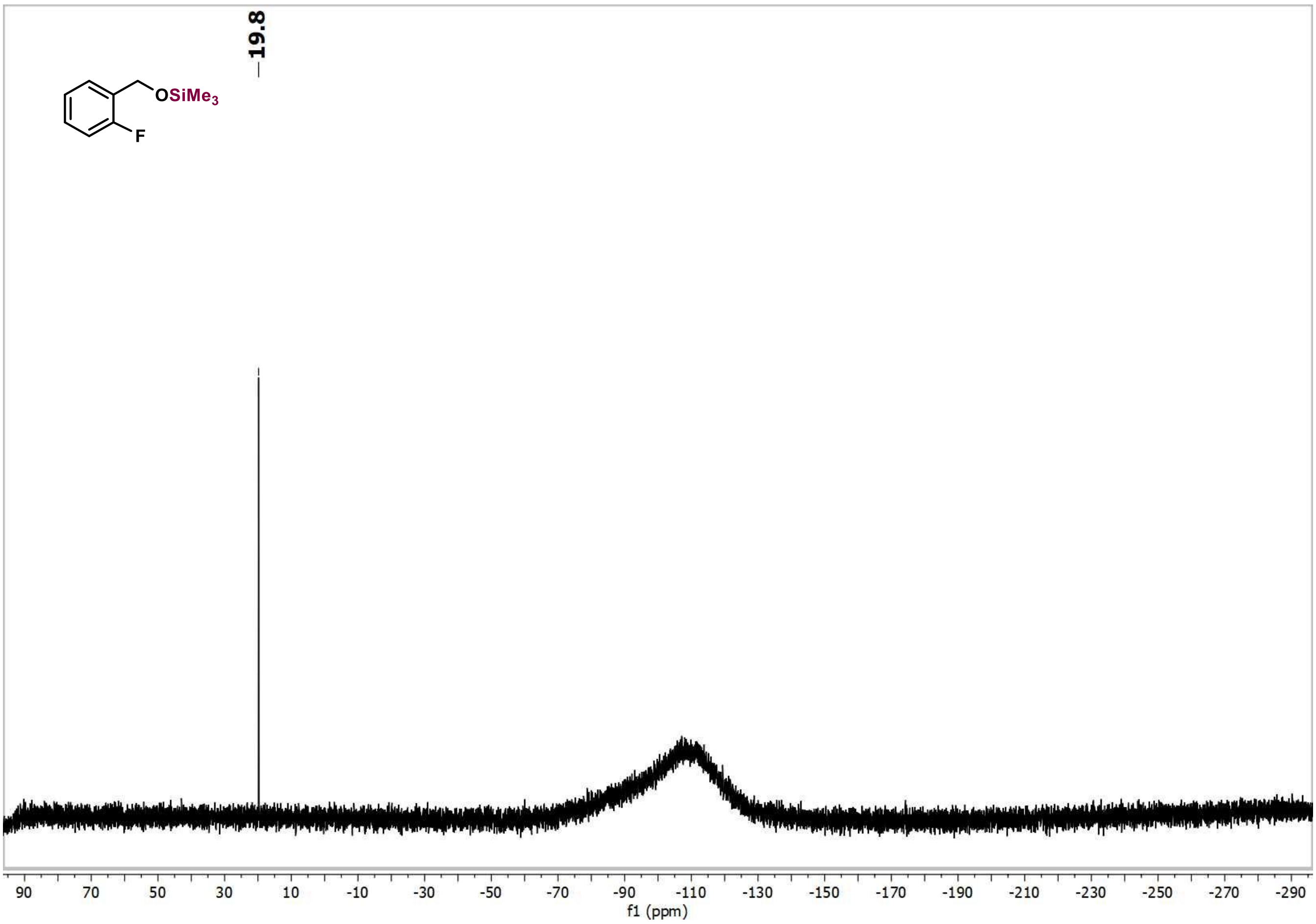

Figure S11. ^{29}Si NMR (79 MHz, Chloroform-d, 25°C) of ((2-fluorobenzyl)oxy)trimethylsilane (**3c**).

((3-Iodobenzyl)oxy)trimethylsilane (**3d**)

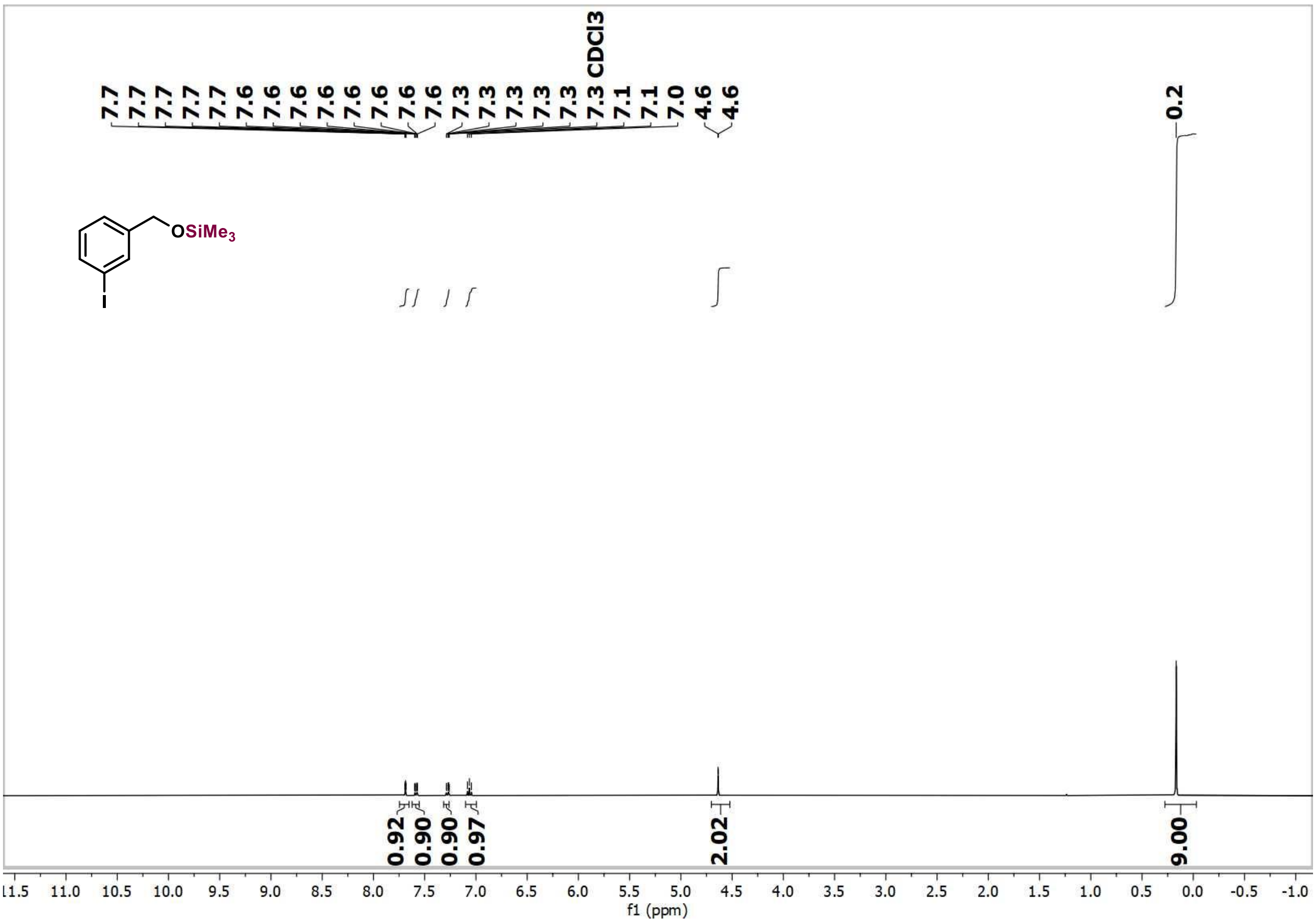

Figure S12. ^{1}H NMR (400 MHz, Chloroform-d, 25°C) of ((3-iodobenzyl)oxy)trimethylsilane (**3d**).

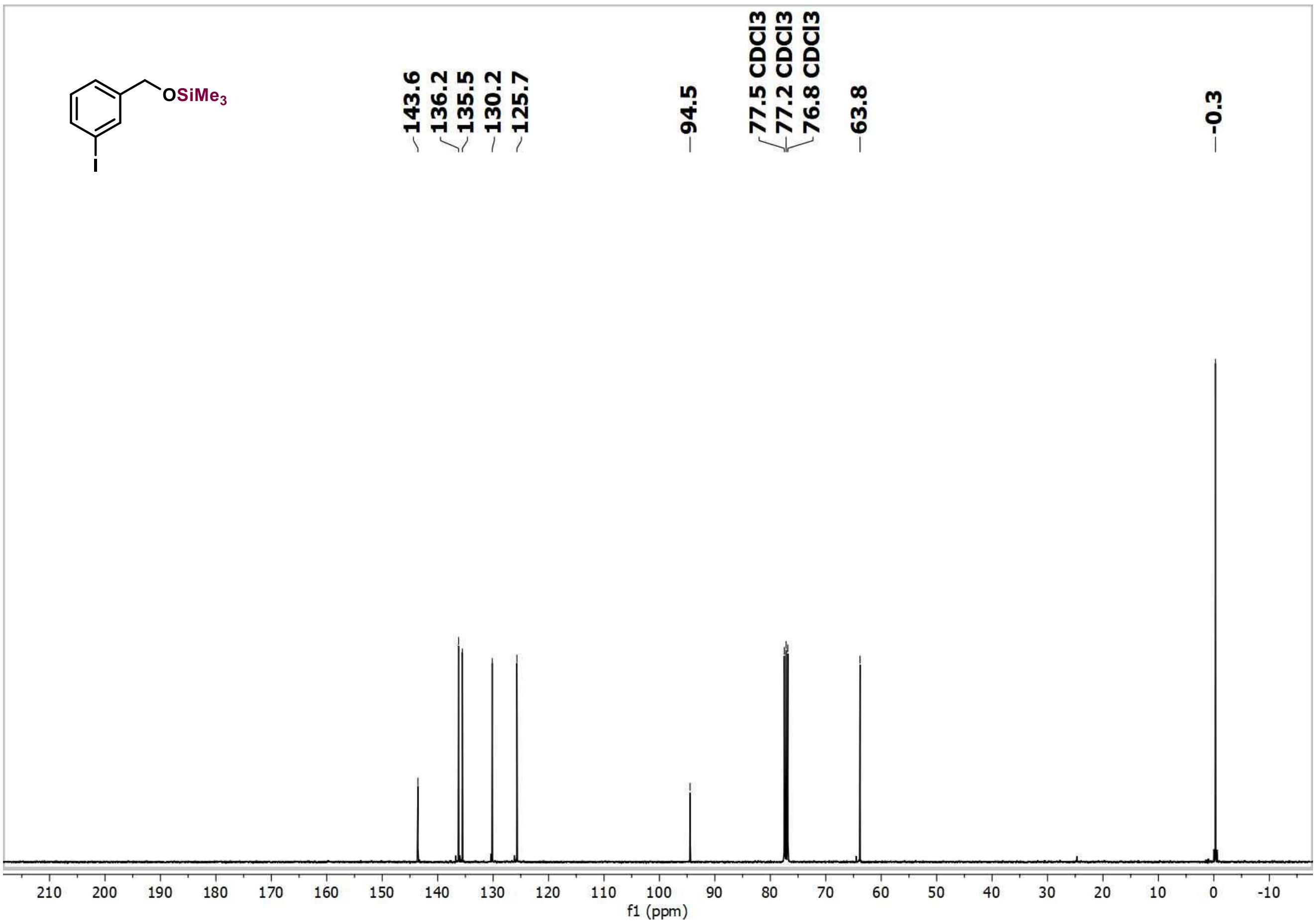

Figure S13. ^{13}C NMR (101 MHz, Chloroform-d, 25°C) of ((3-iodobenzyl)oxy)trimethylsilane (**3d**).

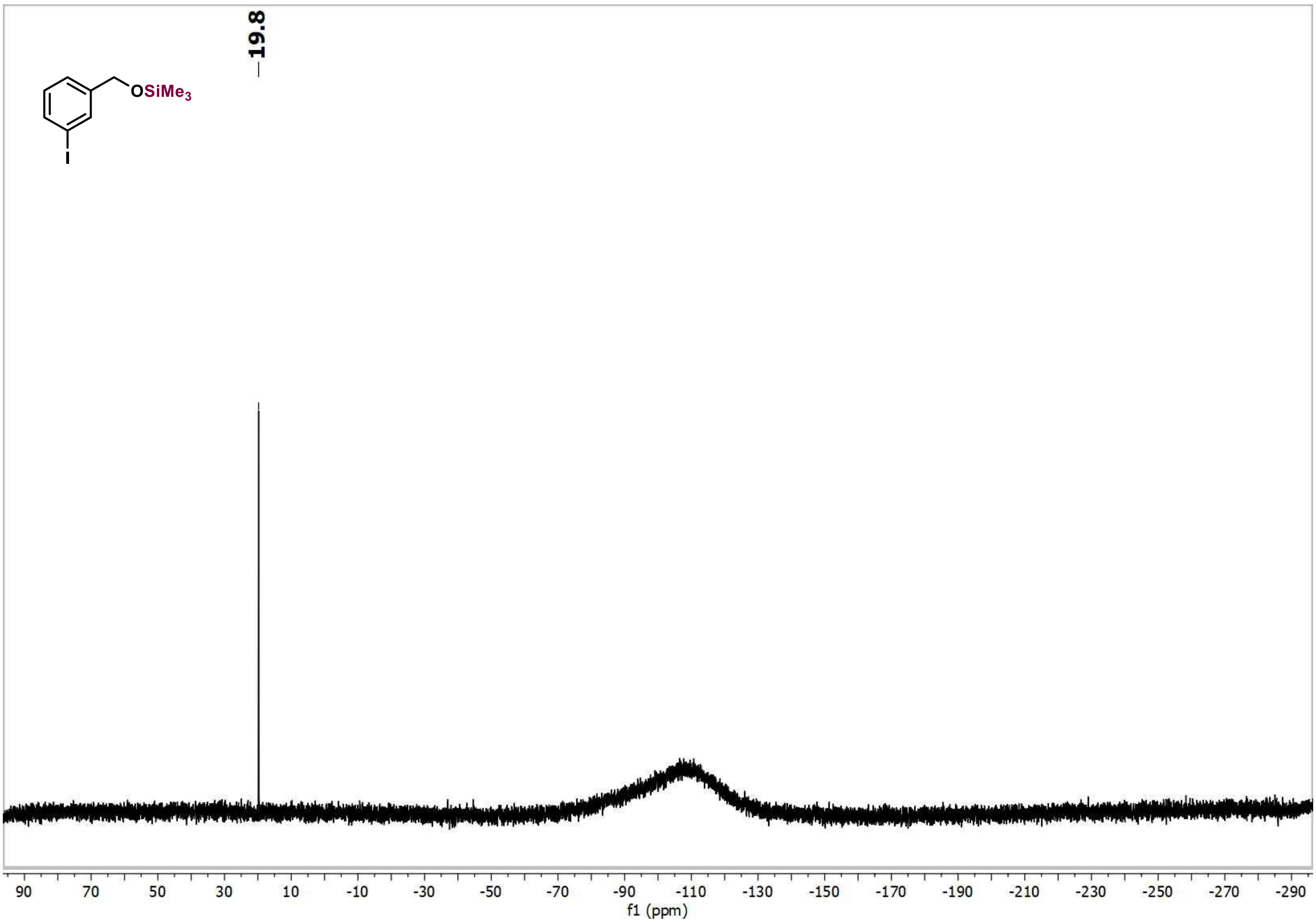

Figure S14. ^{29}Si NMR (79 MHz, Chloroform-d, 25°C) of ((3-iodobenzyl)oxy)trimethylsilane (3d).

((3,5-Dichlorobenzyl)oxy)trimethylsilane (**3e**)

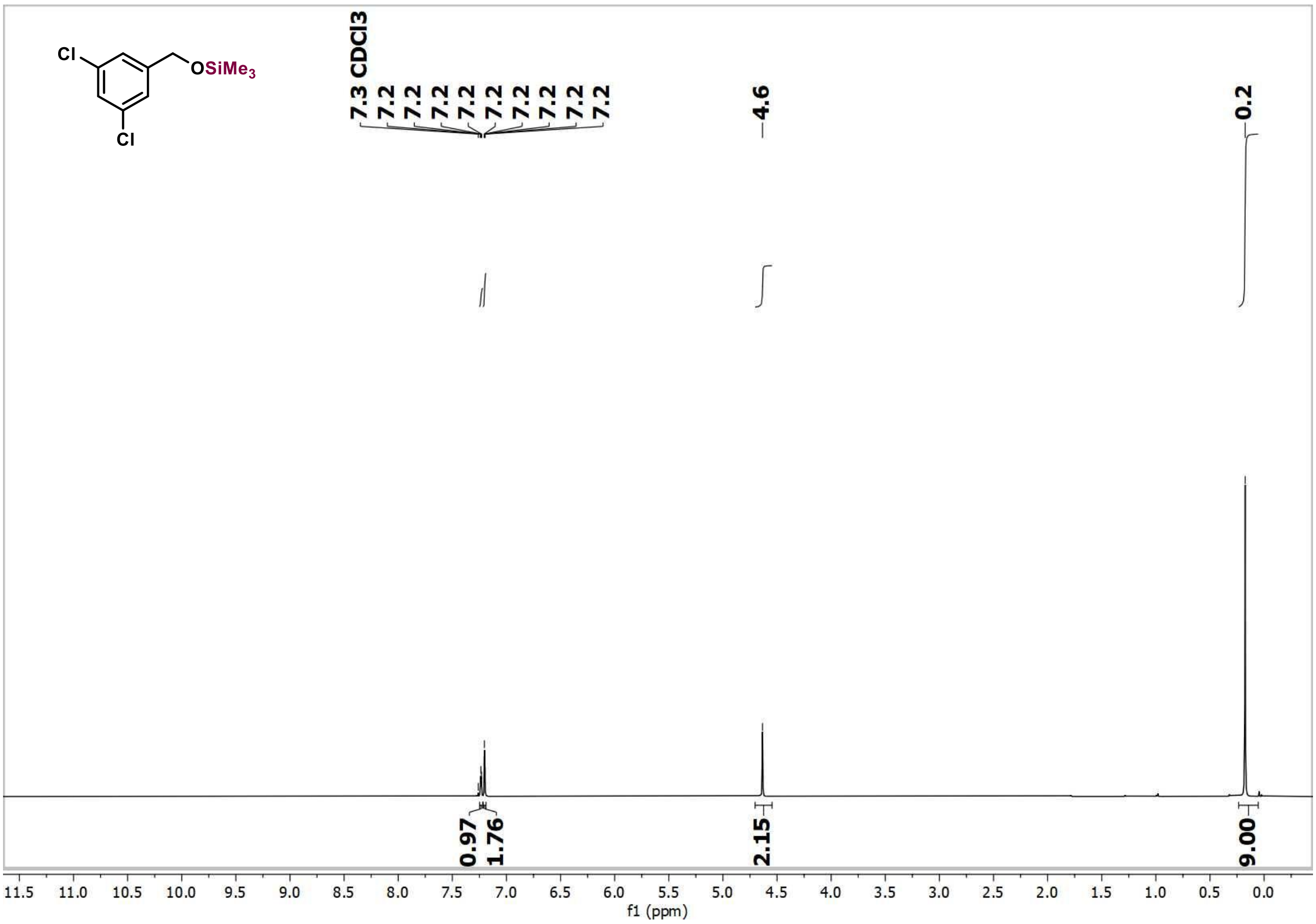

Figure S15. ^{1}H NMR (400 MHz, Chloroform-d, 25°C) of ((3,5-dichlorobenzyl)oxy)trimethylsilane (**3e**).

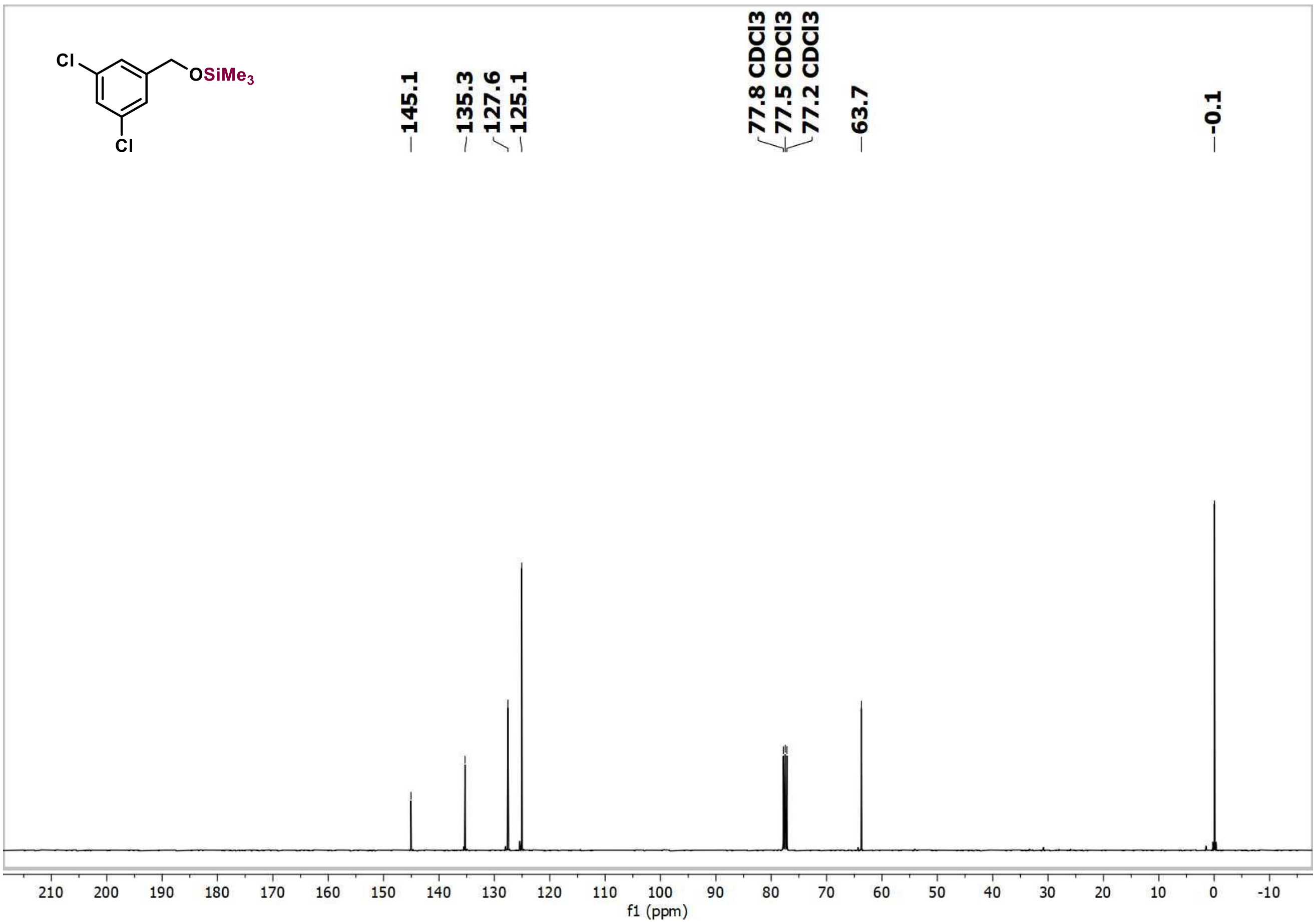

Figure S16. ^{13}C NMR (101 MHz, Chloroform-d, 25°C) of ((3,5-dichlorobenzyl)oxy)trimethylsilane (**3e**).

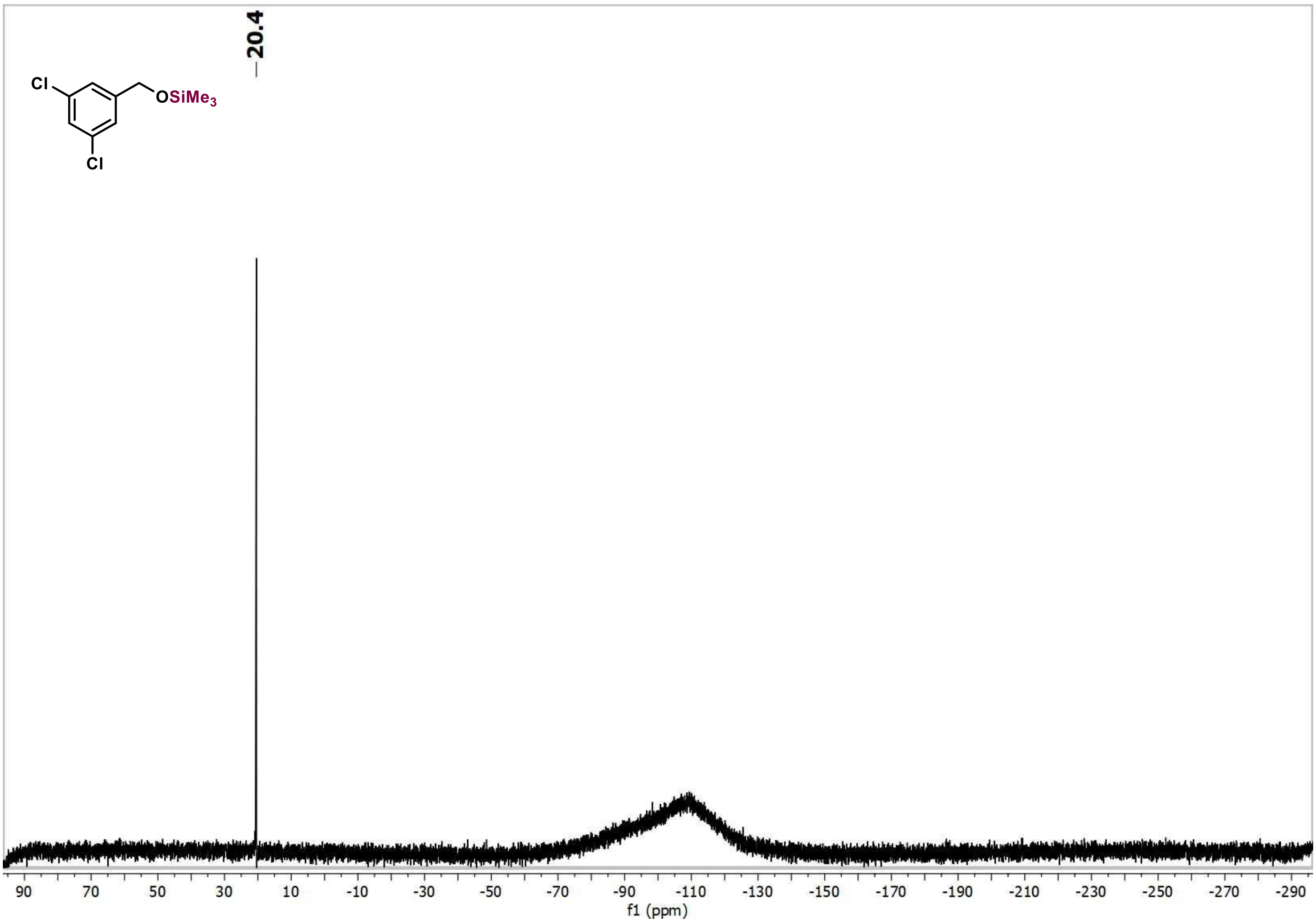

Figure S17. ^{29}Si NMR (79 MHz, Chloroform-d, 25°C) of ((3,5-dichlorobenzyl)oxy)trimethylsilane (**3e**).

Trimethyl((4-(trifluoromethyl)benzyl)oxy)silane (**3f**)

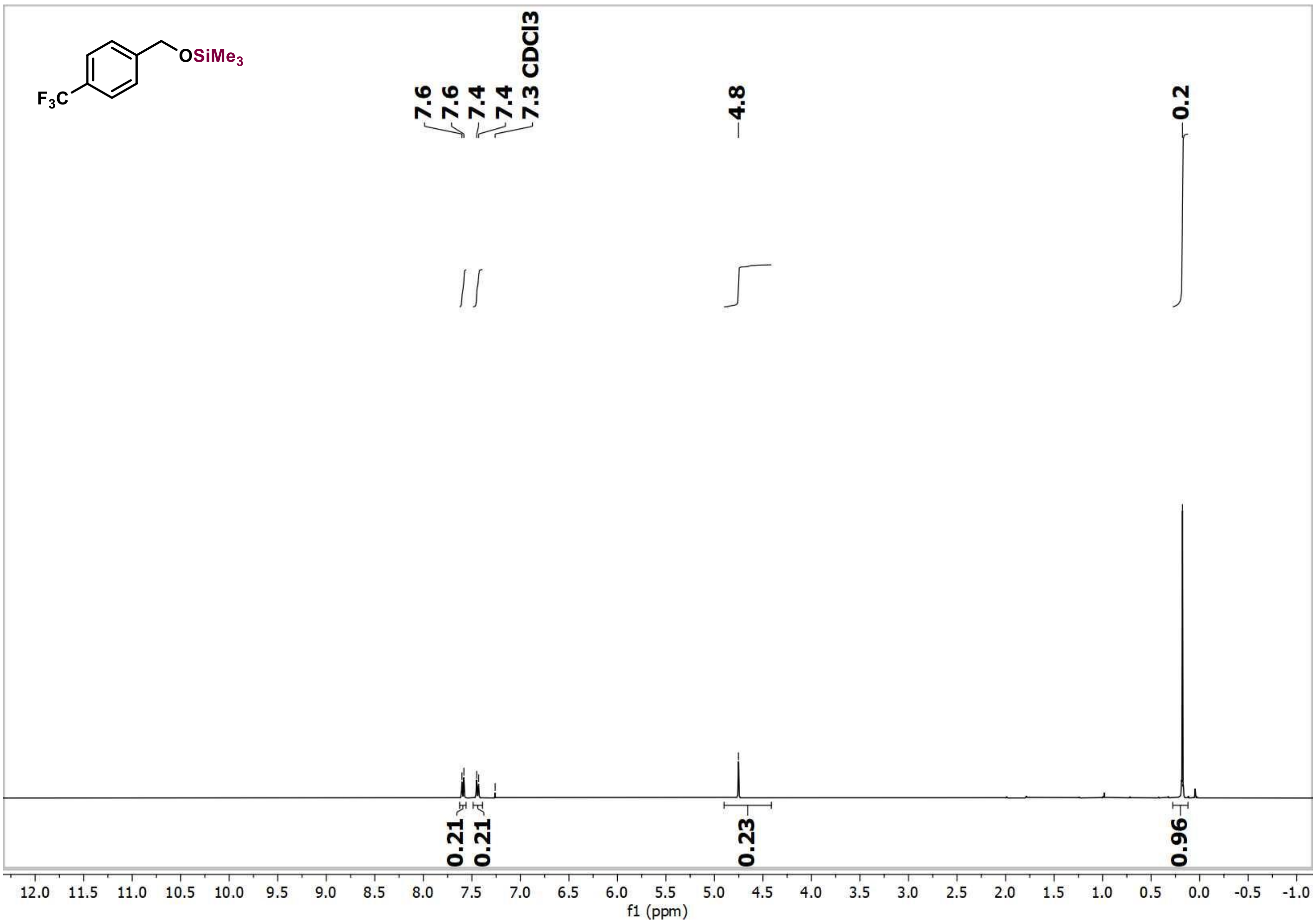

Figure S18. ^{1}H NMR (400 MHz, Chloroform-d, 25°C) of trimethyl((4-(trifluoromethyl)benzyl)oxy)silane (**3f**).

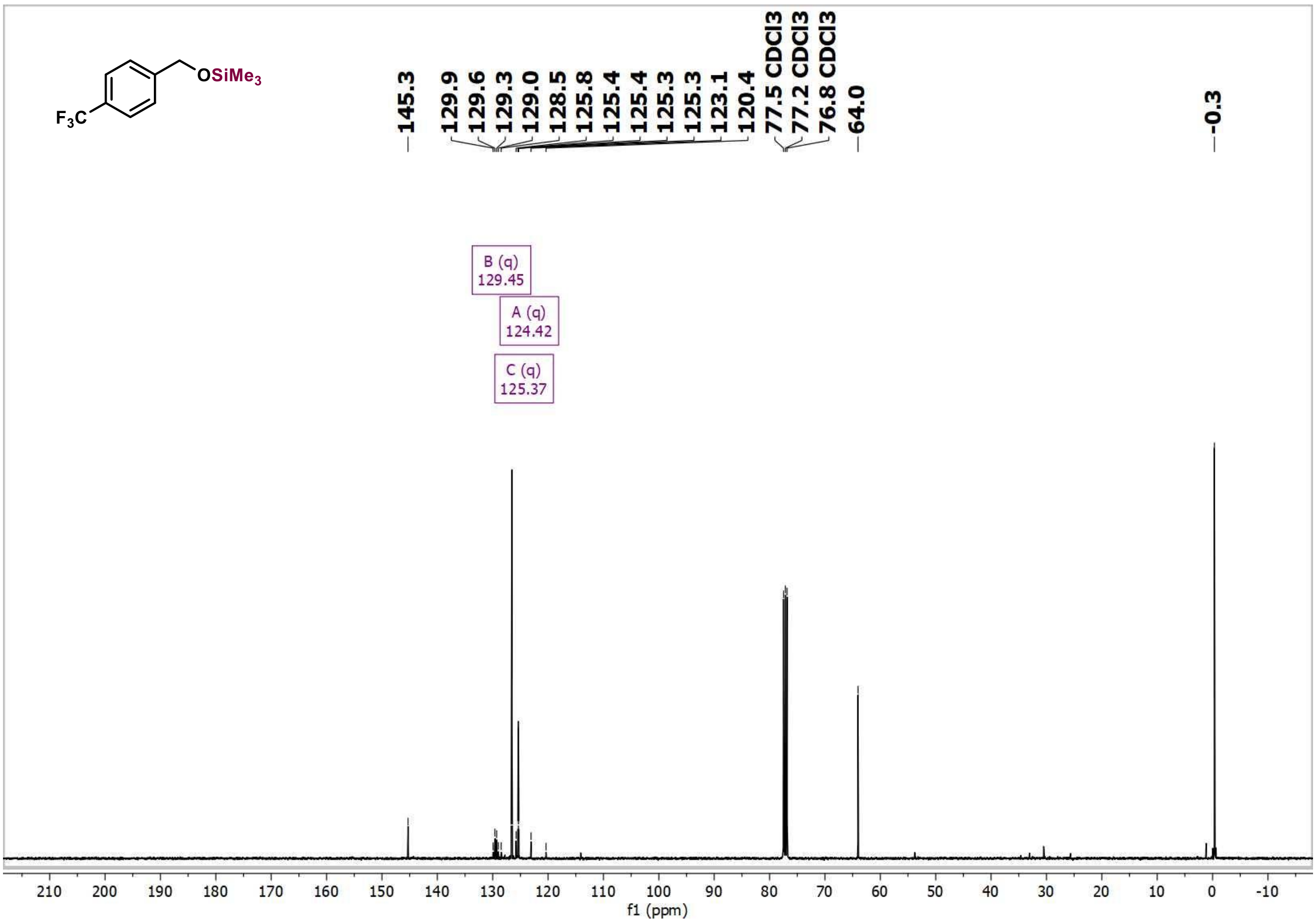

Figure S19. ^{13}C NMR (101 MHz, Chloroform-d, 25°C) of trimethyl((4-(trifluoromethyl)benzyl)oxy)silane (**3f**).

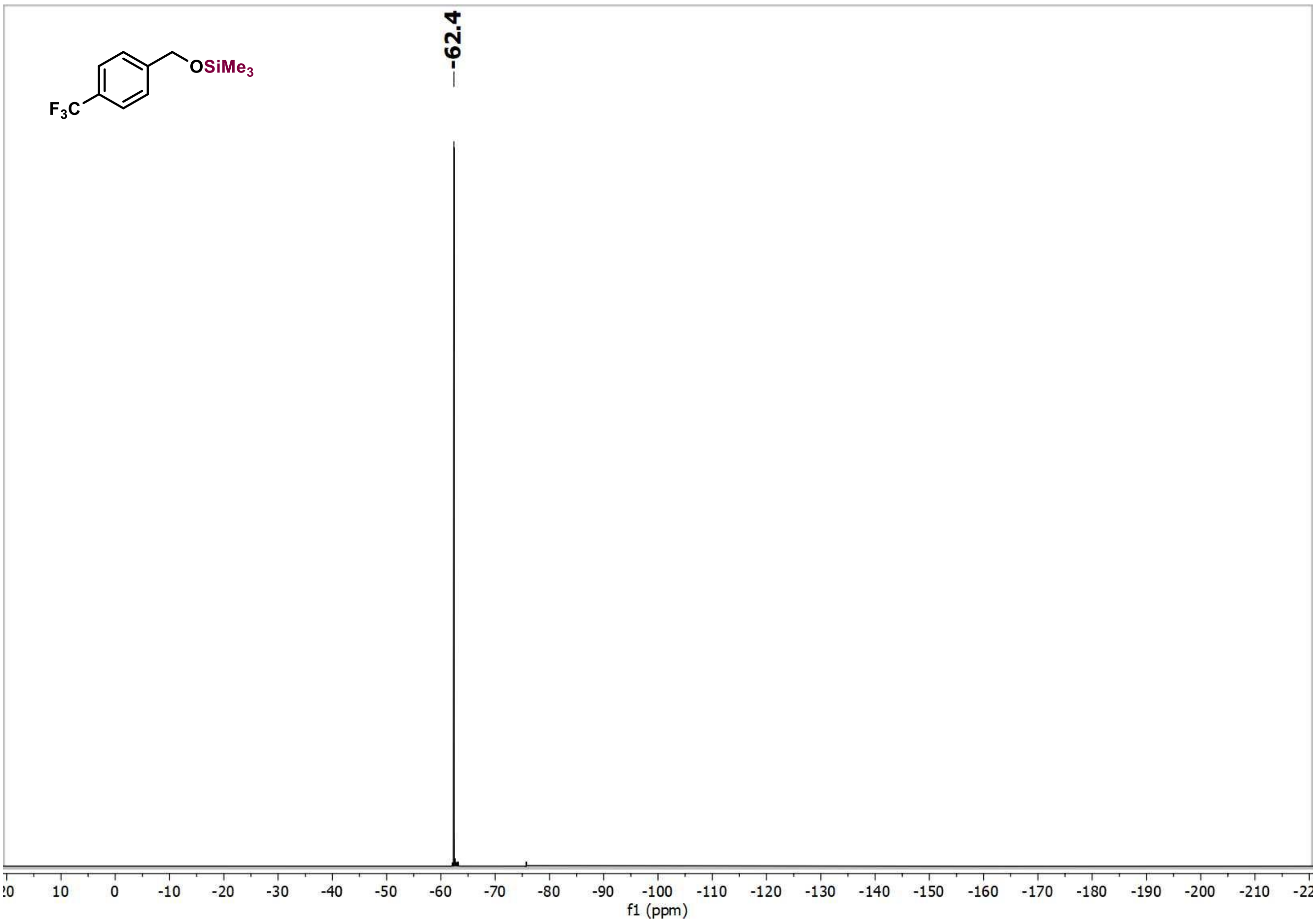

Figure S20. ^{19}F NMR (377 MHz, Chloroform-d, 25°C) of trimethyl((4-(trifluoromethyl)benzyl)oxy)silane (**3f**).

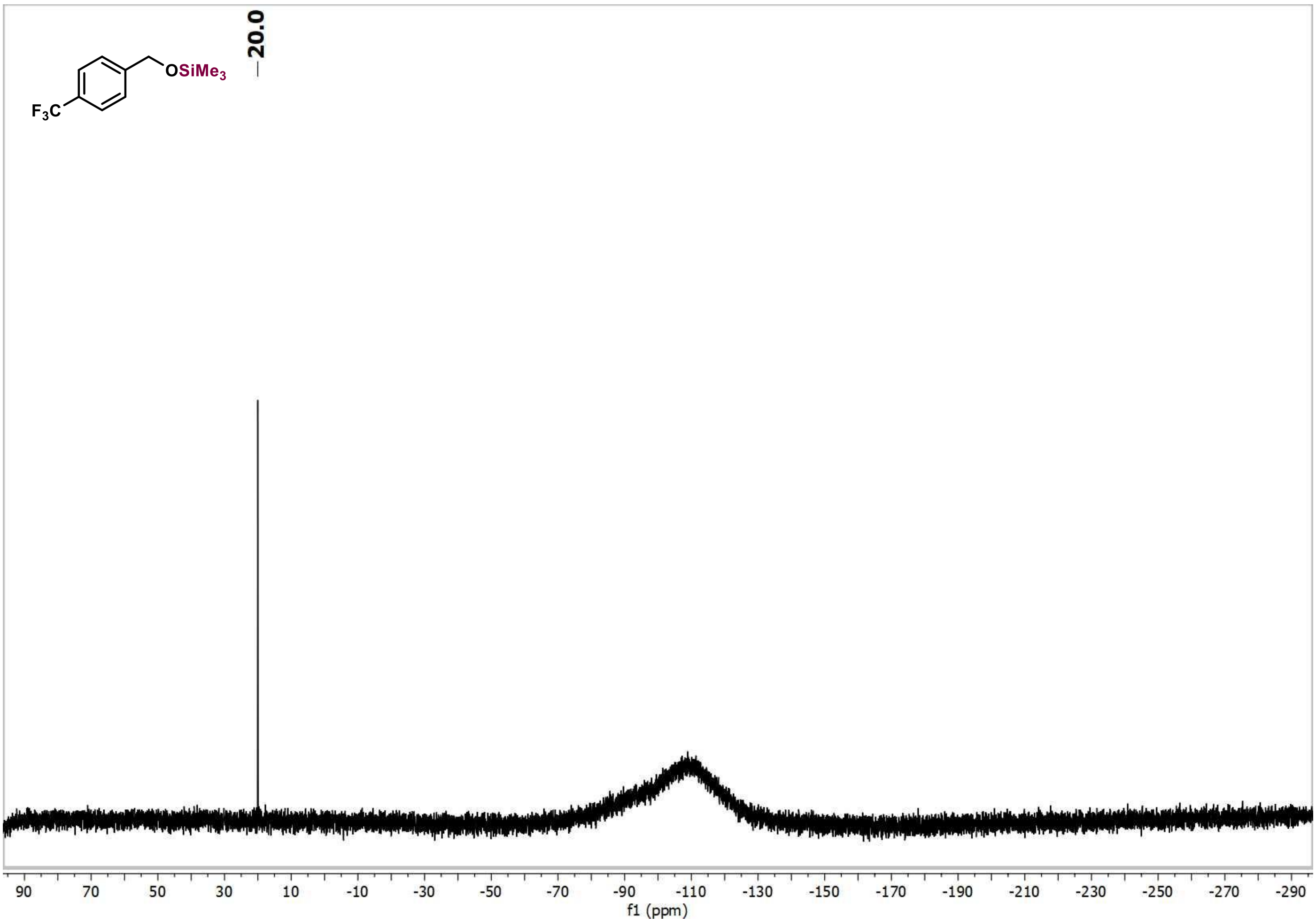

Figure S21. ^{29}Si NMR (79 MHz, Chloroform-d, 25°C) of trimethyl((4-(trifluoromethyl)benzyl)oxy)silane (**3f**).

4-(((Trimethylsilyl)oxy)methyl)benzonitrile (**3g**)

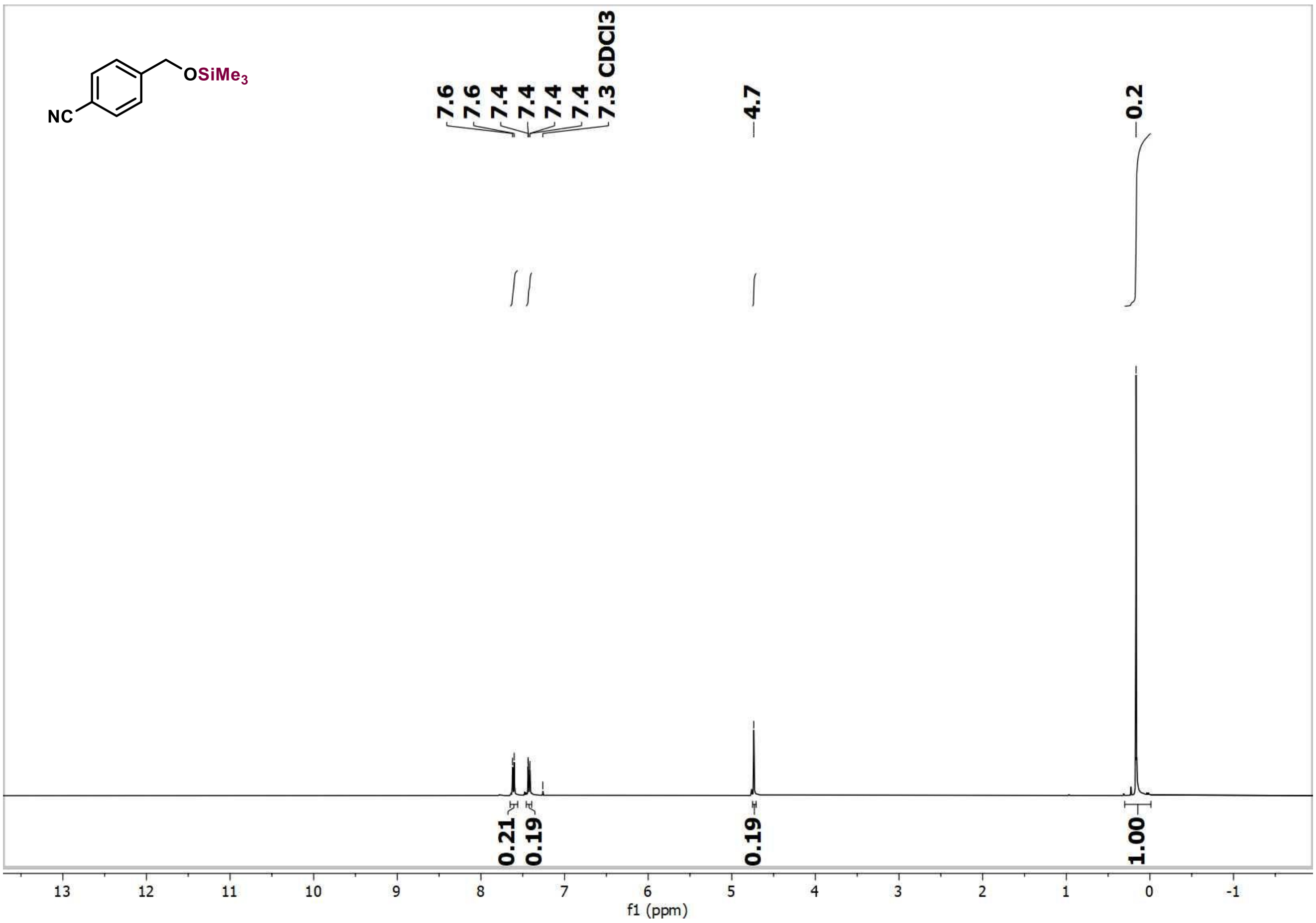

Figure S22. ^{1}H NMR (400 MHz, Chloroform-d, 25°C) of 4-(((trimethylsilyl)oxy)methyl)benzonitrile (**3g**)

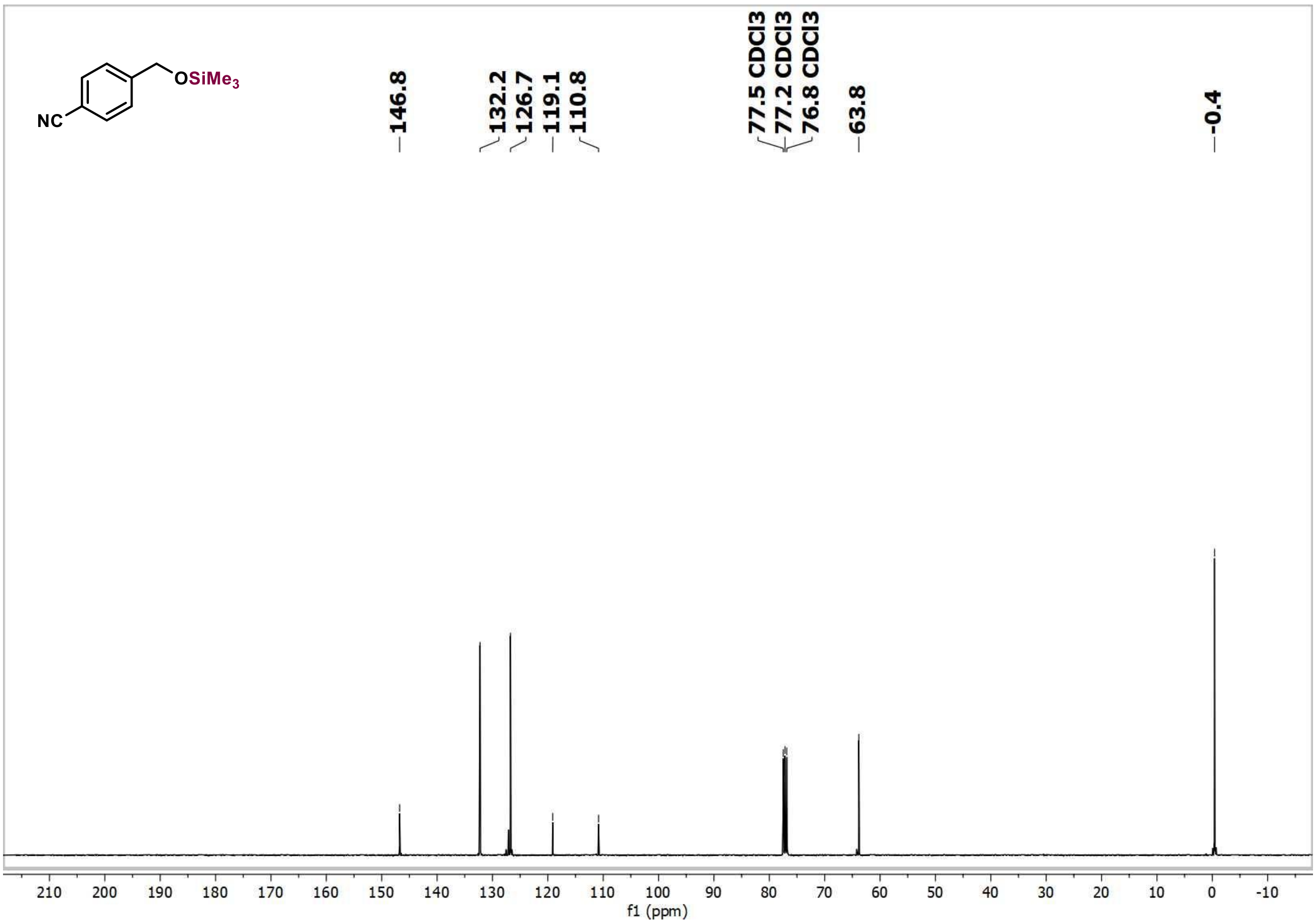

Figure S23. ^{13}C NMR (101 MHz, Chloroform-d, 25°C) of 4-(((trimethylsilyl)oxy)methyl)benzonitrile (**3g**)

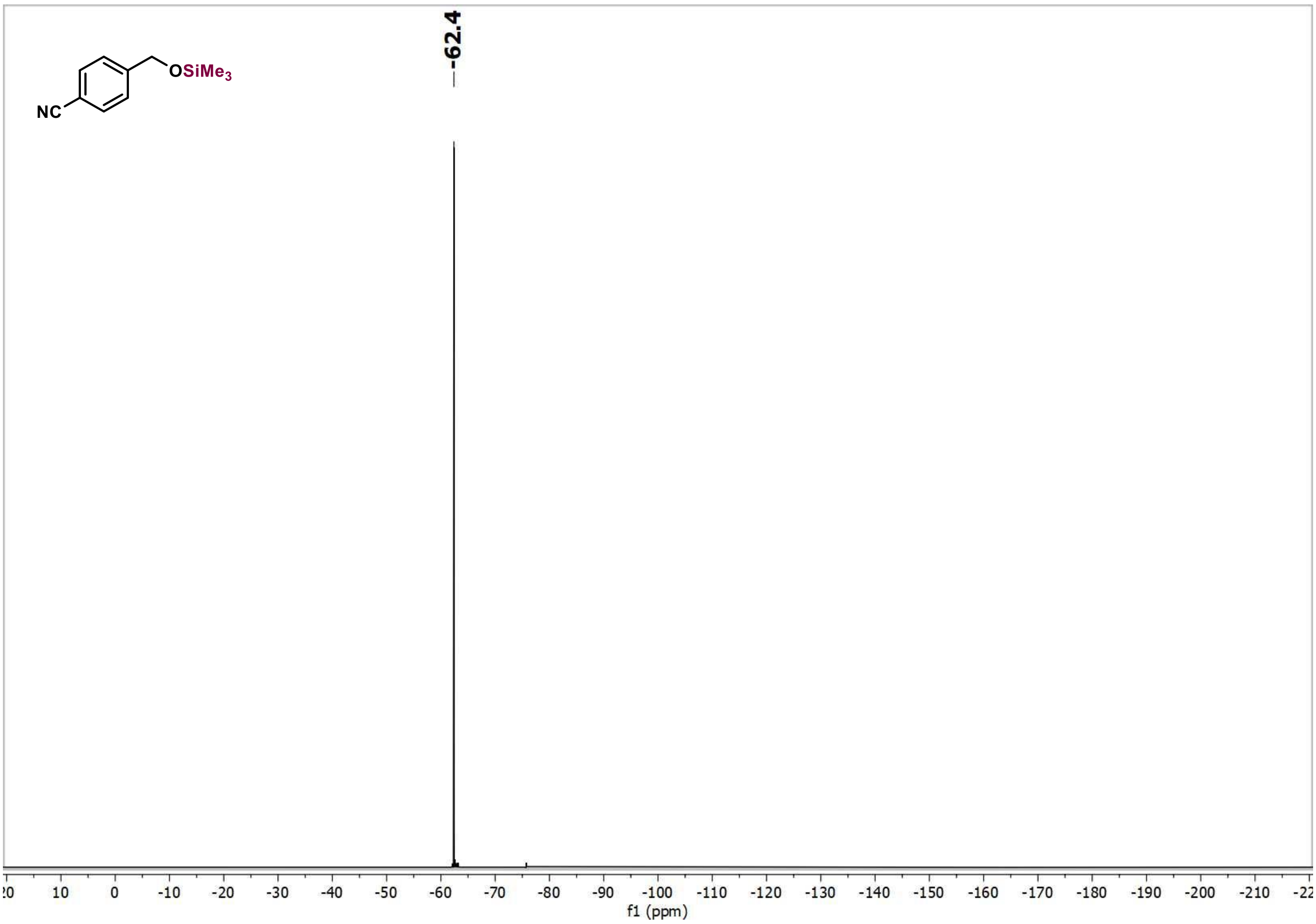

Figure S24. ^{19}F NMR (377 MHz, Chloroform-d, 25°C) of 4-(((trimethylsilyl)oxy)methyl)benzonitrile (**3g**).

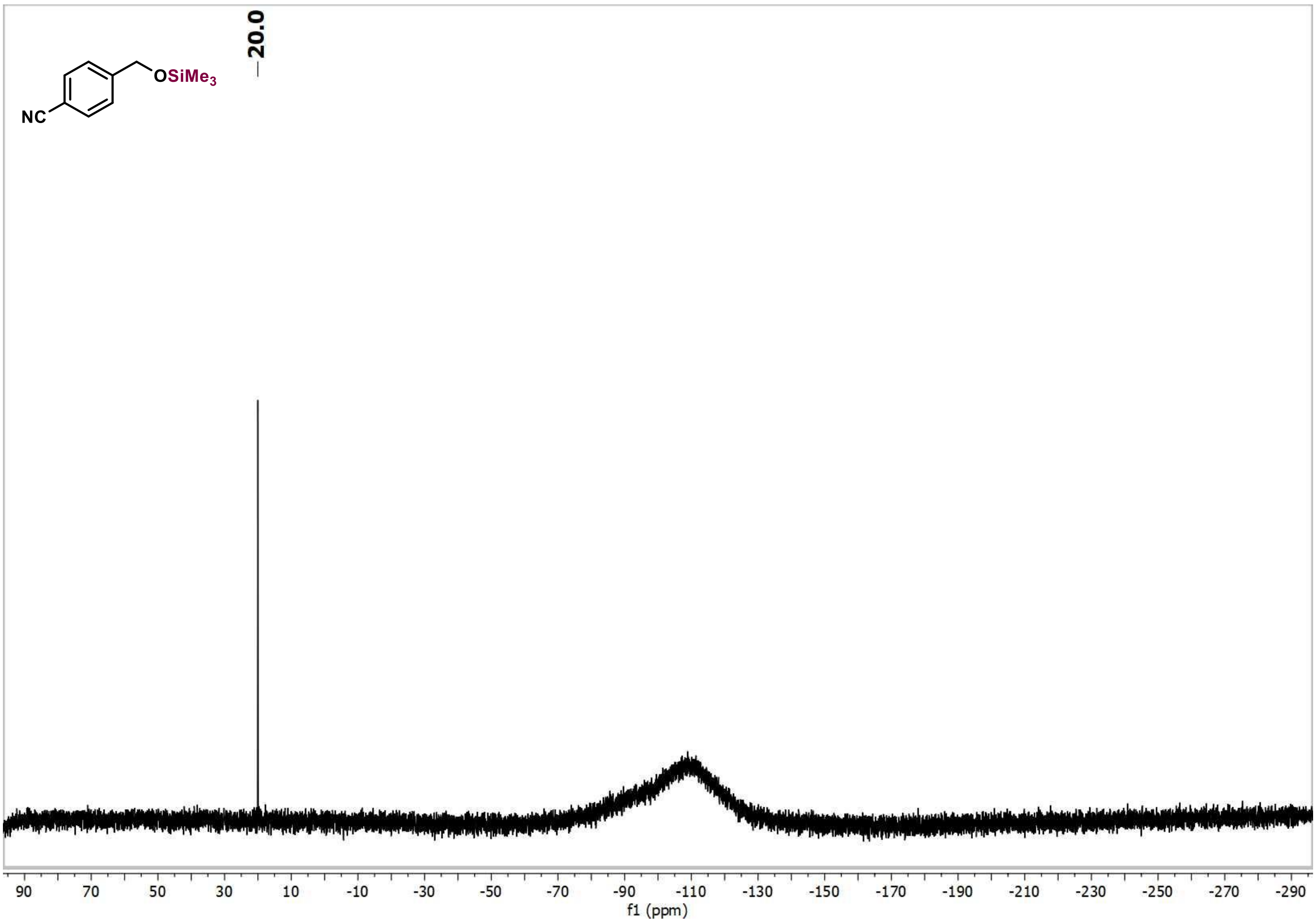

Figure S25. ^{29}Si NMR (79 MHz, Chloroform-d, 25°C) of 4-(((trimethylsilyl)oxy)methyl)benzonitrile (**3g**).

(*S*)-(3-Chloro-1-phenylpropoxy)trimethylsilane (**3h**)

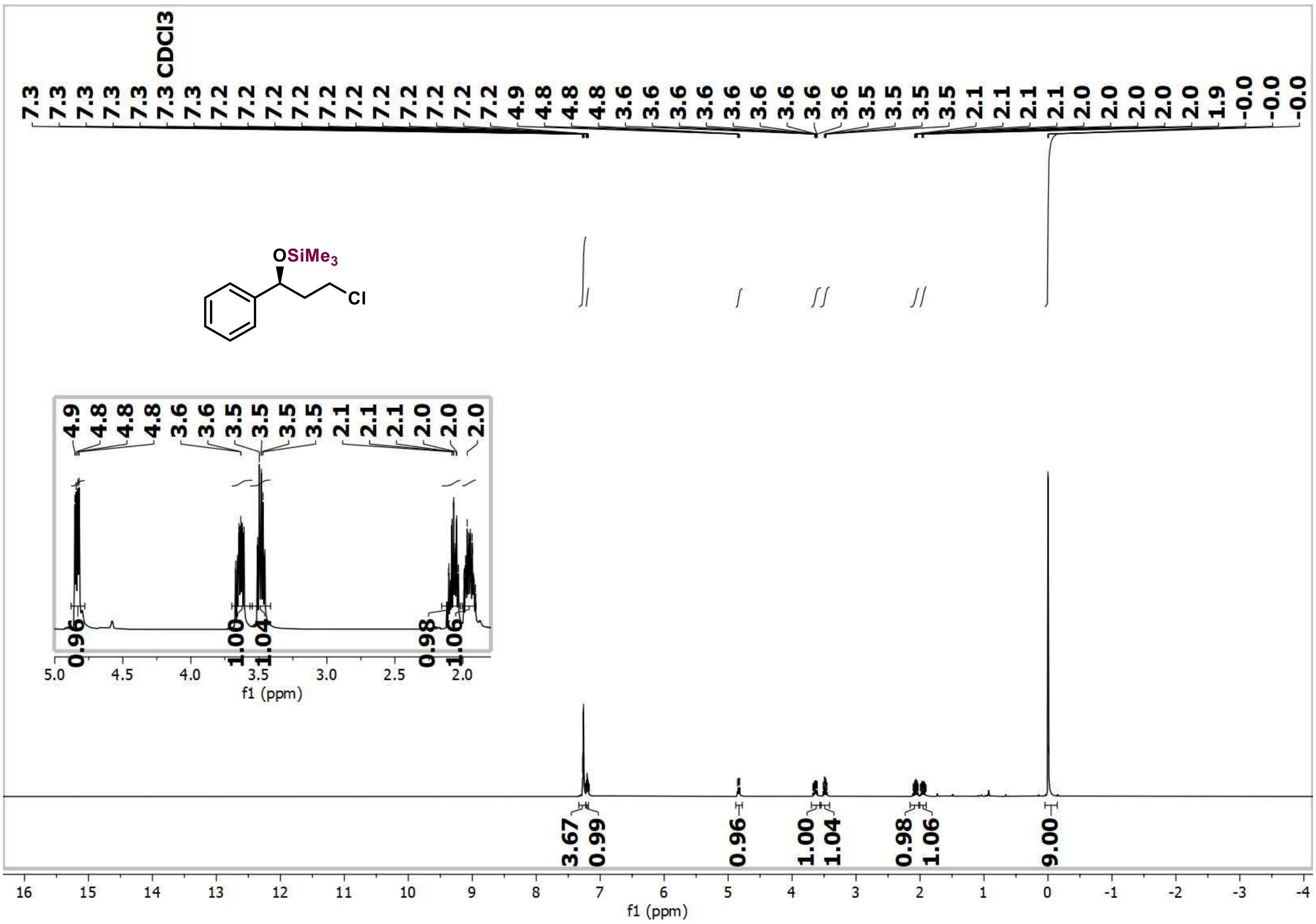

Figure S26. ^{1}H NMR (400 MHz, Chloroform-d, 25°C) of (*S*)-(3-chloro-1-phenylpropoxy)trimethylsilane (**3h**).

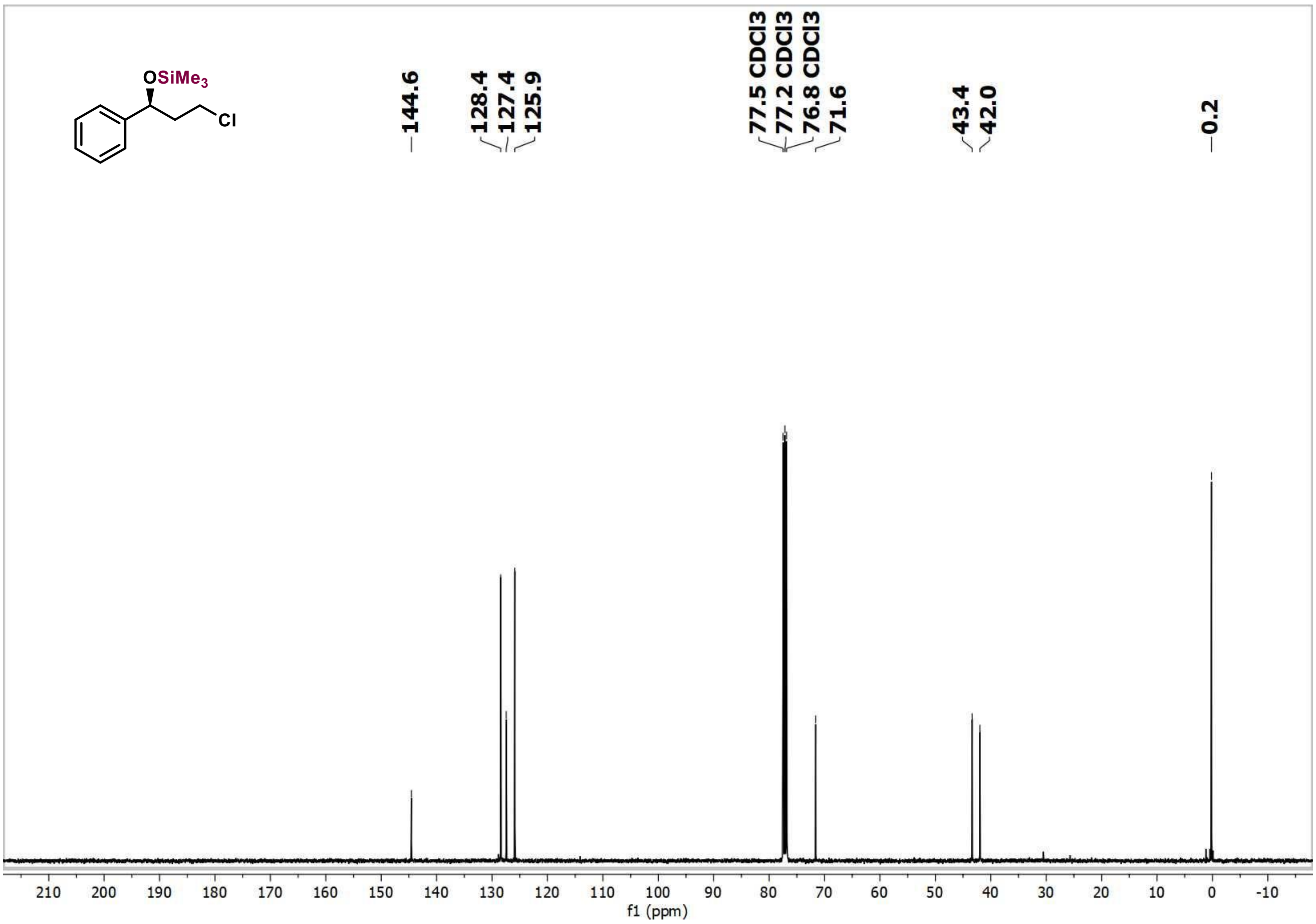

Figure S27. ^{13}C NMR (101 MHz, Chloroform-d, 25°C) of (*S*)-(3-chloro-1-phenylpropoxy)trimethylsilane (**3h**).

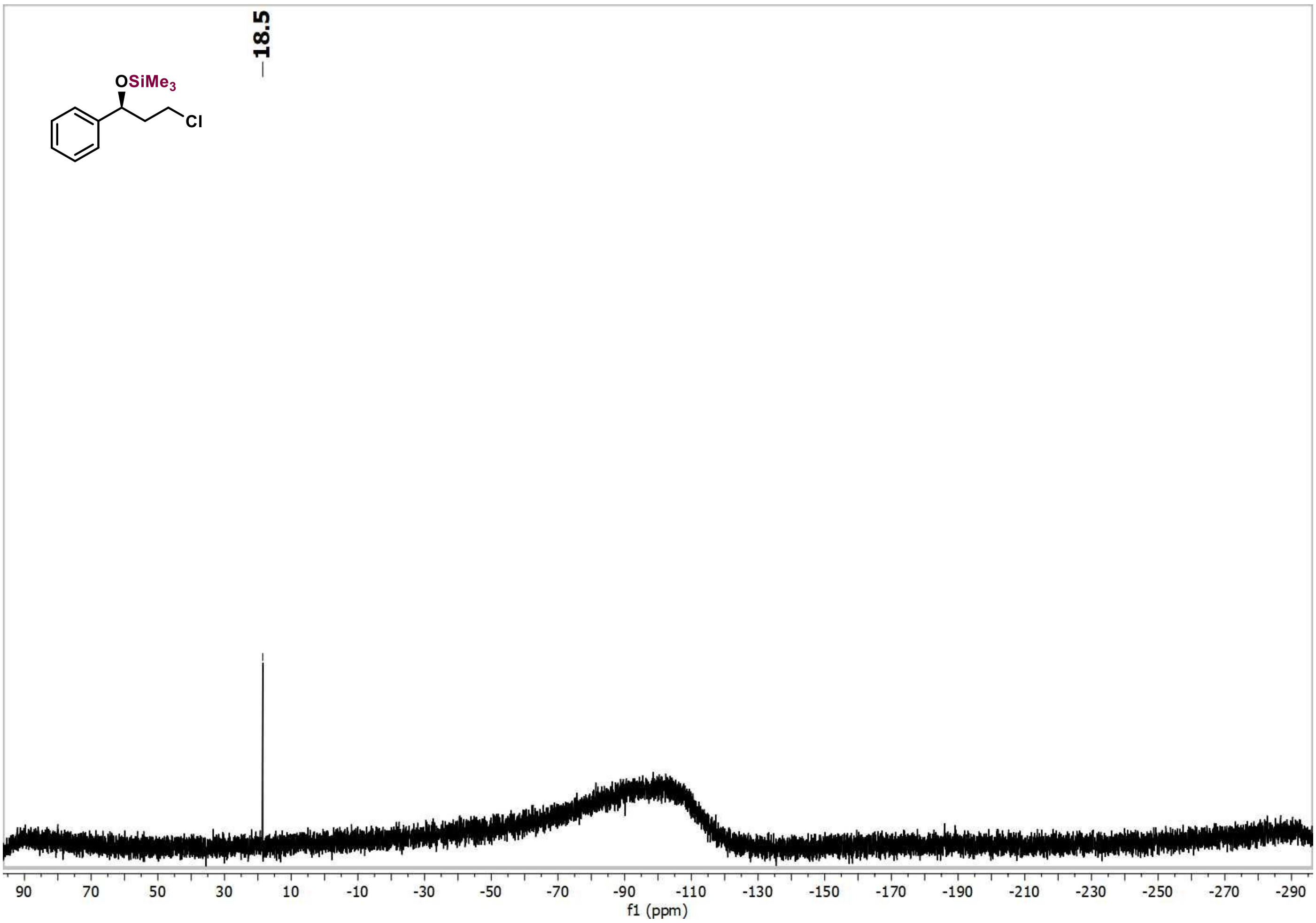

Figure S28. ^{29}Si NMR (79 MHz, Chloroform-d, 25°C) of (*S*)-(3-chloro-1-phenylpropoxy)trimethylsilane (**3h**).

((2,3-Dimethylbenzyl)oxy)trimethylsilane (**3i**)

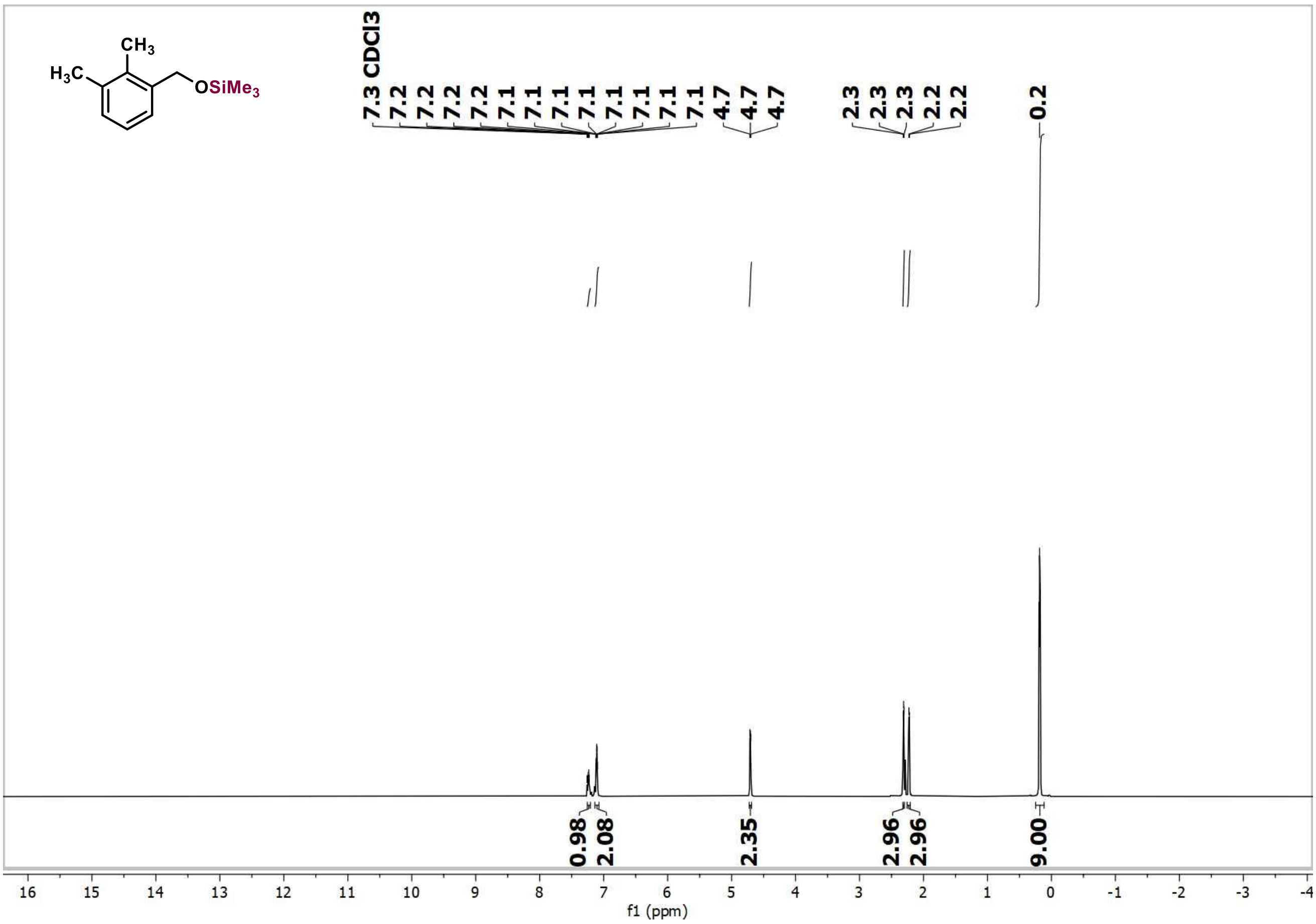

Figure S29. ^{1}H NMR (400 MHz, Chloroform-d, 25°C) of ((2,3-dimethylbenzyl)oxy)trimethylsilane (**3i**).

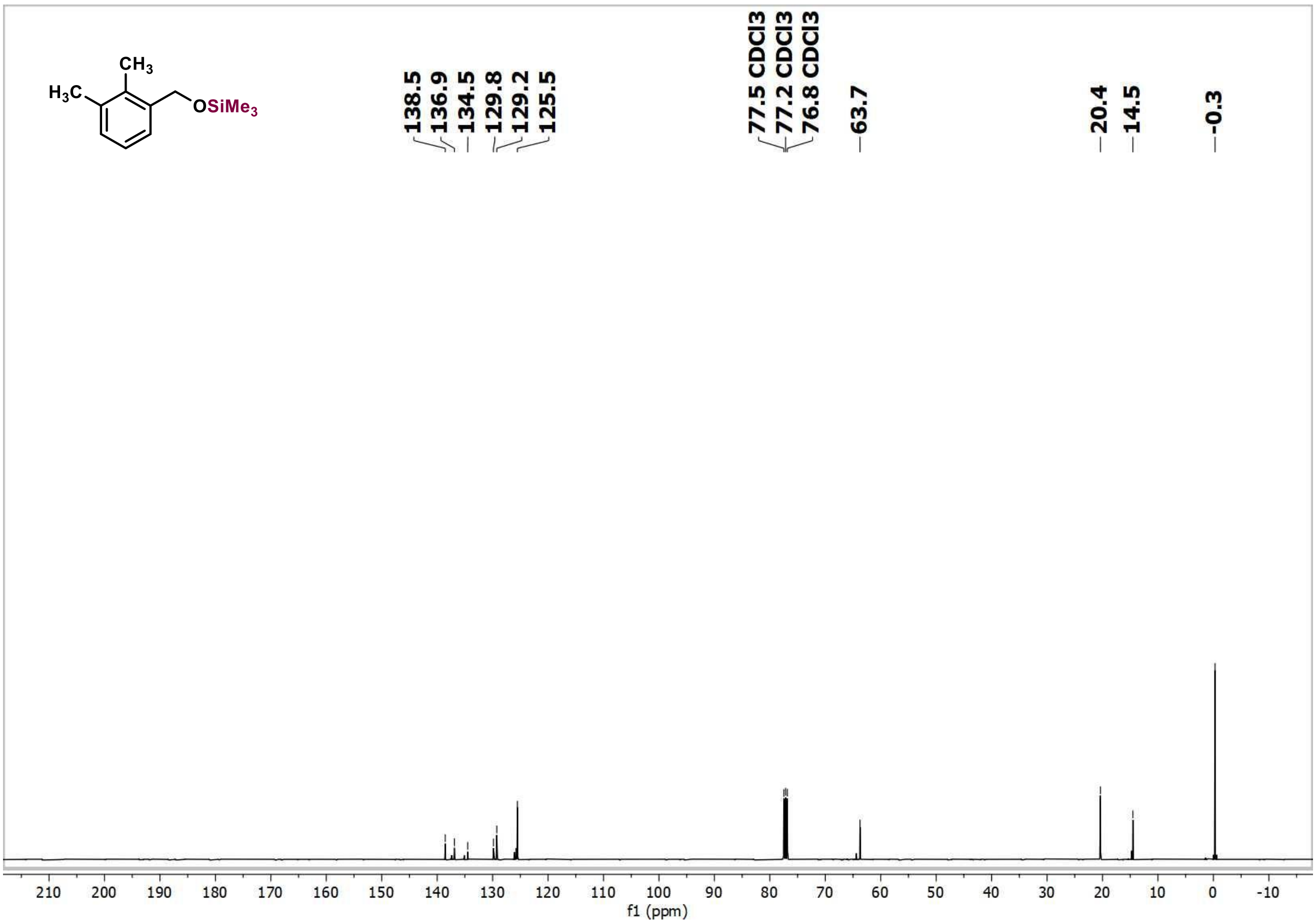

Figure S30. ^{13}C NMR (101 MHz, Chloroform-d, 25°C) of ((2,3-dimethylbenzyl)oxy)trimethylsilane (**3i**).

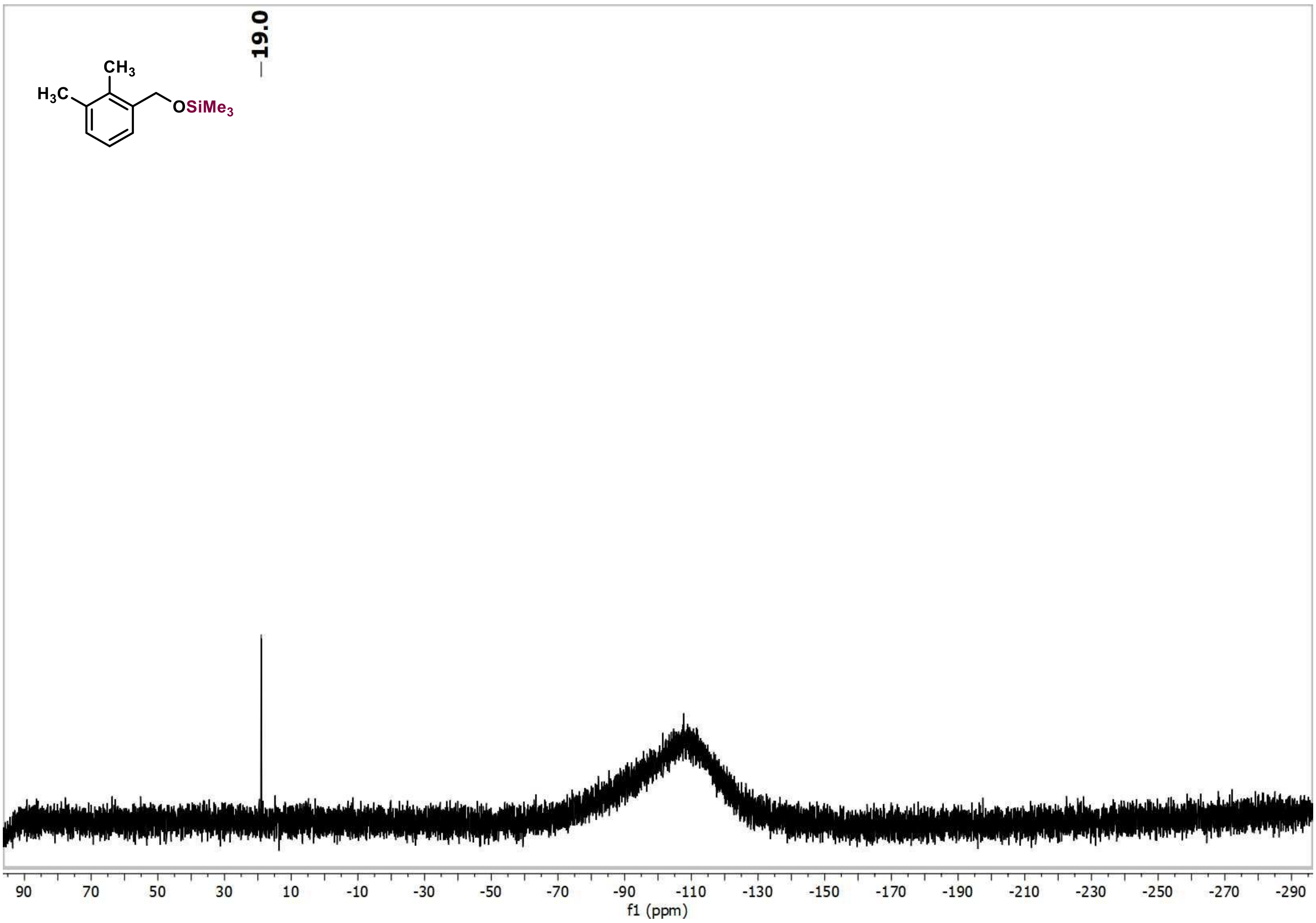

Figure S31. ^{29}Si NMR (79 MHz, Chloroform-d, 25°C) of ((2,3-dimethylbenzyl)oxy)trimethylsilane (**3i**).

Trimethyl(naphthalen-2-ylmethoxy)silane (**3j**)

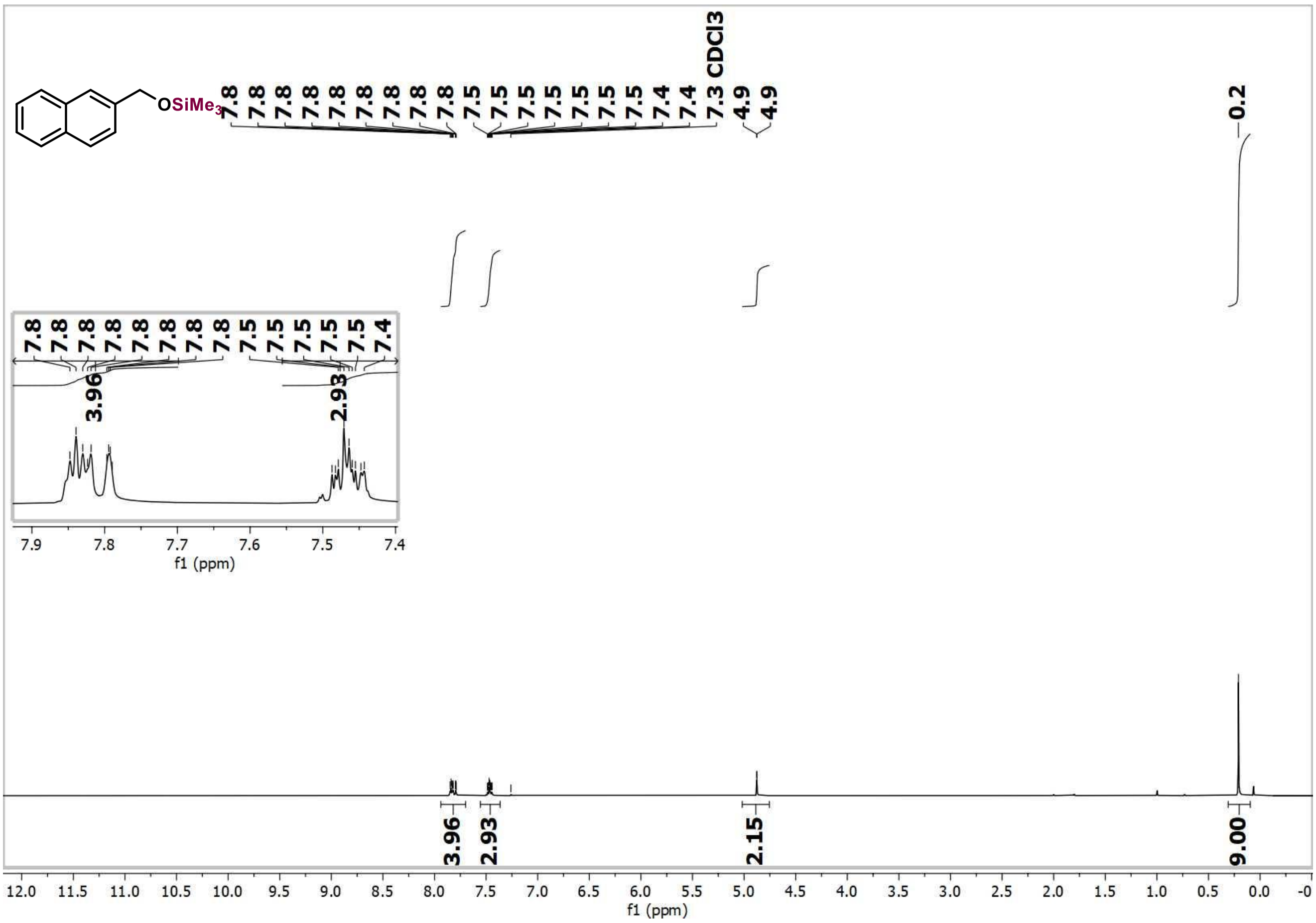

Figure S32. ^{1}H NMR (400 MHz, Chloroform-d, 25°C) of trimethyl(naphthalen-2-ylmethoxy)silane (**3j**).

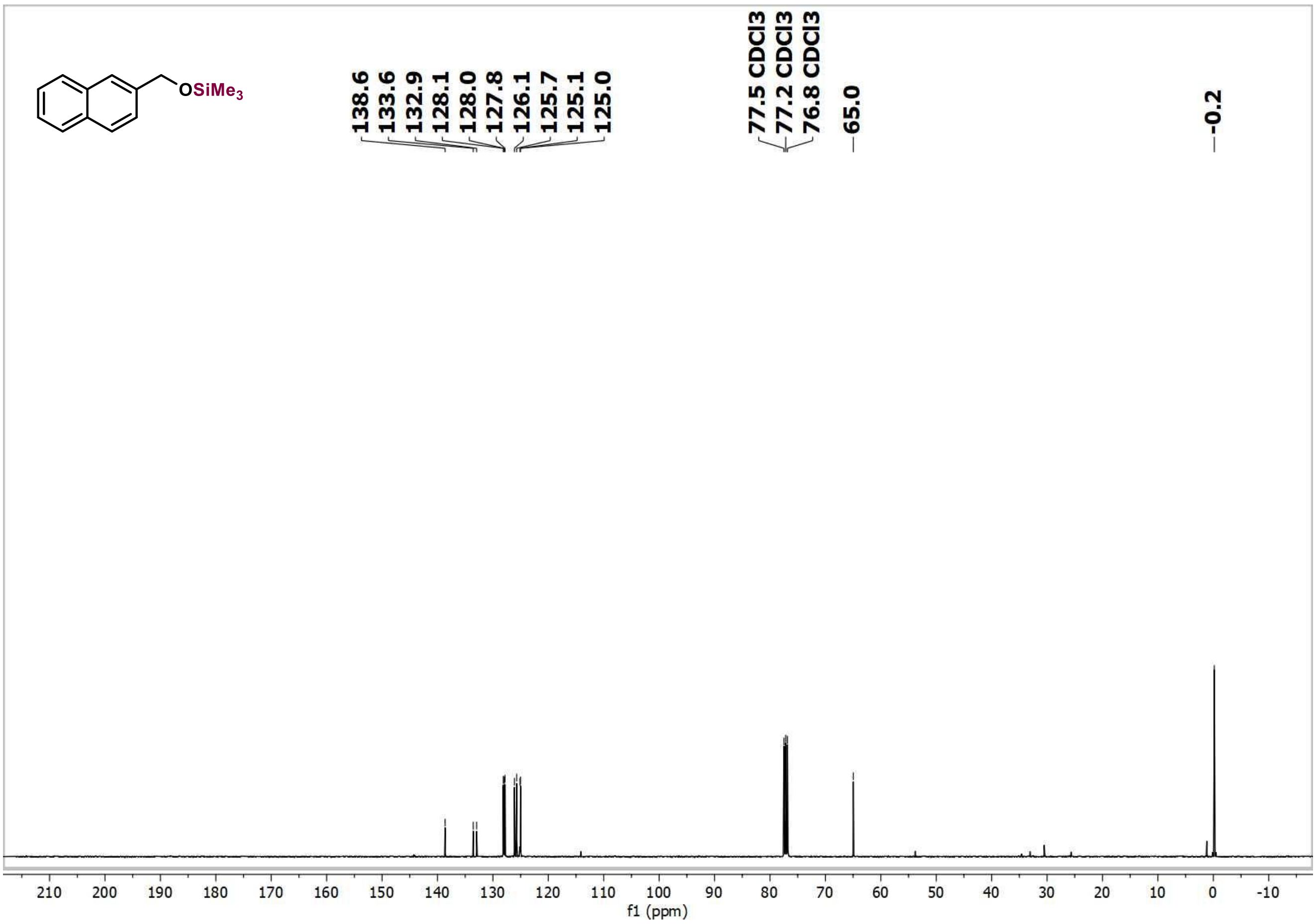

Figure S33. ^{13}C NMR (101 MHz, Chloroform-d, 25°C) of trimethyl(naphthalen-2-ylmethoxy)silane (**3j**).

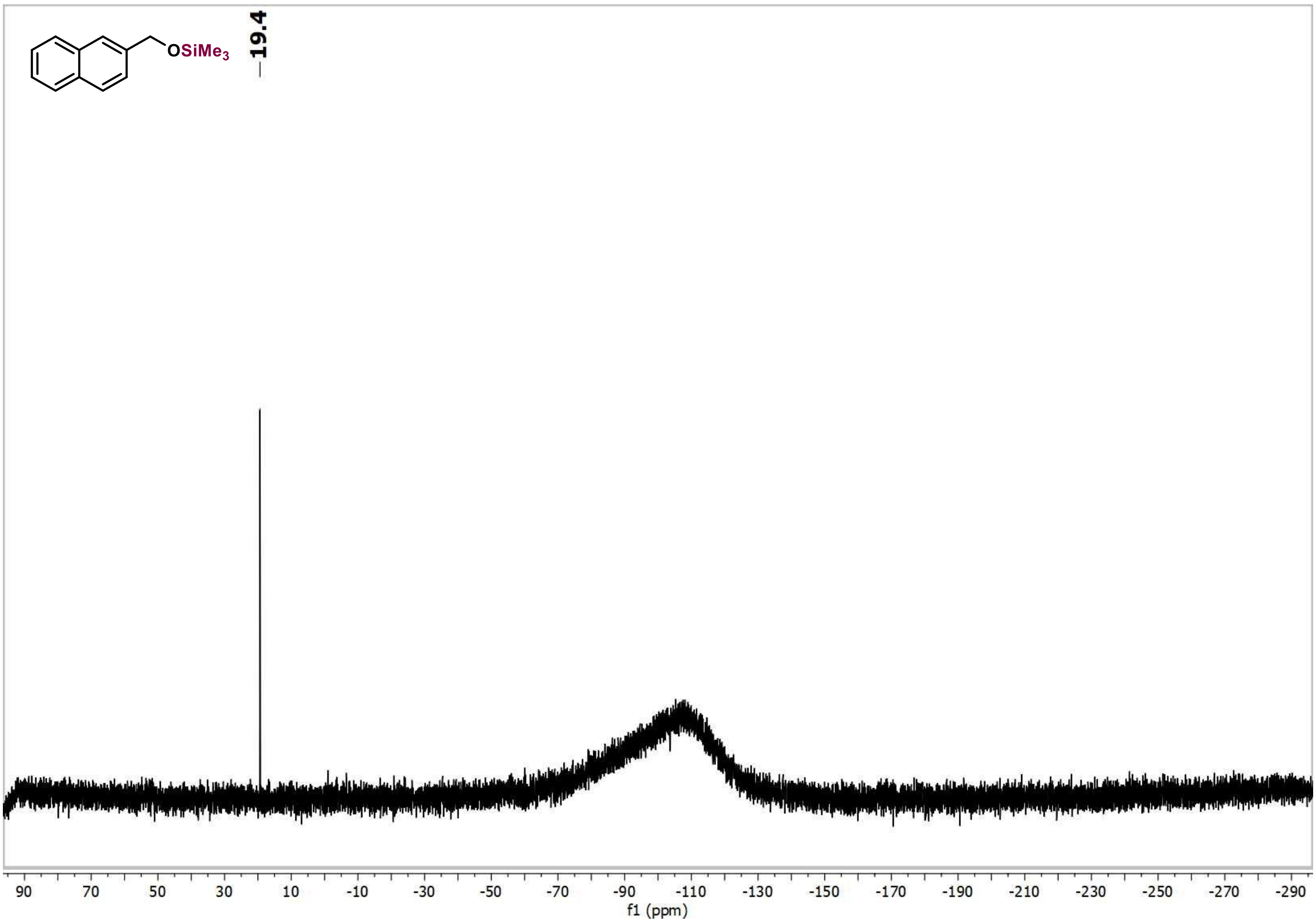

Figure S34. ^{29}Si NMR (79 MHz, Chloroform-d, 25°C) of trimethyl(naphthalen-2-ylmethoxy)silane (**3j**).

((4-Ethynylbenzyl)oxy)trimethylsilane (**3k**)

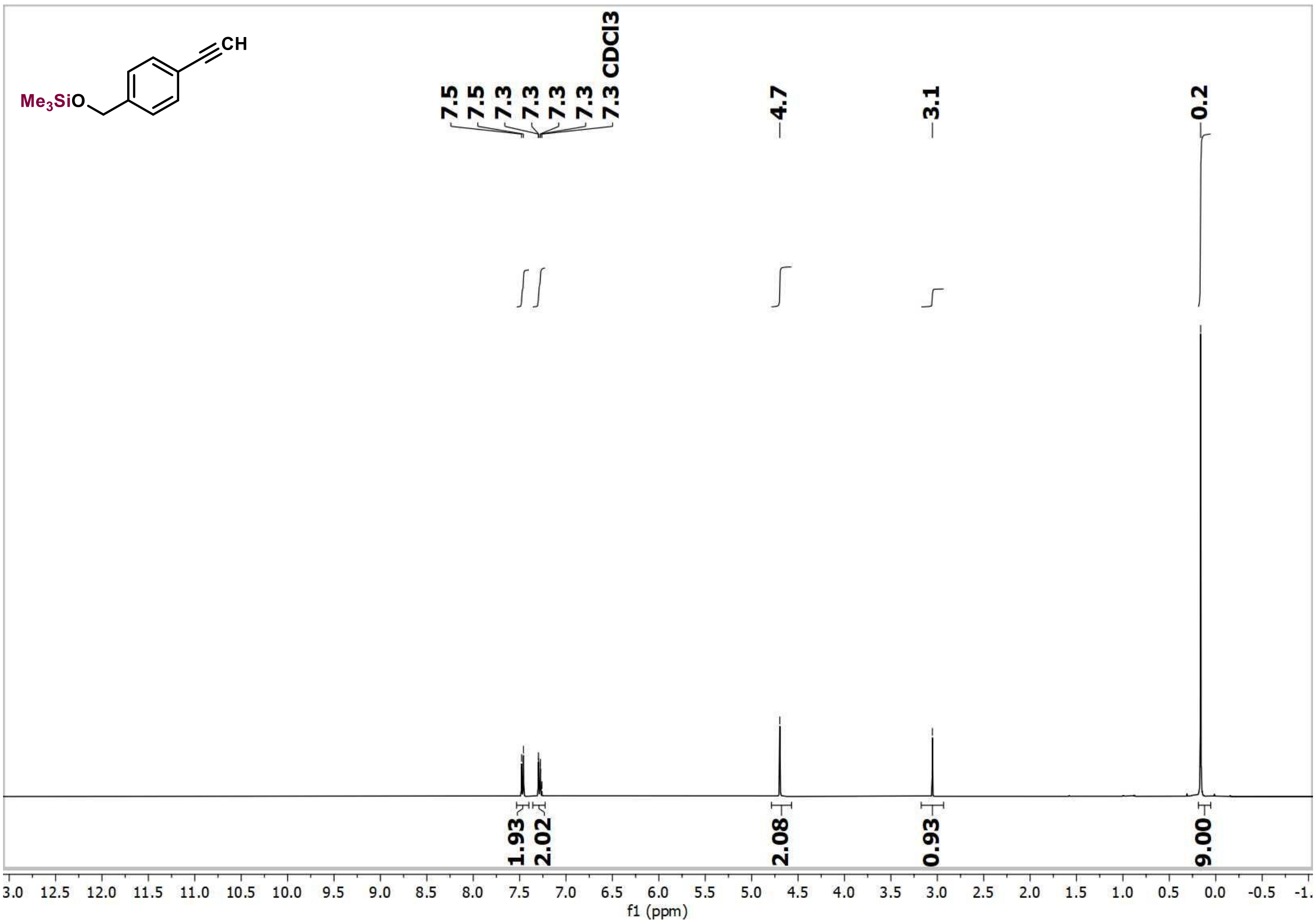

Figure S35. ^{1}H NMR (400 MHz, Chloroform-d, 25°C) of ((4-ethynylbenzyl)oxy)trimethylsilane (**3k**).

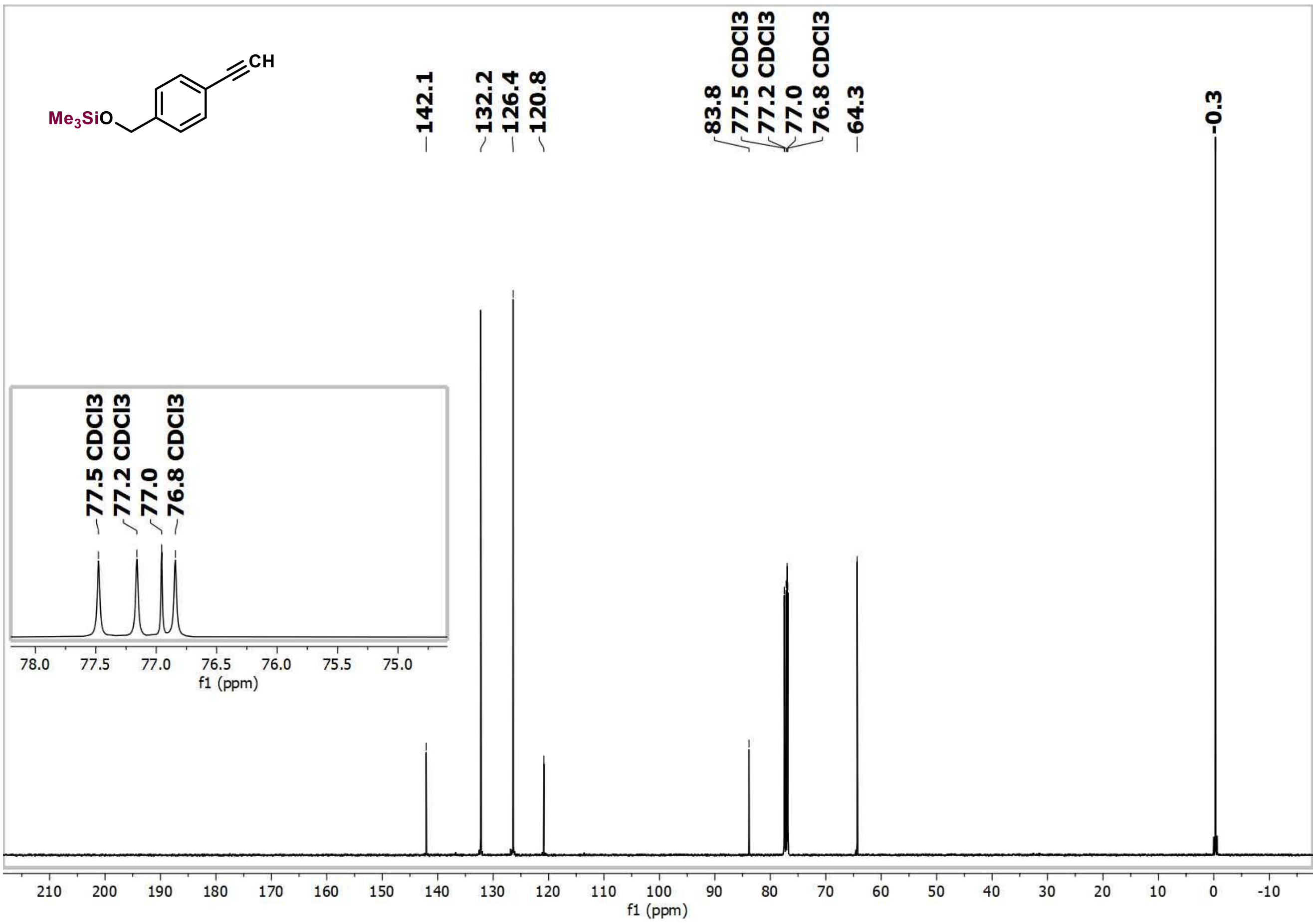

Figure S36. ^{13}C NMR (101 MHz, Chloroform-d, 25°C) of ((4-ethynylbenzyl)oxy)trimethylsilane (**3k**).

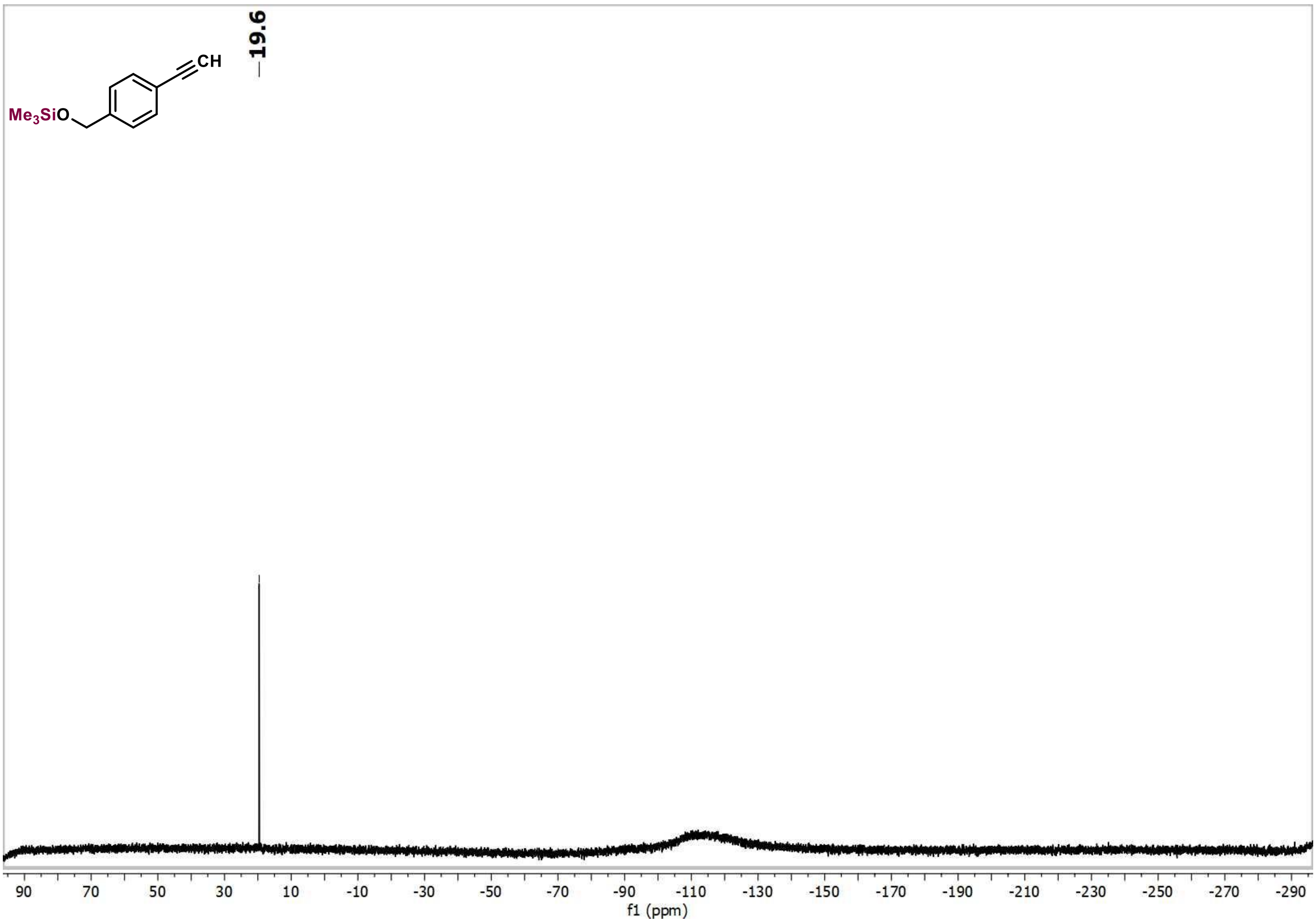

Figure S37. ^{29}Si NMR (79 MHz, Chloroform-d, 25°C) of ((4-ethynylbenzyl)oxy)trimethylsilane (**3k**).

Trimethyl((3-phenylprop-2-yn-1-yl)oxy)silane (**3l**)

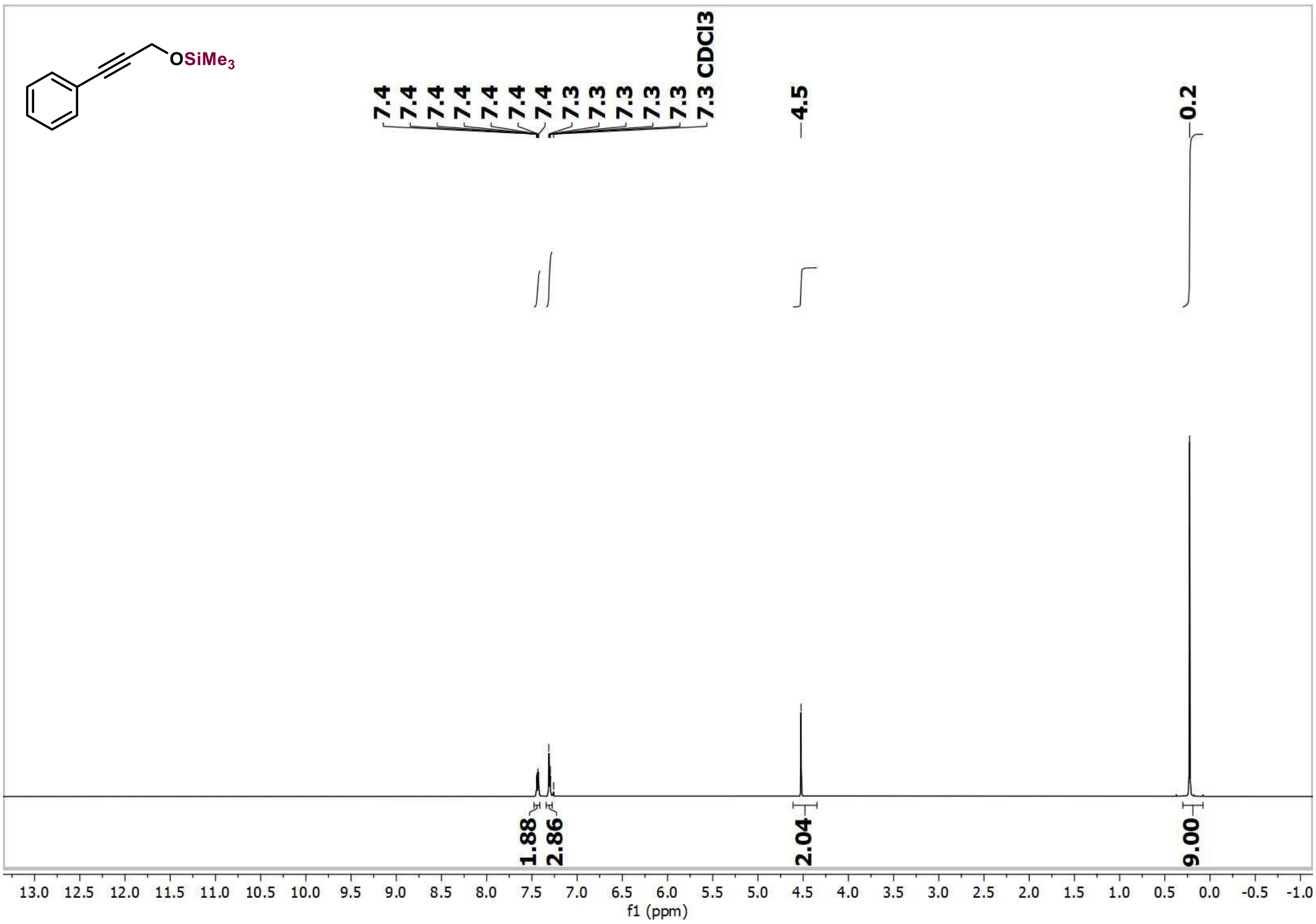

Figure S38. ^{1}H NMR (400 MHz, Chloroform-d, 25°C) of trimethyl((3-phenylprop-2-yn-1-yl)oxy)silane (**3l**).

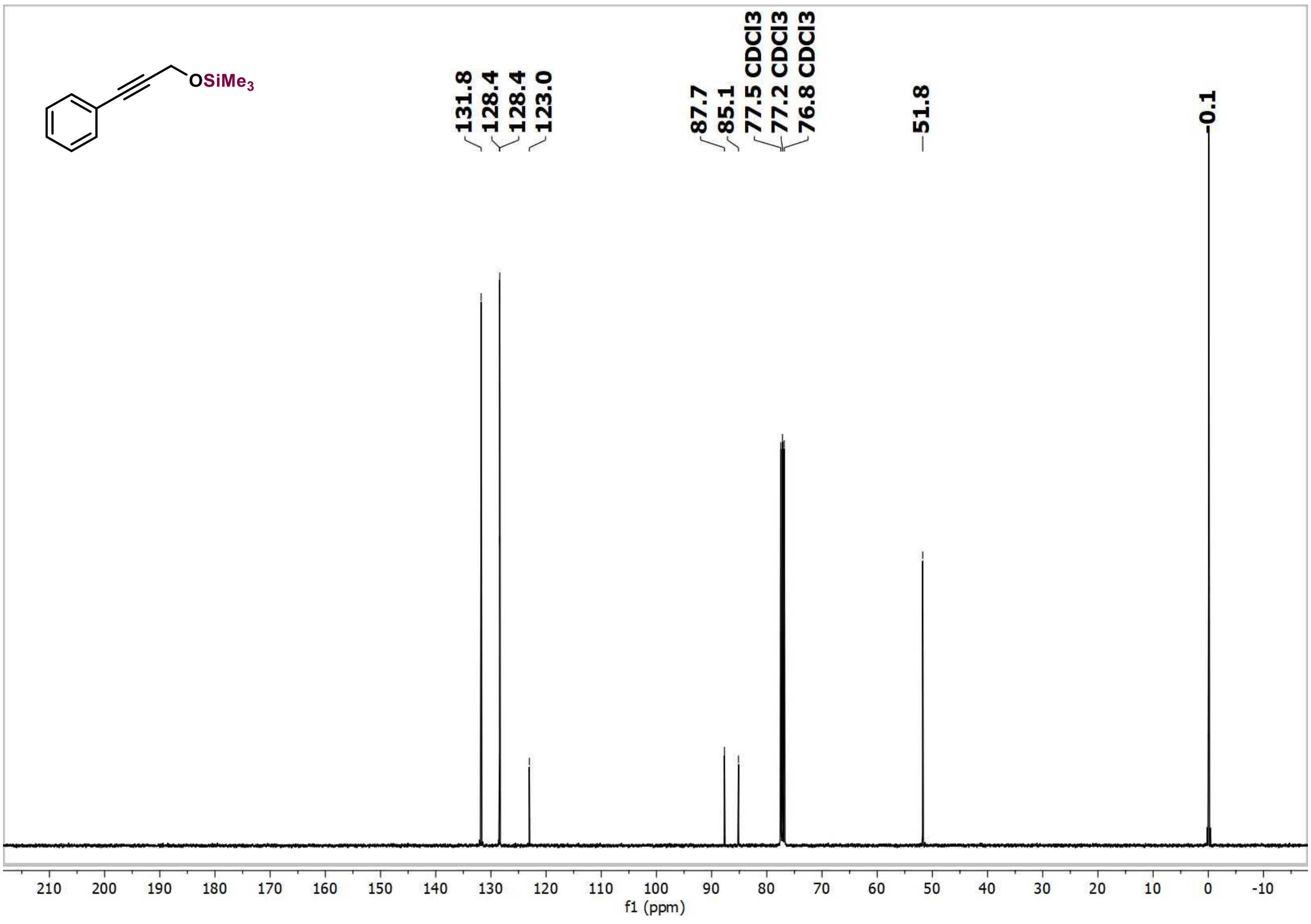

Figure S39. ^{13}C NMR (101 MHz, Chloroform-d, 25°C) of trimethyl((3-phenylprop-2-yn-1-yl)oxy)silane (**3l**).

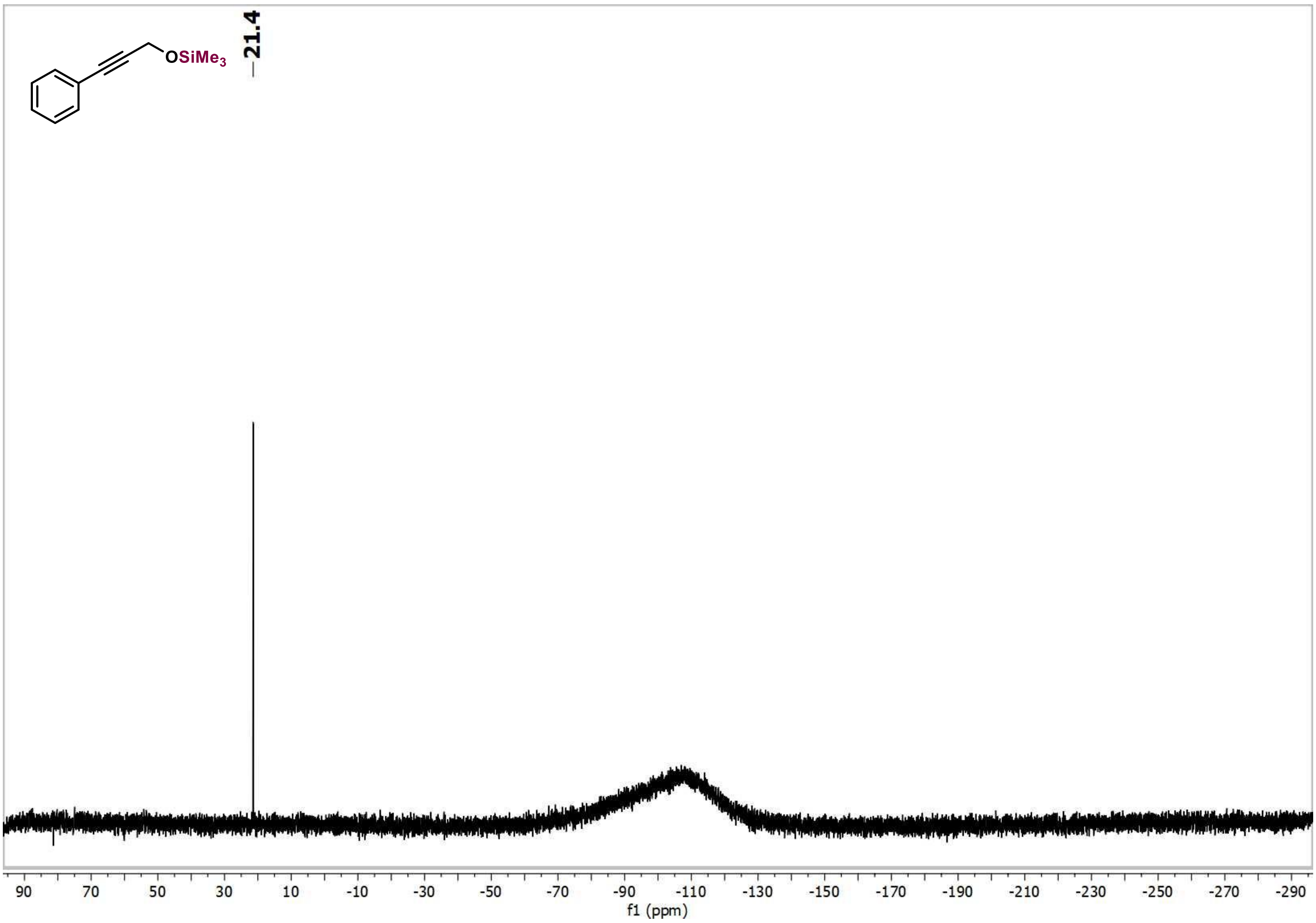

Figure S40. ^{29}Si NMR (79 MHz, Chloroform-d, 25°C) of trimethyl((3-phenylprop-2-yn-1-yl)oxy)silane (**3l**).

Trimethyl(oct-1-yn-3-yloxy)silane (**3m**)

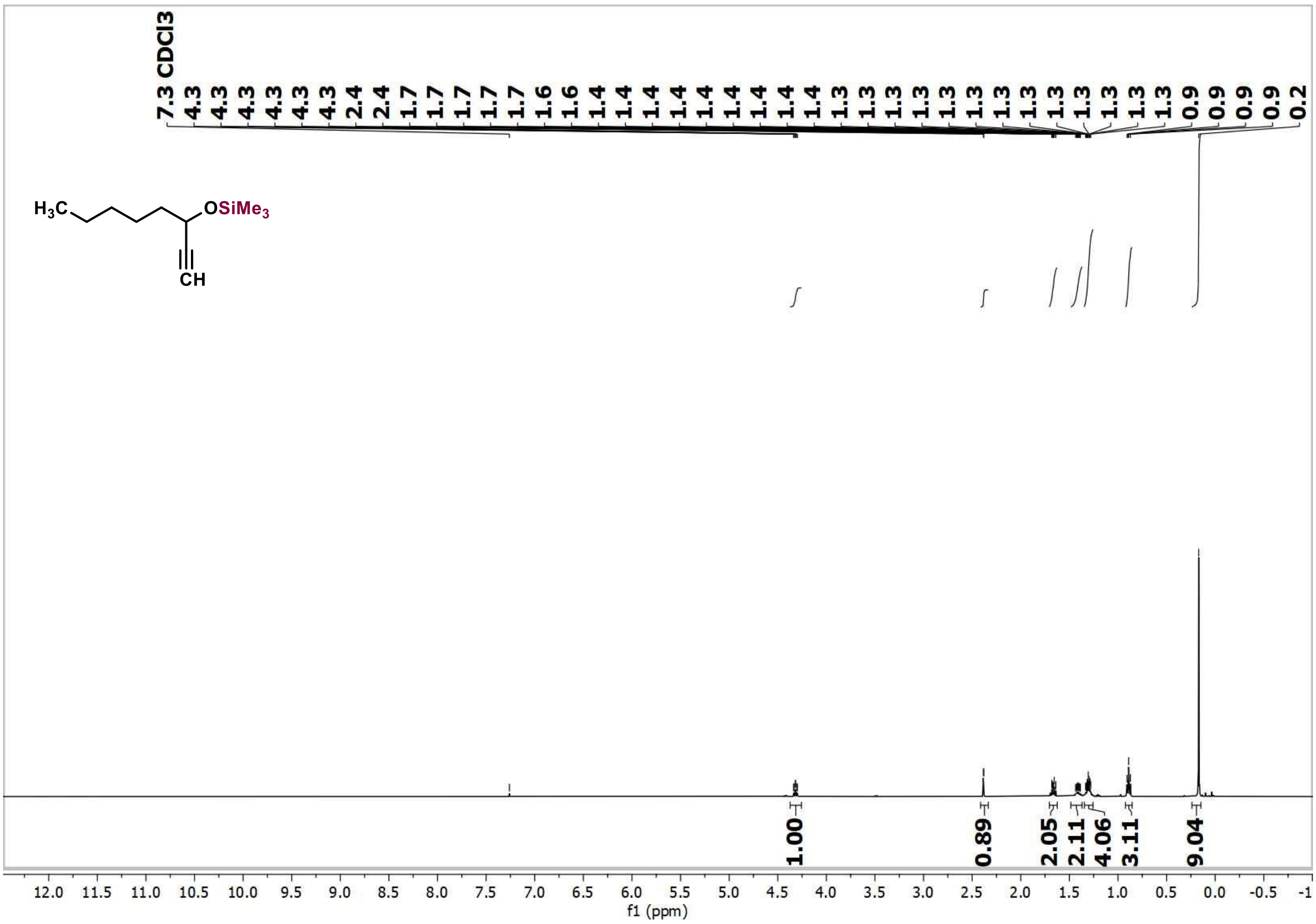

Figure S41. ^{1}H NMR (400 MHz, Chloroform-d, 25°C) of trimethyl(oct-1-yn-3-yloxy)silane (**3m**).

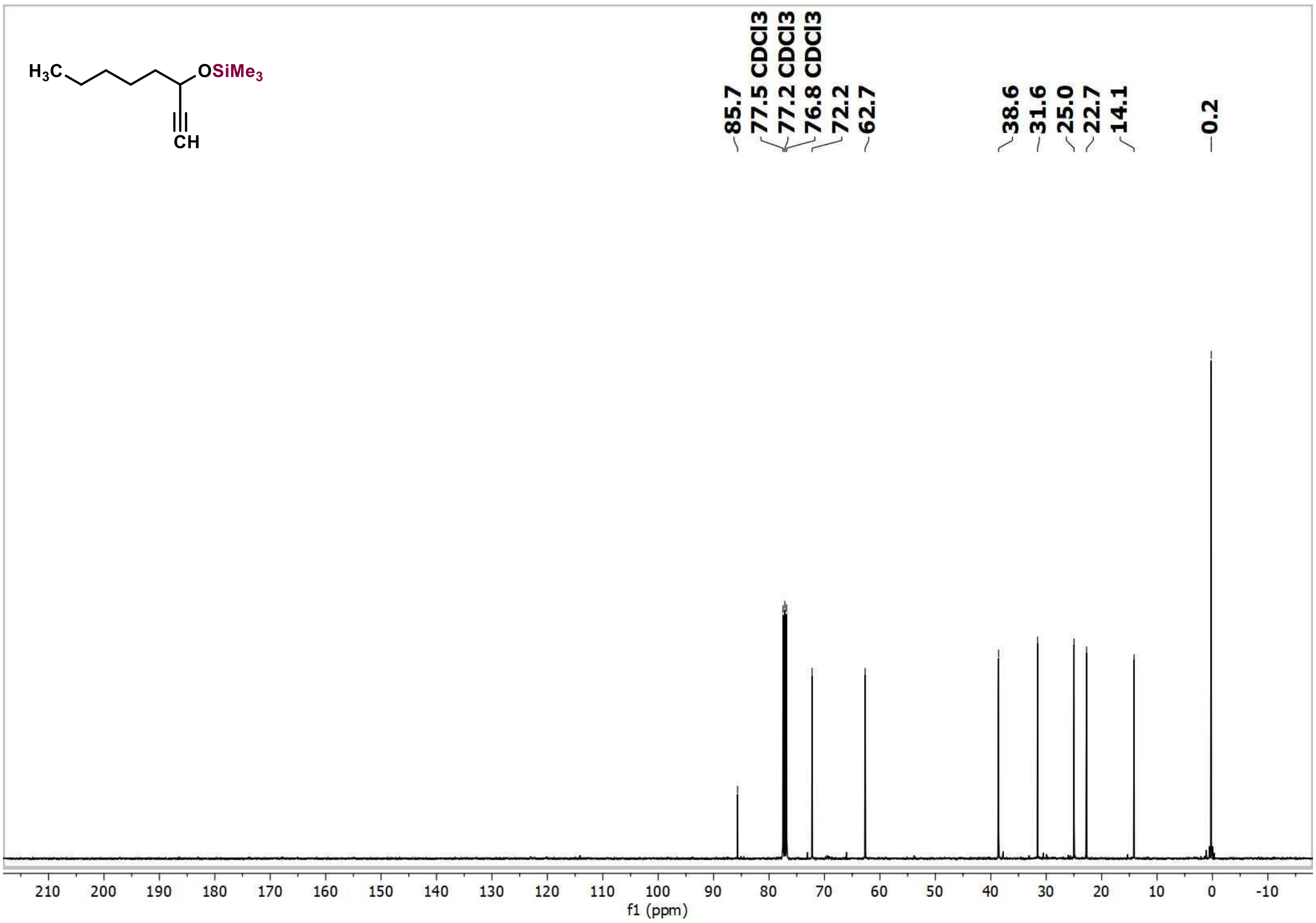

Figure S42. ^{13}C NMR (101 MHz, Chloroform-d, 25°C) of trimethyl(oct-1-yn-3-yloxy)silane (**3m**).

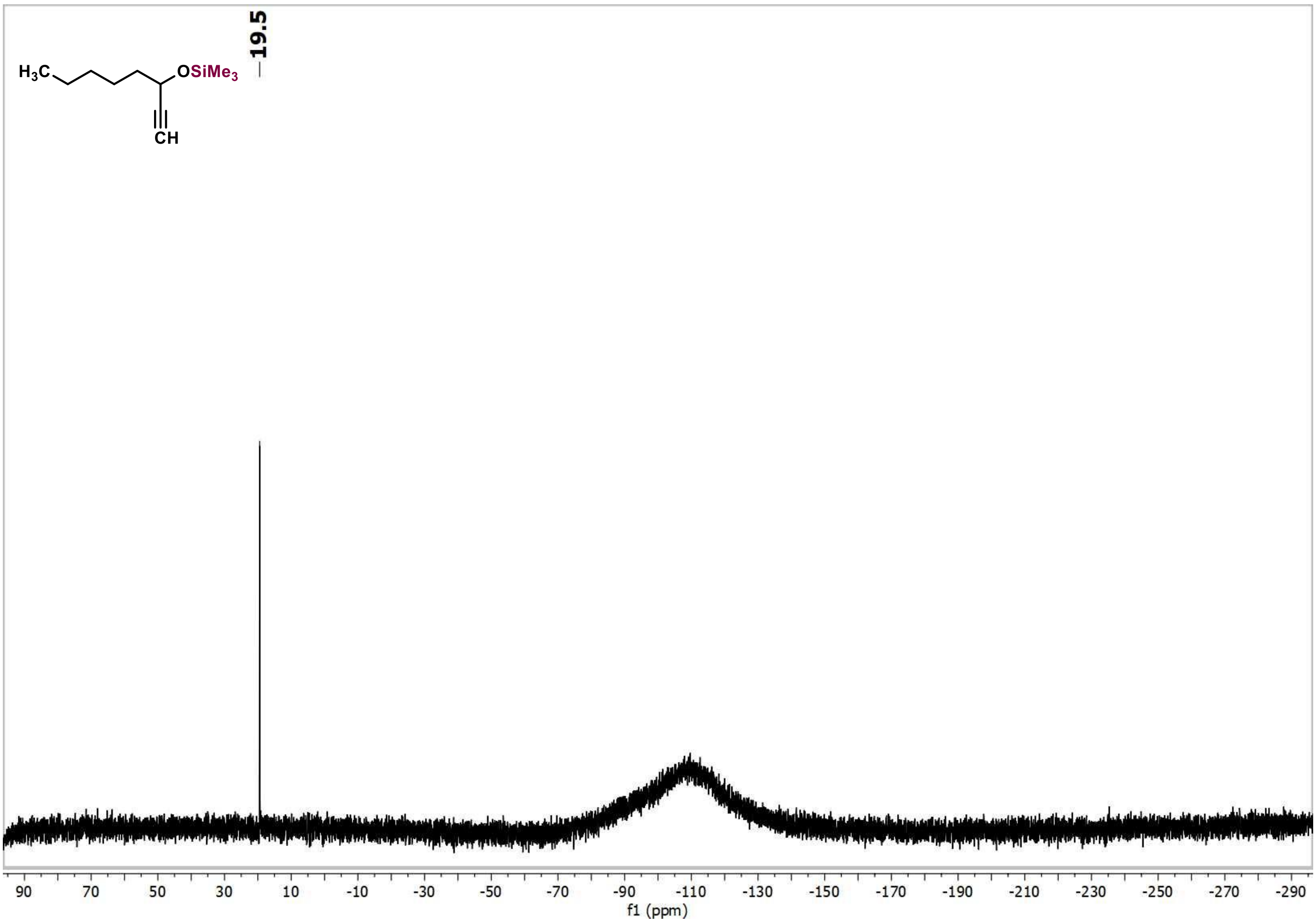

Figure S43. ^{29}Si NMR (79 MHz, Chloroform-d, 25°C) of trimethyl(oct-1-yn-3-yloxy)silane (**3m**).

(Dec-9-en-1-yloxy)trimethylsilane (**3n**)

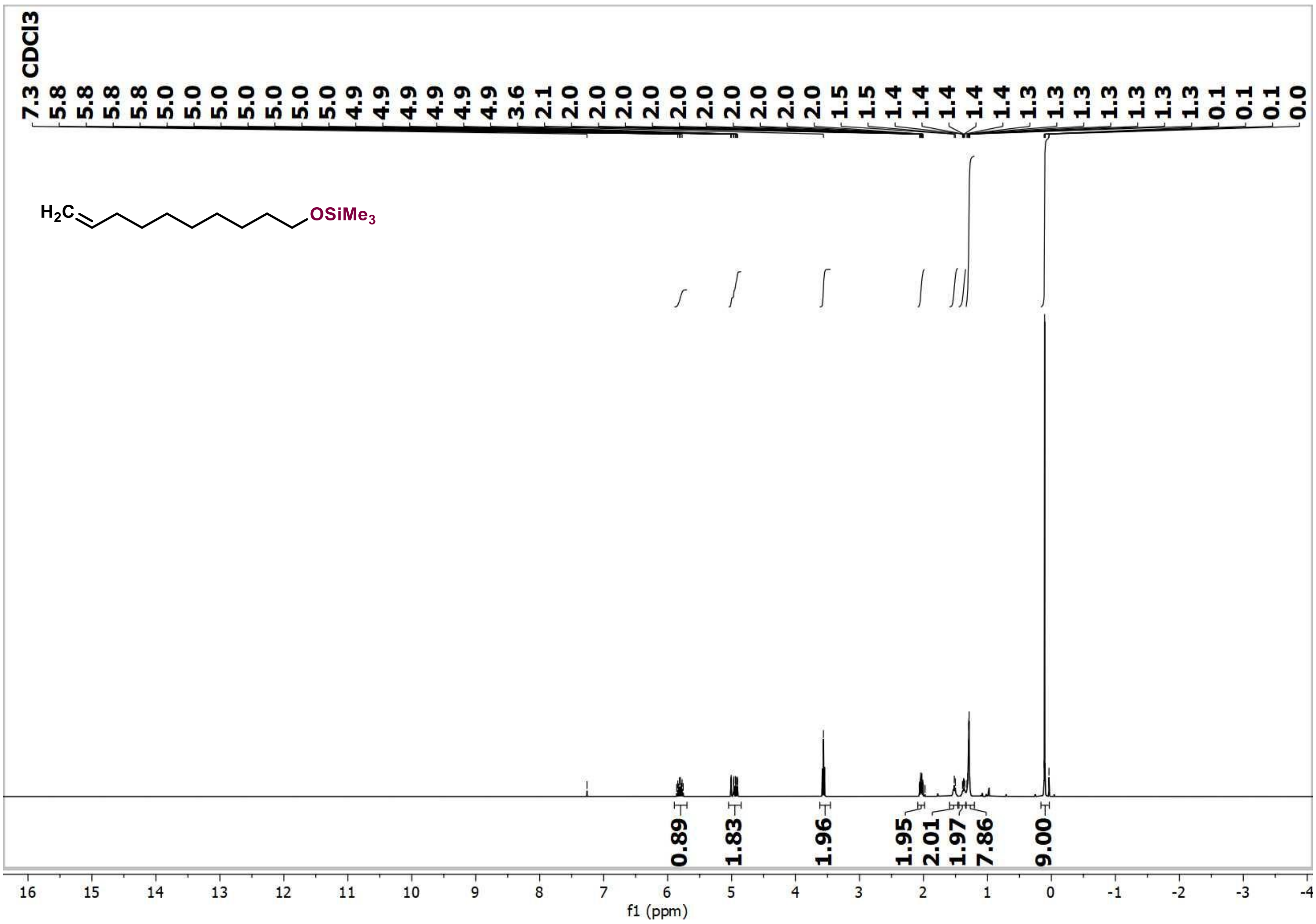

Figure S44. ^{1}H NMR (400 MHz, Chloroform-d, 25°C) of (dec-9-en-1-yloxy)trimethylsilane (**3n**).

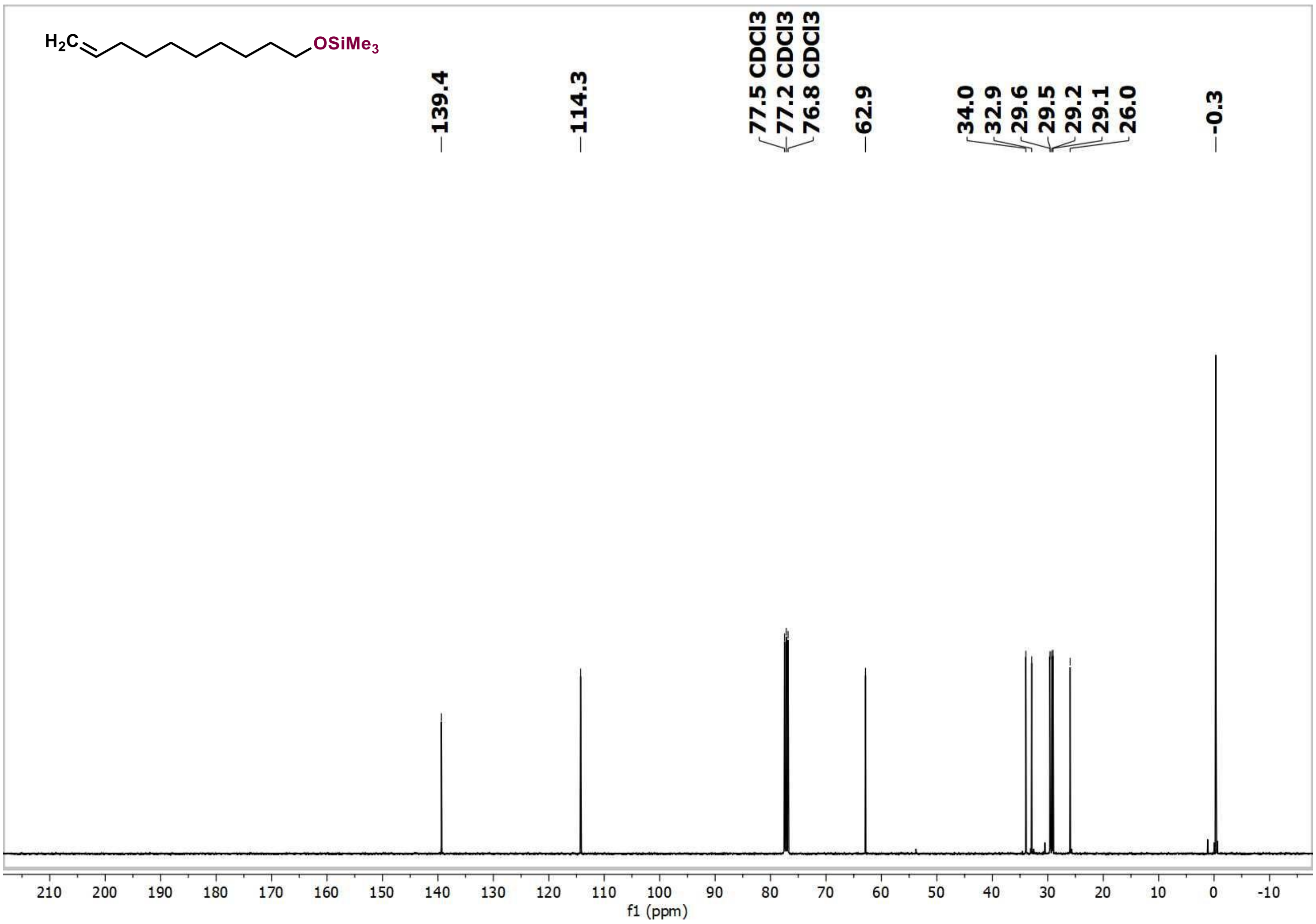

Figure S45. ^{13}C NMR (101 MHz, Chloroform-d, 25°C) of (dec-9-en-1-yloxy)trimethylsilane (**3n**).

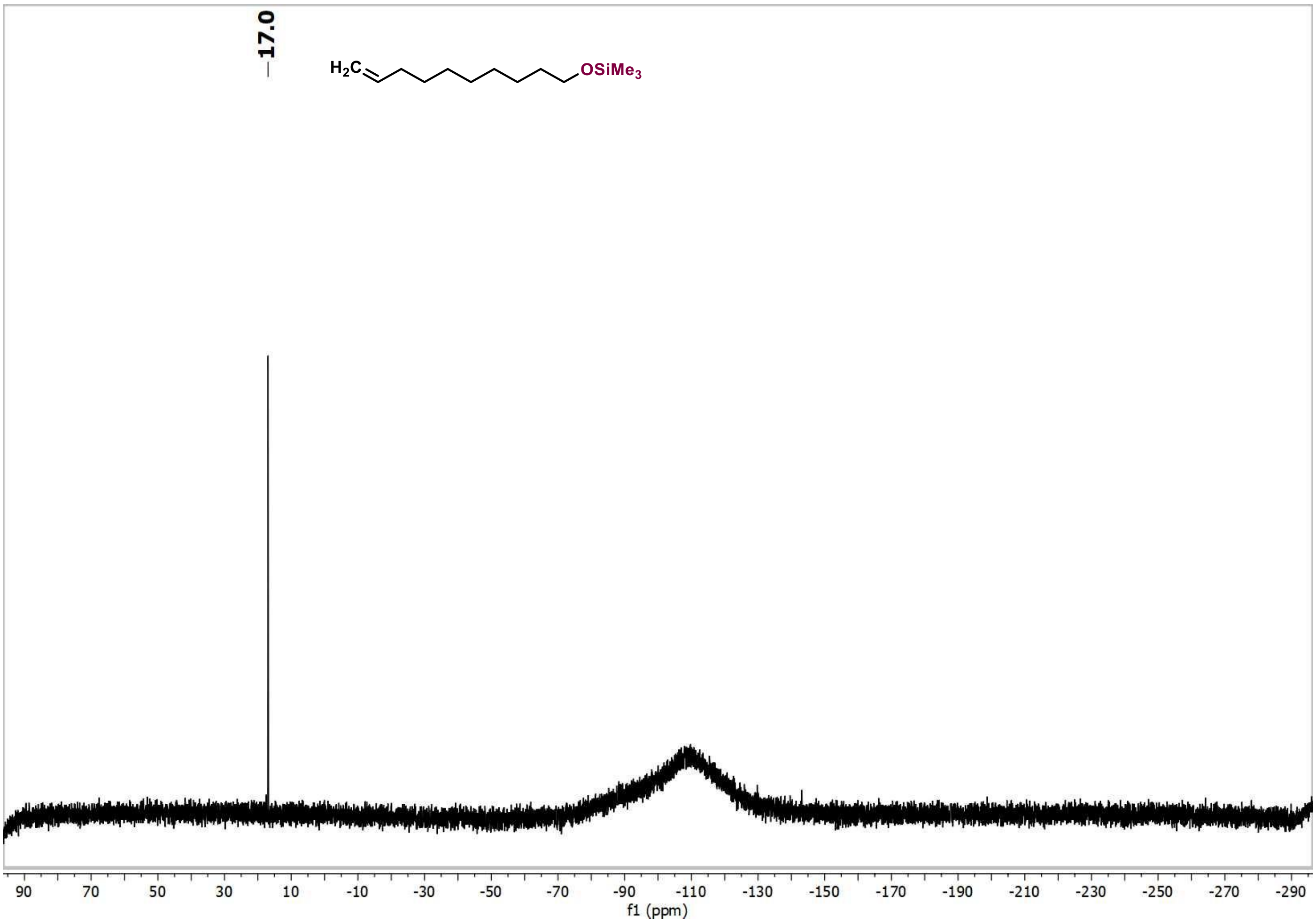

Figure S46. ^{29}Si NMR (79 MHz, Chloroform-d, 25°C) of (dec-9-en-1-yloxy)trimethylsilane (**3n**).

Trimethyl(undec-10-en-1-yloxy)silane (**3o**)

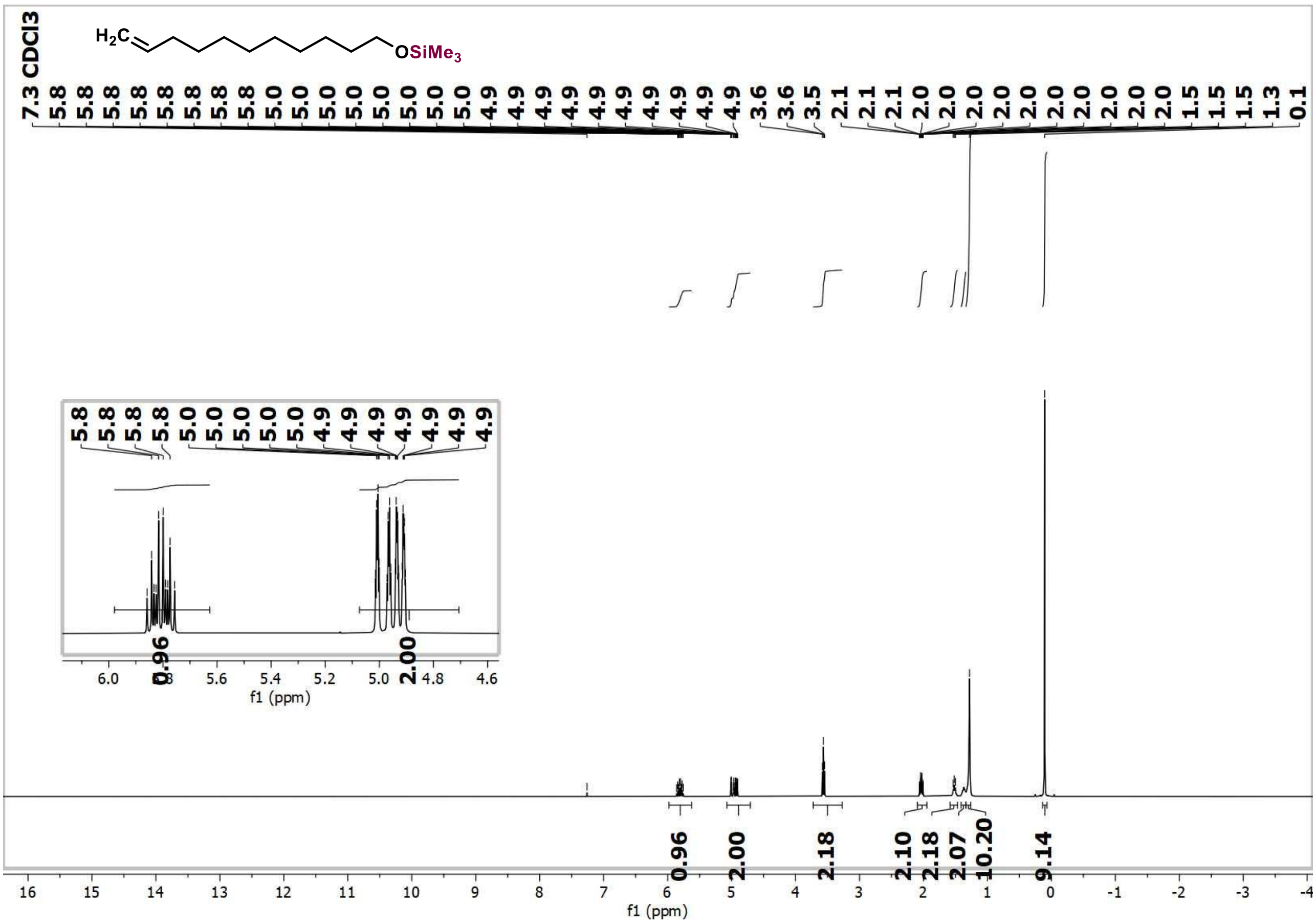

Figure S47. ^{1}H NMR (400 MHz, Chloroform-d, 25°C) of trimethyl(undec-10-en-1-yloxy)silane (**3o**).

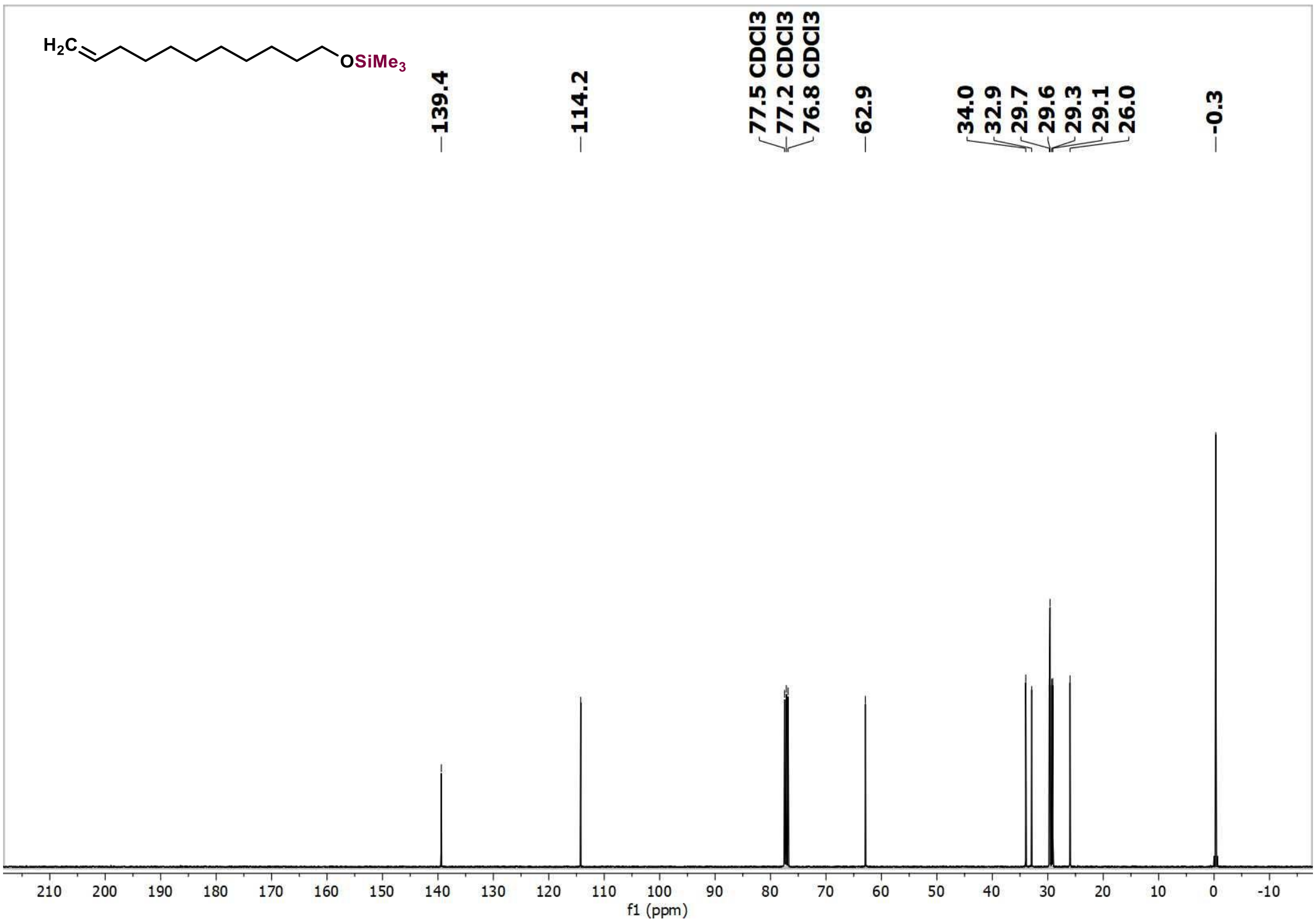

Figure S48. ^{13}C NMR (101 MHz, Chloroform-d, 25°C) of trimethyl(undec-10-en-1-yloxy)silane (**3o**).

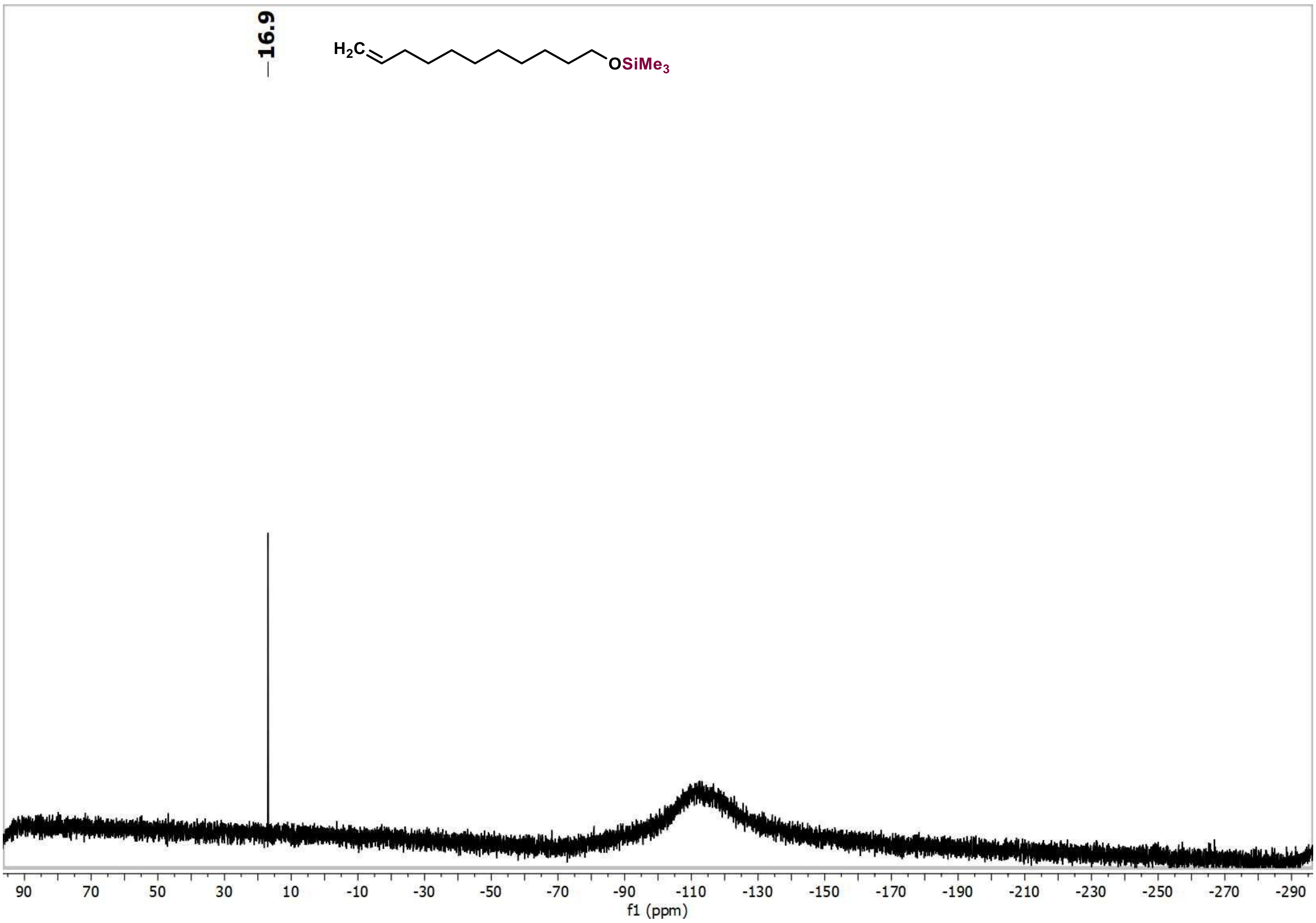

Figure S49. ^{29}Si NMR (79 MHz, Chloroform-d, 25°C) of trimethyl(undec-10-en-1-yloxy)silane (**3o**).

(Cinnamyloxy)trimethylsilane (**3p**)

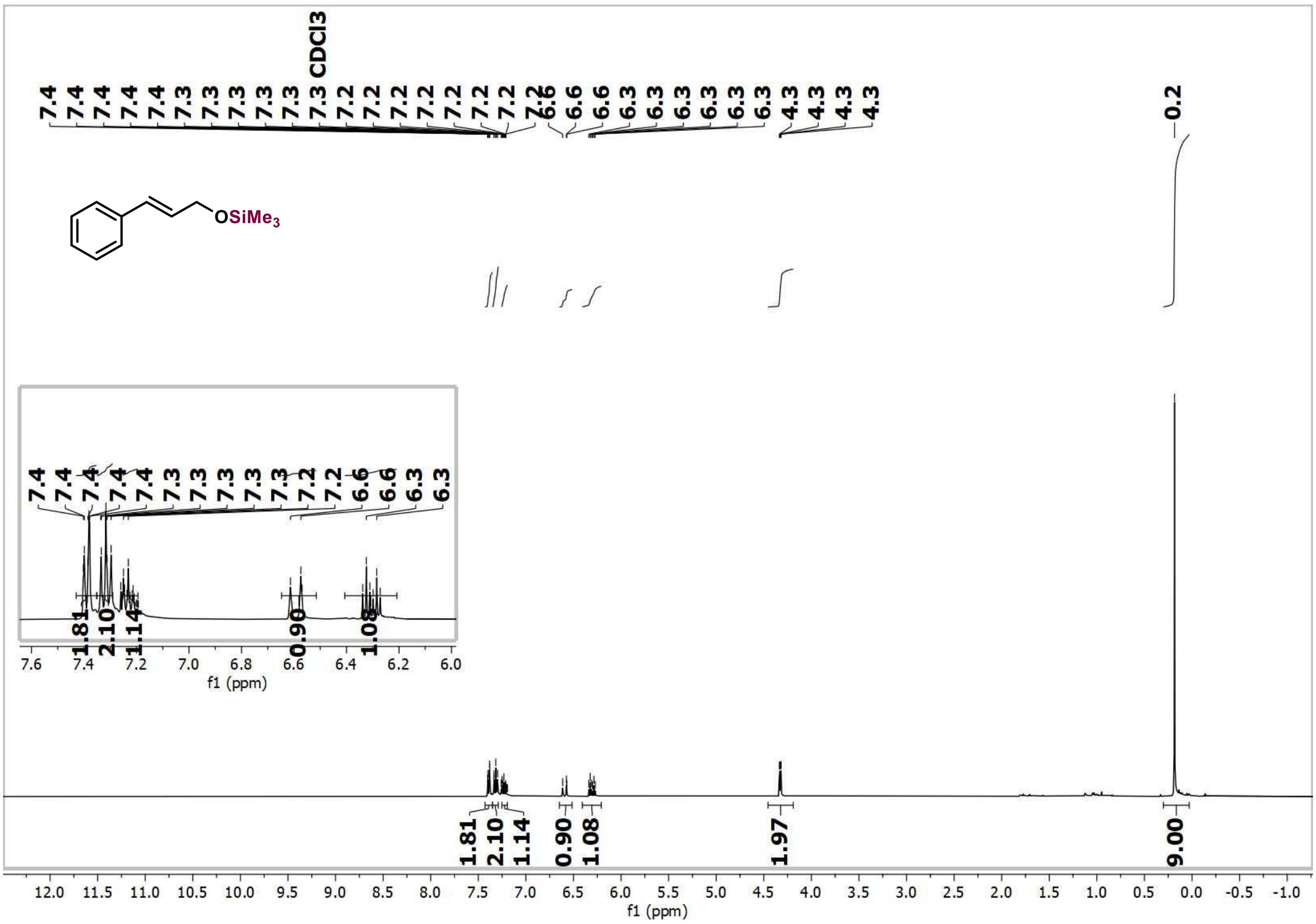

Figure S50. ^{1}H NMR (400 MHz, Chloroform-d, 25°C) of (cinnamyloxy)trimethylsilane (**3p**).

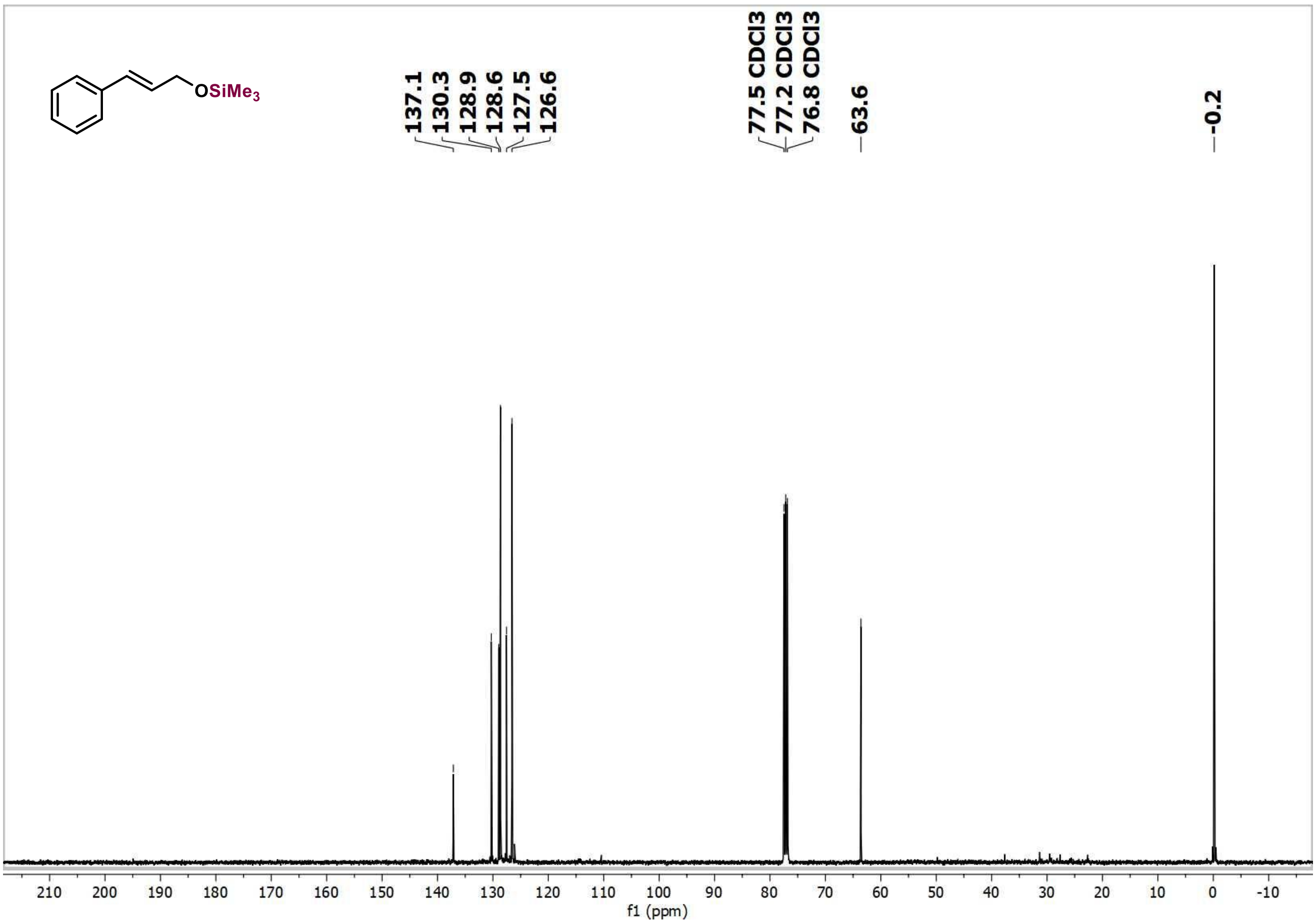

Figure S51. ^{13}C NMR (101 MHz, Chloroform-d, 25°C) of (cinnamyloxy)trimethylsilane (**3p**).

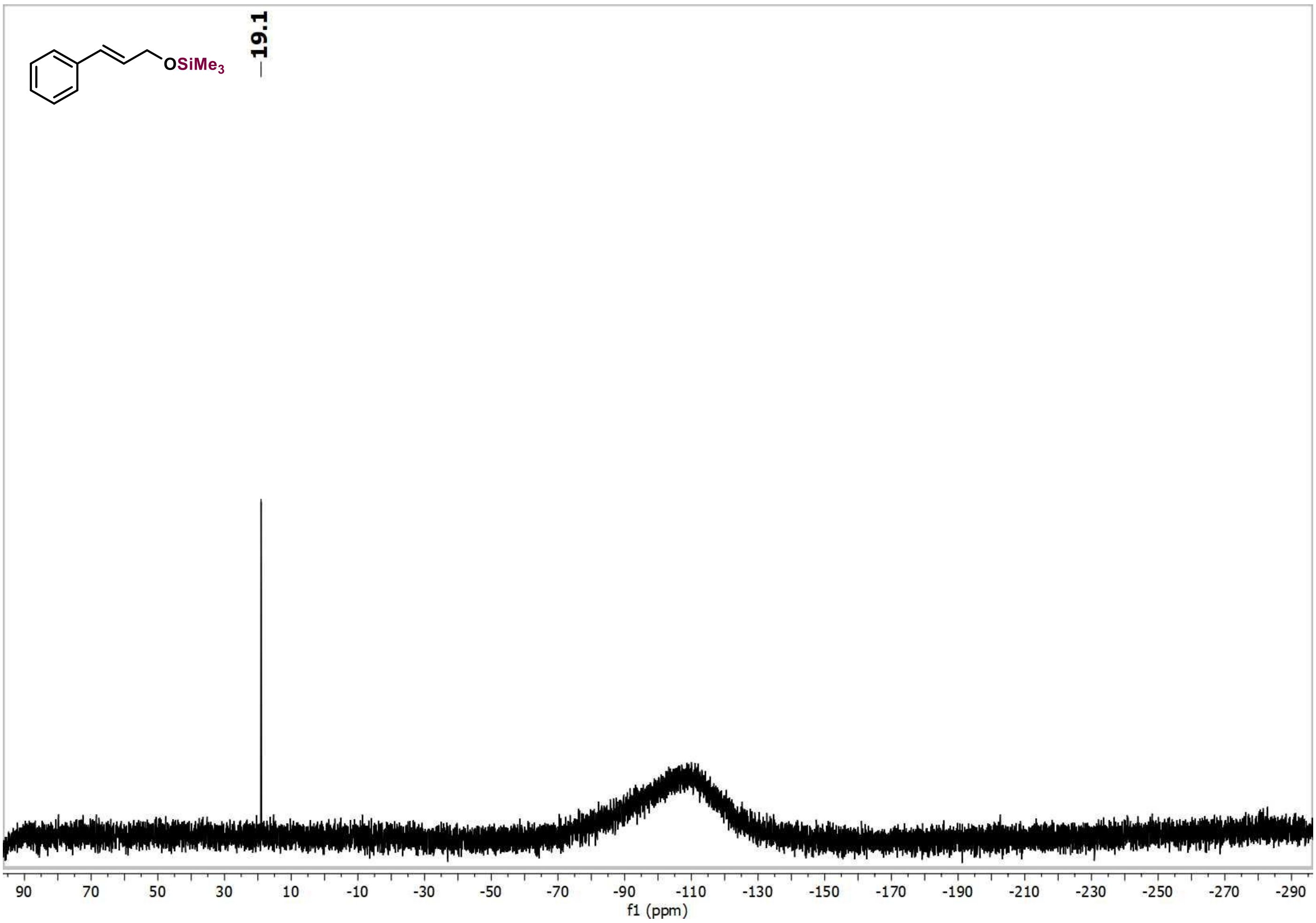

Figure S52. ^{29}Si NMR (79 MHz, Chloroform-d, 25°C) of (cinnamyloxy)trimethylsilane (**3p**).

(*E*)-((3,7-dimethylocta-2,6-dien-1-yl)oxy)trimethylsilane (**3q**)

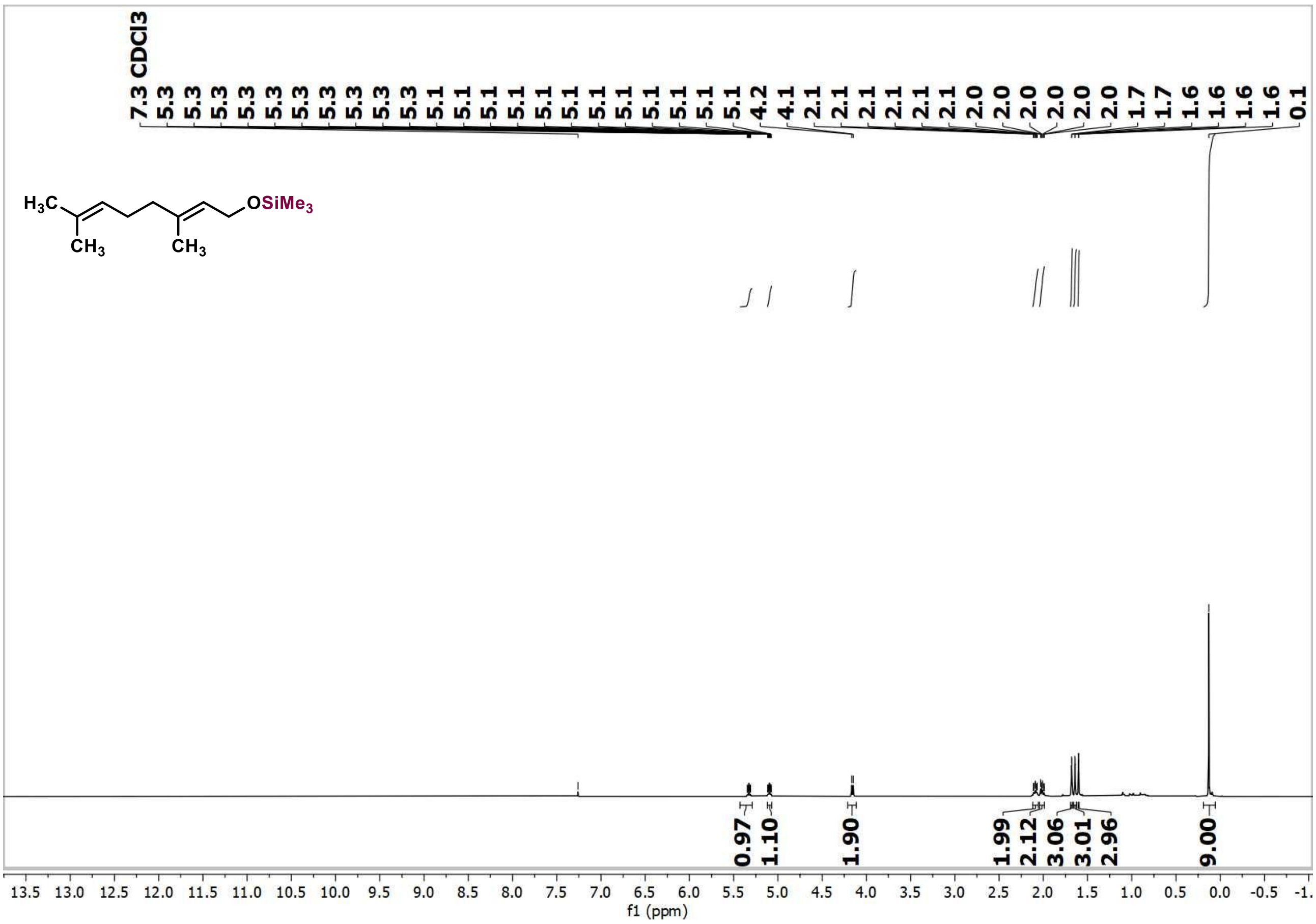

Figure S53. ^{1}H NMR (400 MHz, Chloroform-d, 25°C) of (*E*)-((3,7-dimethylocta-2,6-dien-1-yl)oxy)trimethylsilane (**3q**).

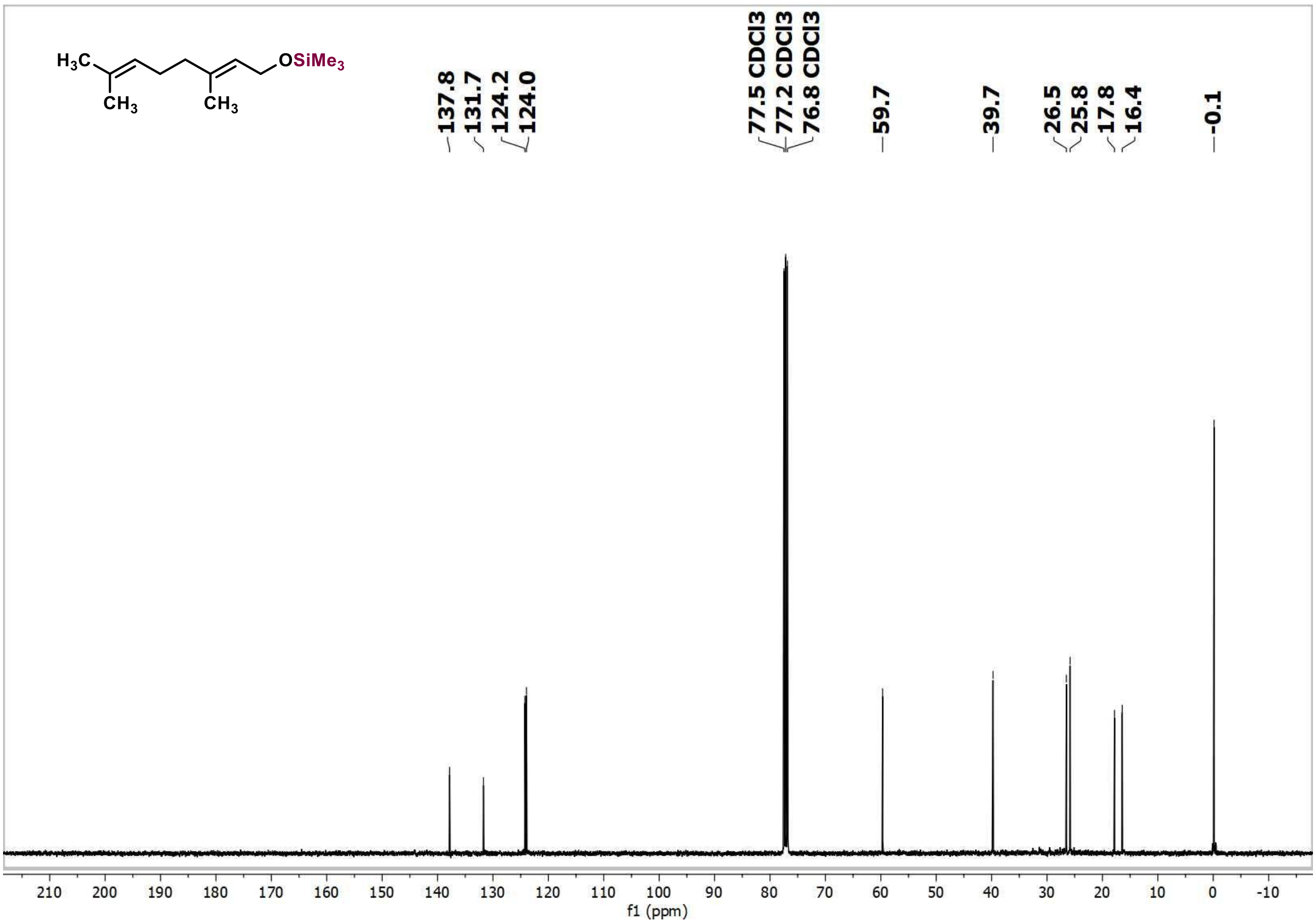

Figure S54. ^{13}C NMR (101 MHz, Chloroform-d, 25°C) of (*E*)-((3,7-dimethylocta-2,6-dien-1-yl)oxy)trimethylsilane (**3q**).

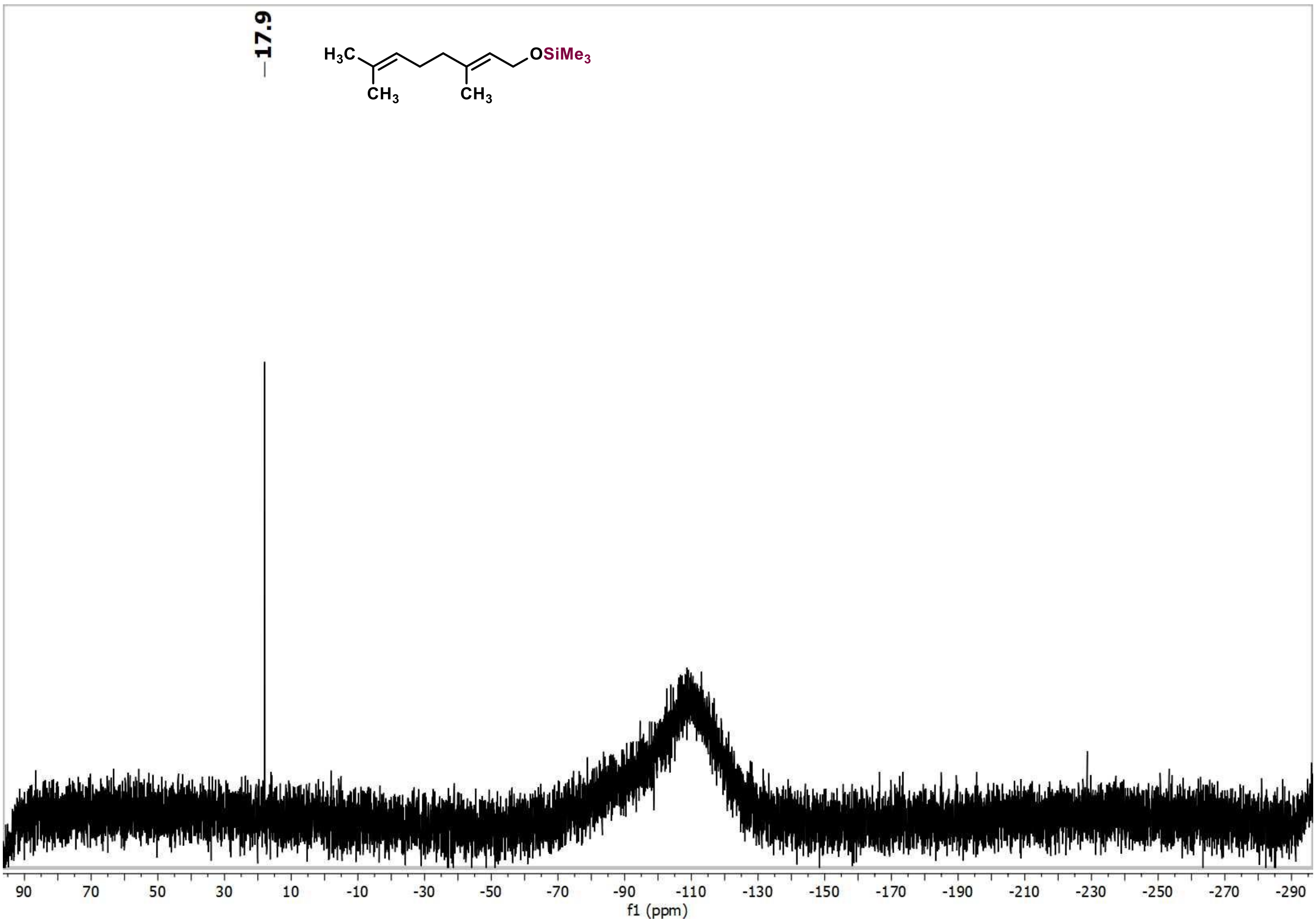

Figure S55. ^{29}Si NMR (79 MHz, Chloroform-d, 25°C) of (*E*)-((3,7-dimethylocta-2,6-dien-1-yl)oxy)trimethylsilane (**3q**).

(Cyclohexylmethoxy)trimethylsilane (**3r**)

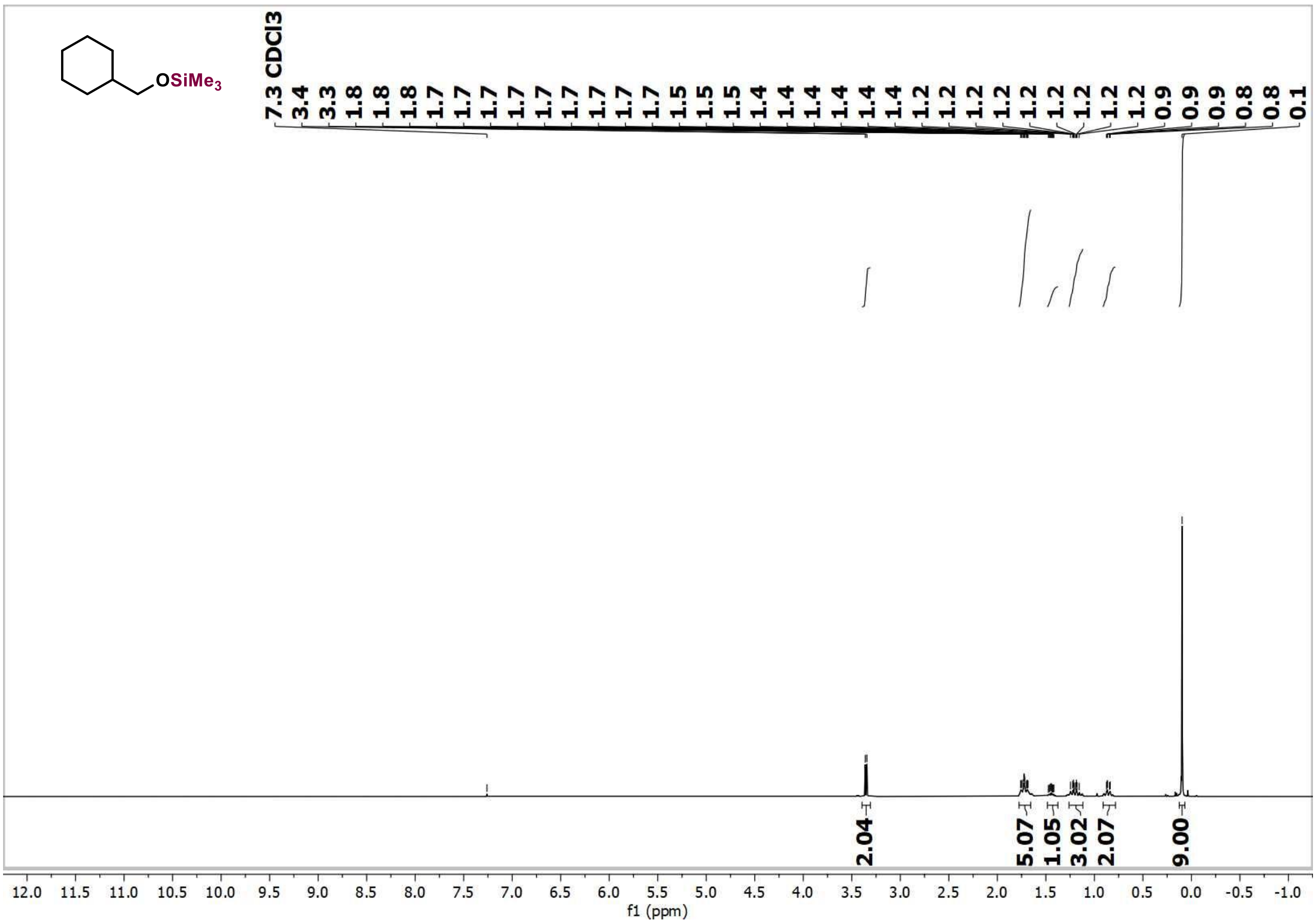

Figure S56. ^{1}H NMR (400 MHz, Chloroform-d, 25°C) of (cyclohexylmethoxy)trimethylsilane (**3r**).

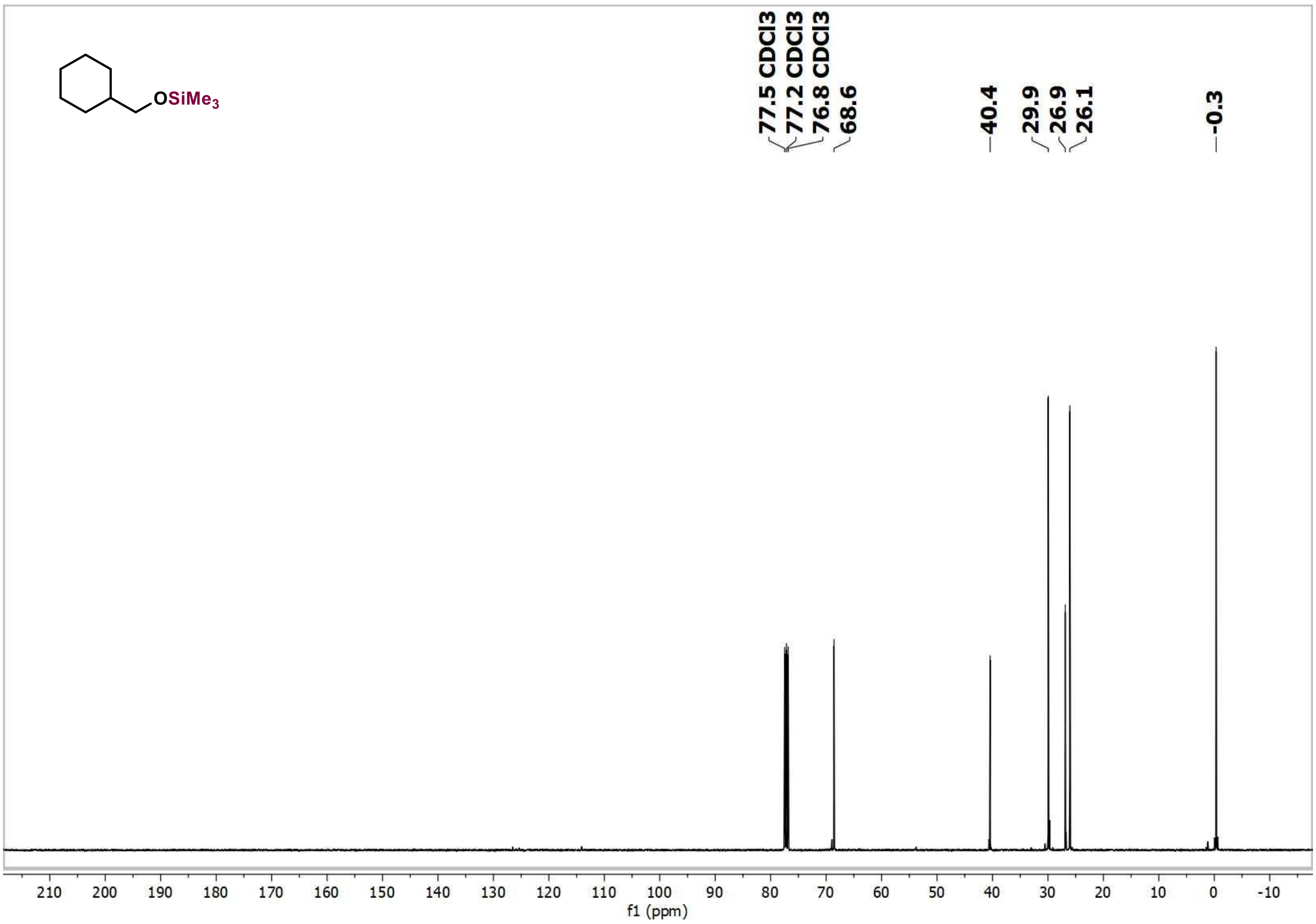

Figure S57. ^{13}C NMR (101 MHz, Chloroform-d, 25°C) of (cyclohexylmethoxy)trimethylsilane (**3r**).

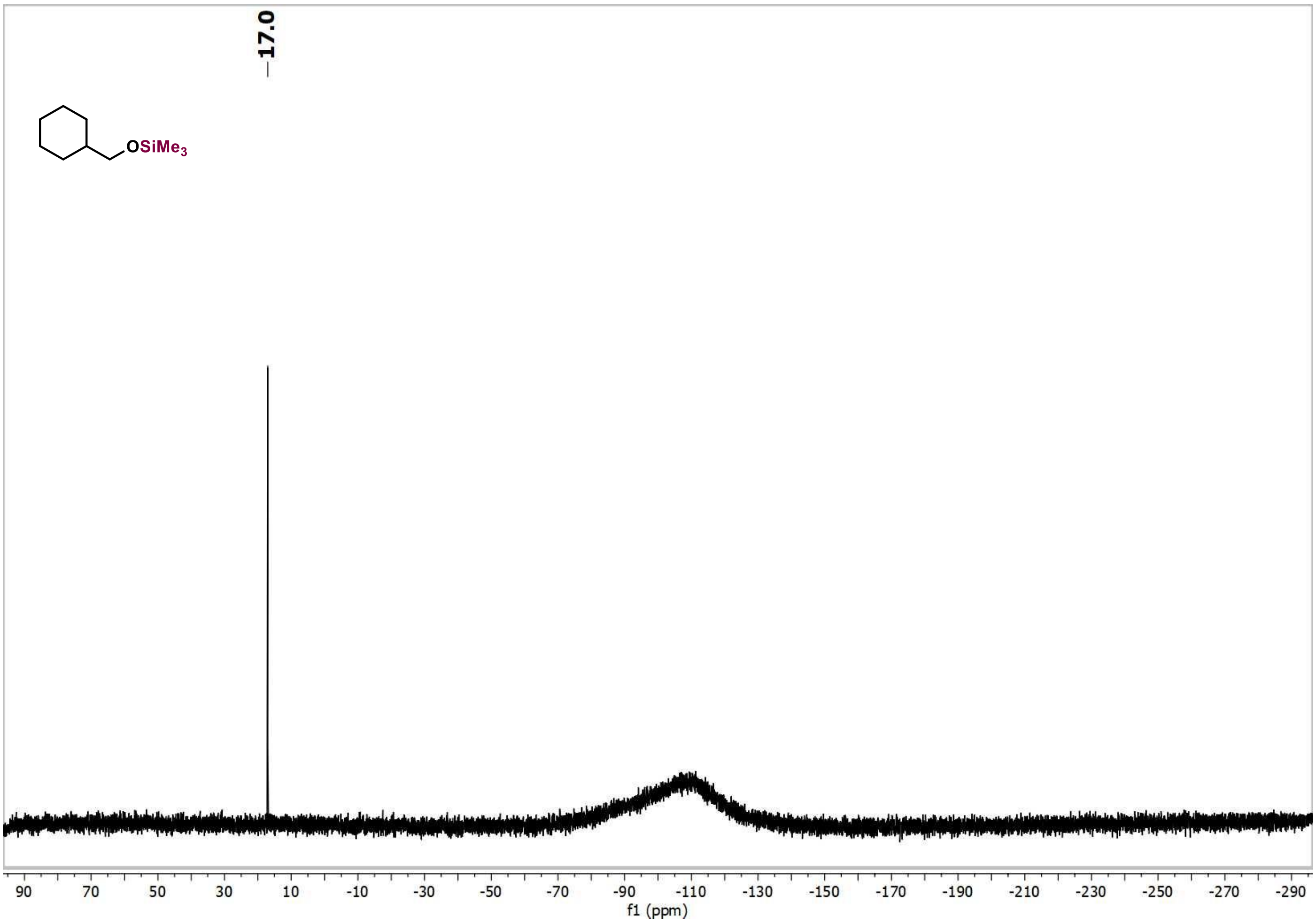

Figure S58. ^{29}Si NMR (79 MHz, Chloroform-d, 25°C) of (cyclohexylmethoxy)trimethylsilane (**3r**).

(2-Cyclohexylethoxy)trimethylsilane (**3s**)

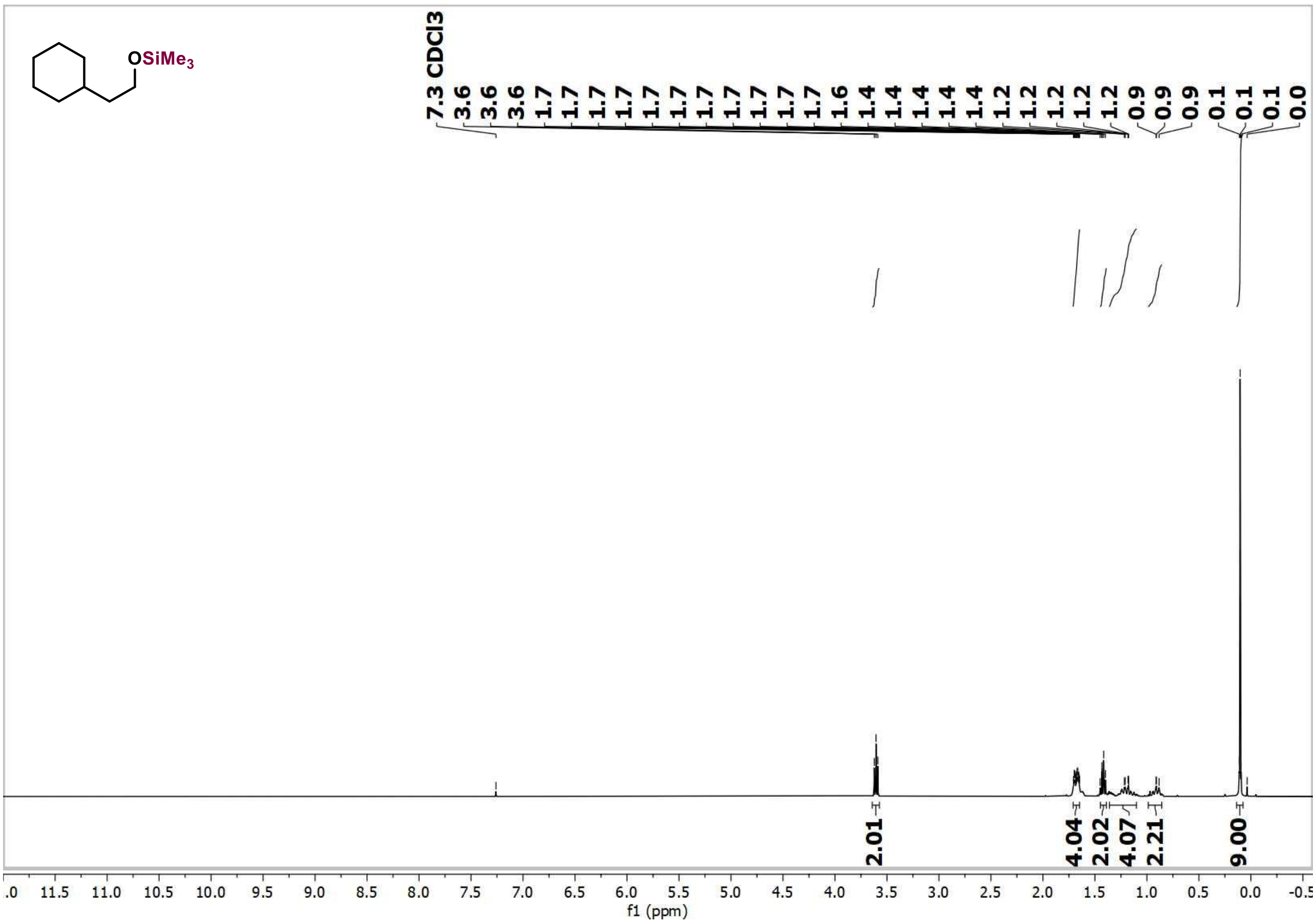

Figure S59. ^{1}H NMR (400 MHz, Chloroform-d, 25°C) of (2-cyclohexylethoxy)trimethylsilane (**3s**).

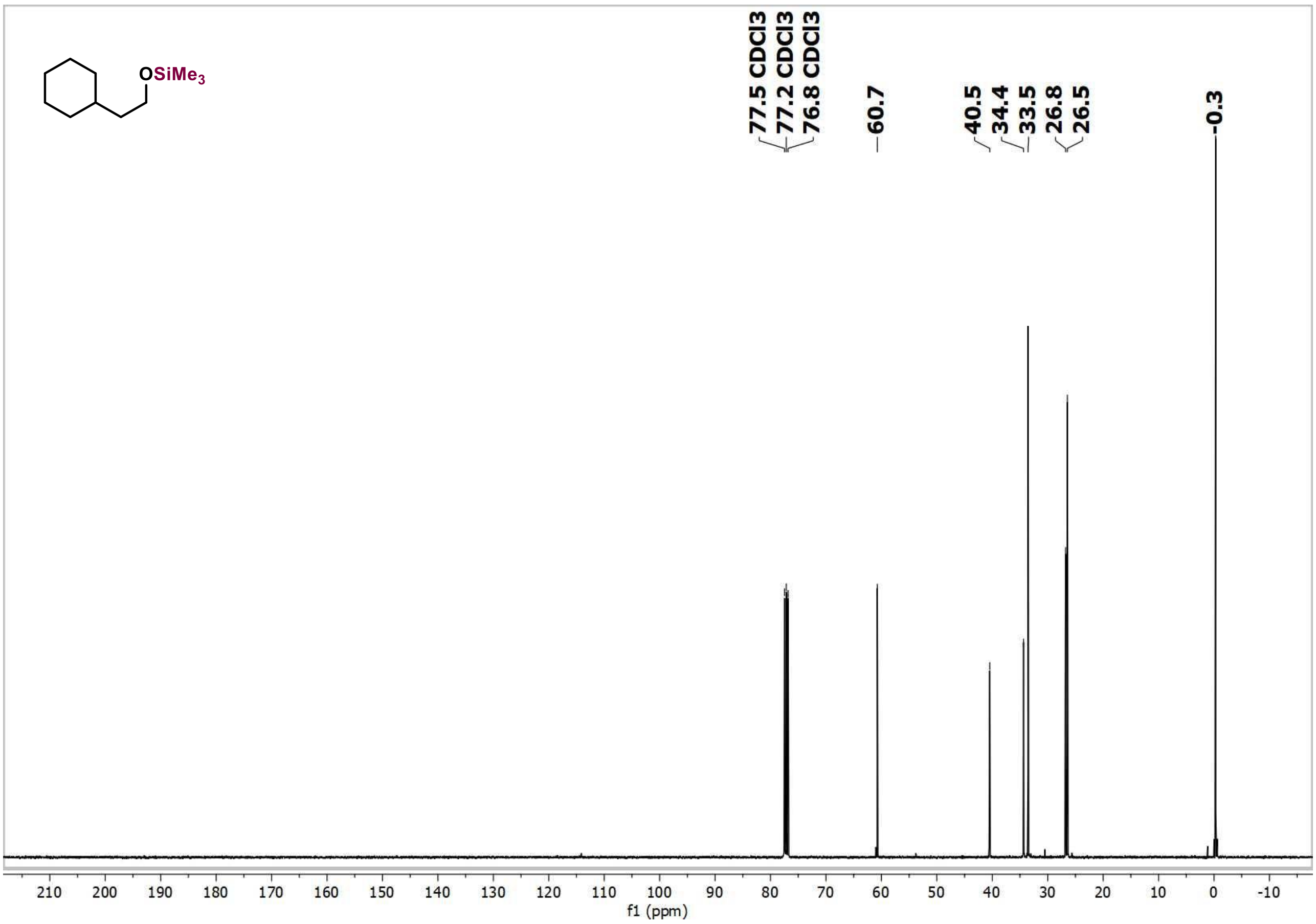

Figure S60. ^{13}C NMR (101 MHz, Chloroform-d, 25°C) of (2-cyclohexylethoxy)trimethylsilane (**3s**).

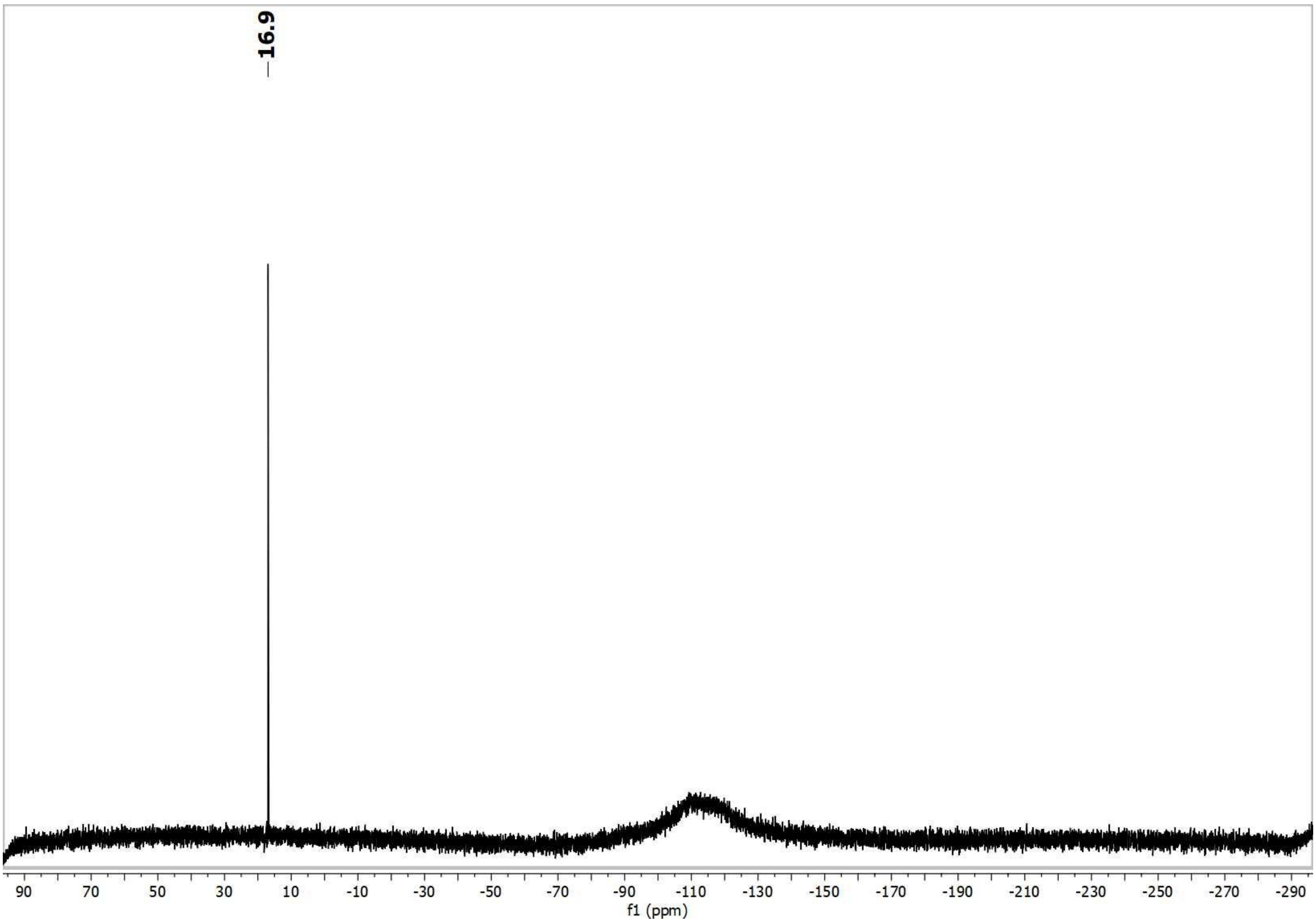

Figure S61. ^{29}Si NMR (79 MHz, Chloroform-d, 25°C) of (2-cyclohexylethoxy)trimethylsilane (**3s**).

((9-Bromononyl)oxy)trimethylsilane (**3t**)

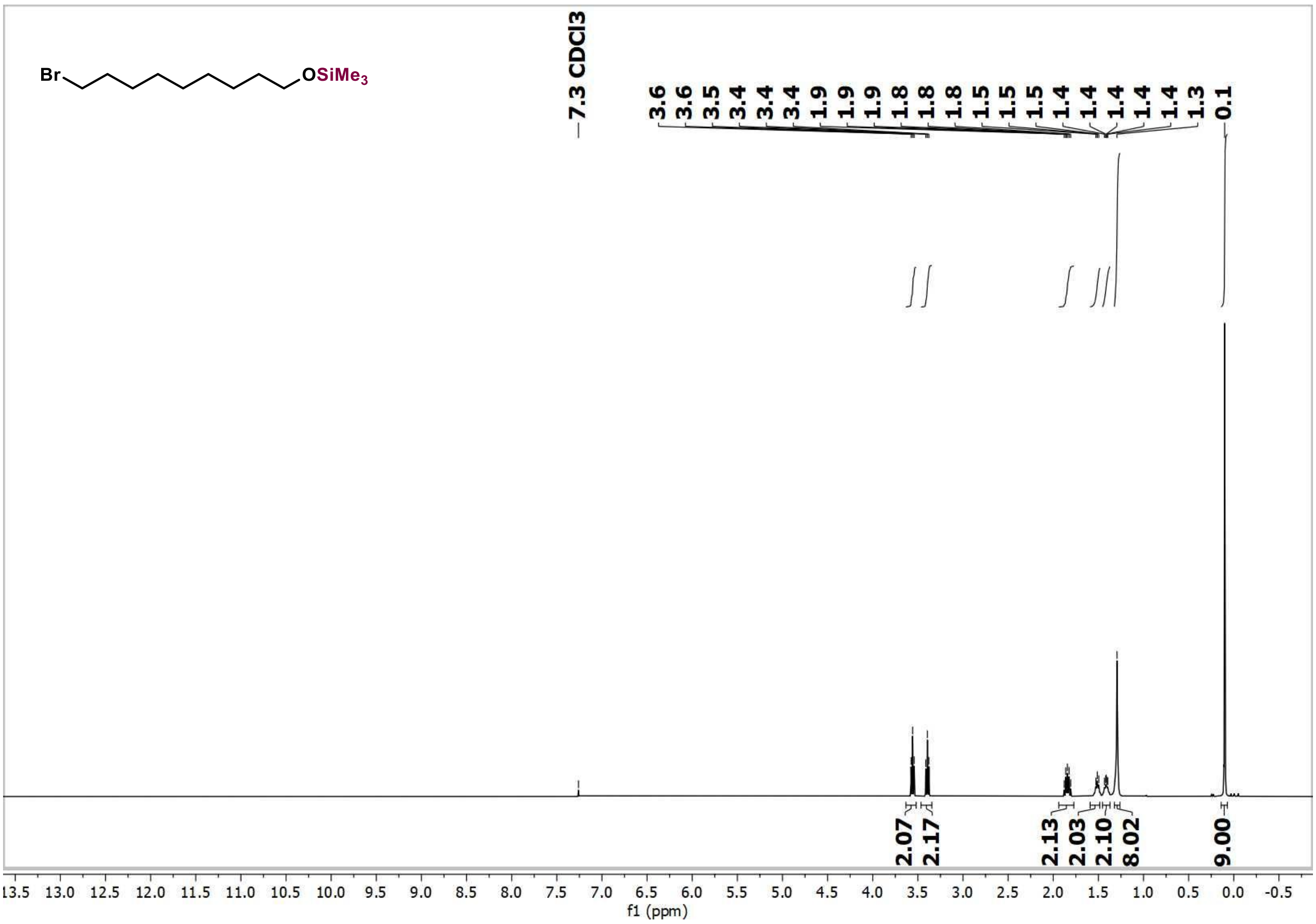

Figure S62. ^{1}H NMR (400 MHz, Chloroform-d, 25°C) of ((9-bromononyl)oxy)trimethylsilane (**3t**).

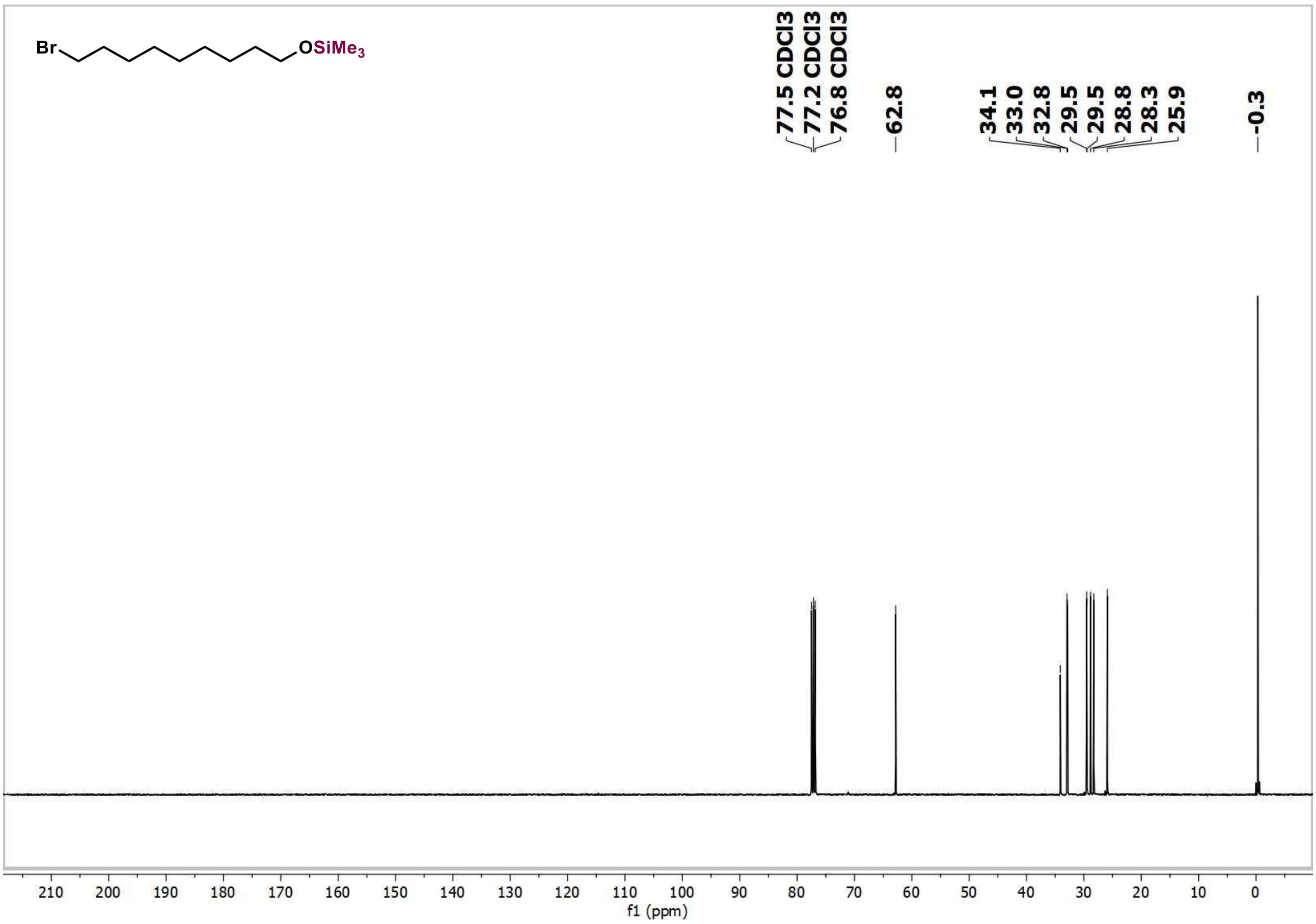

Figure S63. ^{13}C NMR (101 MHz, Chloroform-d, 25°C) of ((9-bromononyl)oxy)trimethylsilane (**3t**).

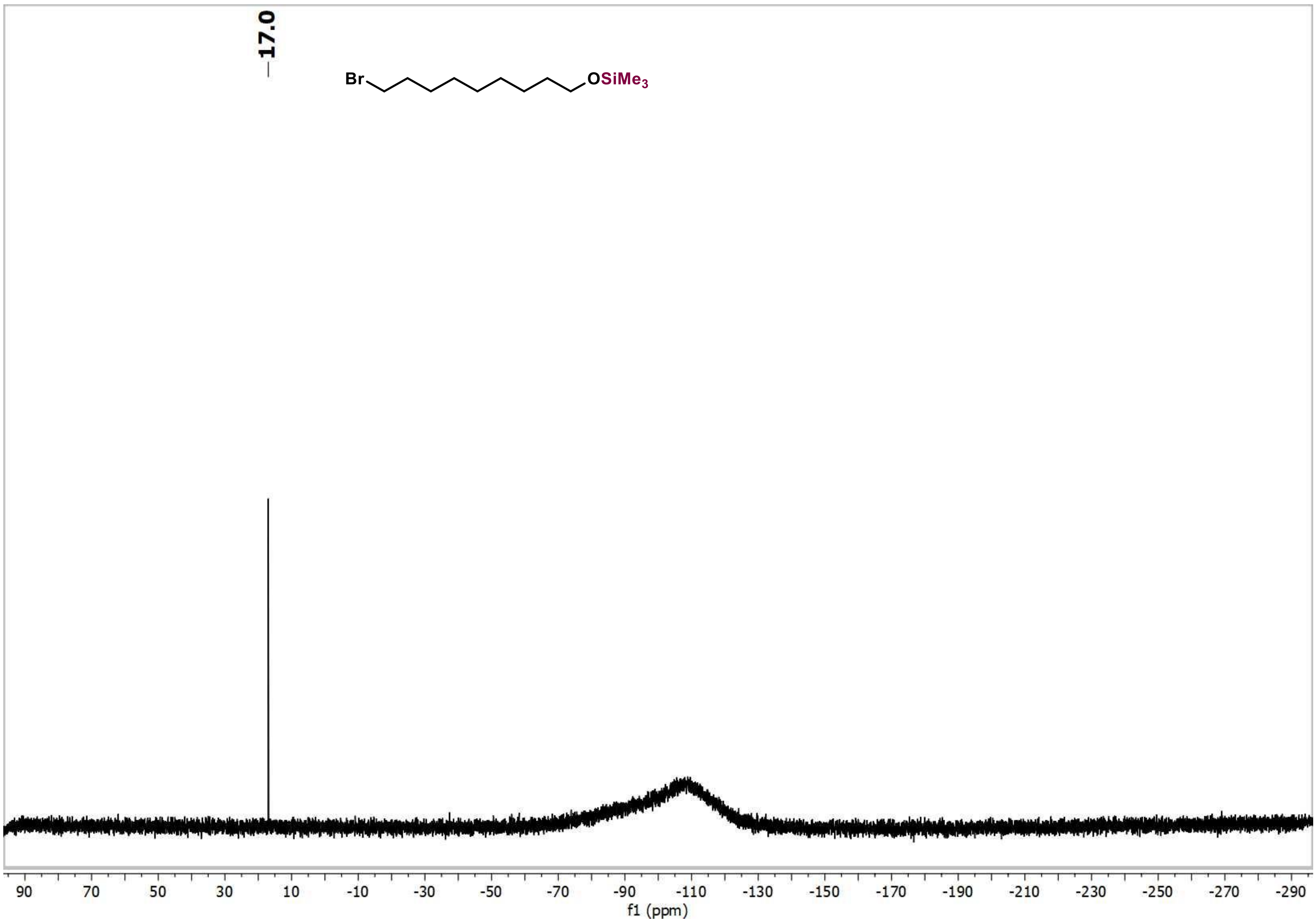

Figure S64. ^{29}Si NMR (79 MHz, Chloroform-d, 25°C) of ((9-bromononyl)oxy)trimethylsilane (**3t**).

2,2,10,10-Tetramethyl-3,9-dioxa-2,10-disilaundecane (**3u**)

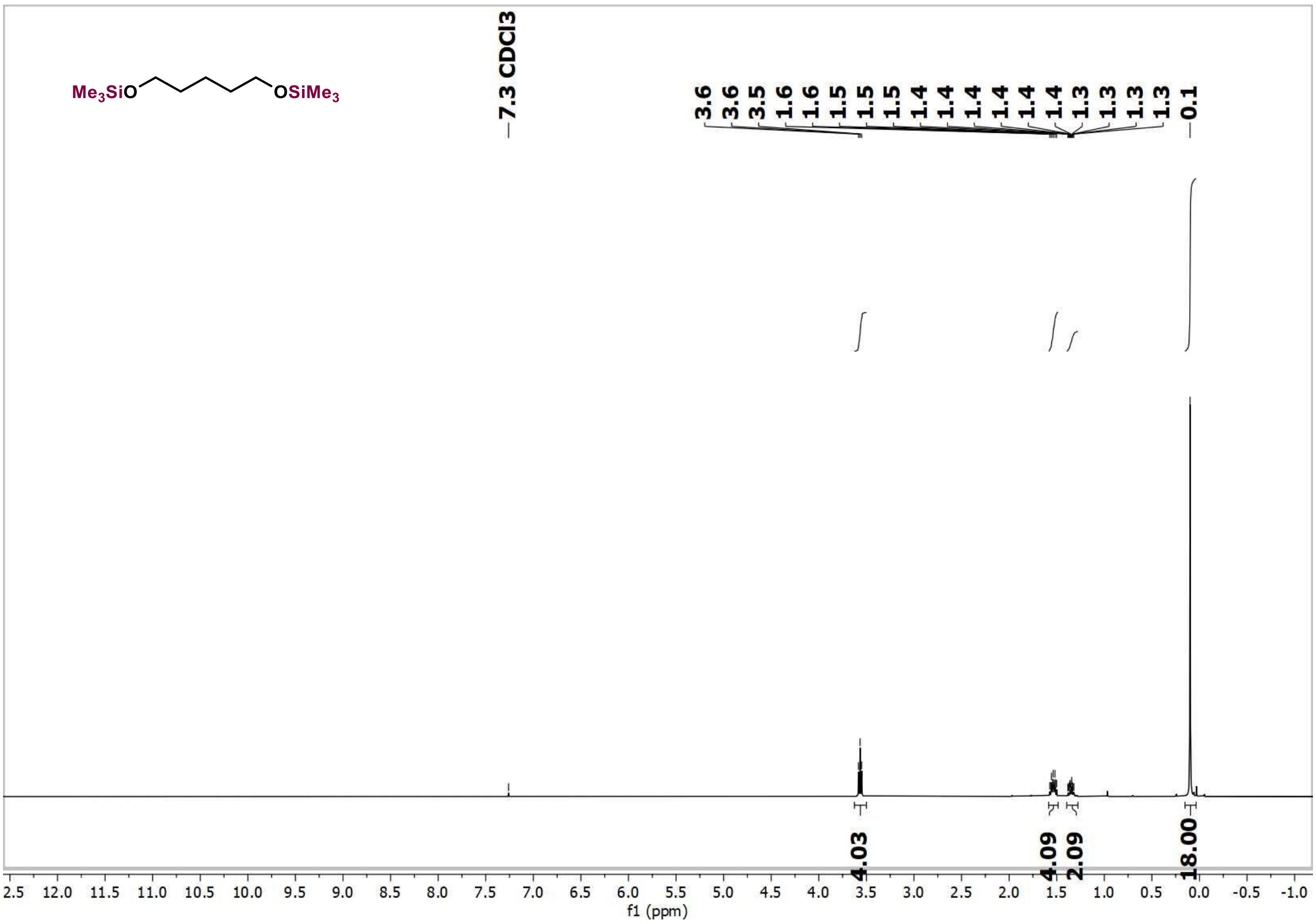

Figure S65. ^{1}H NMR (400 MHz, Chloroform-d, 25°C) of 2,2,10,10-tetramethyl-3,9-dioxa-2,10-disilaundecane (**3u**).

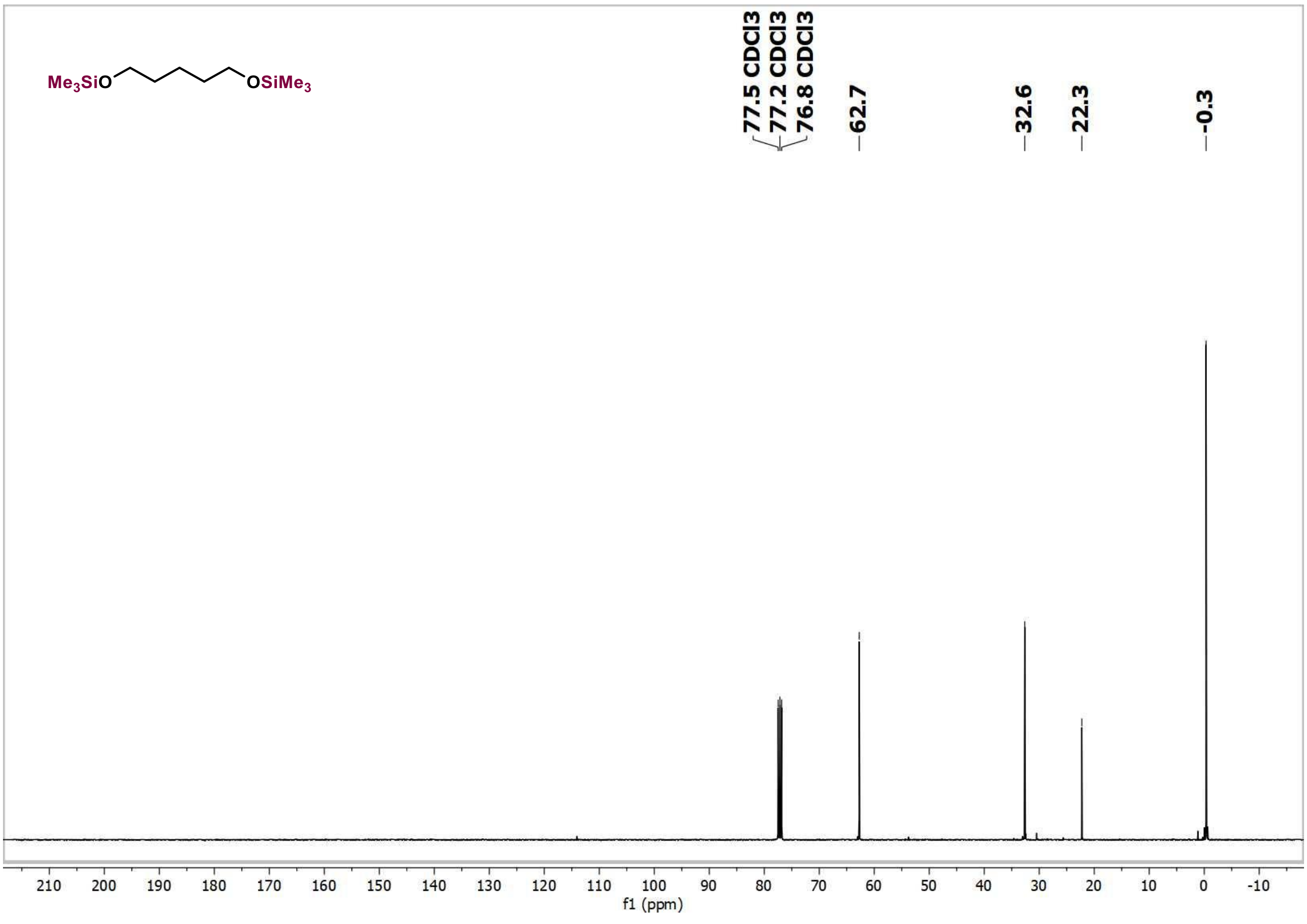

Figure S66. ^{13}C NMR (101 MHz, Chloroform-d, 25°C) of 2,2,10,10-tetramethyl-3,9-dioxa-2,10-disilaundecane (**3u**).

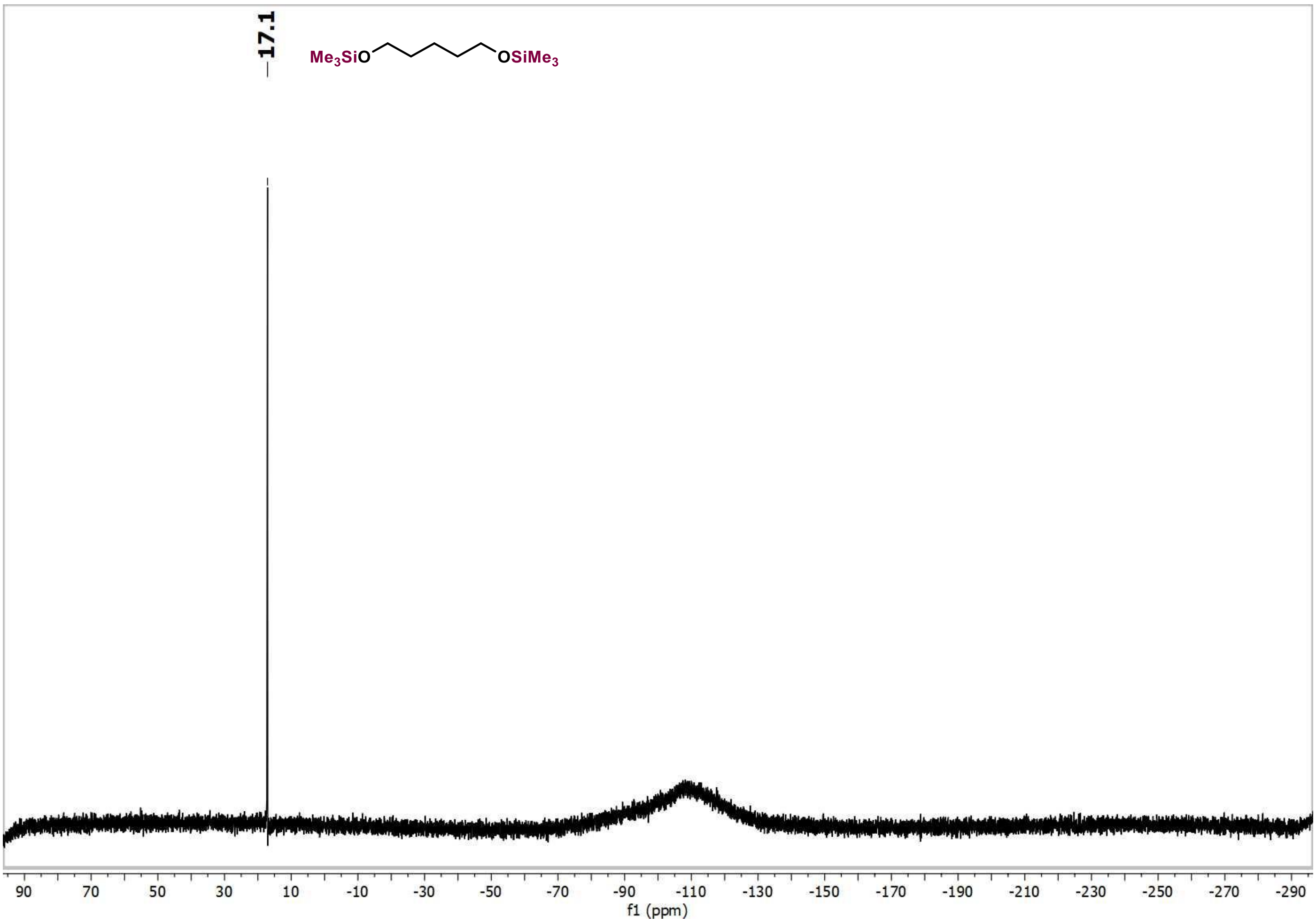

Figure S67. ^{29}Si NMR (79 MHz, Chloroform-d, 25°C) of 2,2,10,10-tetramethyl-3,9-dioxa-2,10-disilaundecane (**3u**).

(Benzo[d][1,3]dioxol-5-ylmethoxy)trimethylsilane (**3v**)

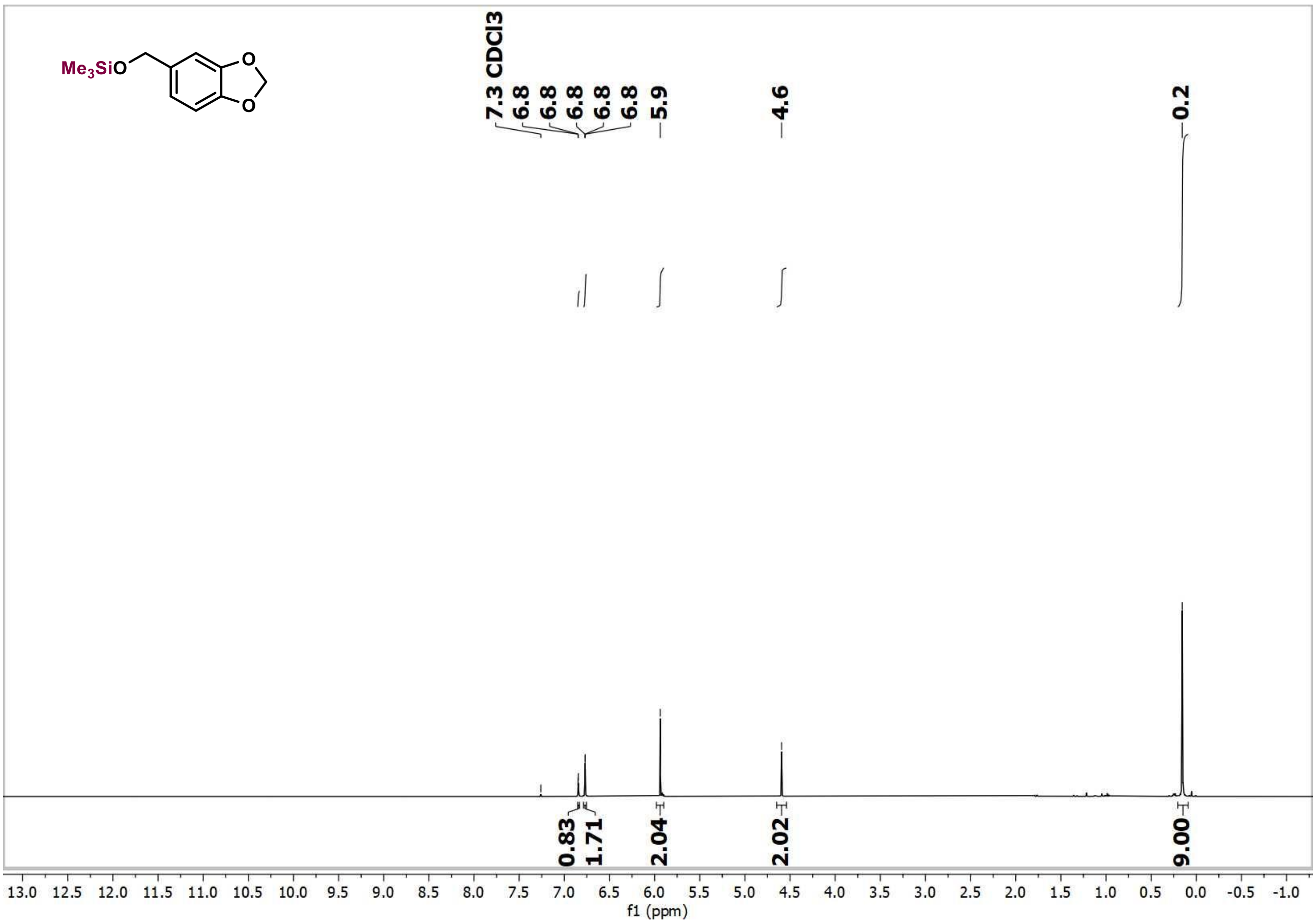

Figure S68. ^{1}H NMR (400 MHz, Chloroform-d, 25°C) of (benzo[*d*][1,3]dioxol-5-ylmethoxy)trimethylsilane (**3v**).

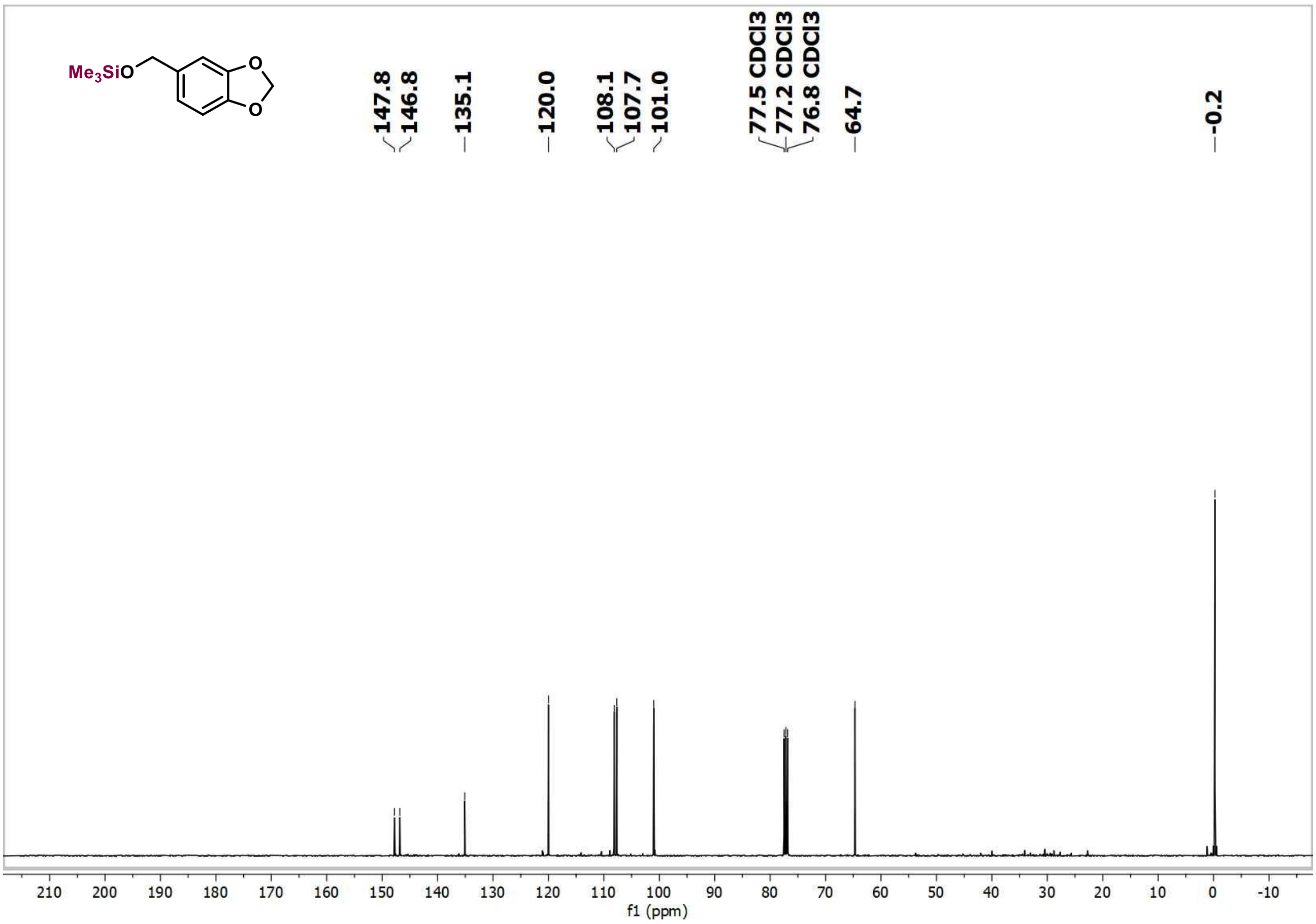

Figure S69. ^{13}C NMR (101 MHz, Chloroform-d, 25°C) of (benzo[*d*][1,3]dioxol-5-ylmethoxy)trimethylsilane (**3v**).

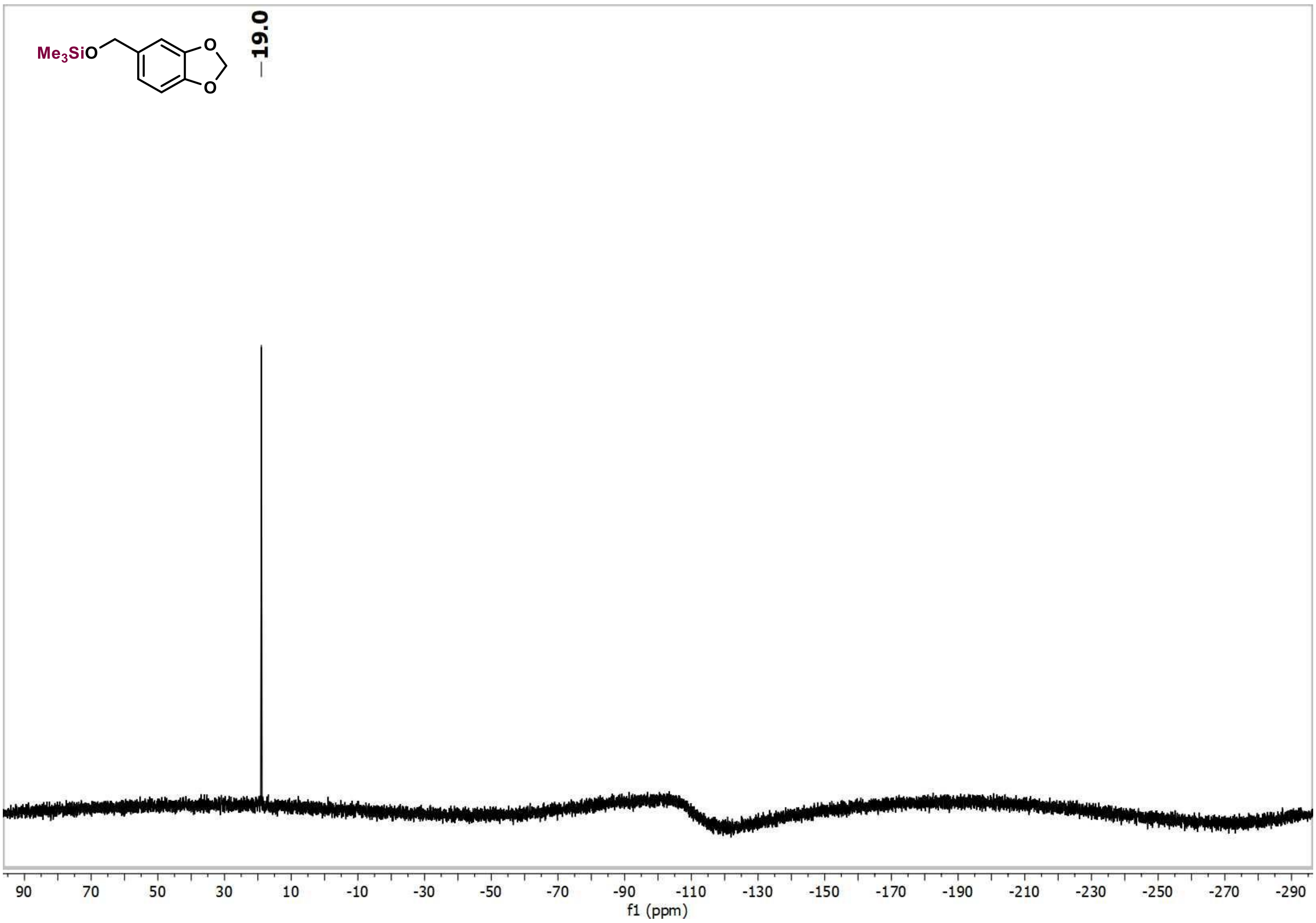

Figure S70. ^{29}Si NMR (79 MHz, Chloroform-d, 25°C) of (benzo[*d*][1,3]dioxol-5-ylmethoxy)trimethylsilane (**3v**).

6-(((Trimethylsilyl)oxy)methyl)benzo[d]thiazole (**3w**)

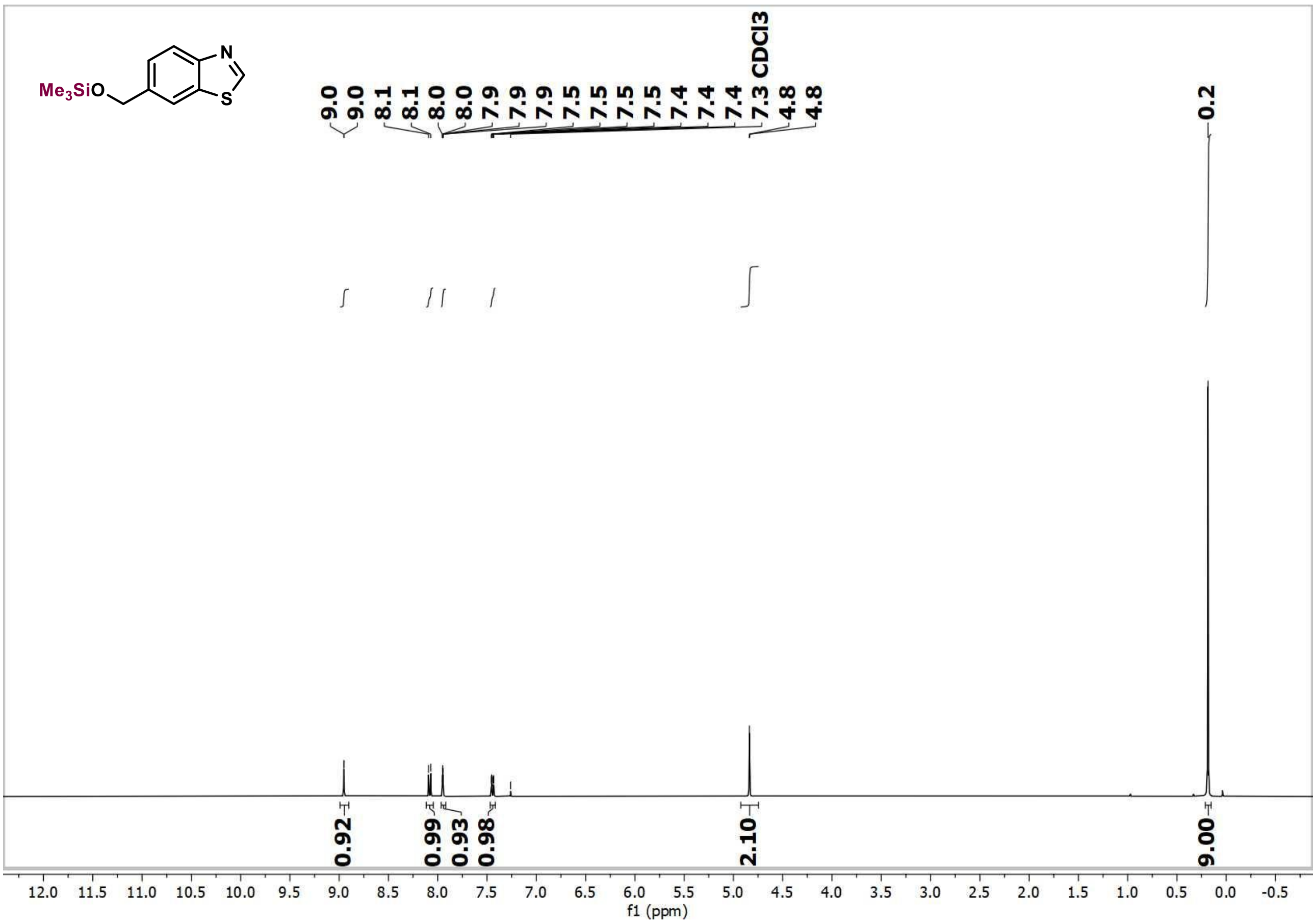

Figure S71. ^{1}H NMR (400 MHz, Chloroform-d, 25°C) of 6-(((trimethylsilyl)oxy)methyl)benzo[*d*]thiazole (**3w**).

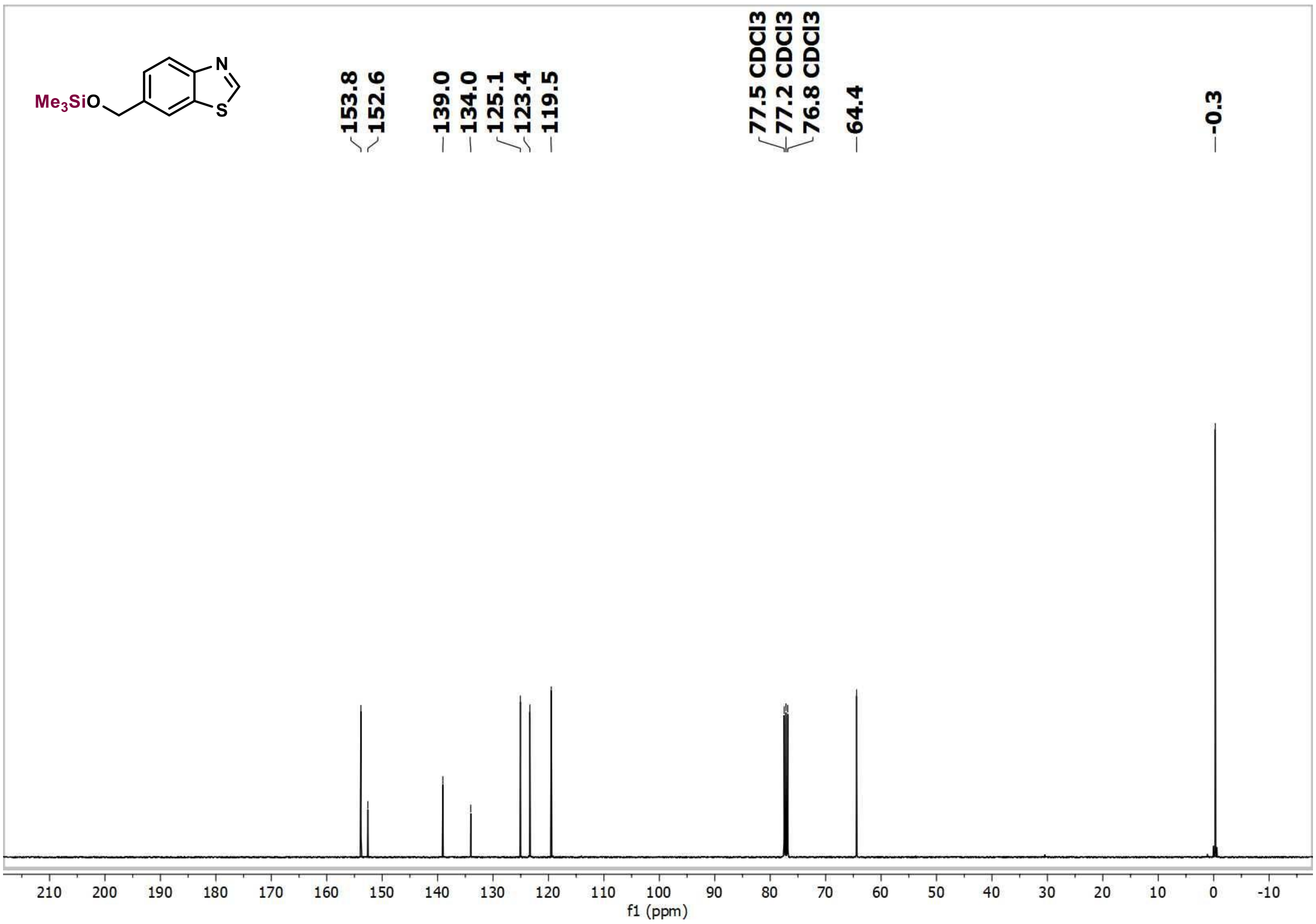

Figure S72. ^{13}C NMR (101 MHz, Chloroform-d, 25°C) of 6-(((trimethylsilyl)oxy)methyl)benzo[*d*]thiazole (**3w**).

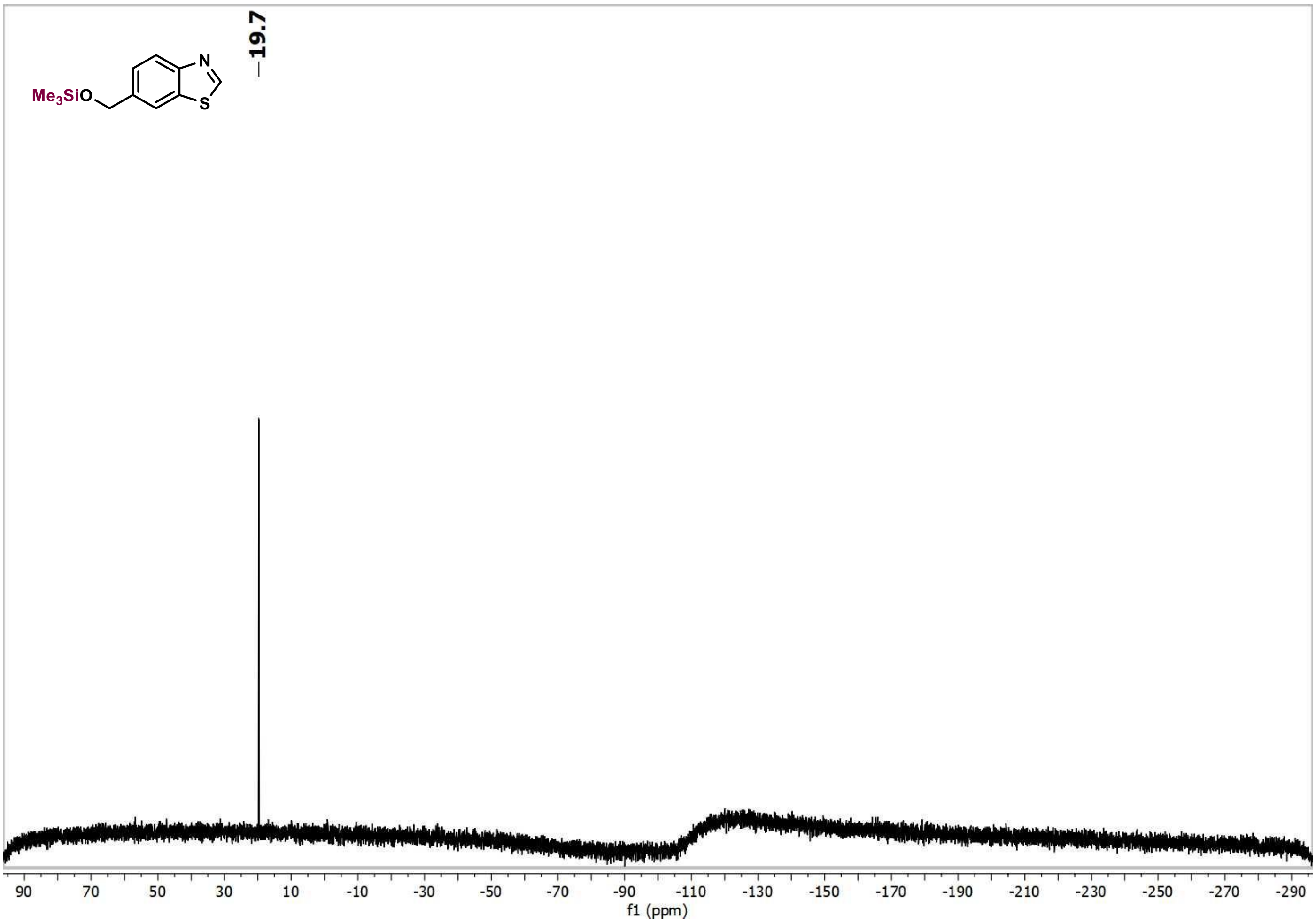

Figure S73. ^{29}Si NMR (79 MHz, Chloroform-d, 25°C) of 6-(((trimethylsilyl)oxy)methyl)benzo[*d*]thiazole (**3w**).

((9*H*-Fluoren-9-yl)oxy)trimethylsilane (**3x**)

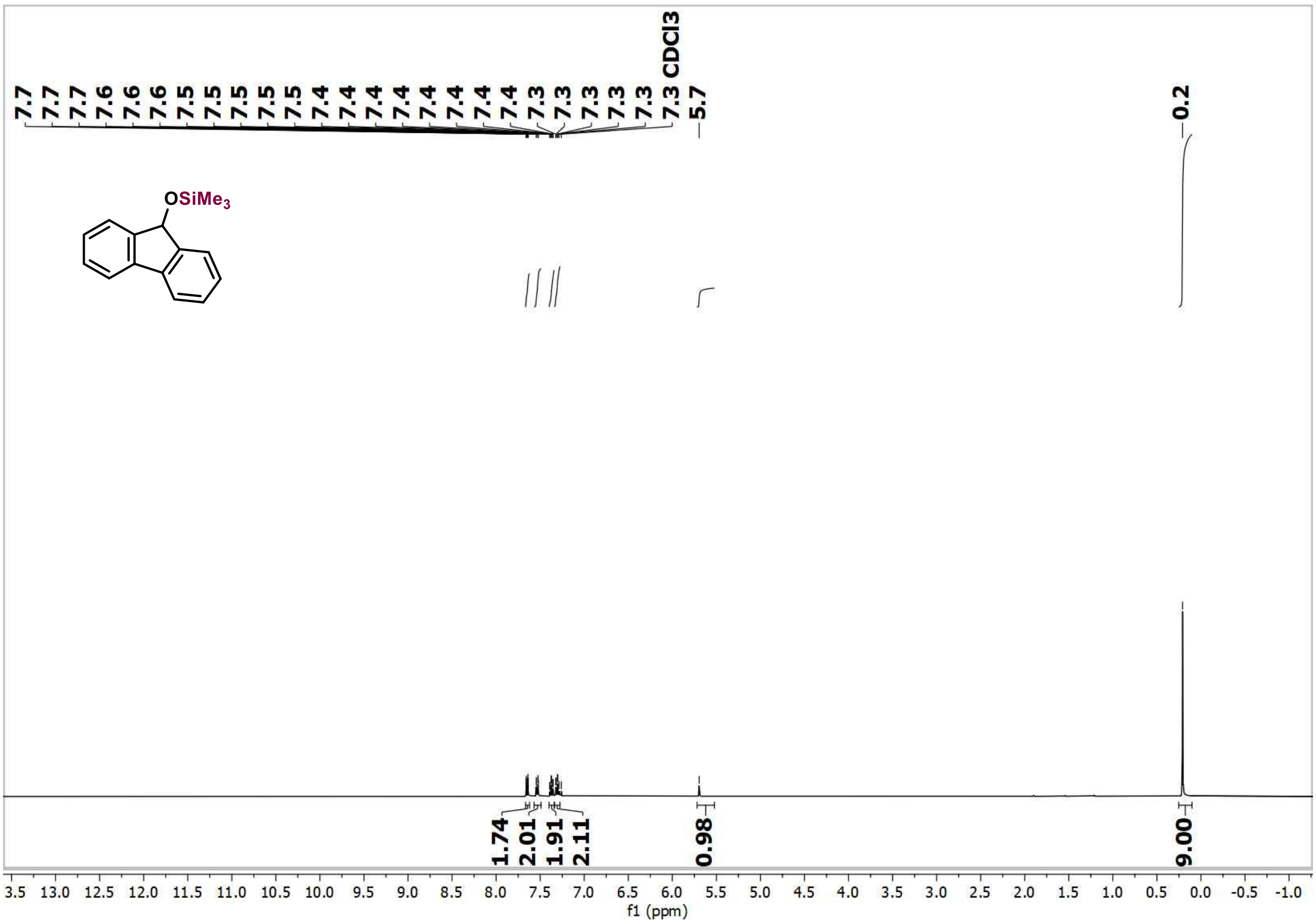

Figure S74. ^{1}H NMR (400 MHz, Chloroform-d, 25°C) of ((9*H*-fluoren-9-yl)oxy)trimethylsilane (**3x**).

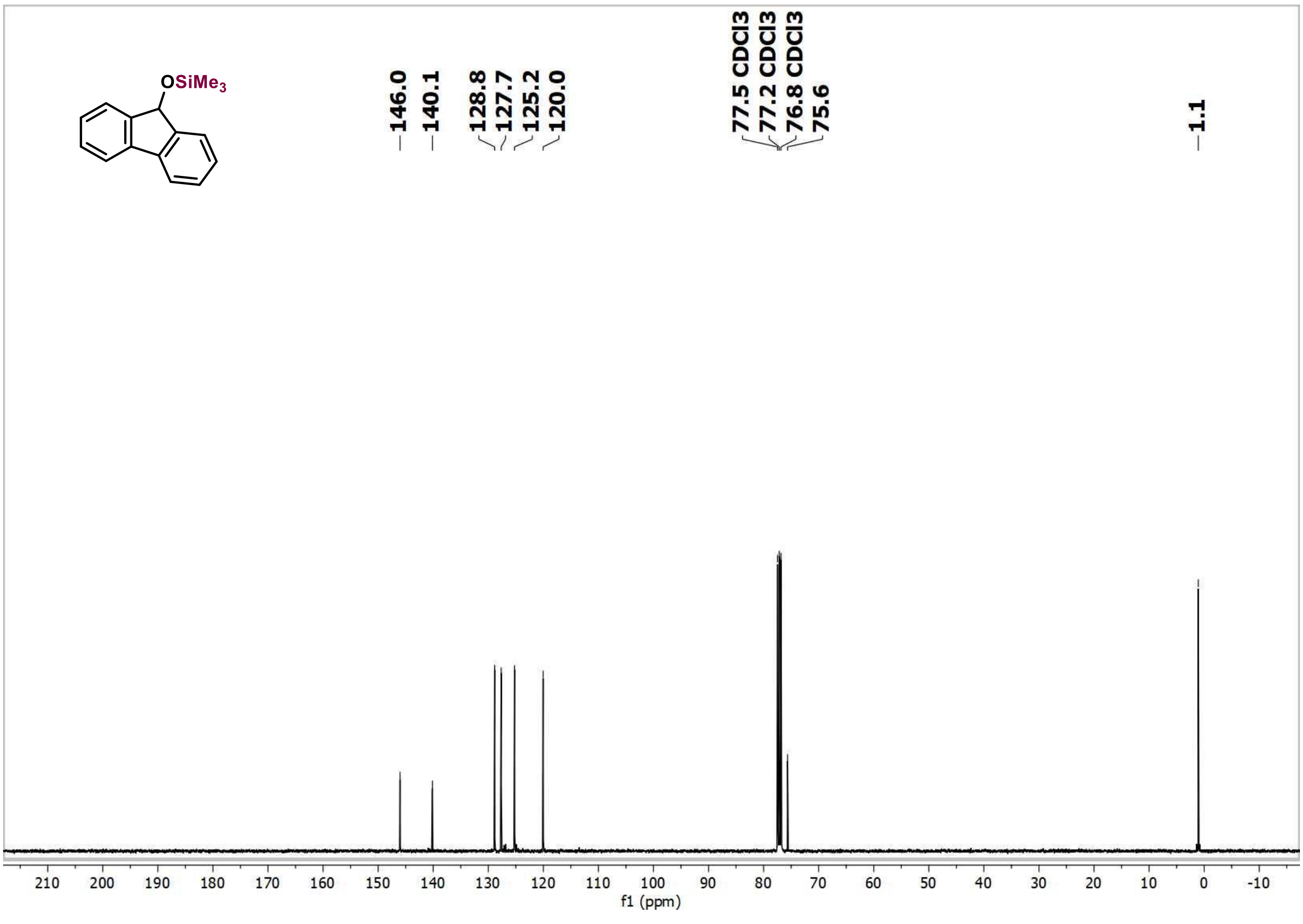

Figure S75. ^{13}C NMR (101 MHz, Chloroform-d, 25°C) of ((9*H*-fluoren-9-yl)oxy)trimethylsilane (**3x**).

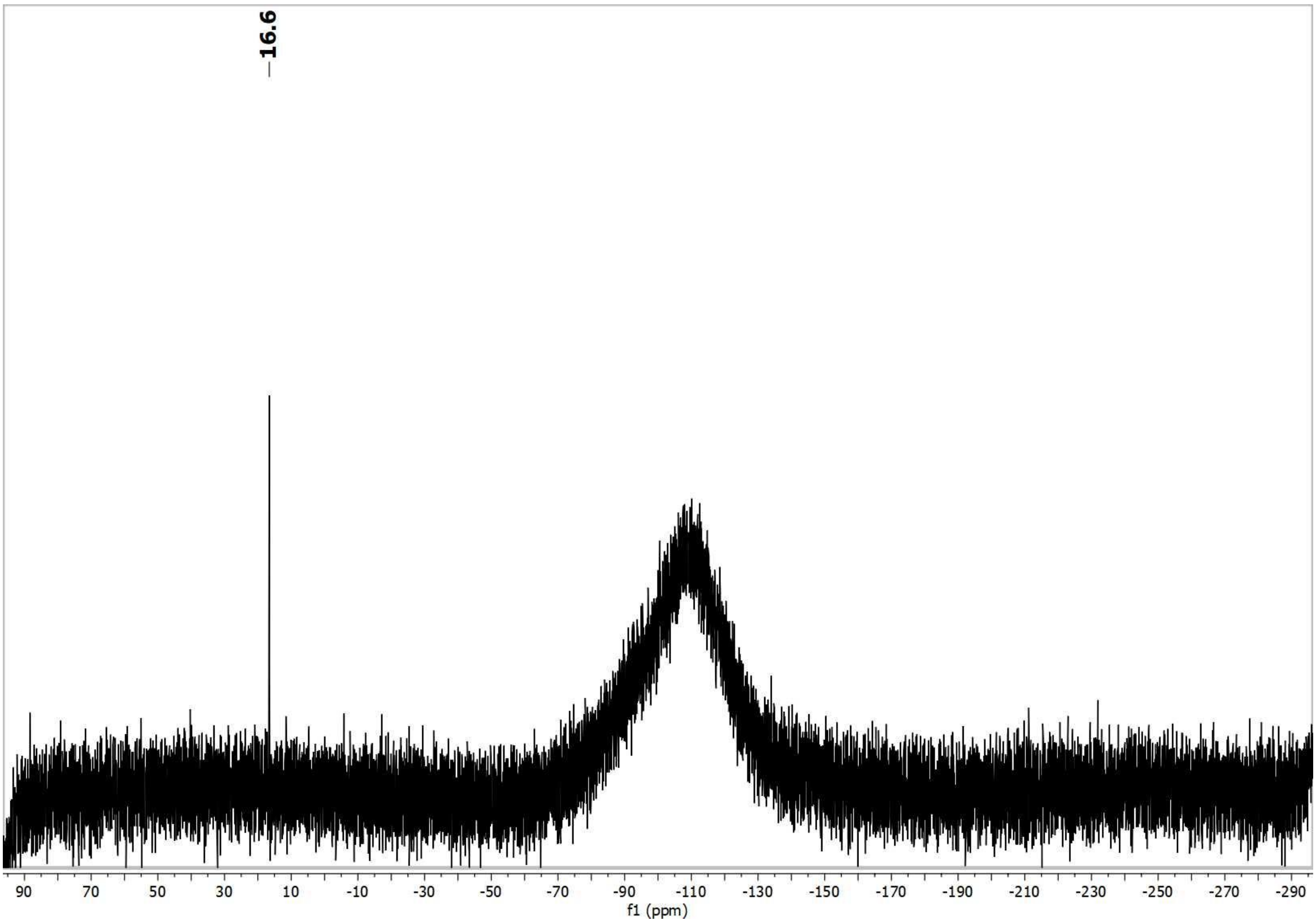

Figure S76. ^{29}Si NMR (79 MHz, Chloroform-d, 25°C) of ((*9H*-fluoren-9-yl)oxy)trimethylsilane (**3x**).

(1-(4-Fluorophenyl)ethoxy)trimethylsilane (**3y**)

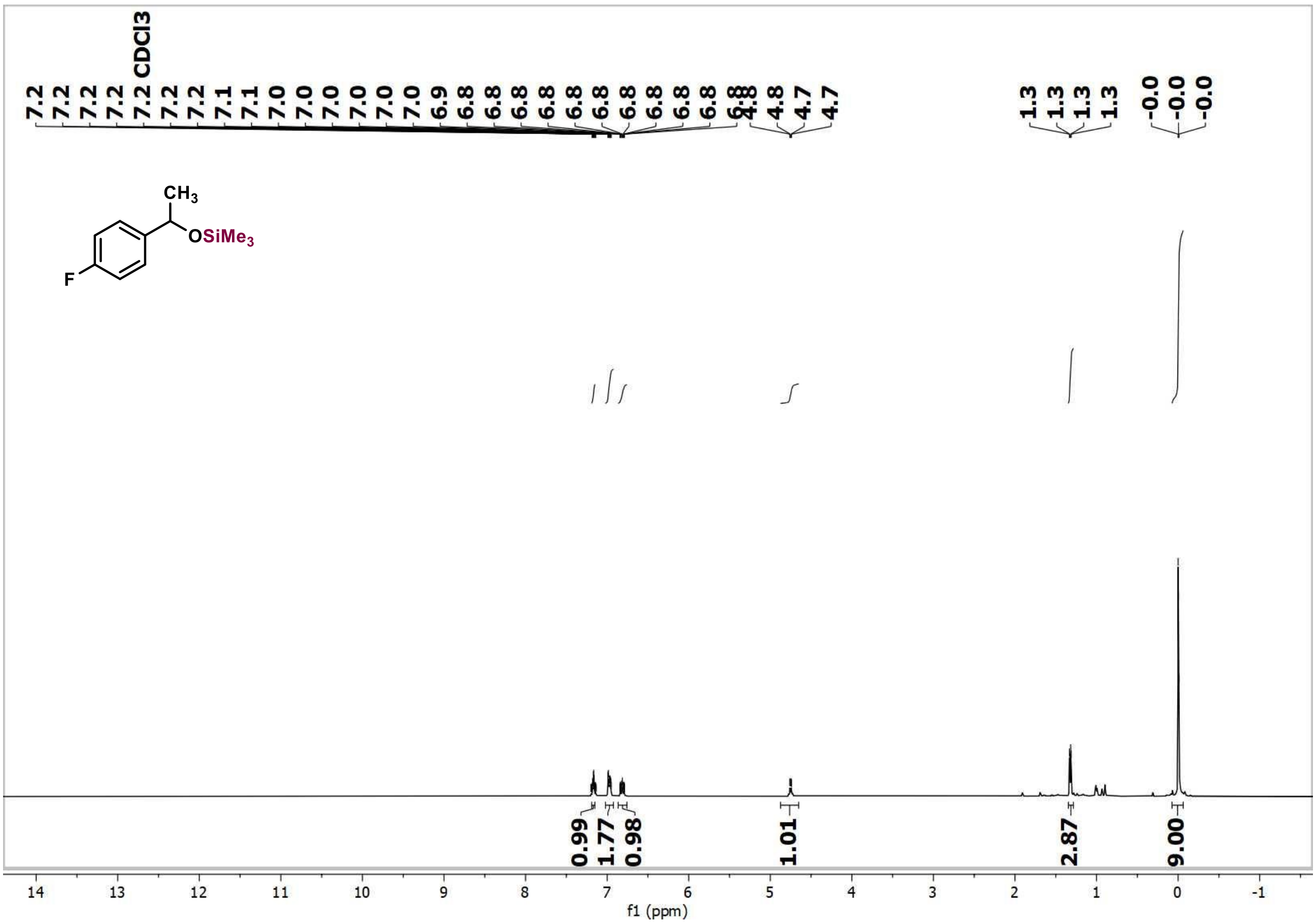

Figure S77. ^{1}H NMR (400 MHz, Chloroform-d, 25°C) of (1-(4-fluorophenyl)ethoxy)trimethylsilane (**3y**).

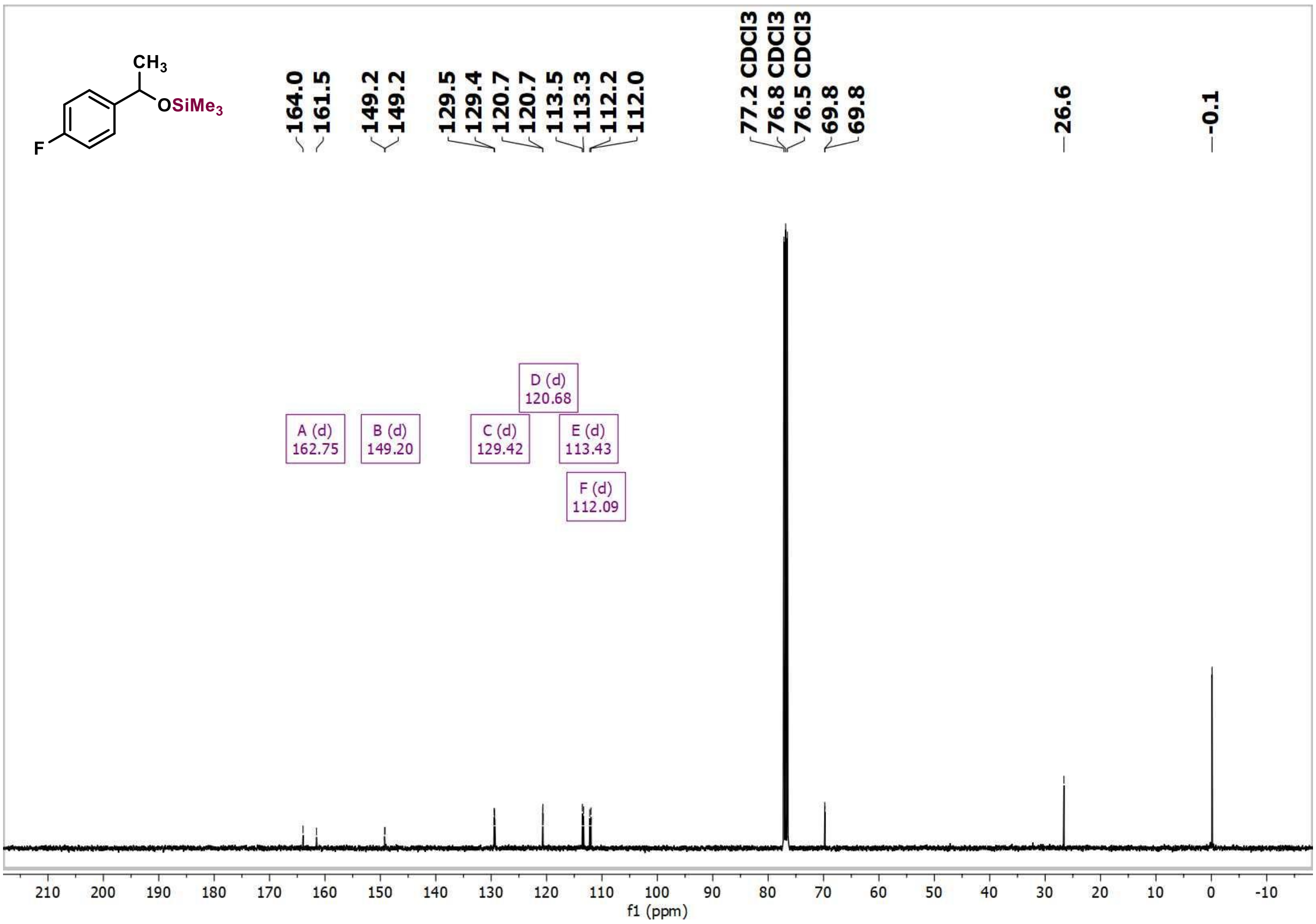

Figure S78. ^{13}C NMR (101 MHz, Chloroform-d, 25°C) of (1-(4-fluorophenyl)ethoxy)trimethylsilane (**3y**).

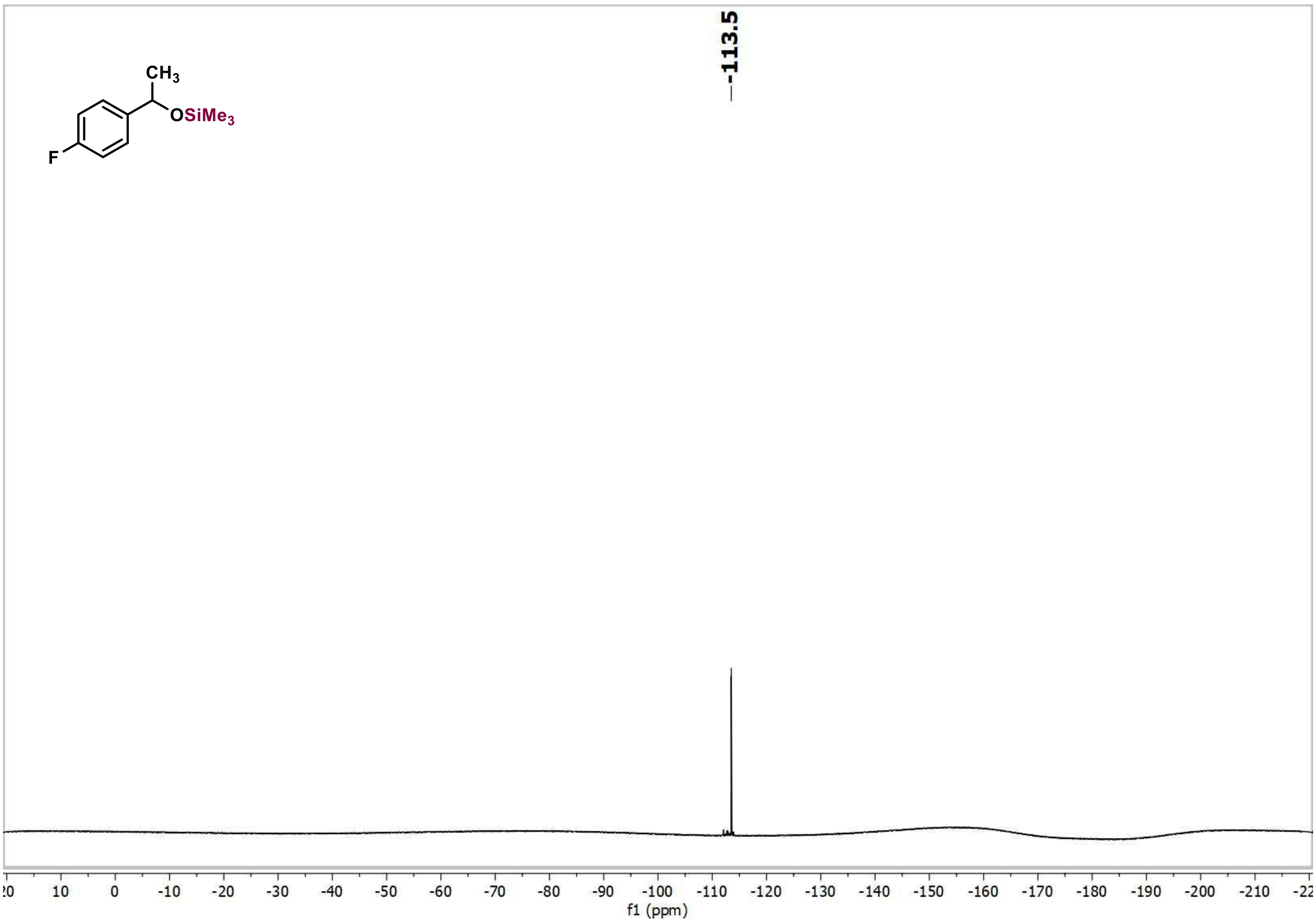

Figure S79. ^{19}F NMR (377 MHz, Chloroform-d, 25°C) of (1-(4-fluorophenyl)ethoxy)trimethylsilane (**3y**).

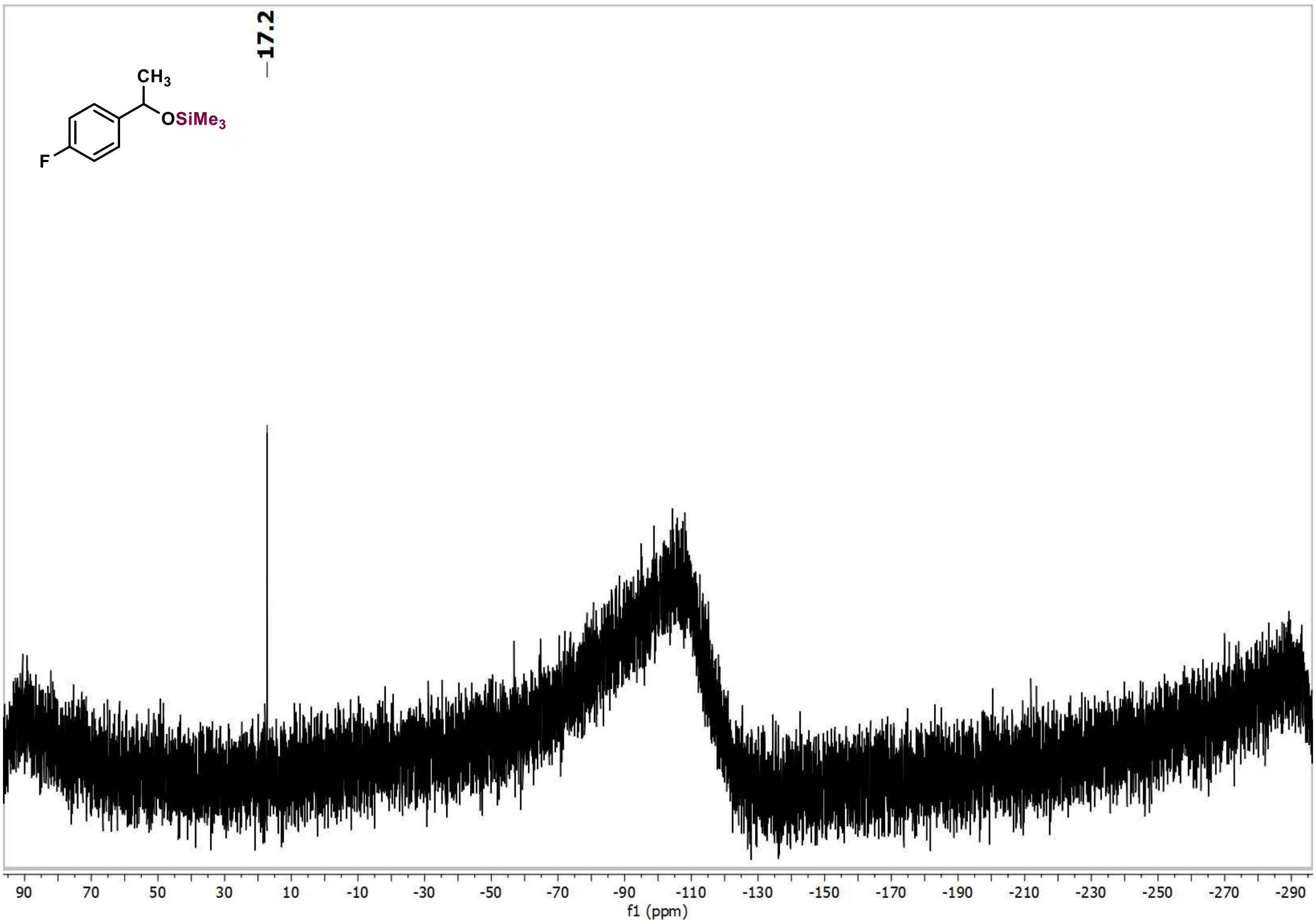

Figure S80. ^{29}Si NMR (79 MHz, Chloroform-d, 25°C) of (1-(4-fluorophenyl)ethoxy)trimethylsilane (**3y**).

(1-(4-Chlorophenyl)ethoxy)trimethylsilane (**3z**)

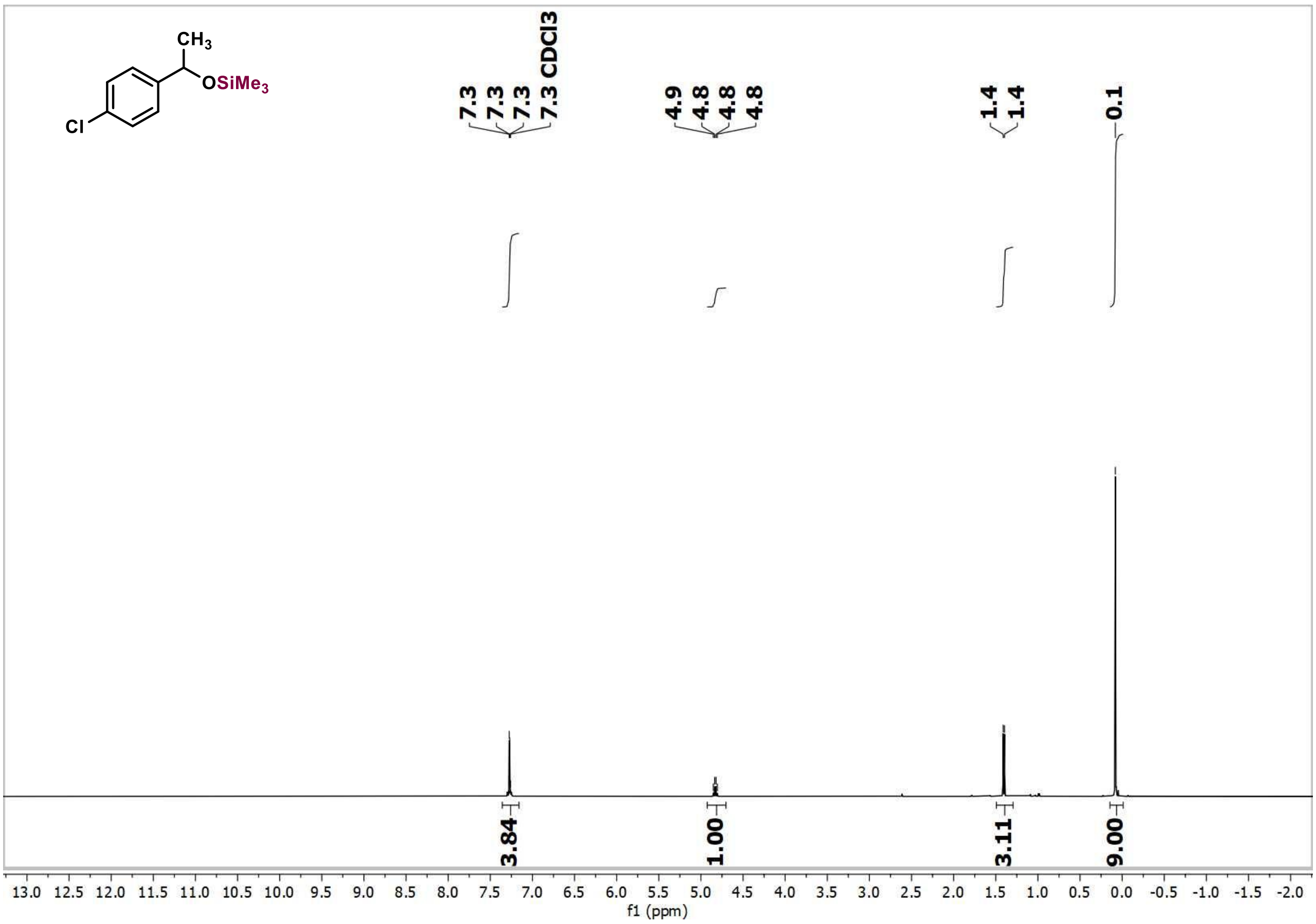

Figure S81. ^{1}H NMR (400 MHz, Chloroform-d, 25°C) of (1-(4-chlorophenyl)ethoxy)trimethylsilane (**3z**).

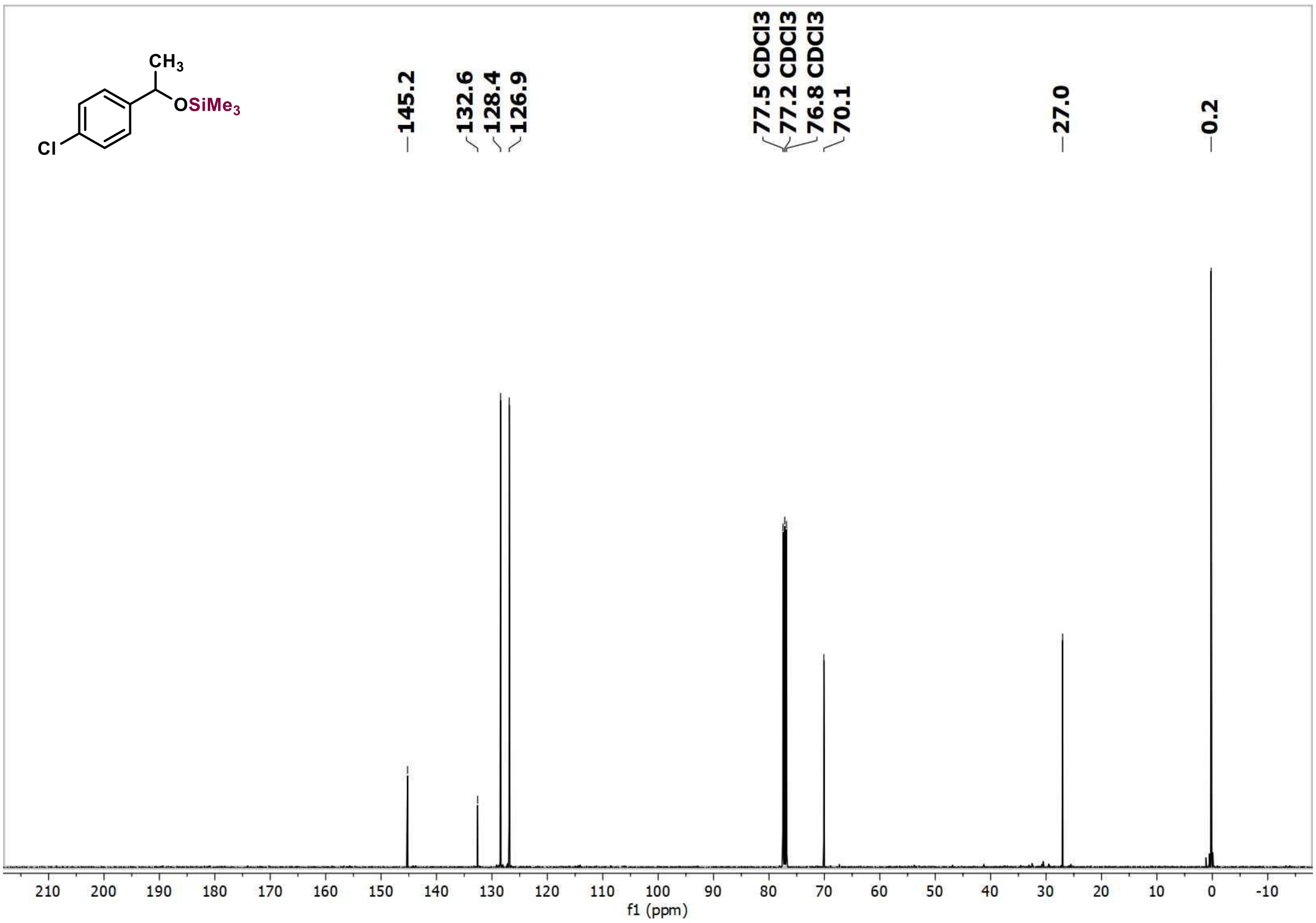

Figure S82. ^{13}C NMR (101 MHz, Chloroform-d, 25°C) of (1-(4-chlorophenyl)ethoxy)trimethylsilane (**3z**).

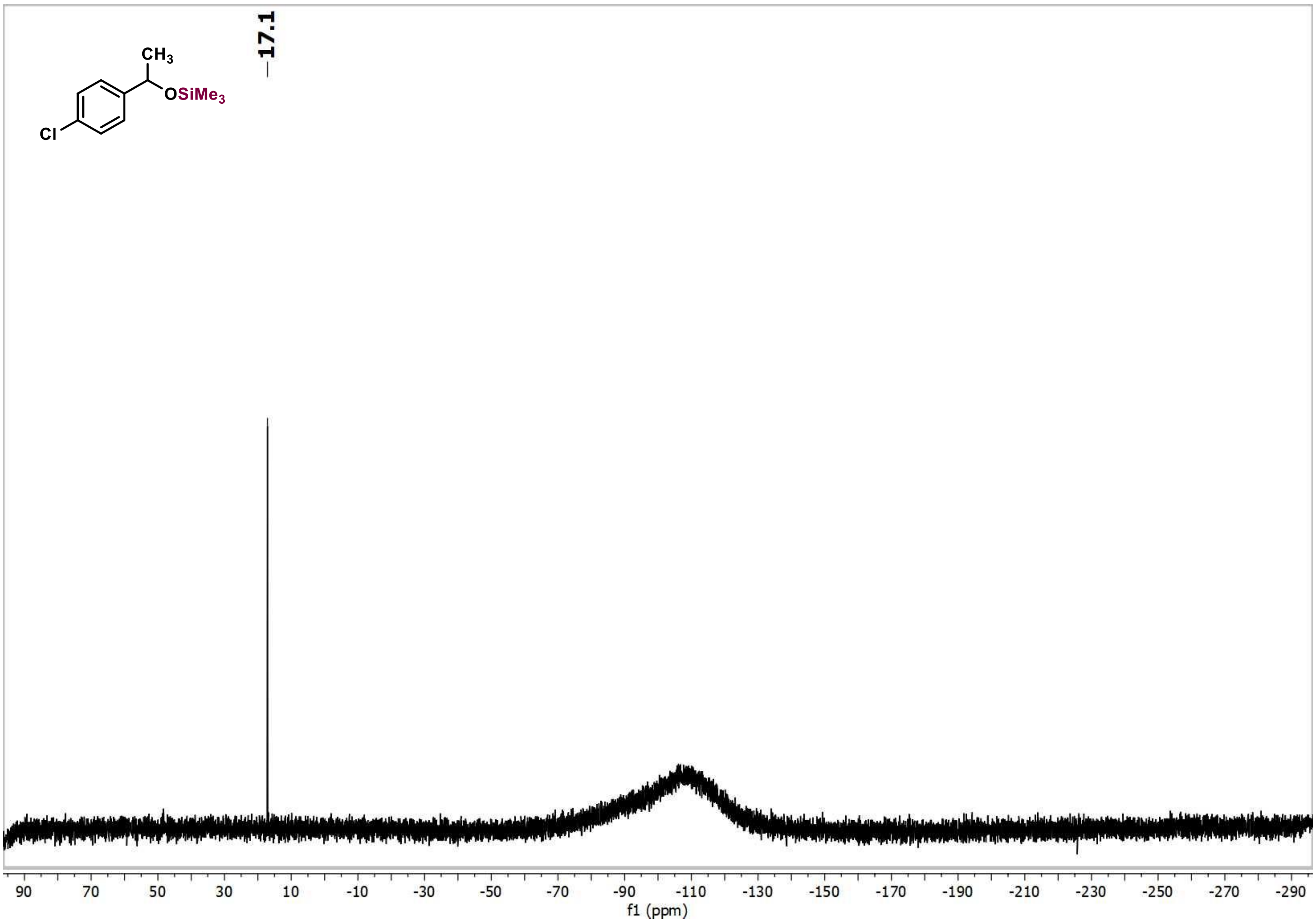

Figure S83. ^{29}Si NMR (79 MHz, Chloroform-d, 25°C) of (1-(4-chlorophenyl)ethoxy)trimethylsilane (**3z**).

(1-(4-Bromophenyl)ethoxy)trimethylsilane (**3aa**)

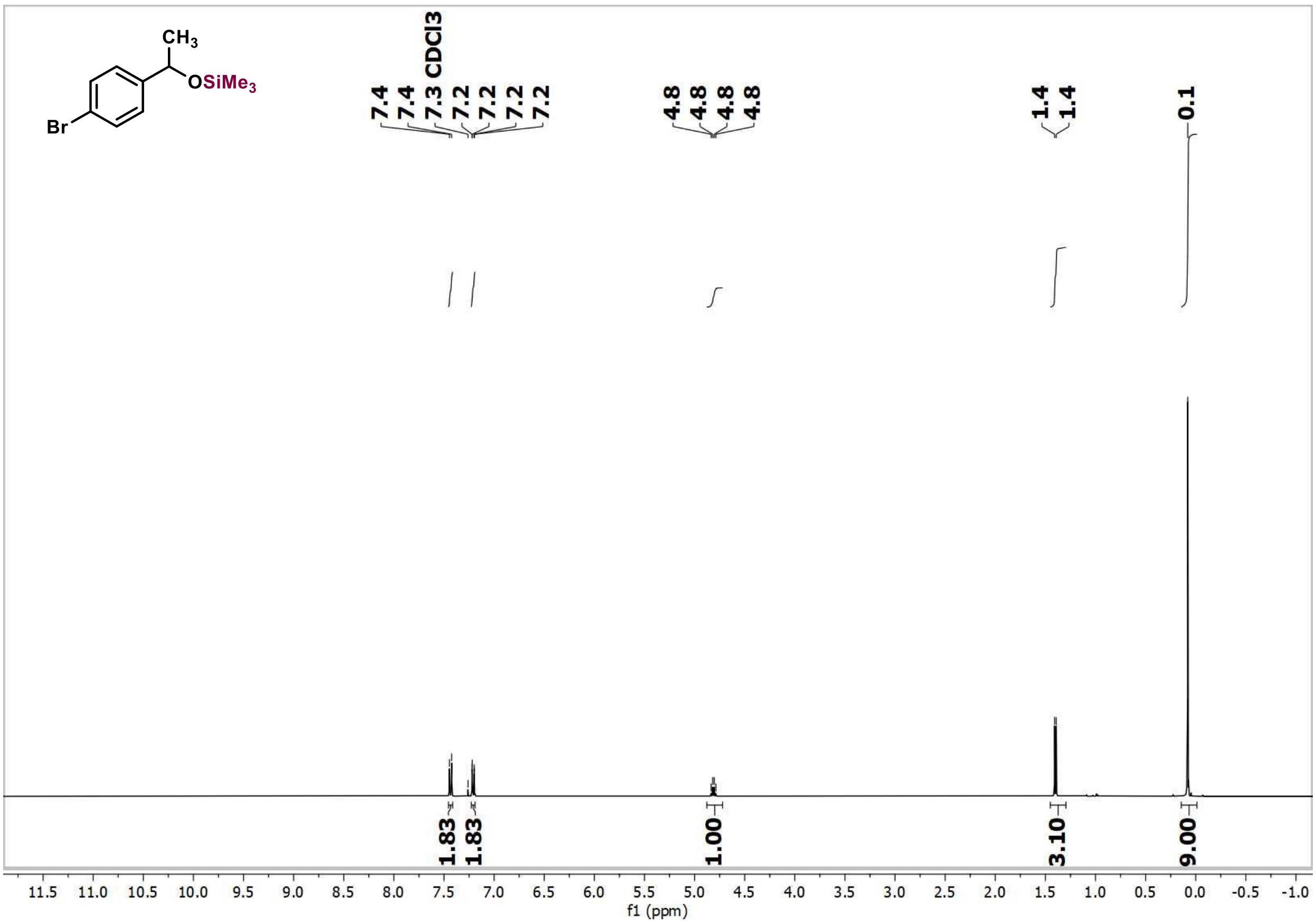

Figure S84. ^{1}H NMR (400 MHz, Chloroform-d, 25°C) of (1-(4-bromophenyl)ethoxy)trimethylsilane (**3aa**).

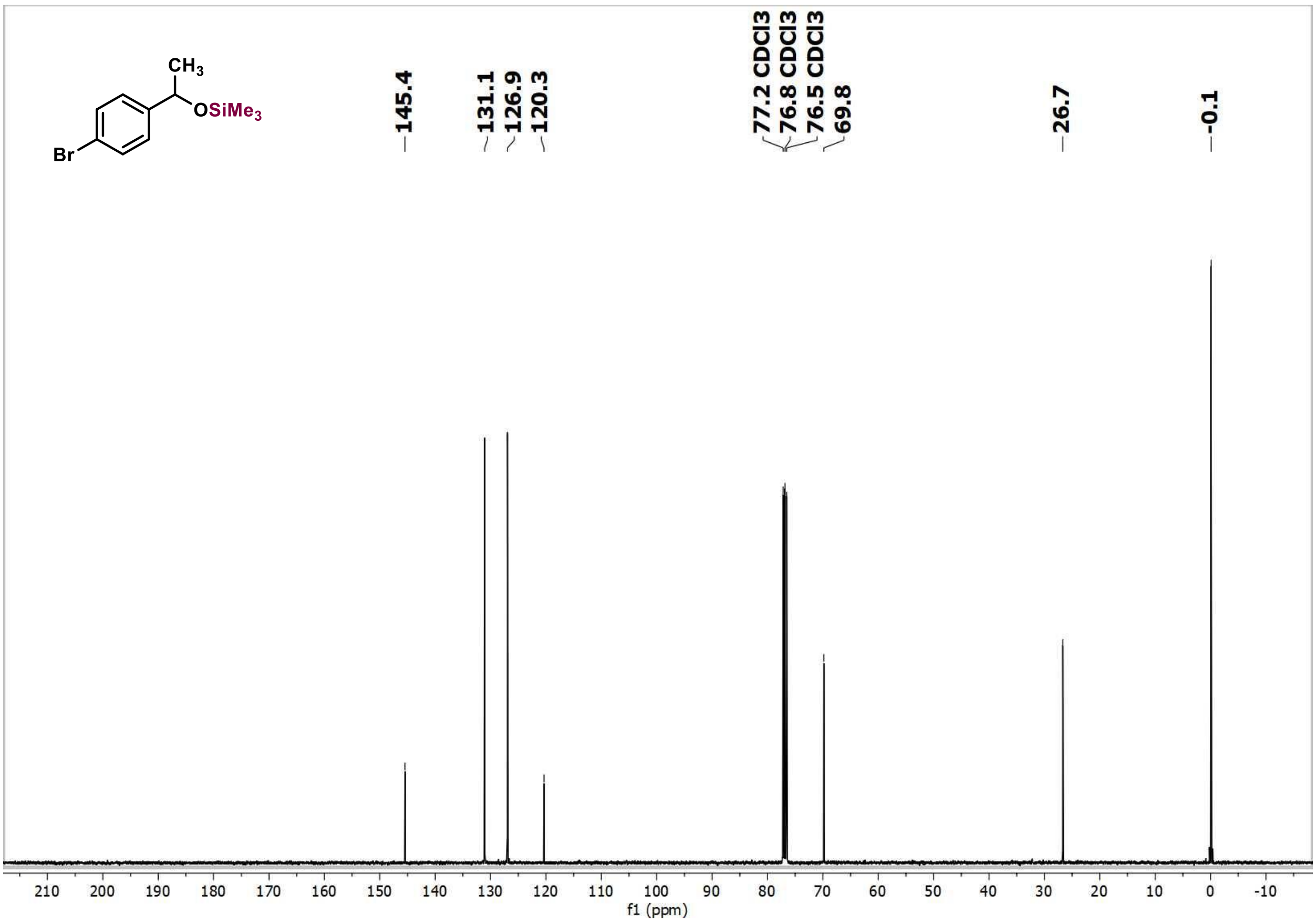

Figure S85. ^{13}C NMR (101 MHz, Chloroform-d, 25°C) of (1-(4-bromophenyl)ethoxy)trimethylsilane (**3aa**).

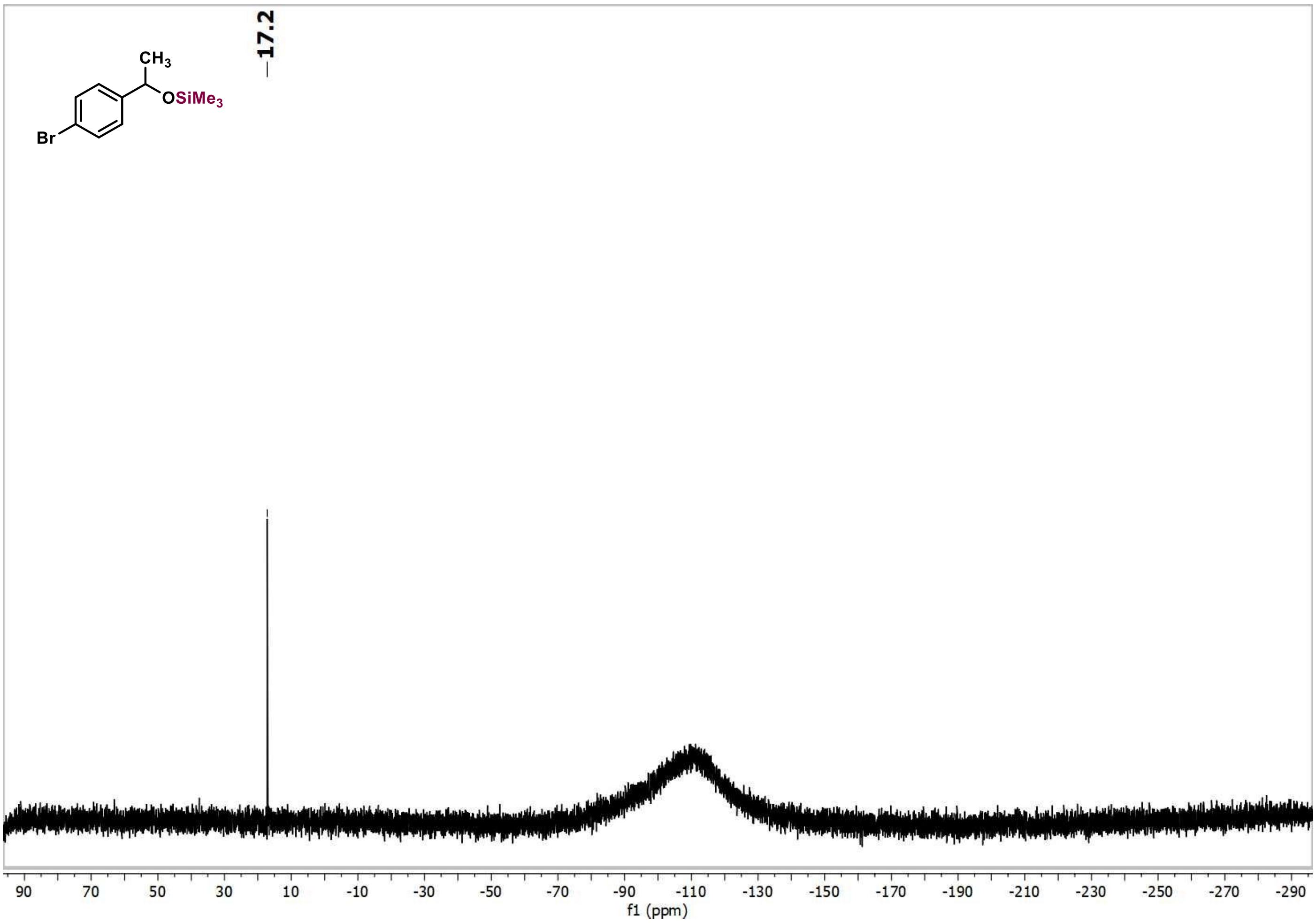

Figure S86. ^{29}Si NMR (79 MHz, Chloroform-d, 25°C) of (1-(4-bromophenyl)ethoxy)trimethylsilane (**3aa**).

1-Trimethylsiloxyadamantane (**3ab**)

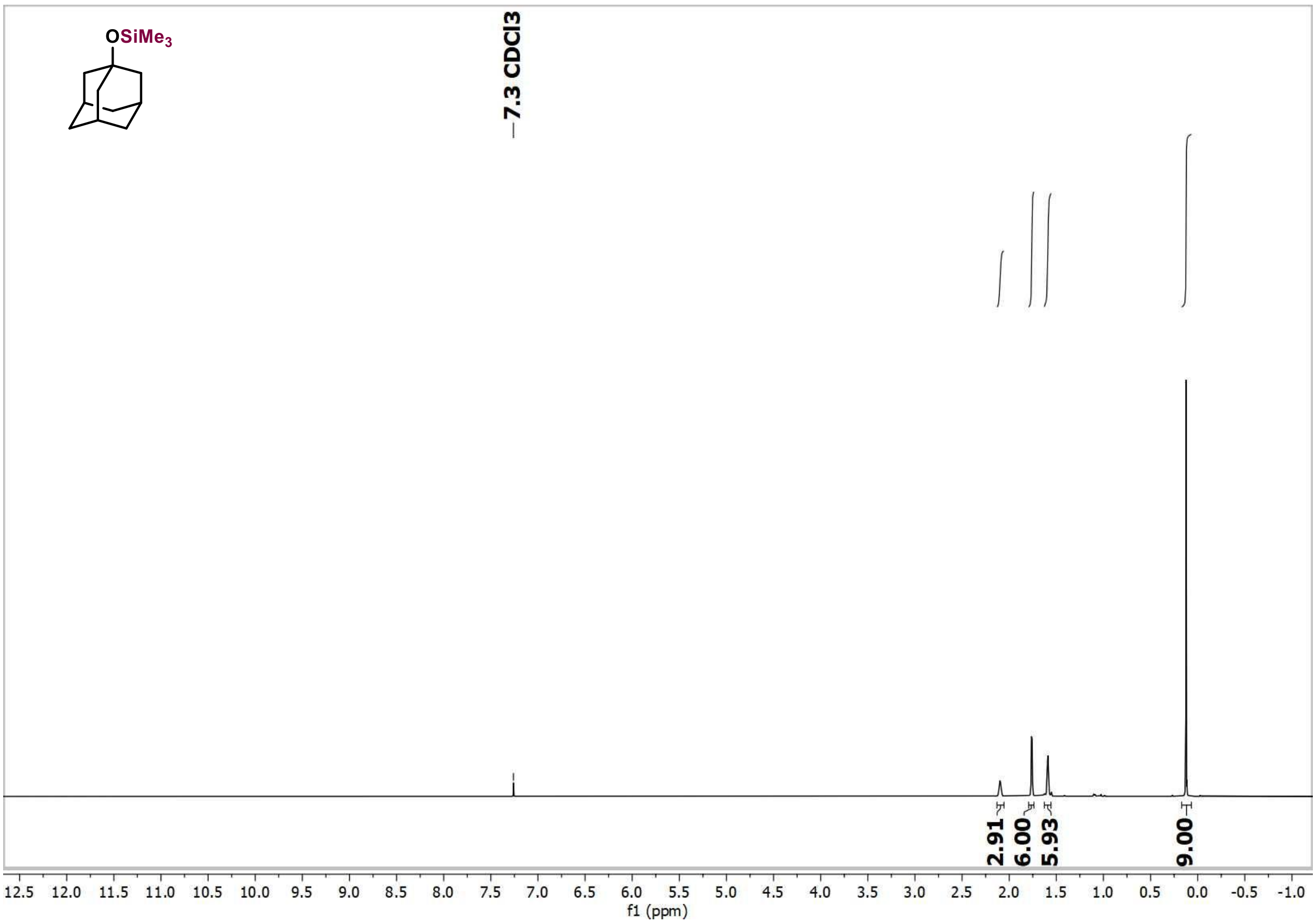

Figure S87. ^{1}H NMR (400 MHz, Chloroform-d, 25°C) of 1-trimethylsiloxyadamantane (**3ab**).

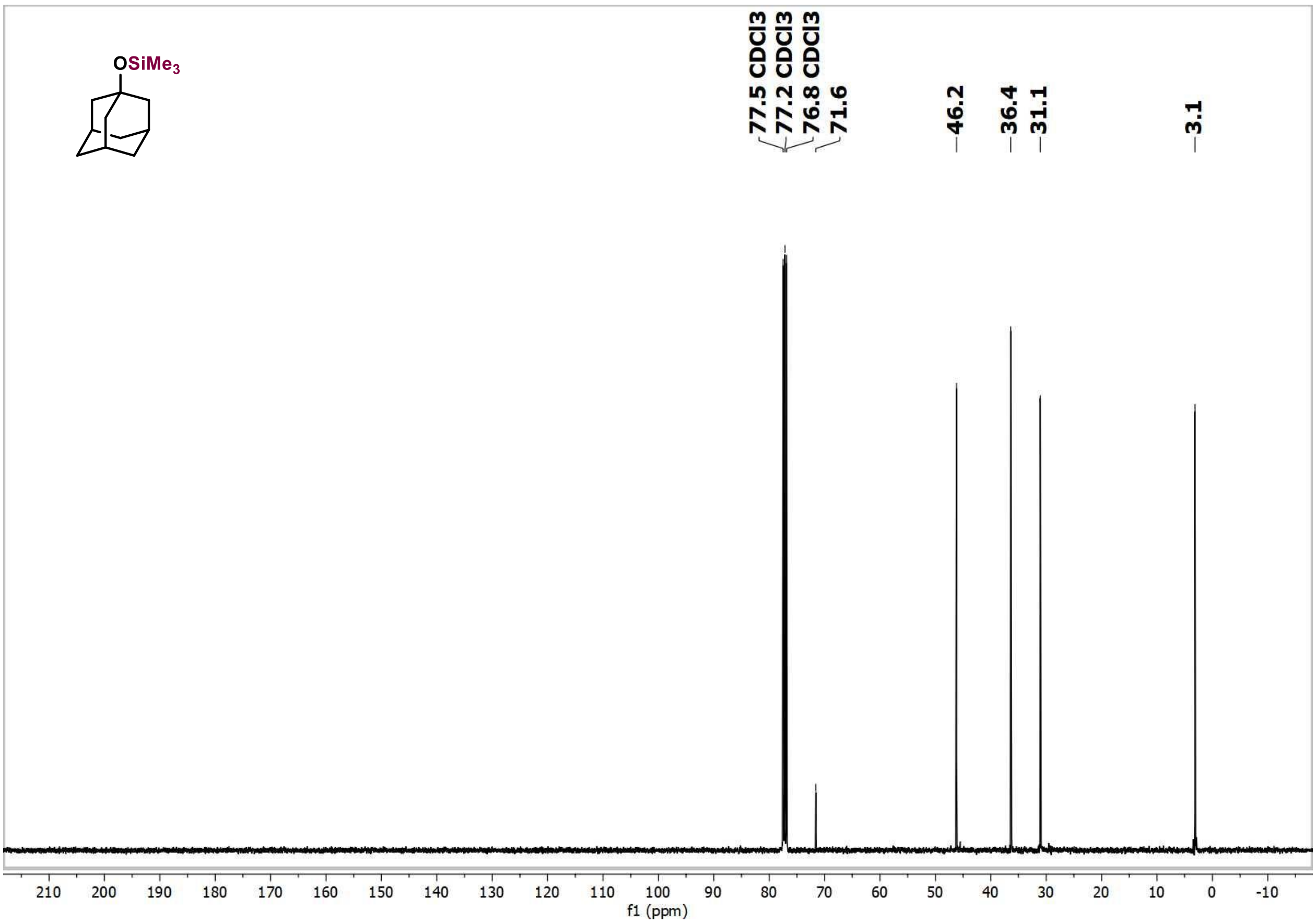

Figure S88. ^{13}C NMR (101 MHz, Chloroform-d, 25°C) of 1-trimethylsiloxyadamantane (**3ab**).

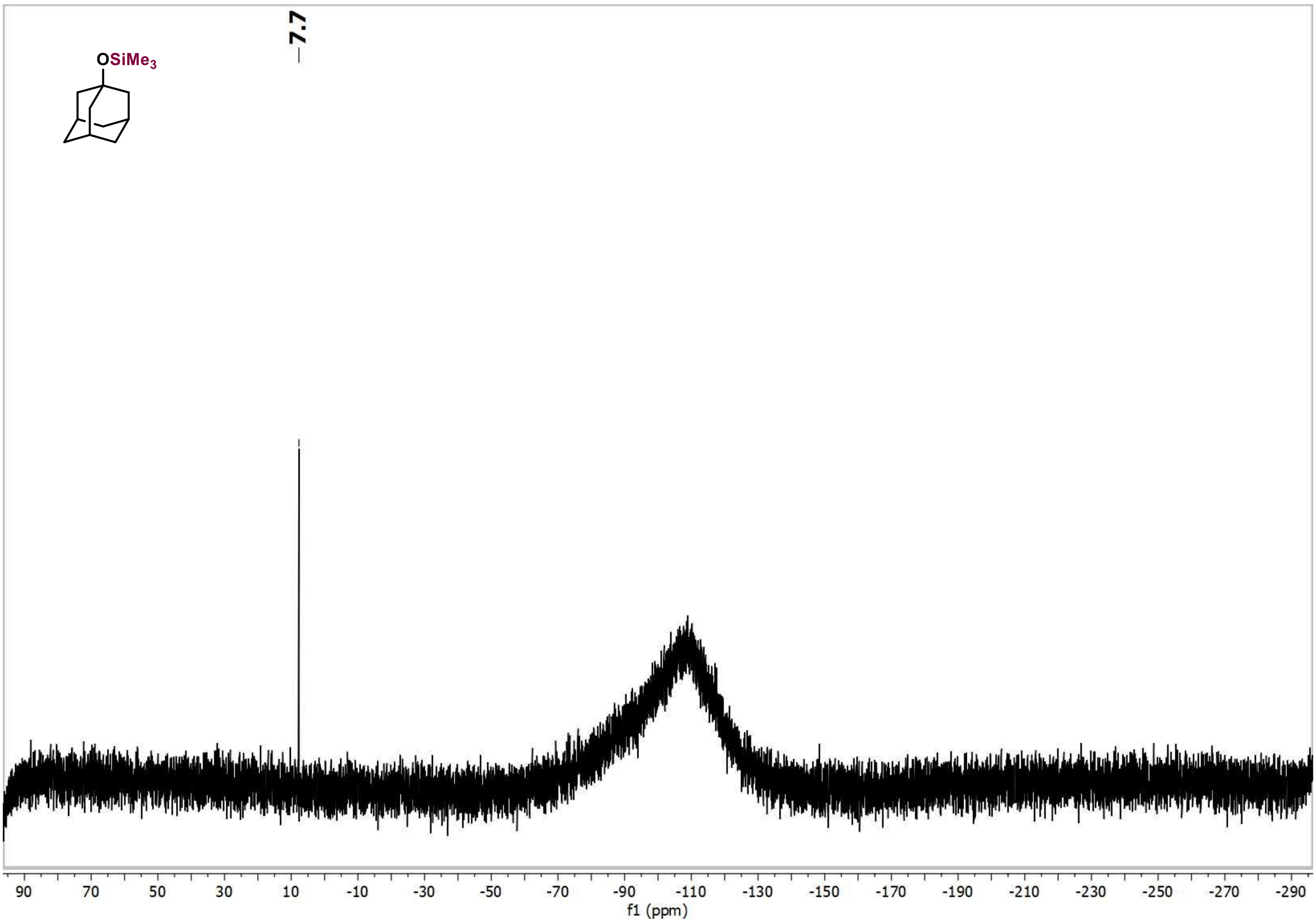

Figure S89. ^{29}Si NMR (79 MHz, Chloroform-d, 25°C) of 1-trimethylsiloxyadamantane (**3ab**).

Bis(trimethylsilyl) 4-methylolphenol (**3ac**)

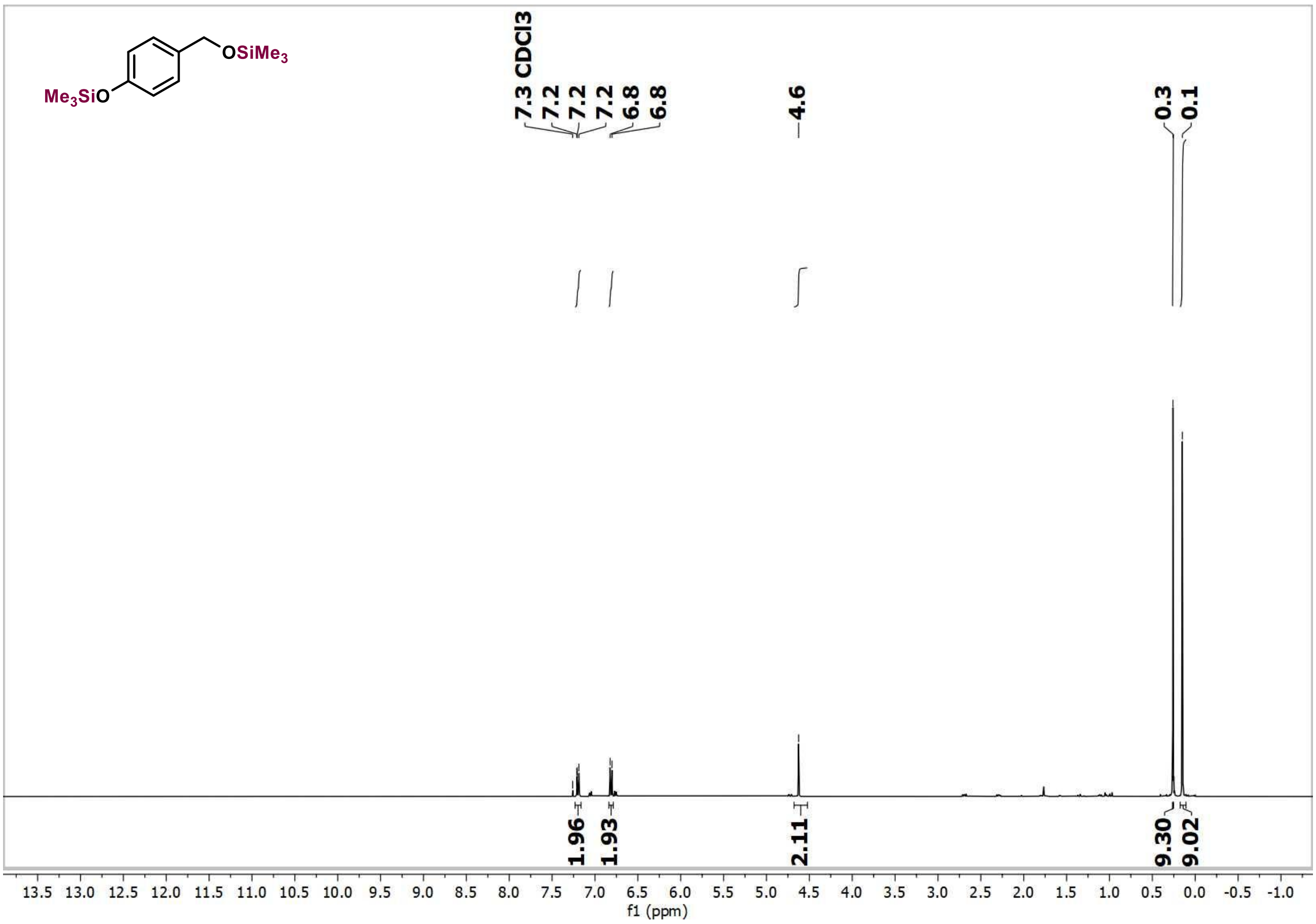

Figure S90. ^{1}H NMR (400 MHz, Chloroform-d, 25°C) of bis(trimethylsilyl) 4-methylolphenol (**3ac**).

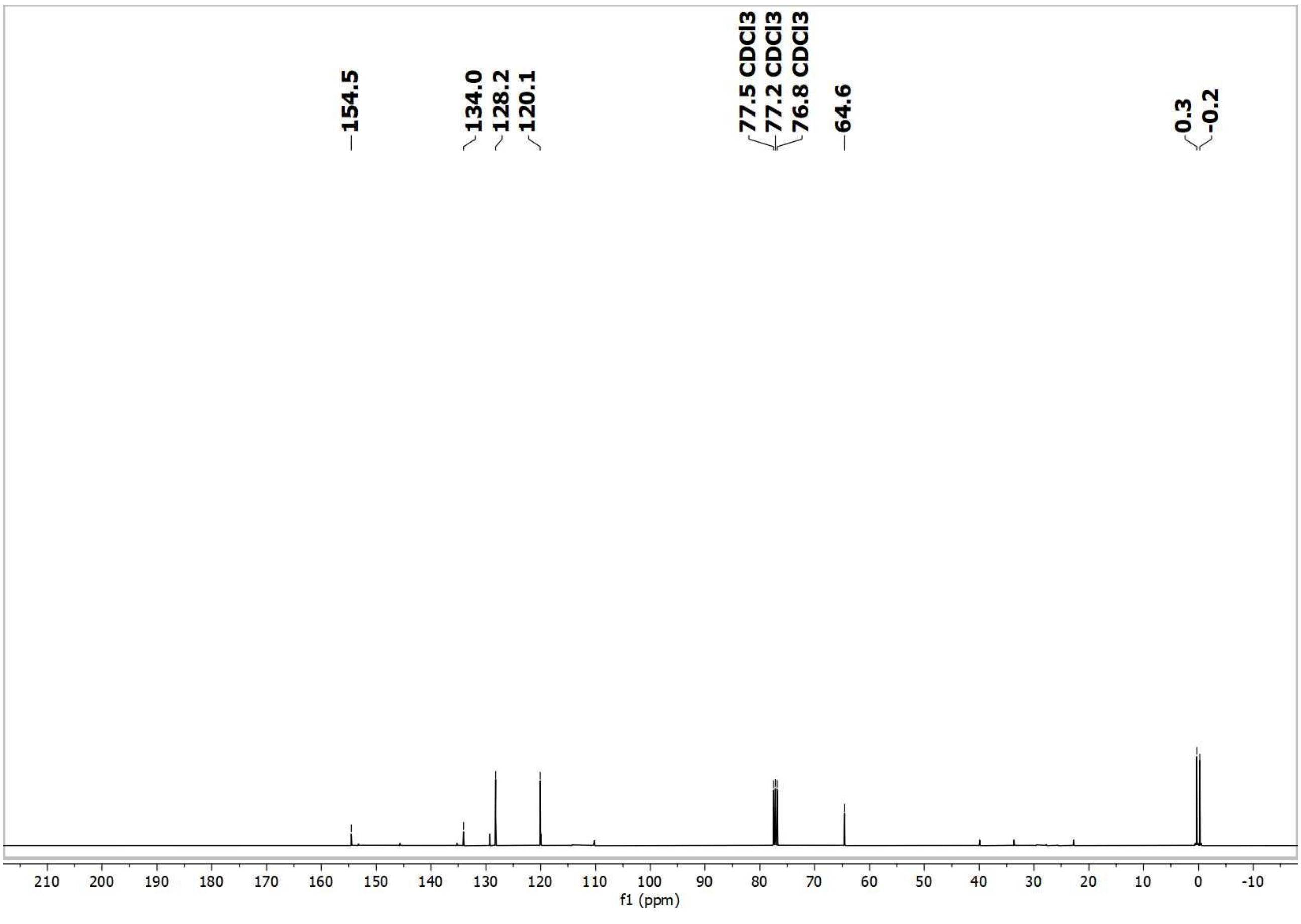

Figure S91. ^{13}C NMR (101 MHz, Chloroform-d, 25°C) of bis(trimethylsilyl) 4-methylolphenol (**3ac**).

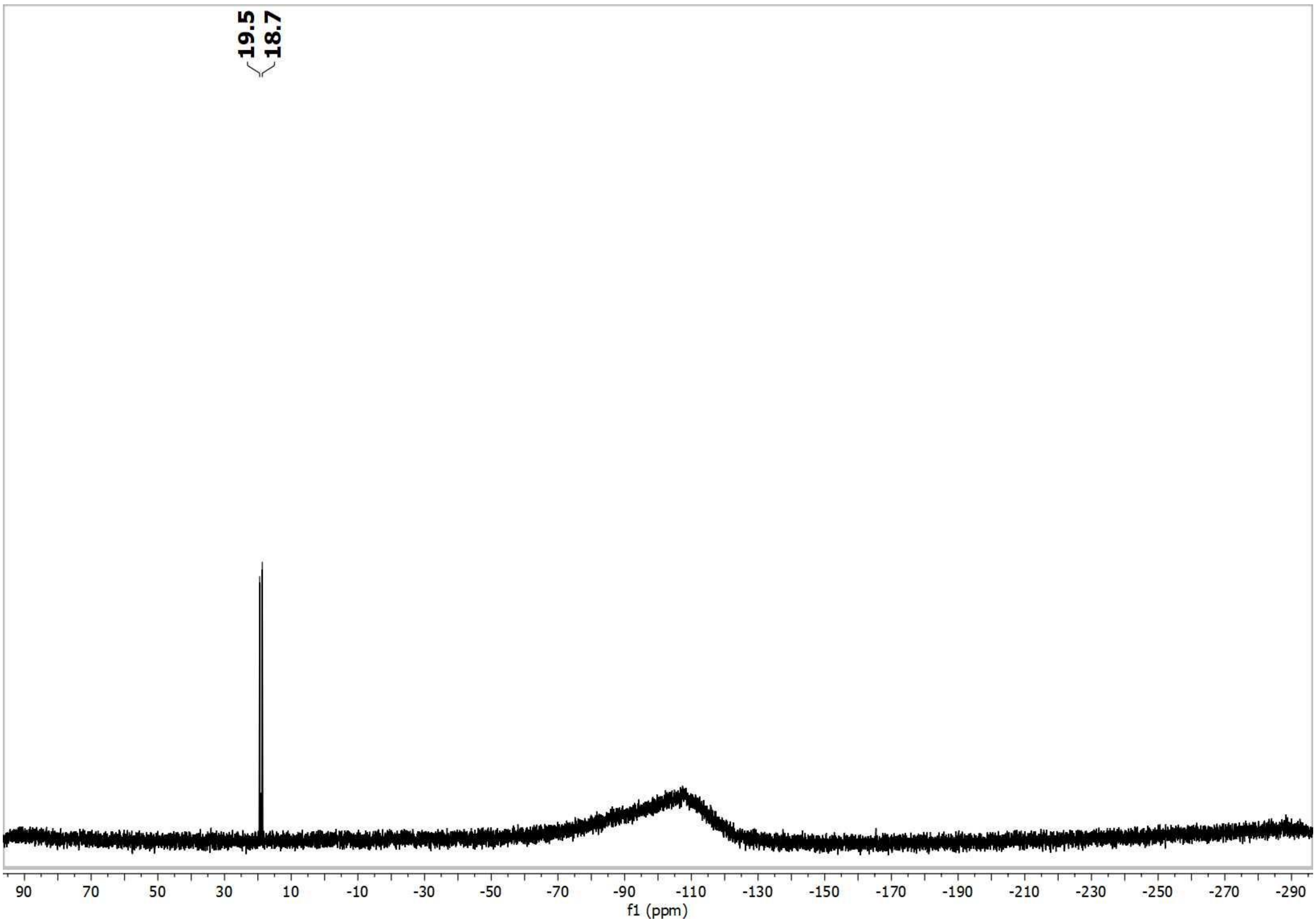

Figure S92. ^{29}Si NMR (79 MHz, Chloroform-d, 25°C) of bis(trimethylsilyl) 4-methylolphenol (**3ac**).

(2,6-Dimethylphenoxy)trimethylsilane (**3ad**)

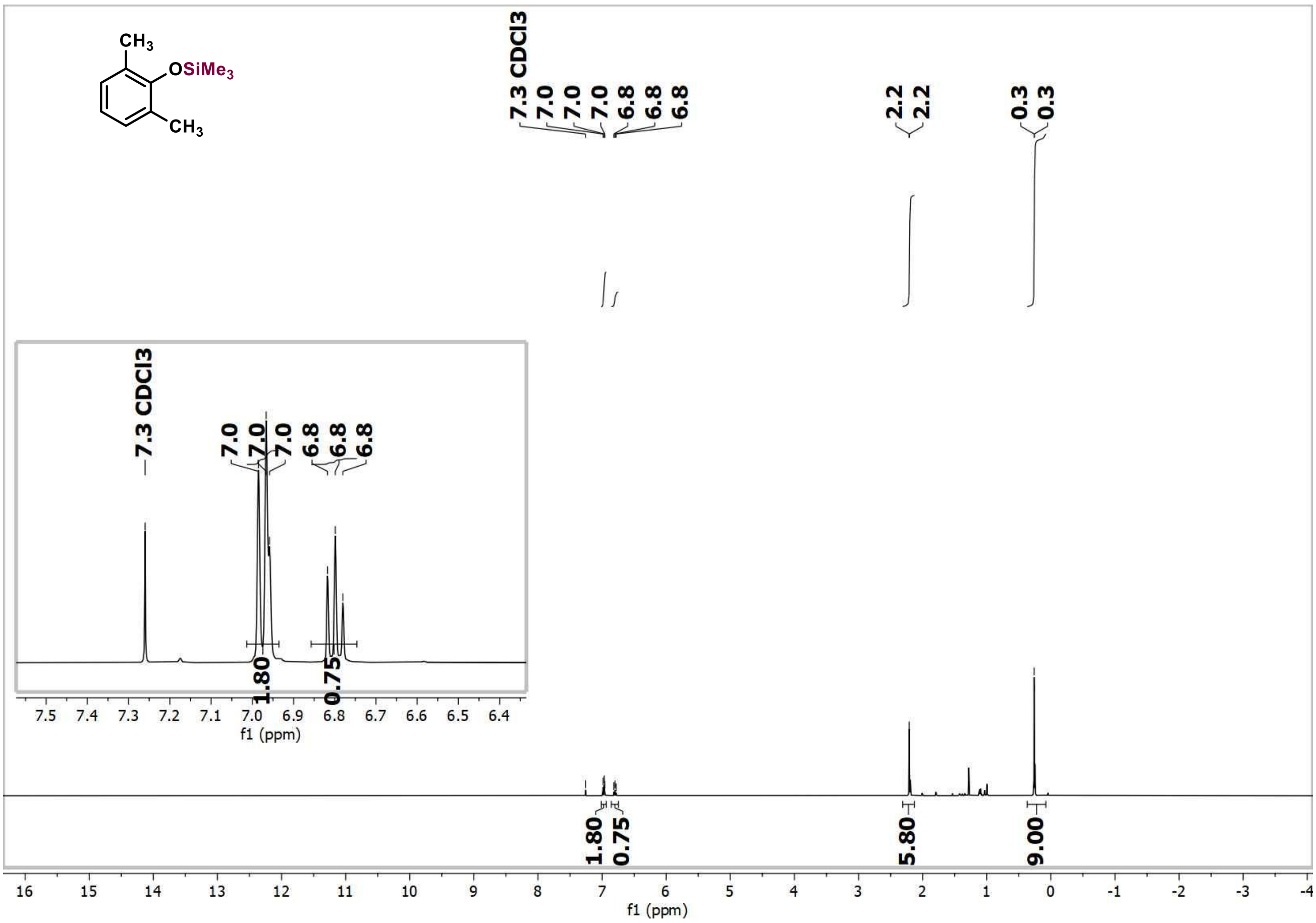

Figure S93. ^{1}H NMR (400 MHz, Chloroform-d, 25°C) of (2,6-dimethylphenoxy)trimethylsilane (**3ad**).

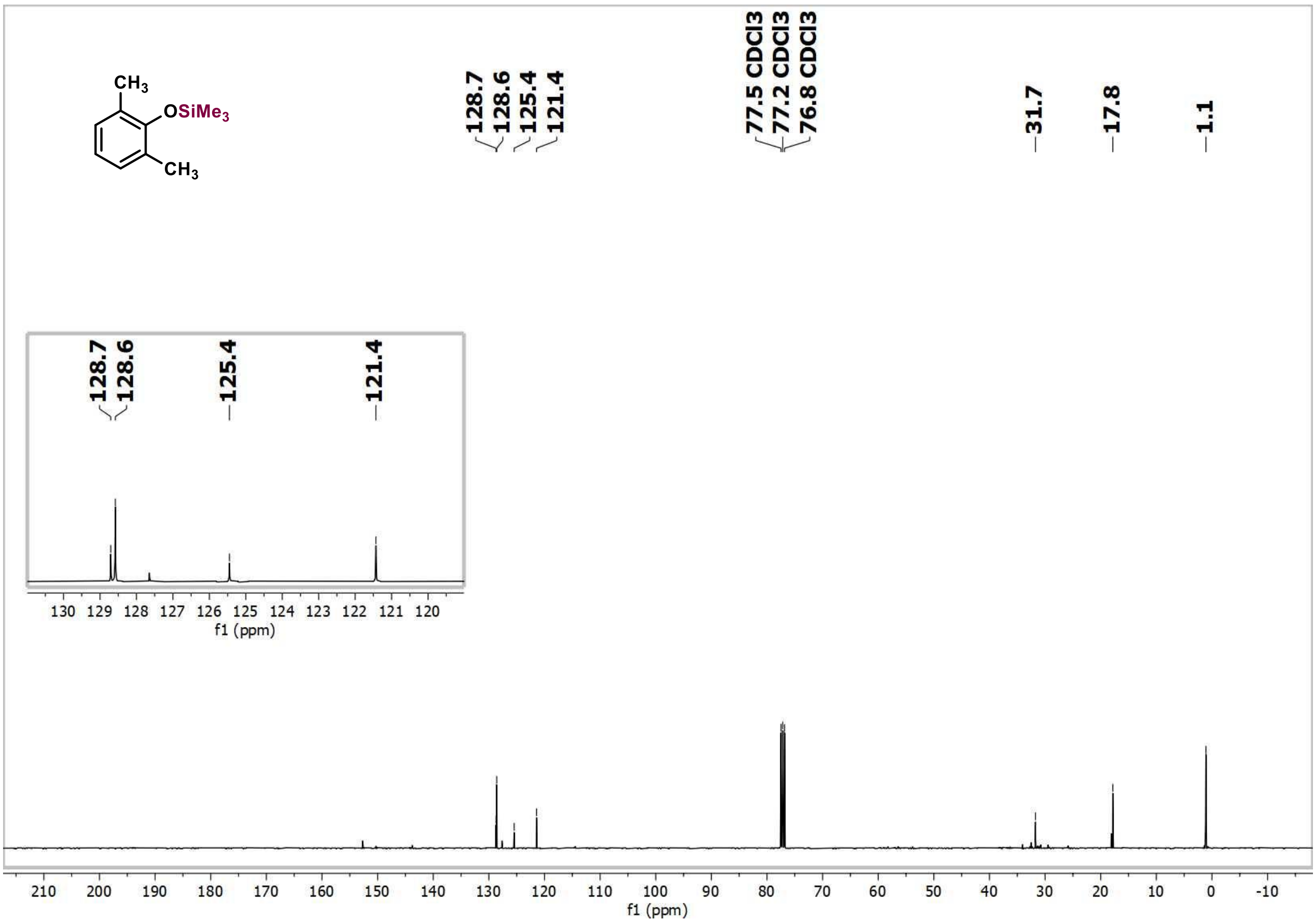

Figure S94. ^{13}C NMR (101 MHz, Chloroform-d, 25°C) of (2,6-dimethylphenoxy)trimethylsilane (**3ad**).

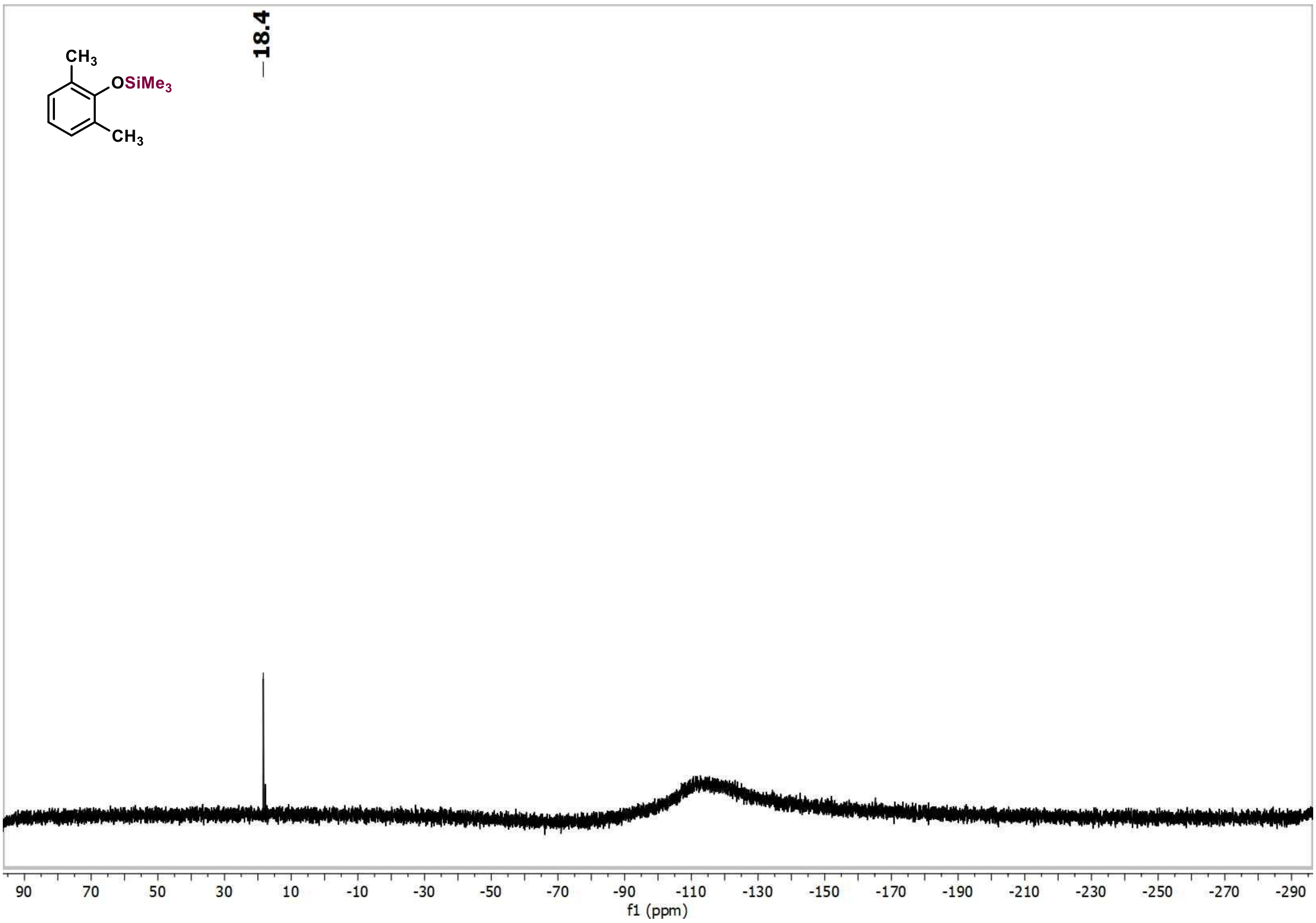

Figure S95. ^{29}Si NMR (79 MHz, Chloroform-d, 25°C) of (2,6-dimethylphenoxy)trimethylsilane (**3ad**).

4-Chloro-3,5-dimethylphenoxytrimethylsilane (**3ae**)

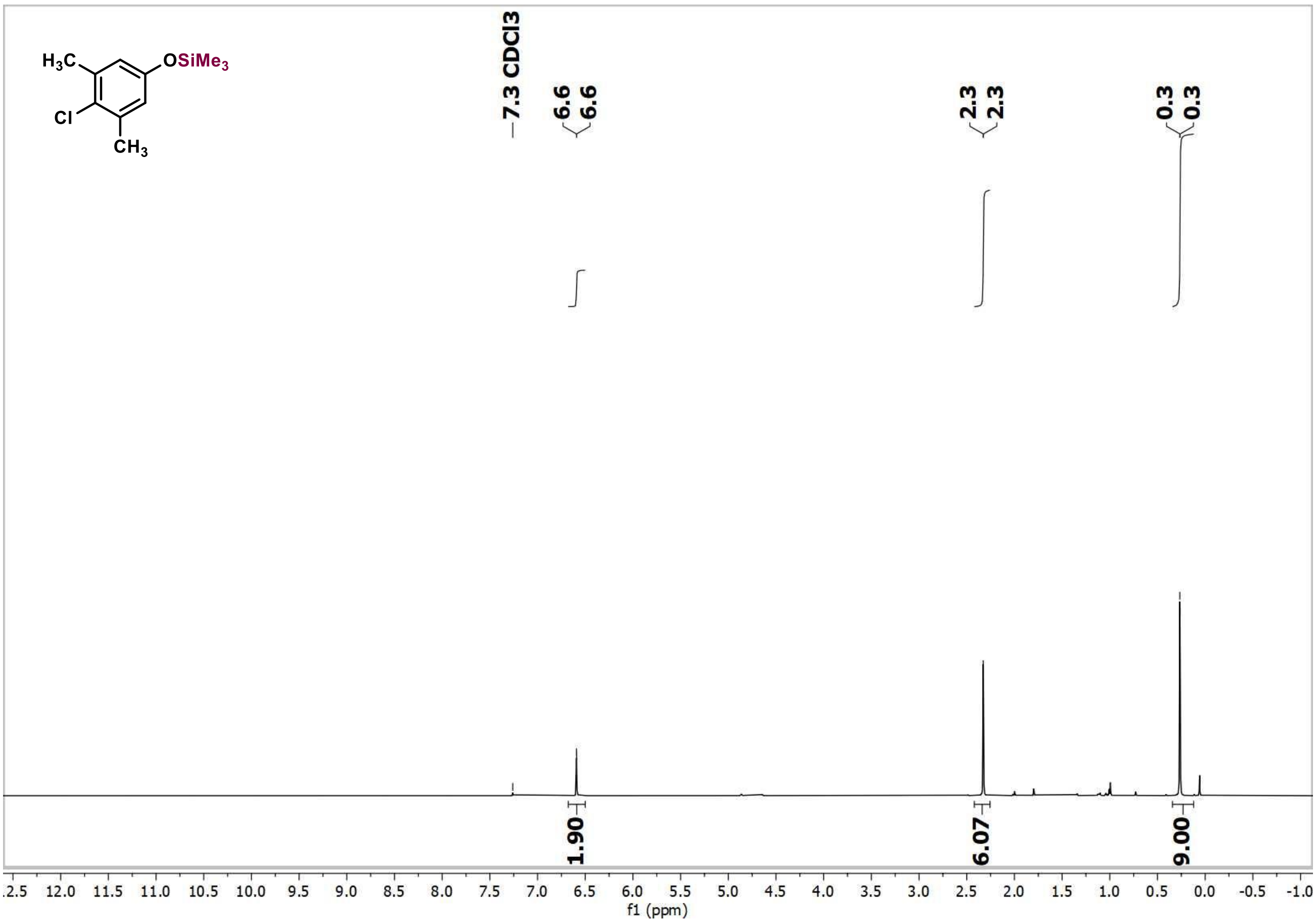

Figure S96. ^{1}H NMR (400 MHz, Chloroform-d, 25°C) of 4-chloro-3,5-dimethylphenoxytrimethylsilane (**3ae**).

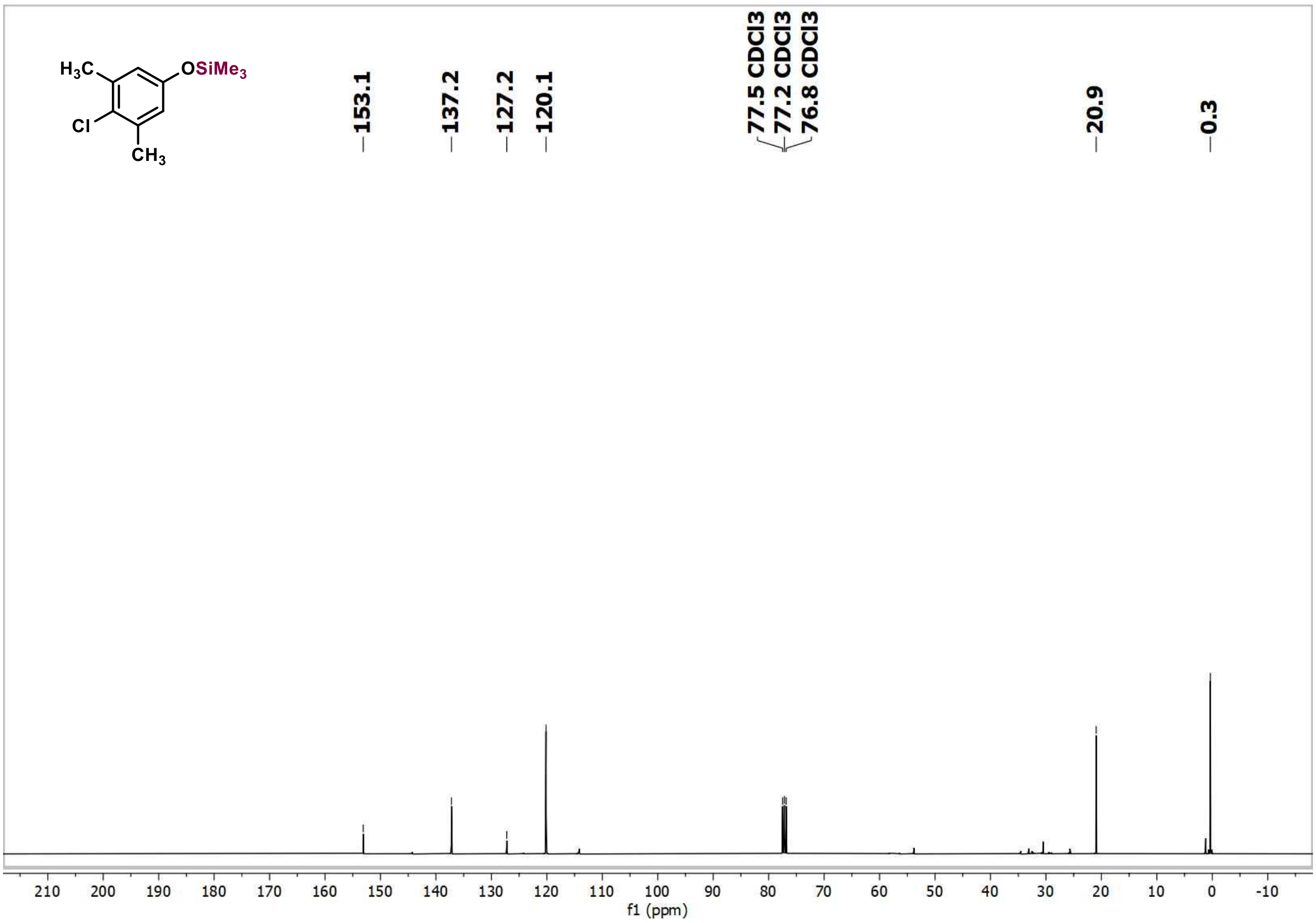

Figure S97. ^{13}C NMR (101 MHz, Chloroform-d, 25°C) of 4-chloro-3,5-dimethylphenoxytrimethylsilane (**3ae**).

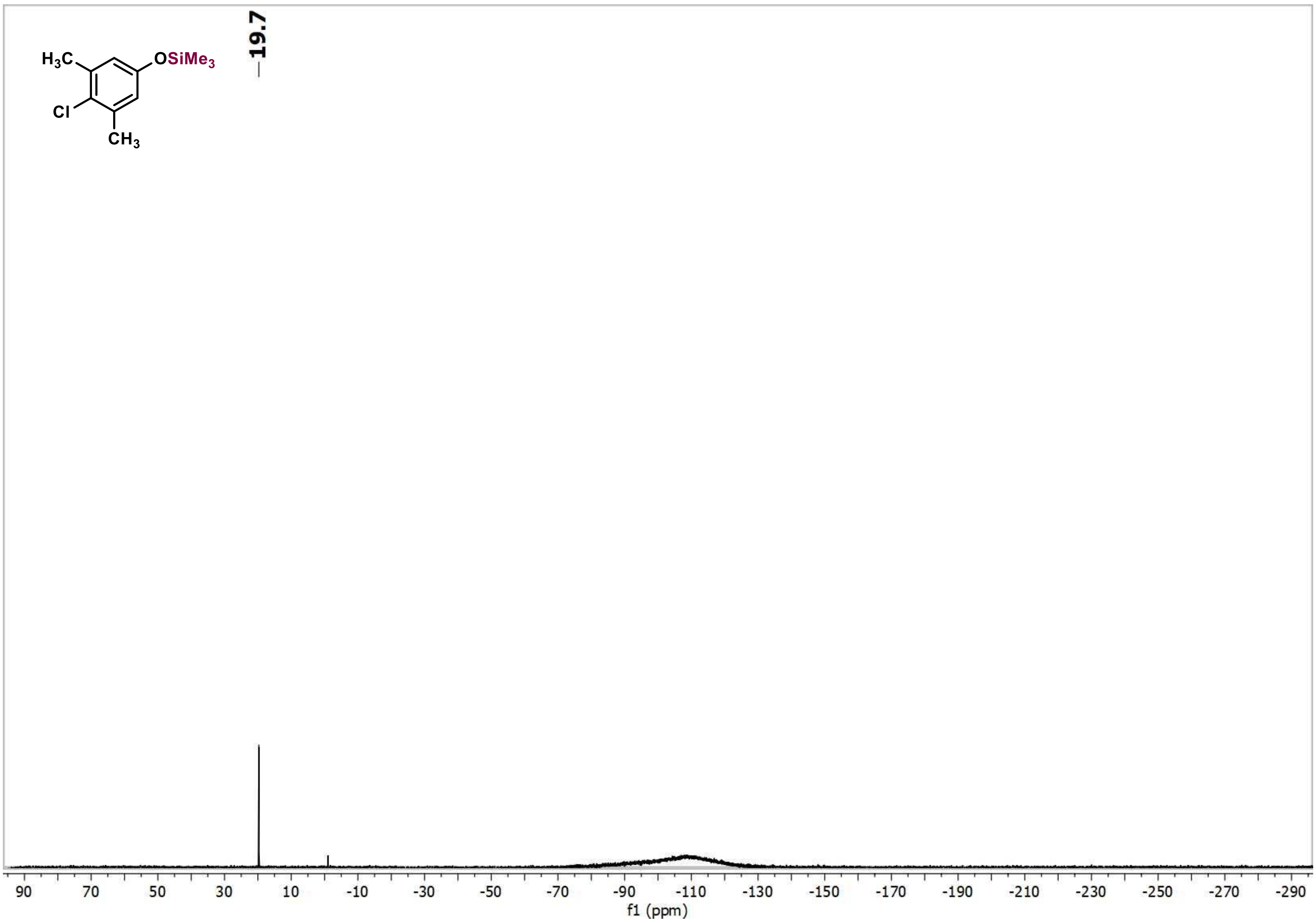

Figure S98. ^{29}Si NMR (79 MHz, Chloroform-d, 25°C) of 4-chloro-3,5-dimethylphenoxytrimethylsilane (**3ae**).

(4-Bromophenoxy)trimethylsilane (**3af**)

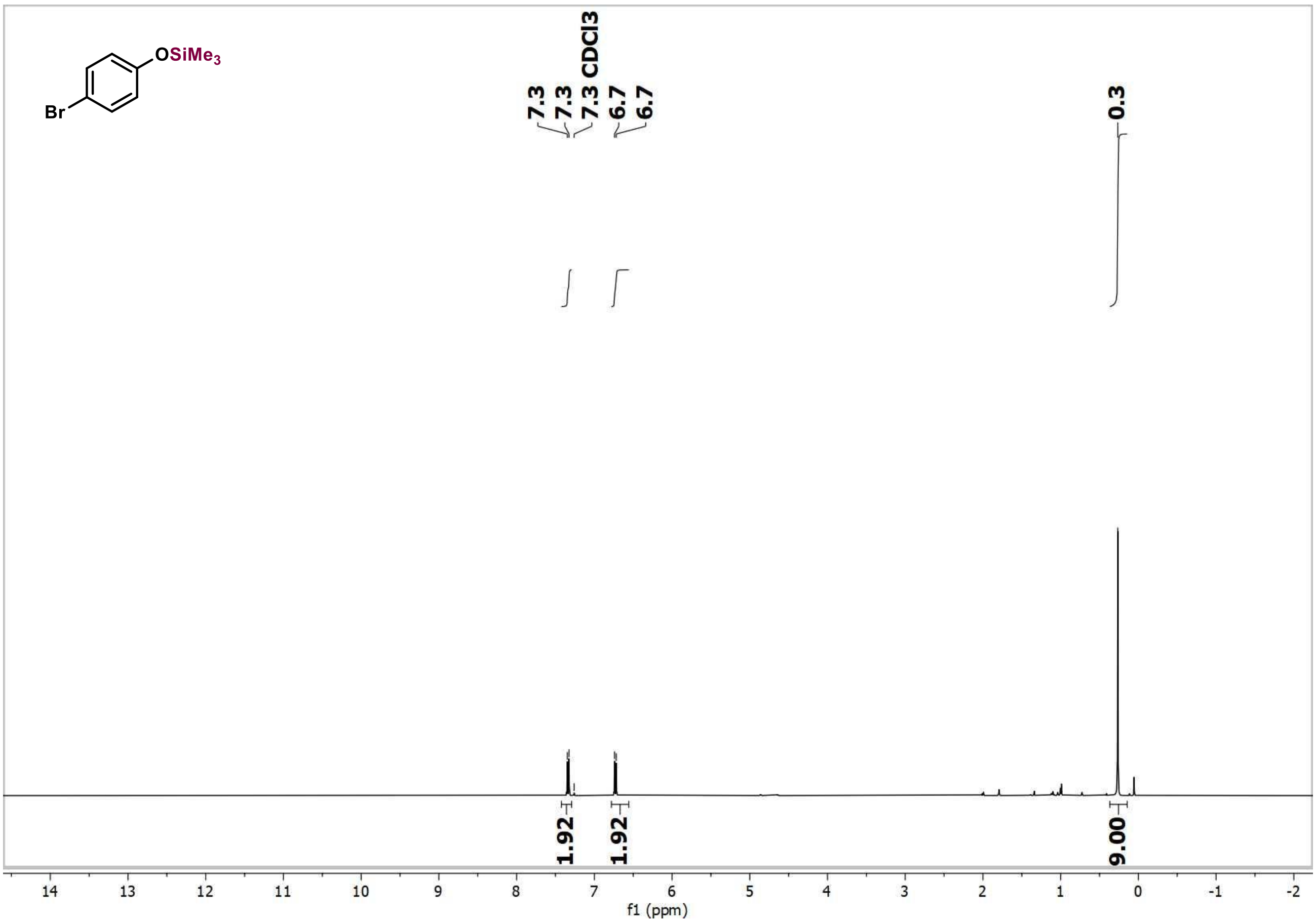

Figure S99. ^{1}H NMR (400 MHz, Chloroform-d, 25°C) of (4-bromophenoxy)trimethylsilane (**3af**).

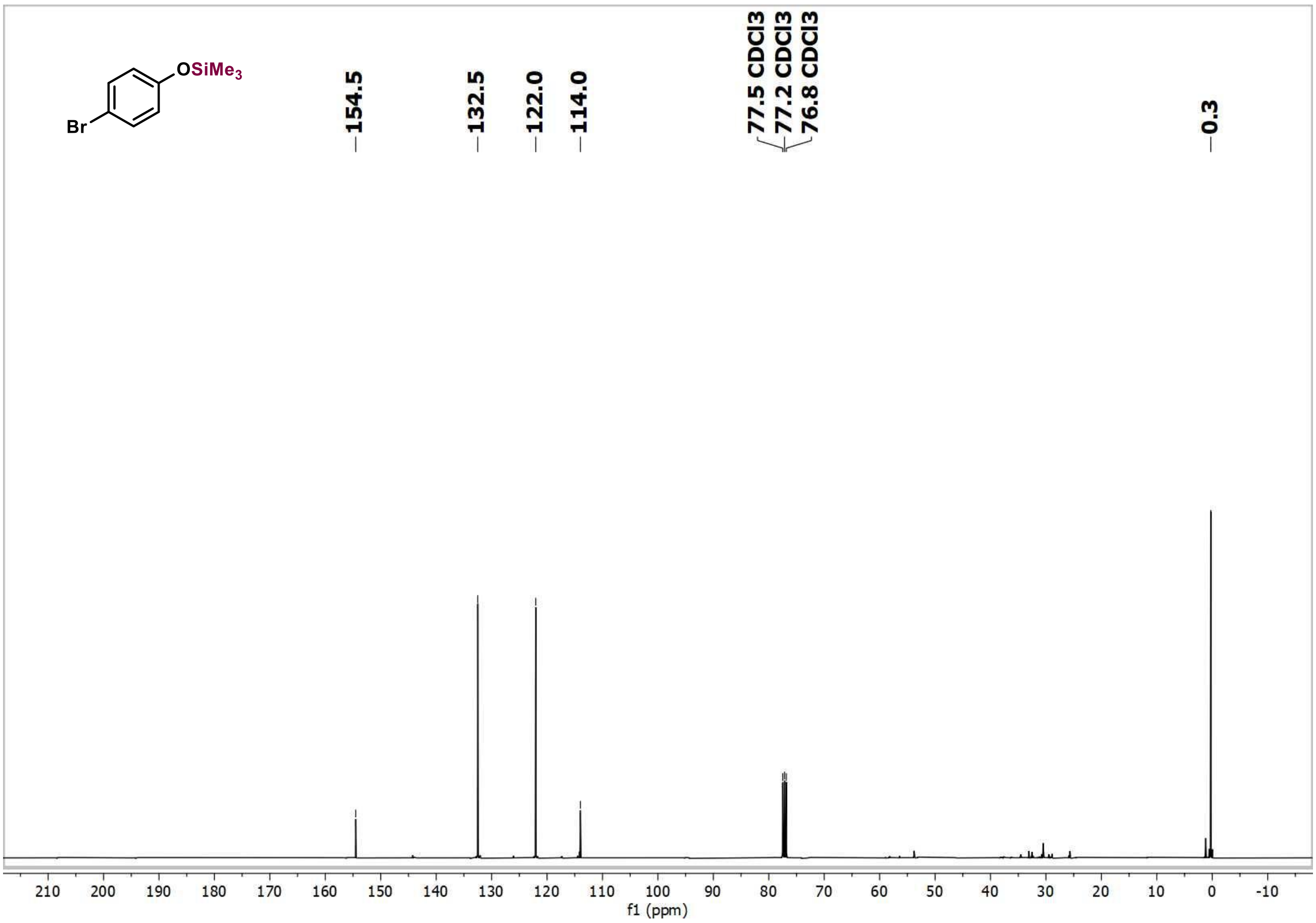

Figure S100. ^{13}C NMR (101 MHz, Chloroform-d, 25°C) of (4-bromophenoxy)trimethylsilane (**3af**).

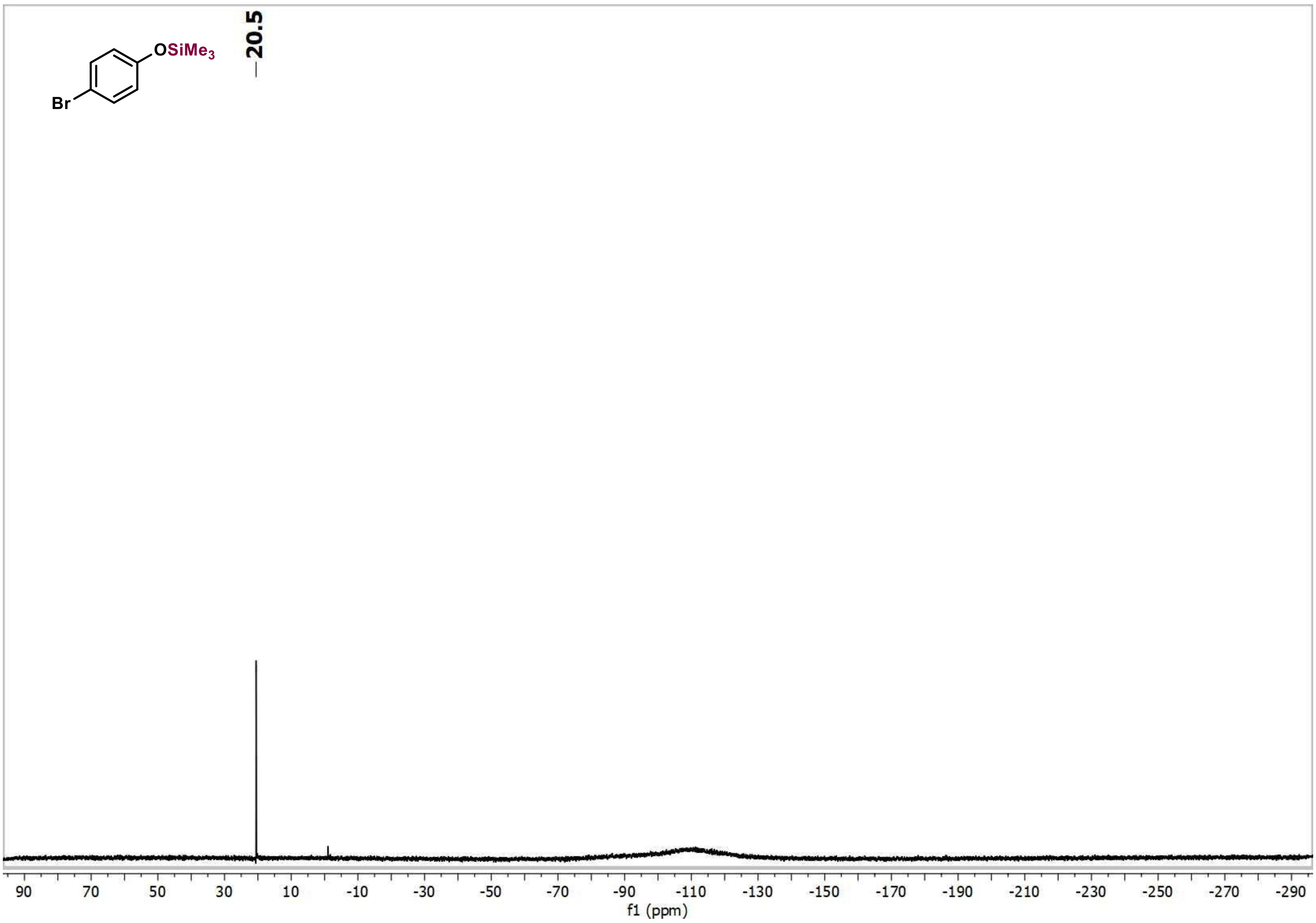

Figure S101. ^{29}Si NMR (79 MHz, Chloroform-d, 25°C) of (4-bromophenoxy)trimethylsilane (**3af**).

((3-Fluorobenzyl)oxy)diisopropylsilane (**3ag**)

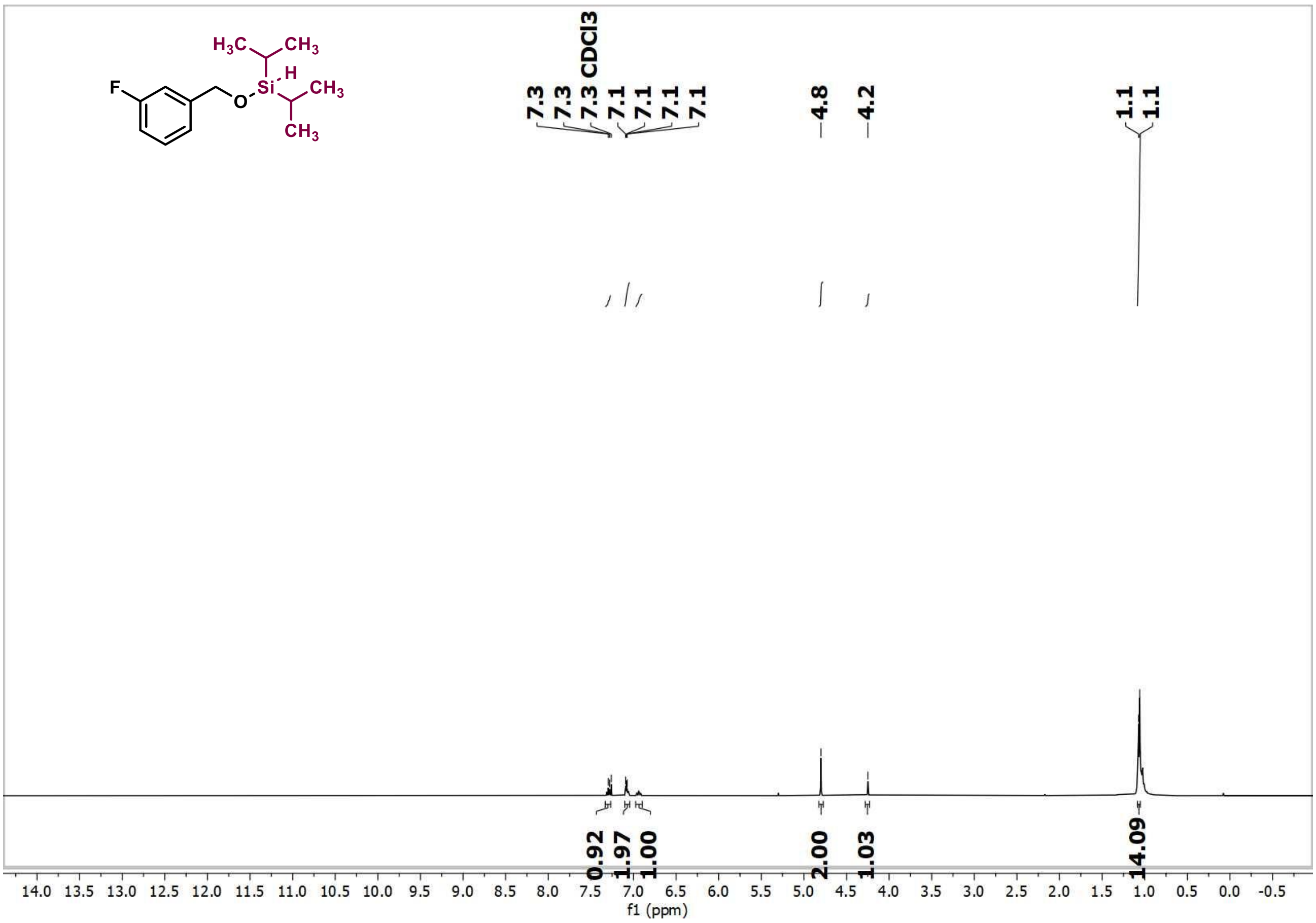

Figure S101. ^{1}H NMR (400 MHz, Chloroform-d, 25°C) of ((3-fluorobenzyl)oxy)diisopropylsilane (**3ag**).

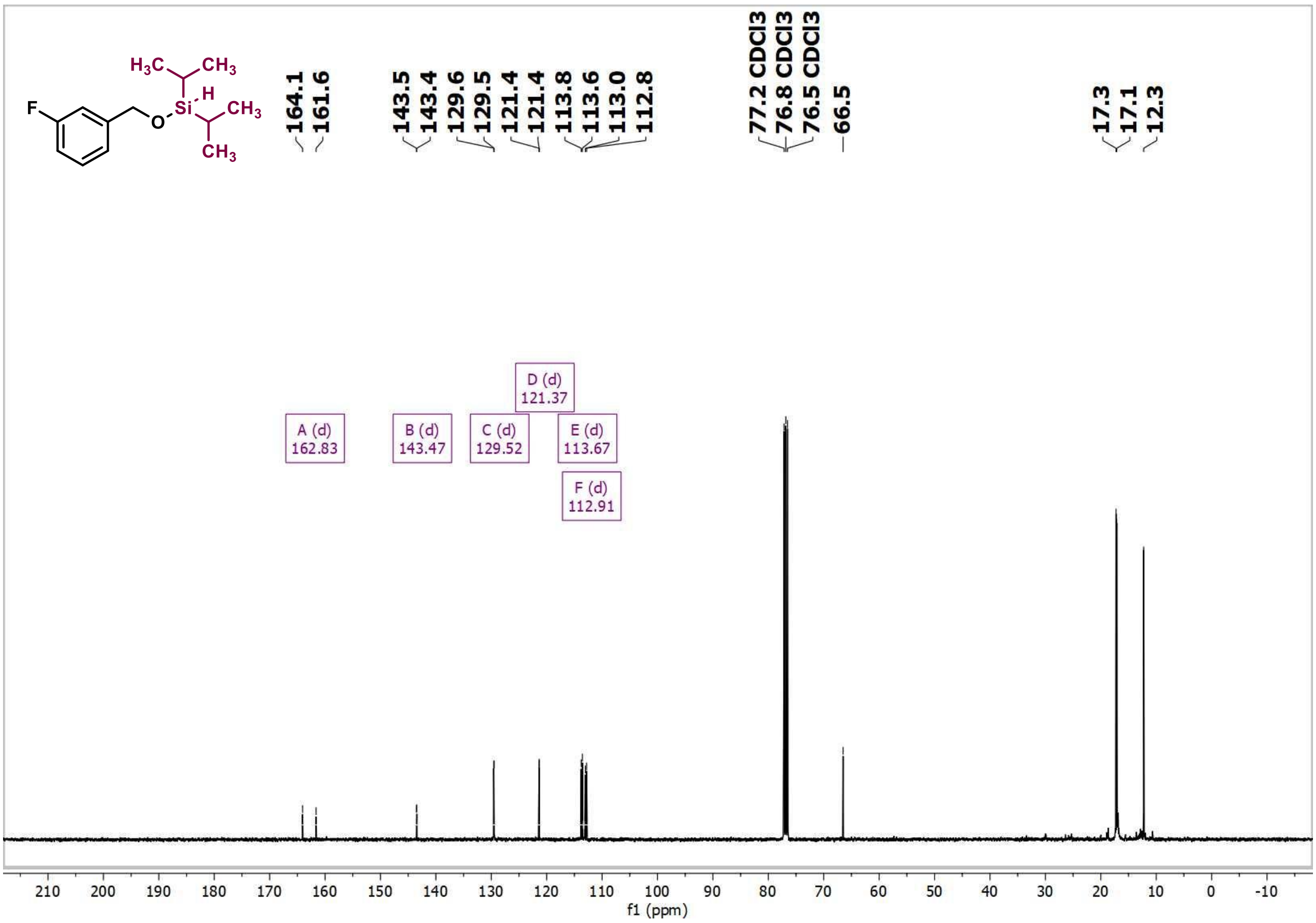

Figure S102. ^{13}C NMR (101 MHz, Chloroform-d, 25°C) of ((3-fluorobenzyl)oxy)diisopropylsilane (**3ag**).

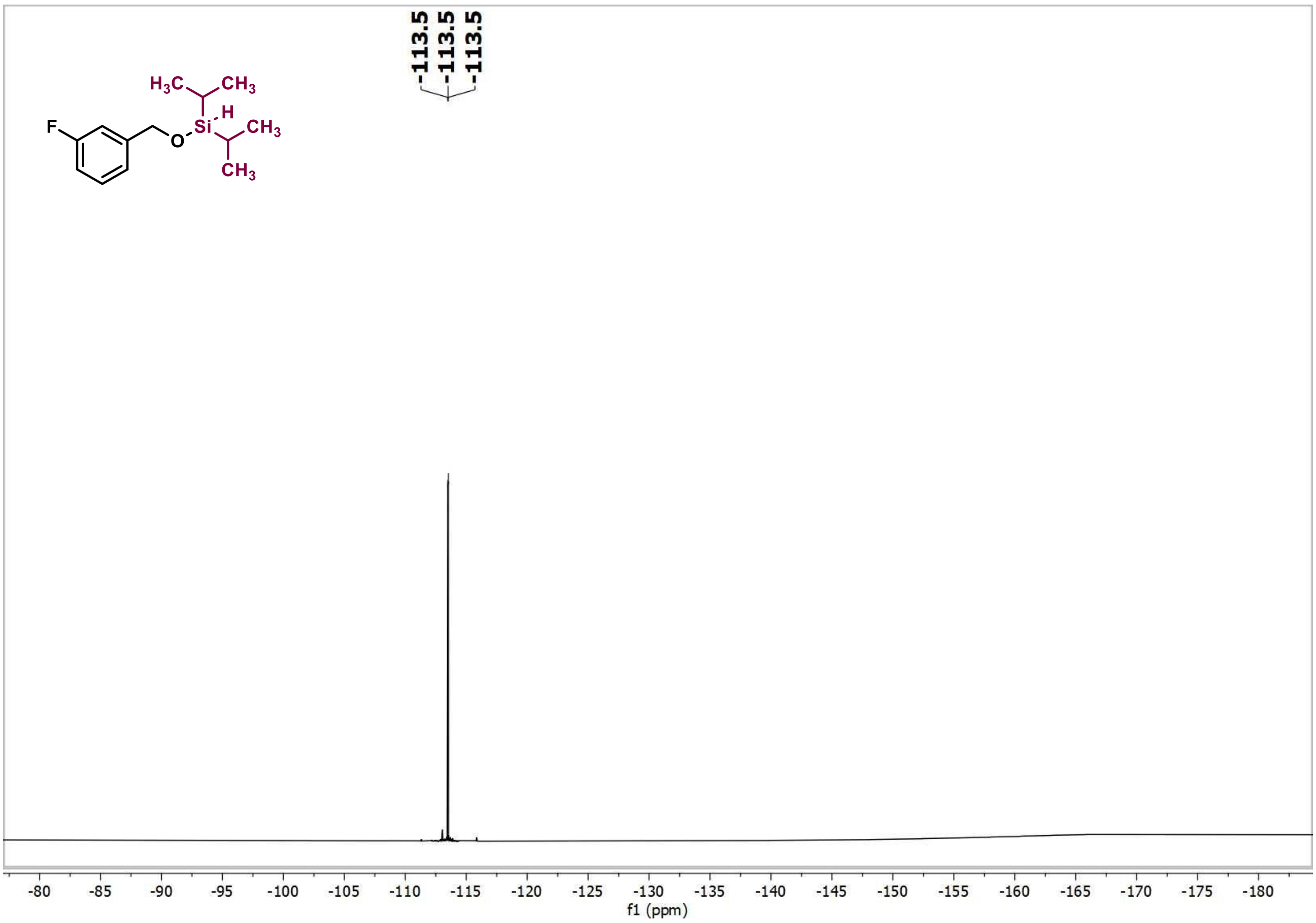

Figure S103. ^{19}F NMR (377 MHz, Chloroform-d, 25°C) of ((3-fluorobenzyl)oxy)diisopropylsilane (**3ag**).

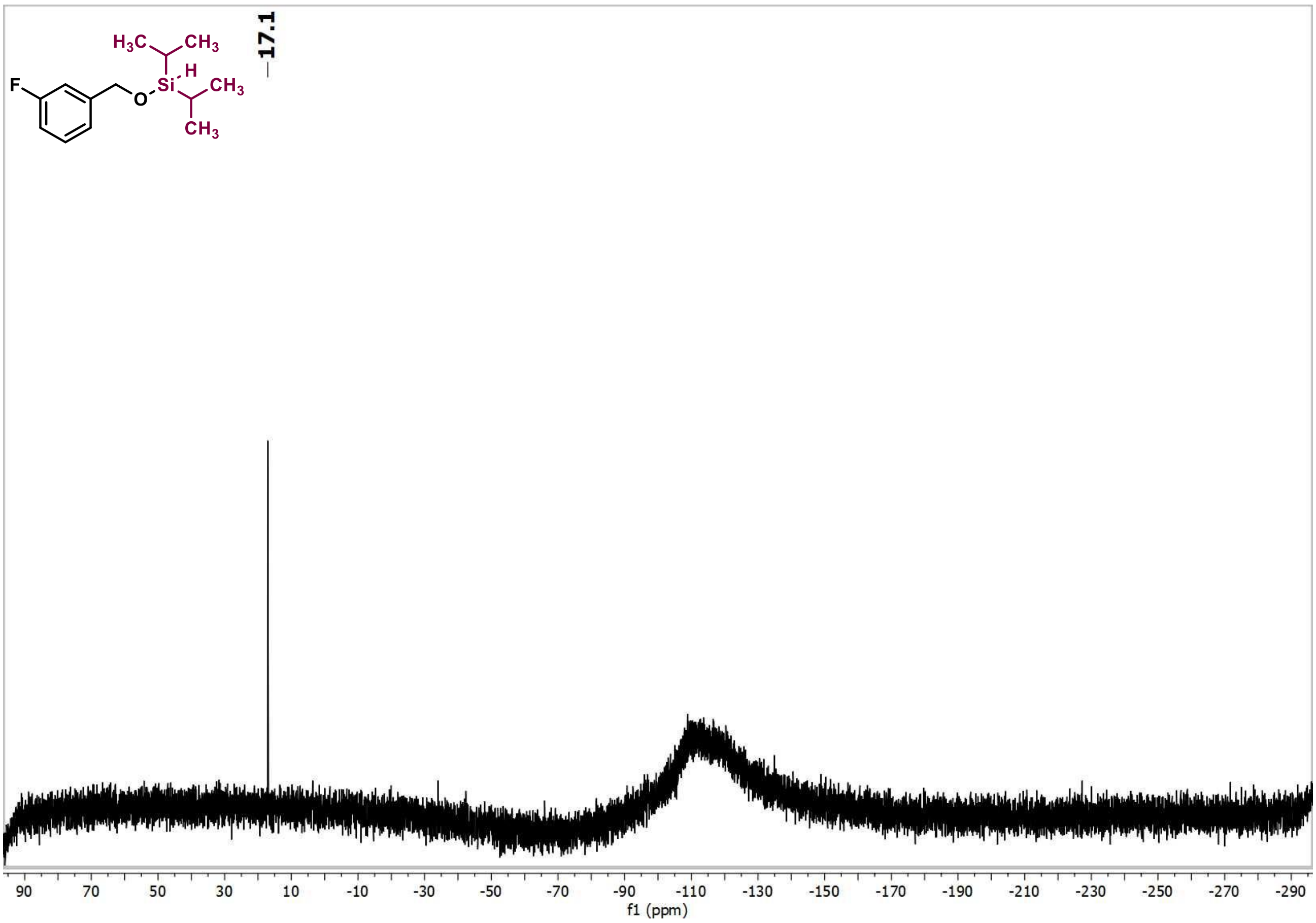

Figure S104. ^{29}Si NMR (79 MHz, Chloroform-d, 25°C) of ((3-fluorobenzyl)oxy)diisopropylsilane (**3ag**).

tert-Butyldimethyl(undec-10-en-1-yloxy)silane (**3ah**)

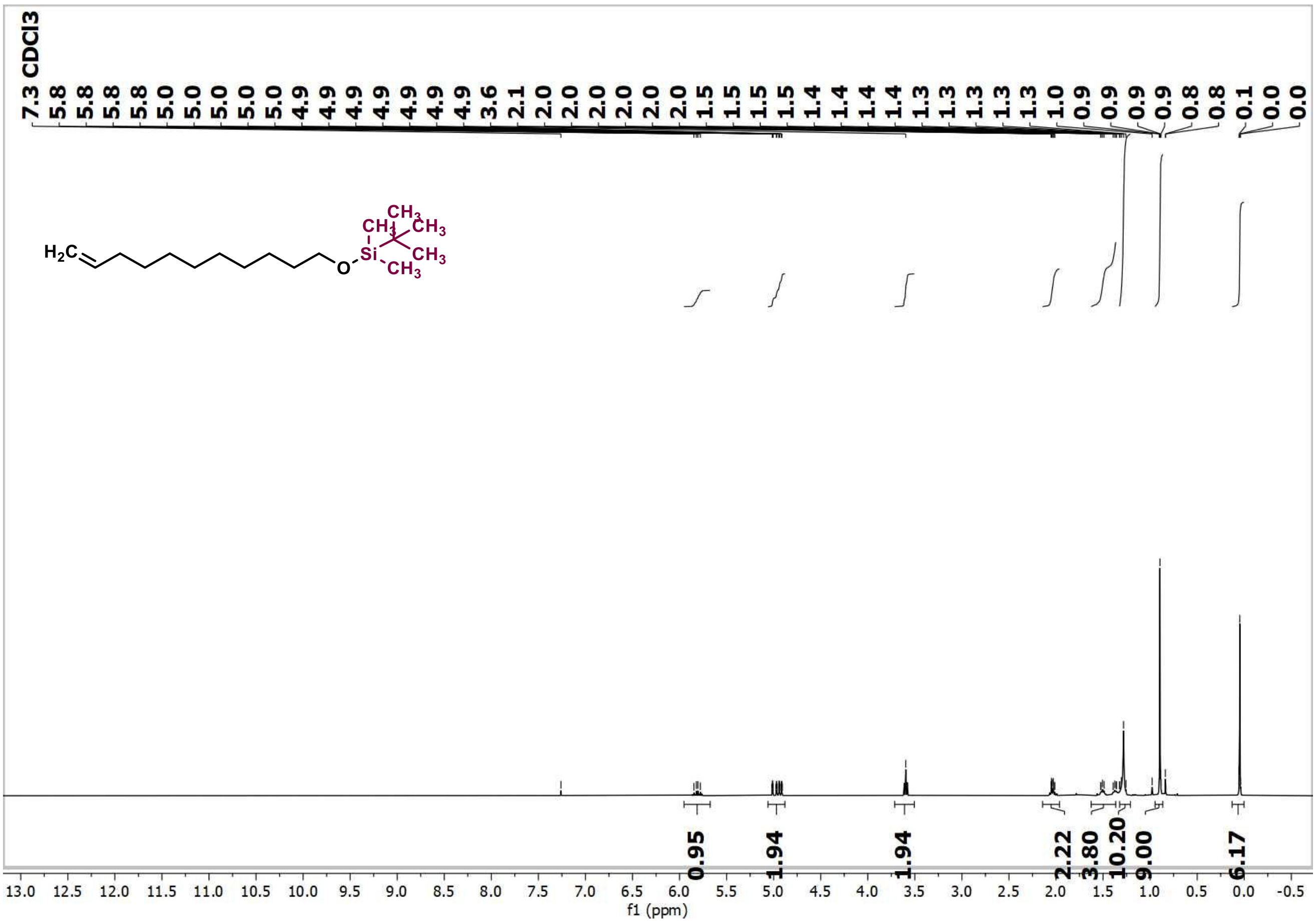

Figure S105. ^{1}H NMR (400 MHz, Chloroform-d, 25°C) of *tert*-butyldimethyl(undec-10-en-1-yloxy)silane (**3ah**).

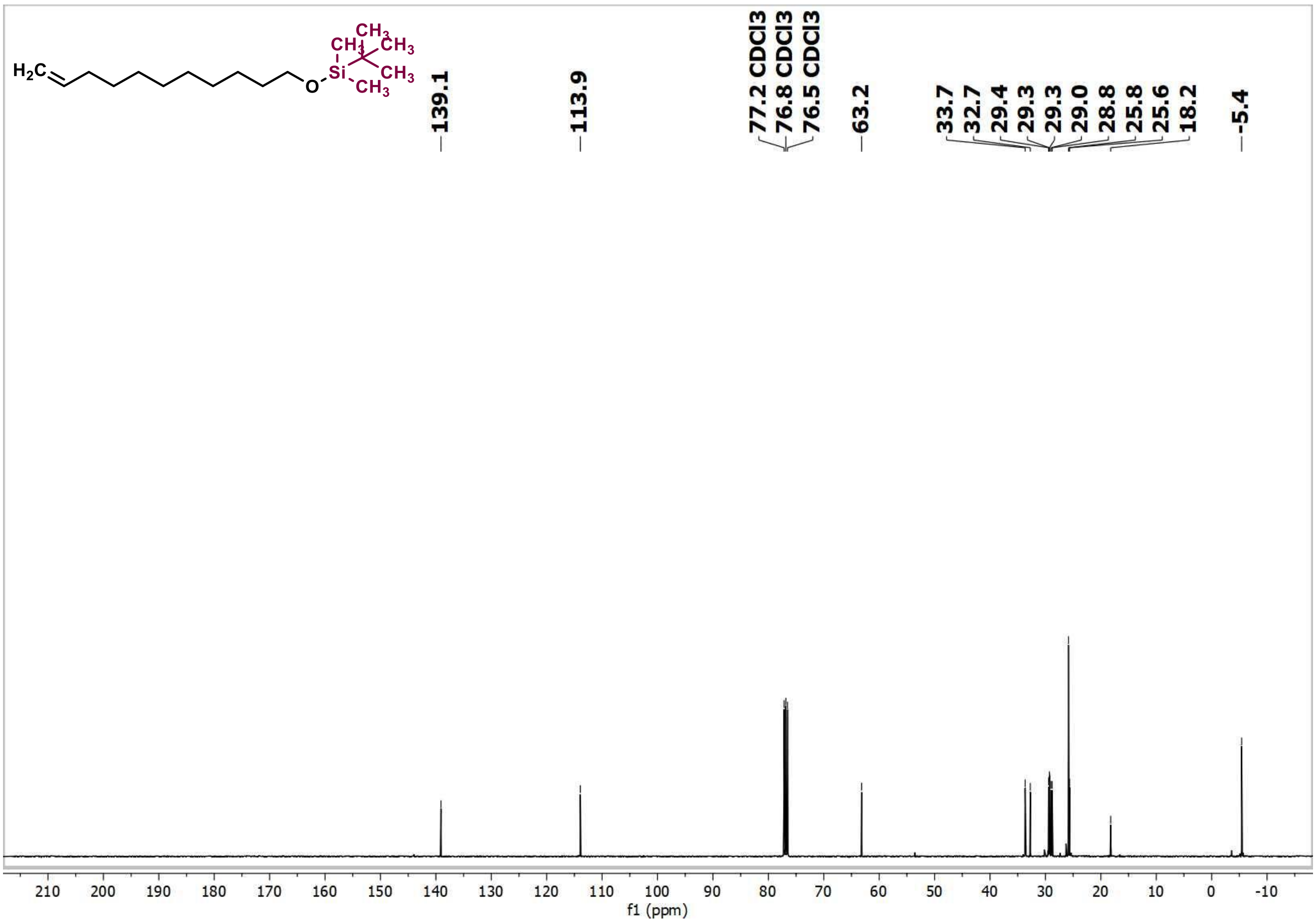

Figure S106. ^{13}C NMR (101 MHz, Chloroform-d, 25°C) of *tert*-butyldimethyl(undec-10-en-1-yloxy)silane (**3ah**).

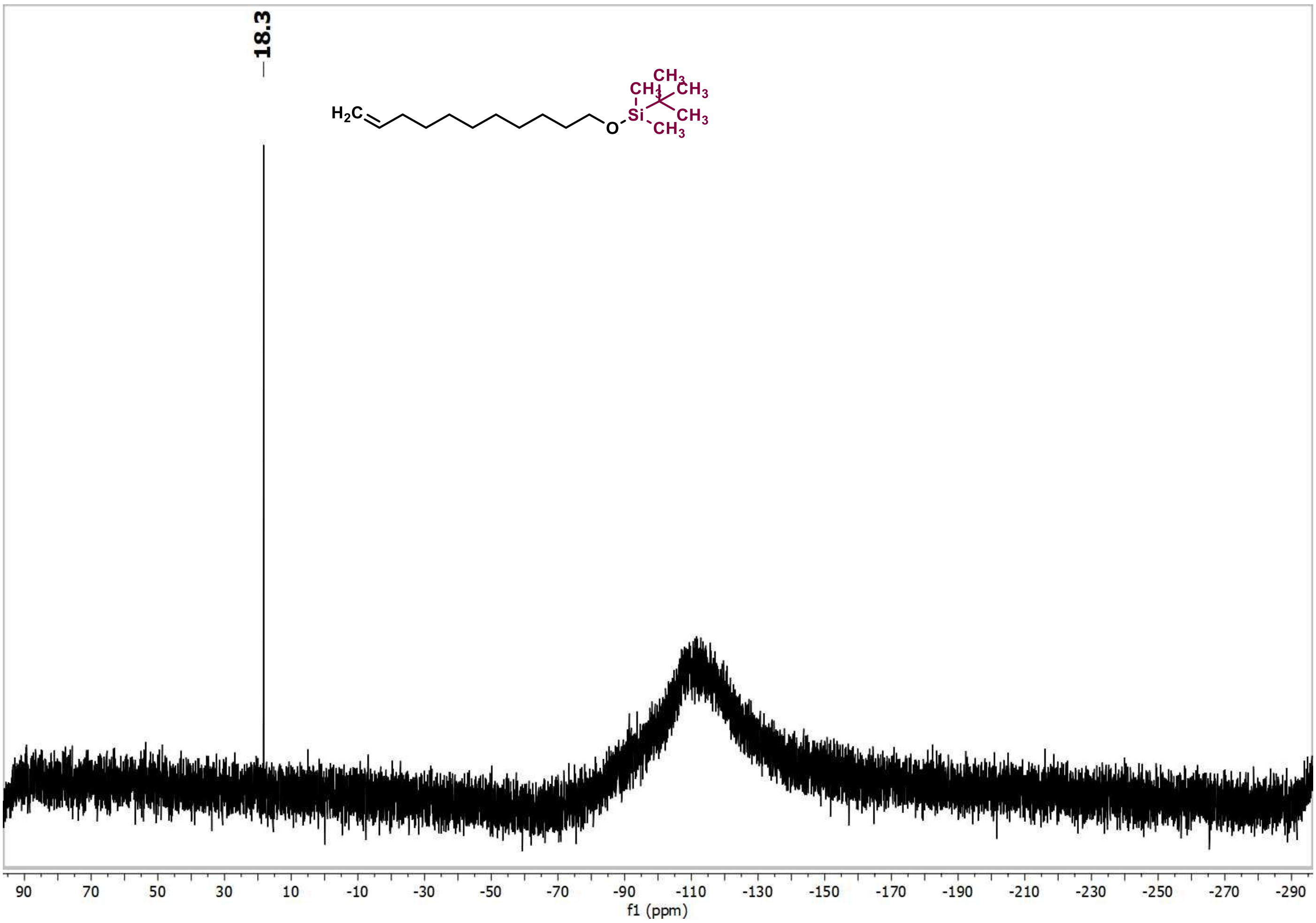

Figure S107. ^{29}Si NMR (101 MHz, Chloroform-d, 25°C) of *tert*-butyldimethyl(undec-10-en-1-yloxy)silane (**3ah**).

1,1,1-Trimethyl-3,3,3-tripropyldisiloxane (**4a**)

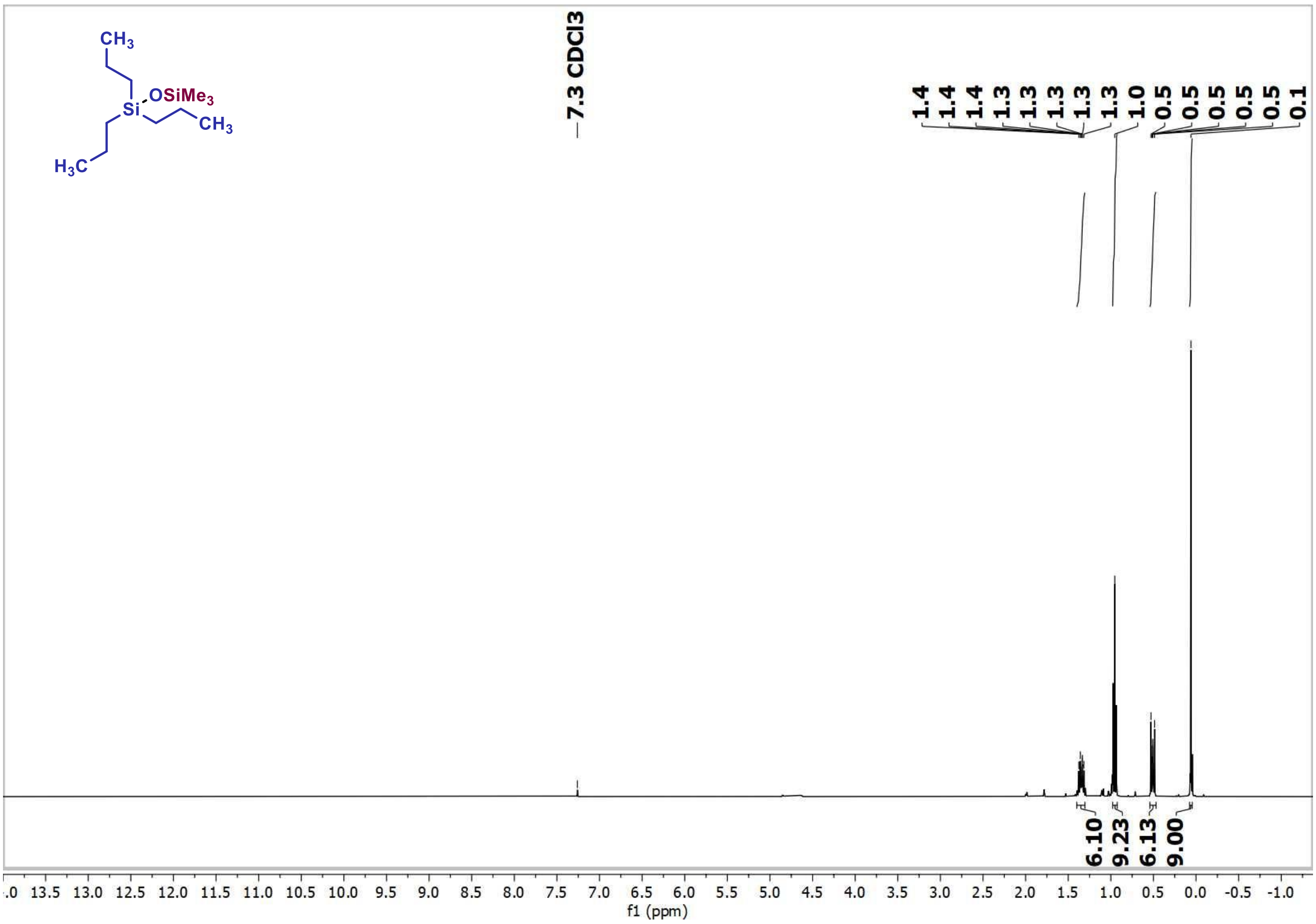

Figure S108. ^{1}H NMR (400 MHz, Chloroform-d, 25°C) of 1,1,1-trimethyl-3,3,3-tripropyldisiloxane (**4a**).

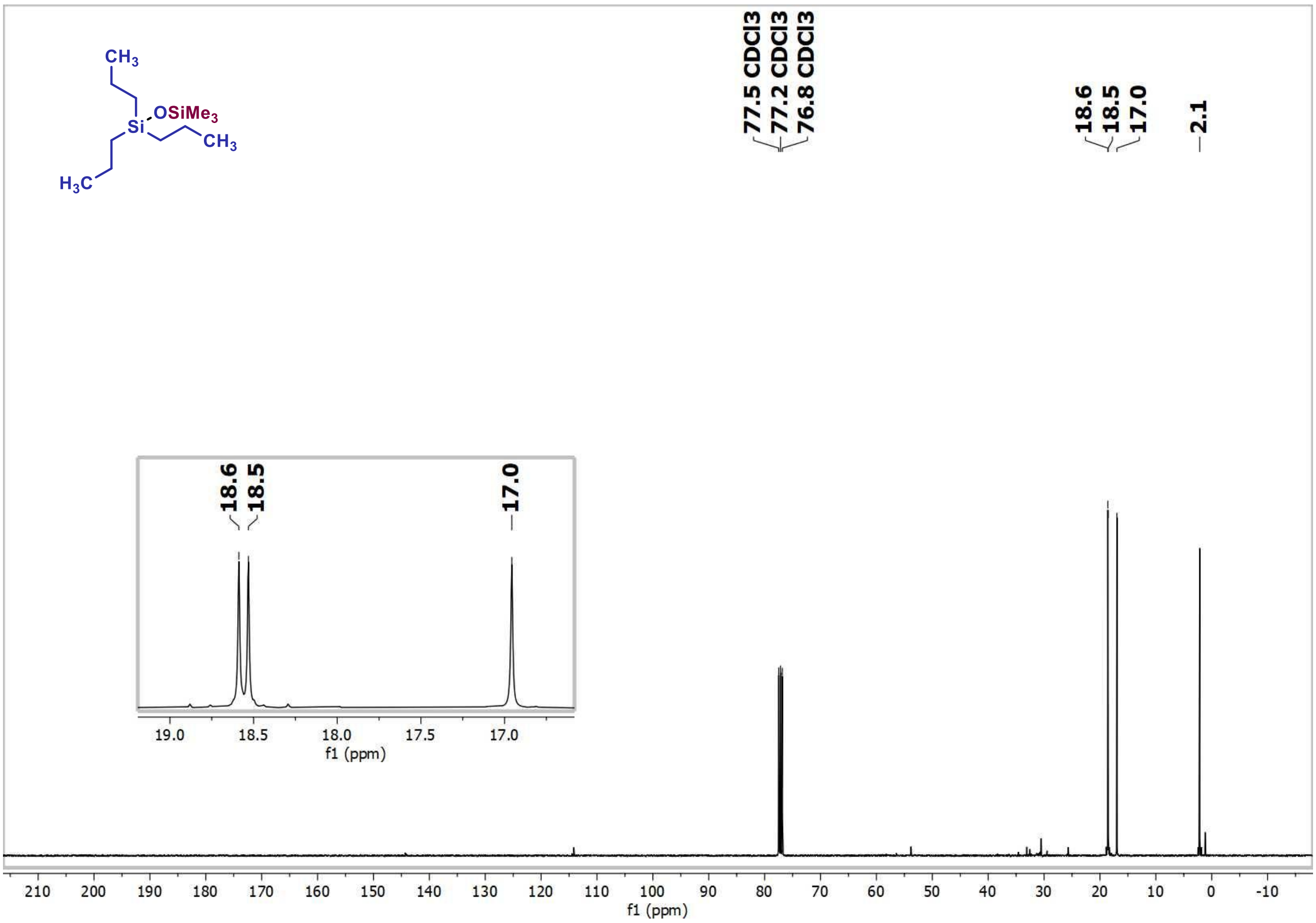

Figure S109. ^{13}C NMR (101 MHz, Chloroform-d, 25°C) of 1,1,1-trimethyl-3,3,3-tripropyldisiloxane (**4a**).

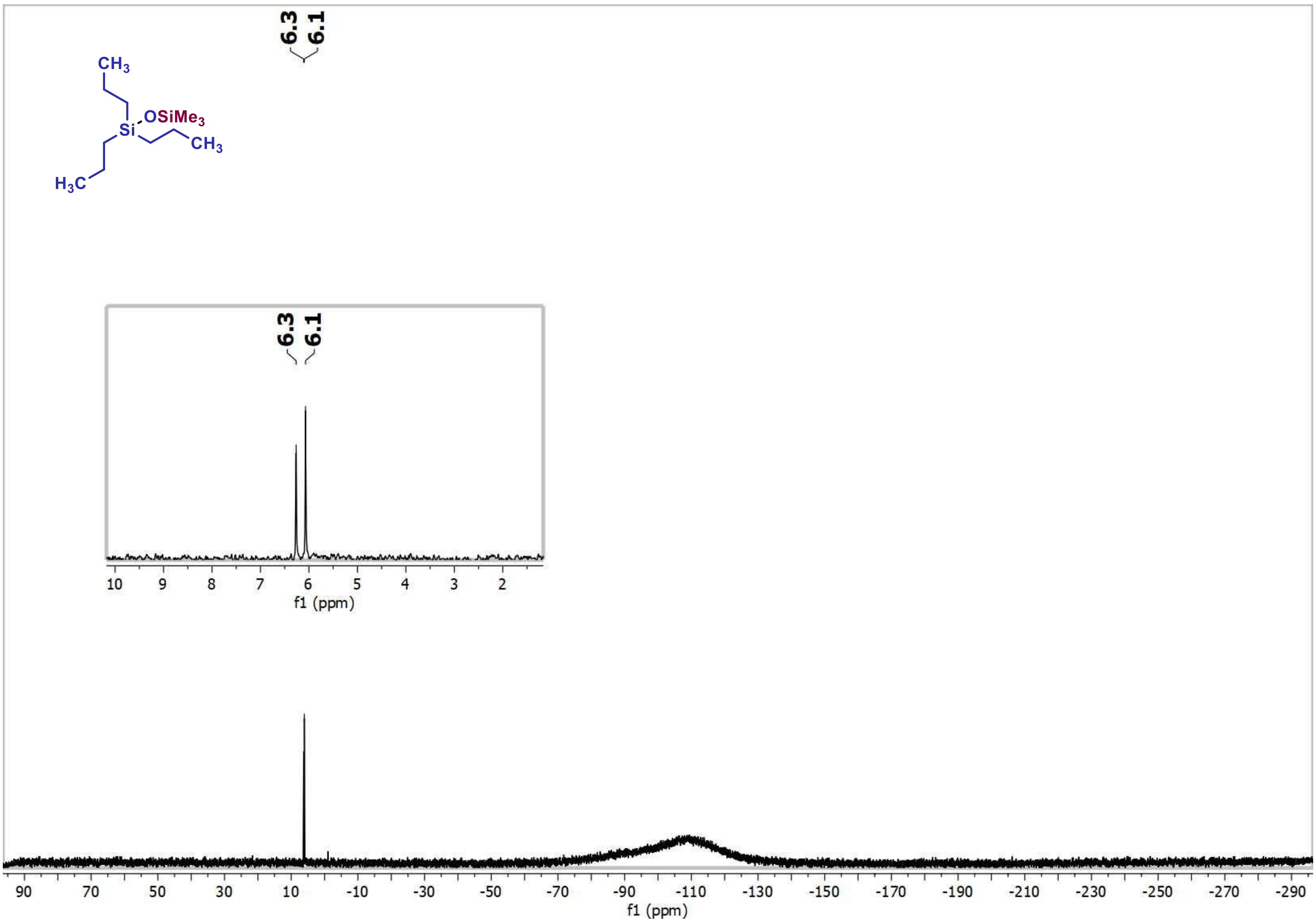

Figure S110. ^{29}Si NMR (79 MHz, Chloroform-d, 25°C) of 1,1,1-trimethyl-3,3,3-tripropyldisiloxane (**4a**).

1,1,1-Triisobutyl-3,3,3-trimethyldisiloxane (**4b**)

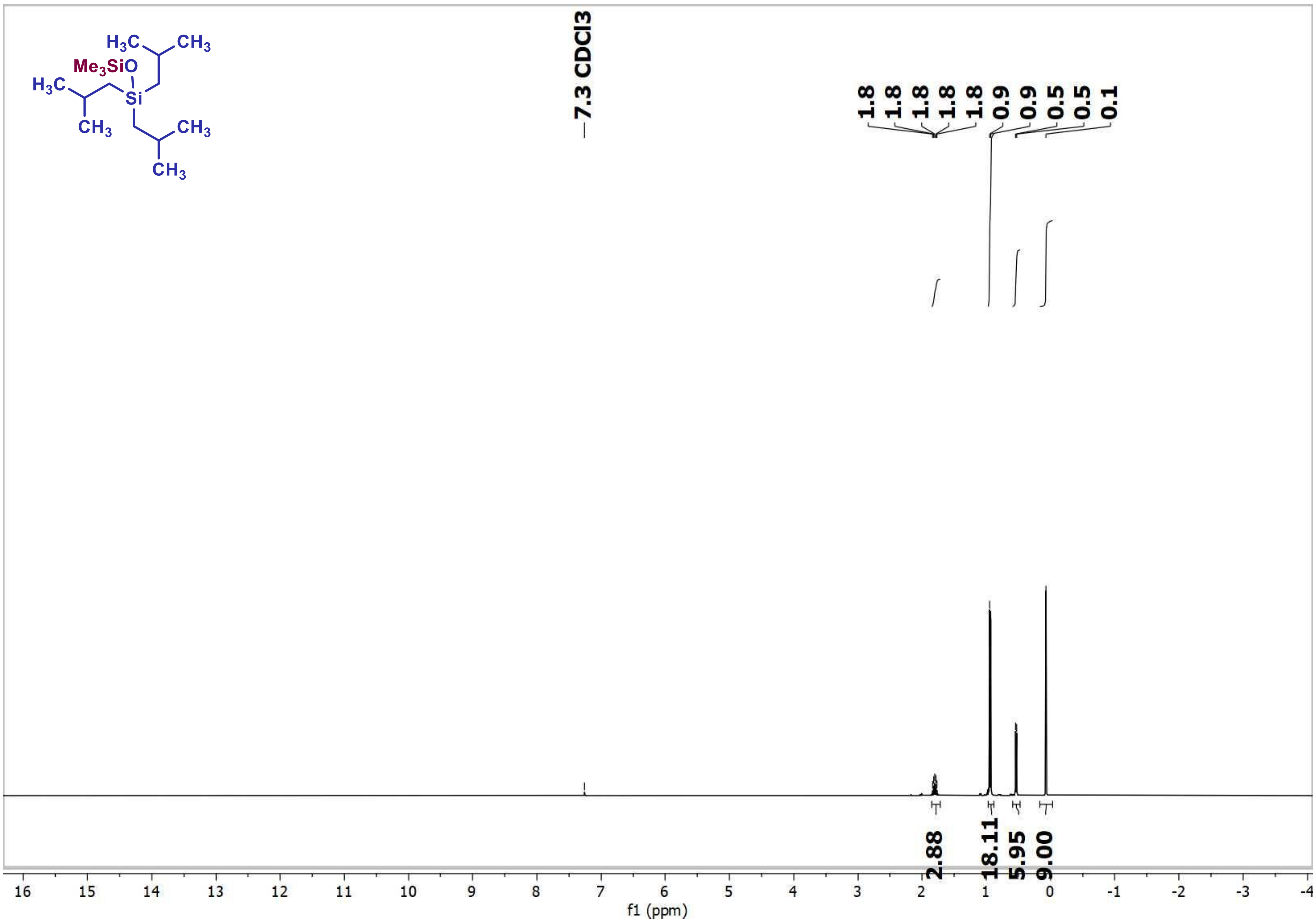

Figure S111. ^{1}H NMR (400 MHz, Chloroform-d, 25°C) of 1,1,1-triisobutyl-3,3,3-trimethyldisiloxane (**4b**).

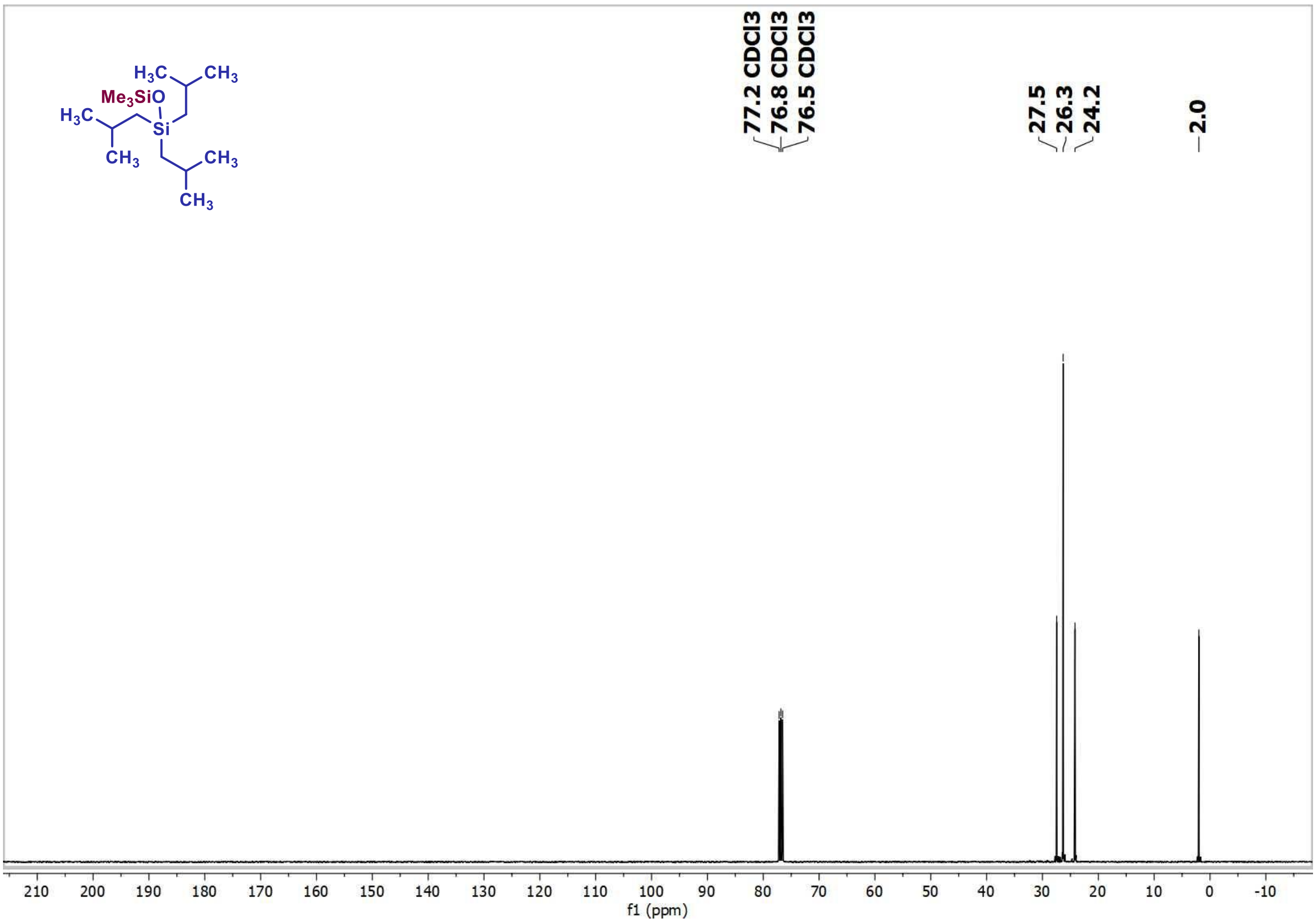

Figure S112. ^{13}C NMR (101 MHz, Chloroform-d, 25°C) of 1,1,1-triisobutyl-3,3,3-trimethyldisiloxane (**4b**).

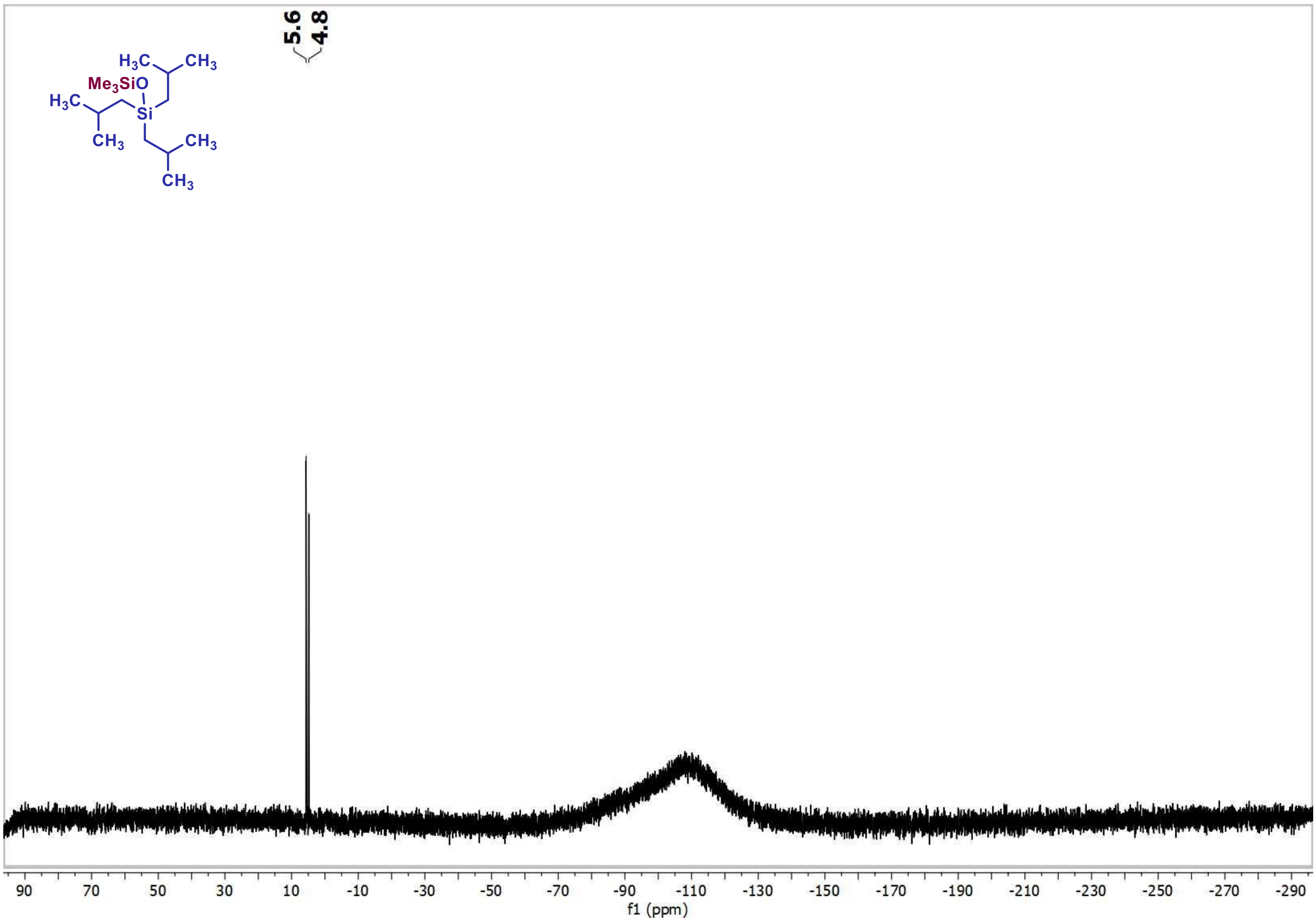

Figure S113. ^{29}Si NMR (79 MHz, Chloroform-d, 25°C) of 1,1,1-triisobutyl-3,3,3-trimethyldisiloxane (**4b**).

1,1,1-Triisopropyl-3,3,3-trimethyldisiloxane (**4c**)

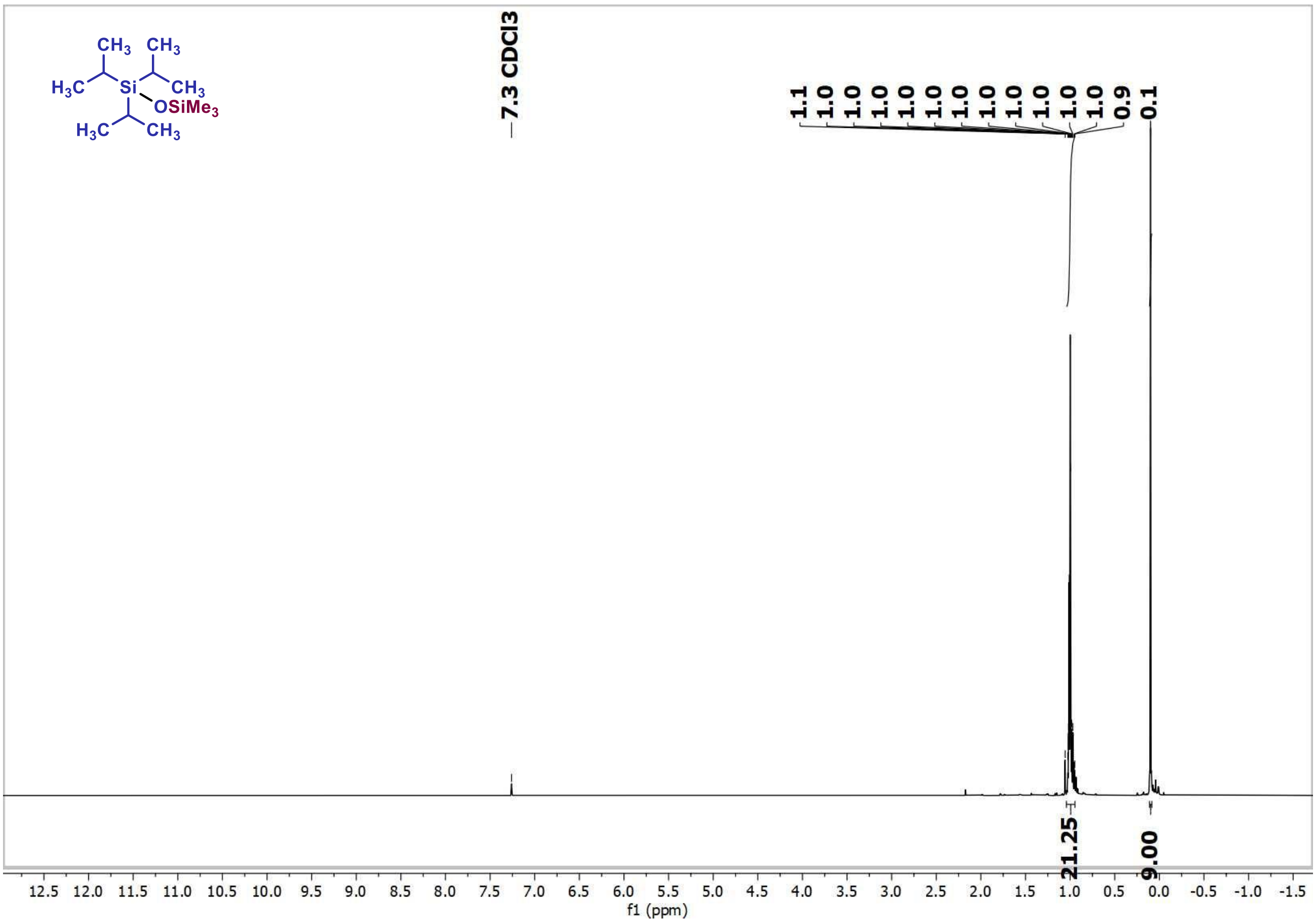

Figure S114. ^{1}H NMR (400 MHz, Chloroform-d, 25°C) of 1,1,1-triisopropyl-3,3,3-trimethyldisiloxane (**4c**).

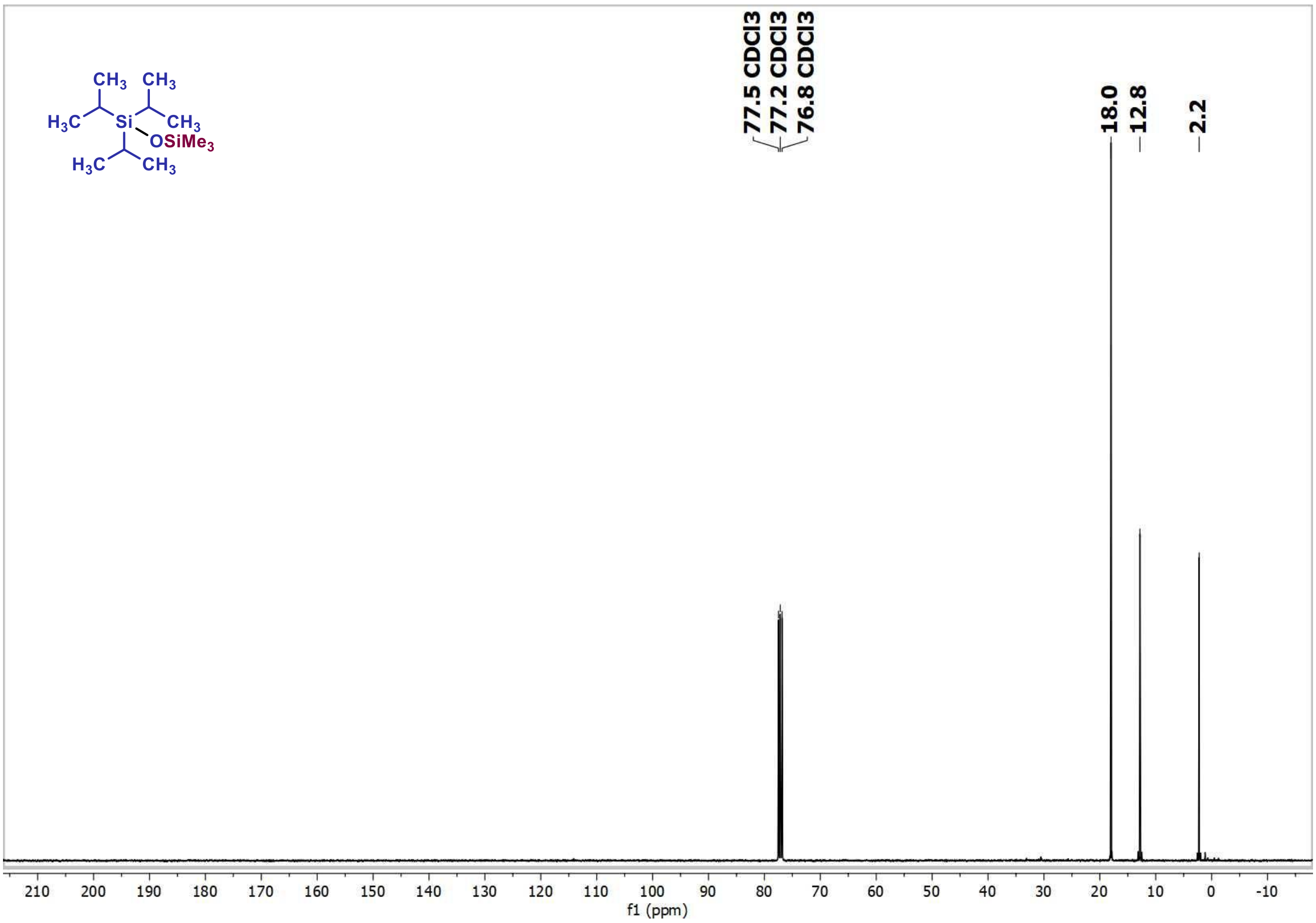

Figure S115. ^{13}C NMR (101 MHz, Chloroform-d, 25°C) of 1,1,1-triisopropyl-3,3,3-trimethyldisiloxane (**4c**).

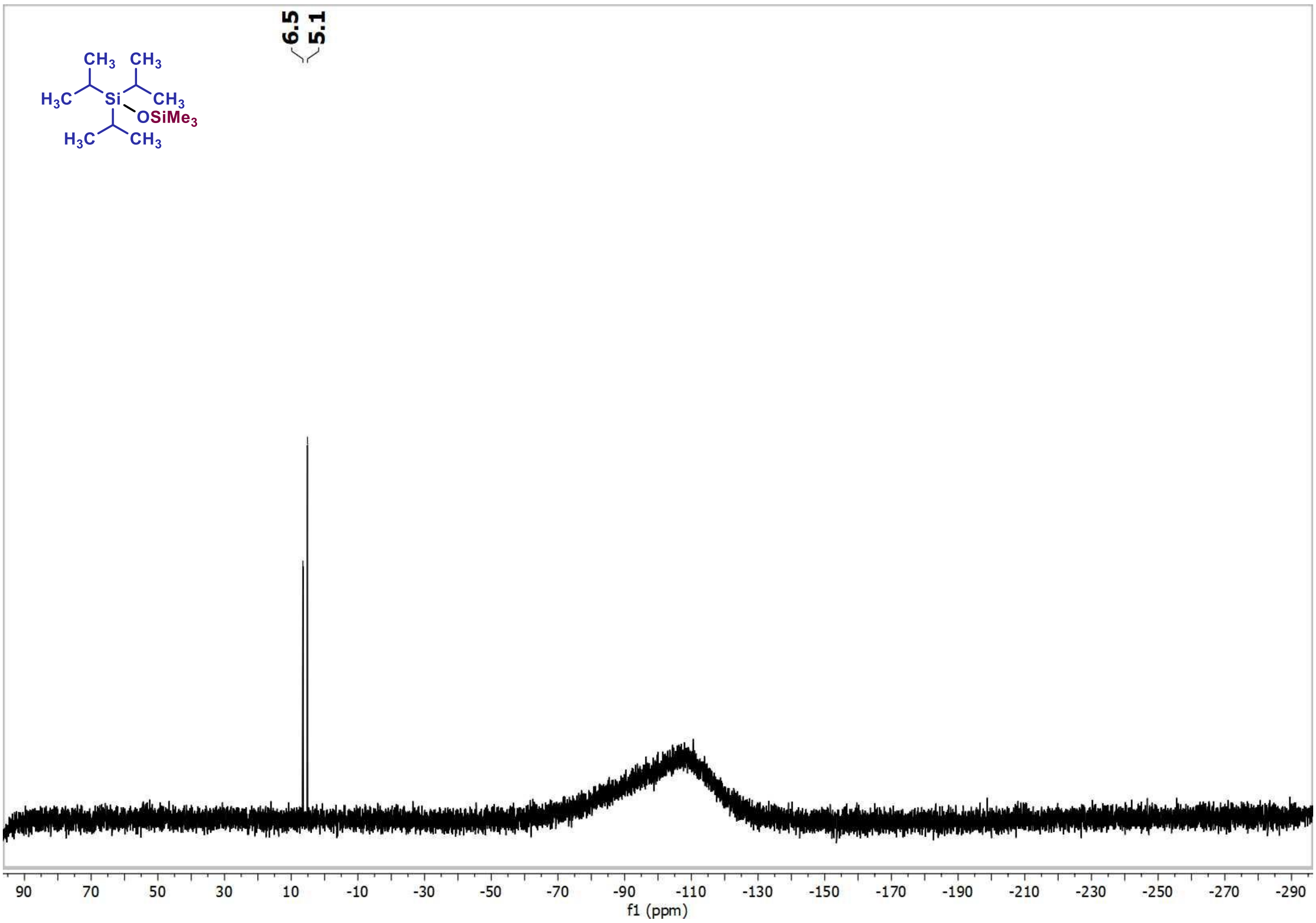

Figure S116. ^{29}Si NMR (79 MHz, Chloroform-d, 25°C) of 1,1,1-triisopropyl-3,3,3-trimethyldisiloxane (**4c**).

Tri-*tert*-butyl (trimethylsilyl) silicate (**4d**)

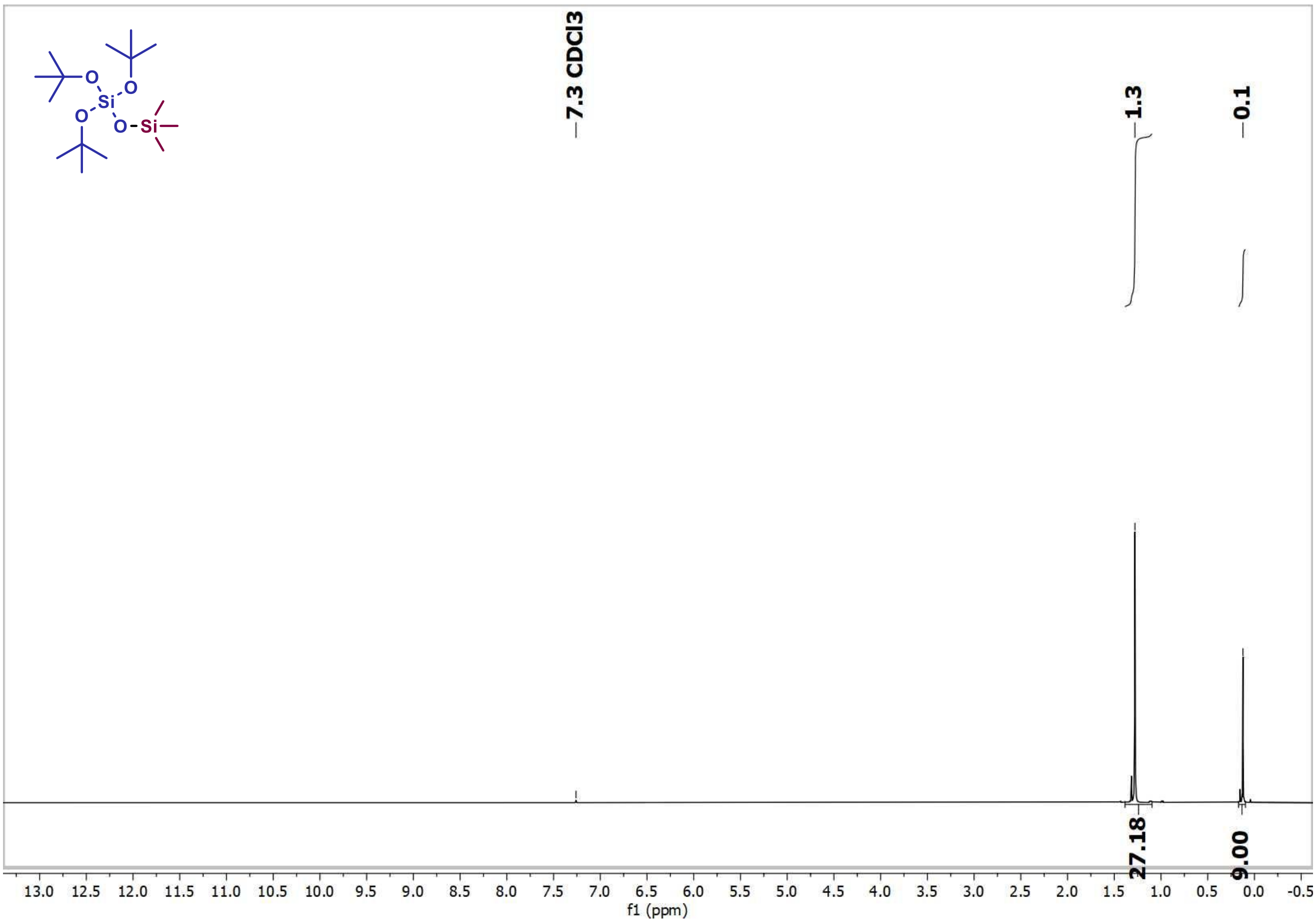

Figure S117. ^{1}H NMR (400 MHz, Chloroform-d, 25°C) of tri-tert-butyl (trimethylsilyl) silicate (**4d**).

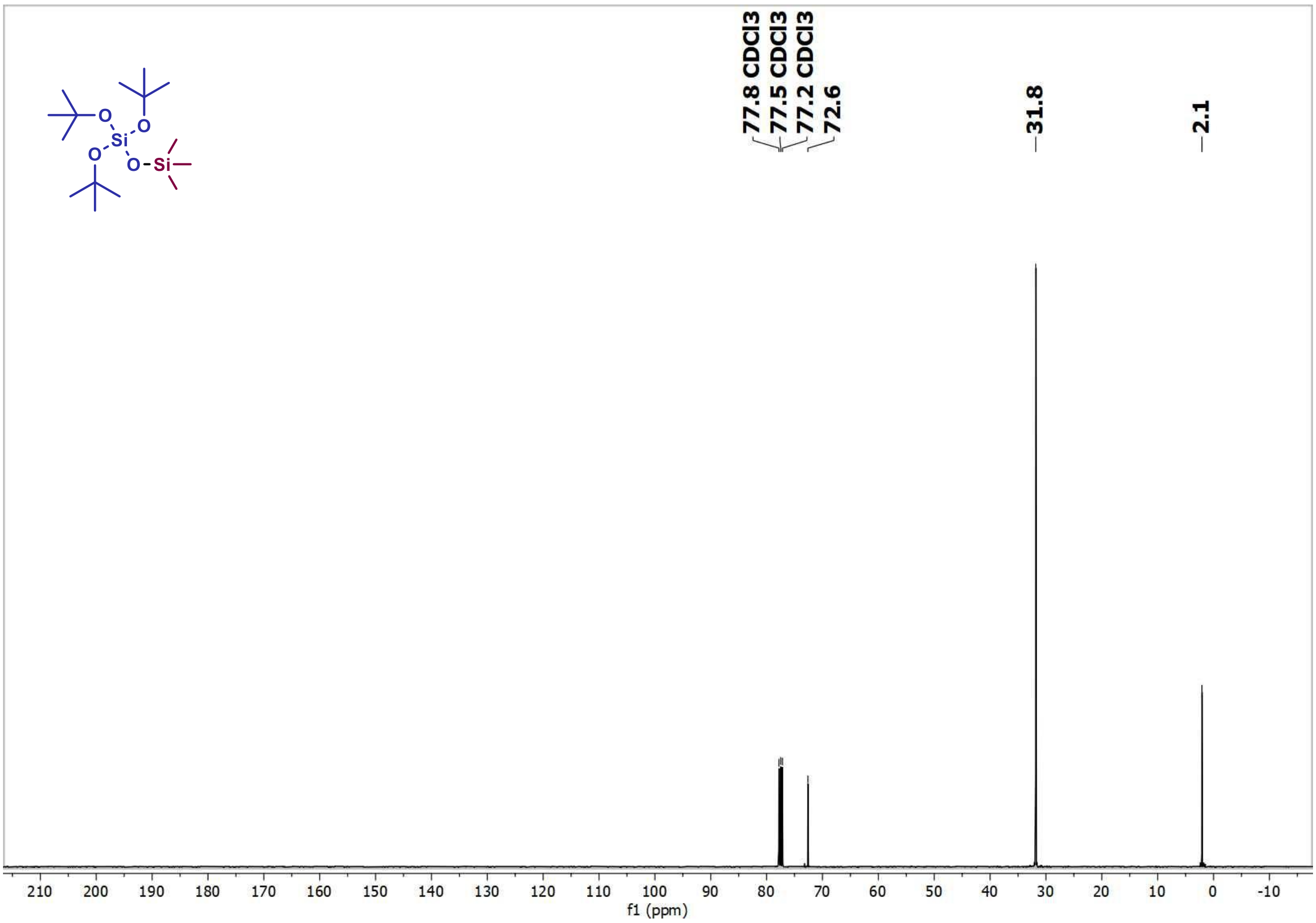

Figure S118. ^{13}C NMR (101 MHz, Chloroform-d, 25°C) of tri-tert-butyl (trimethylsilyl) silicate (**4d**).

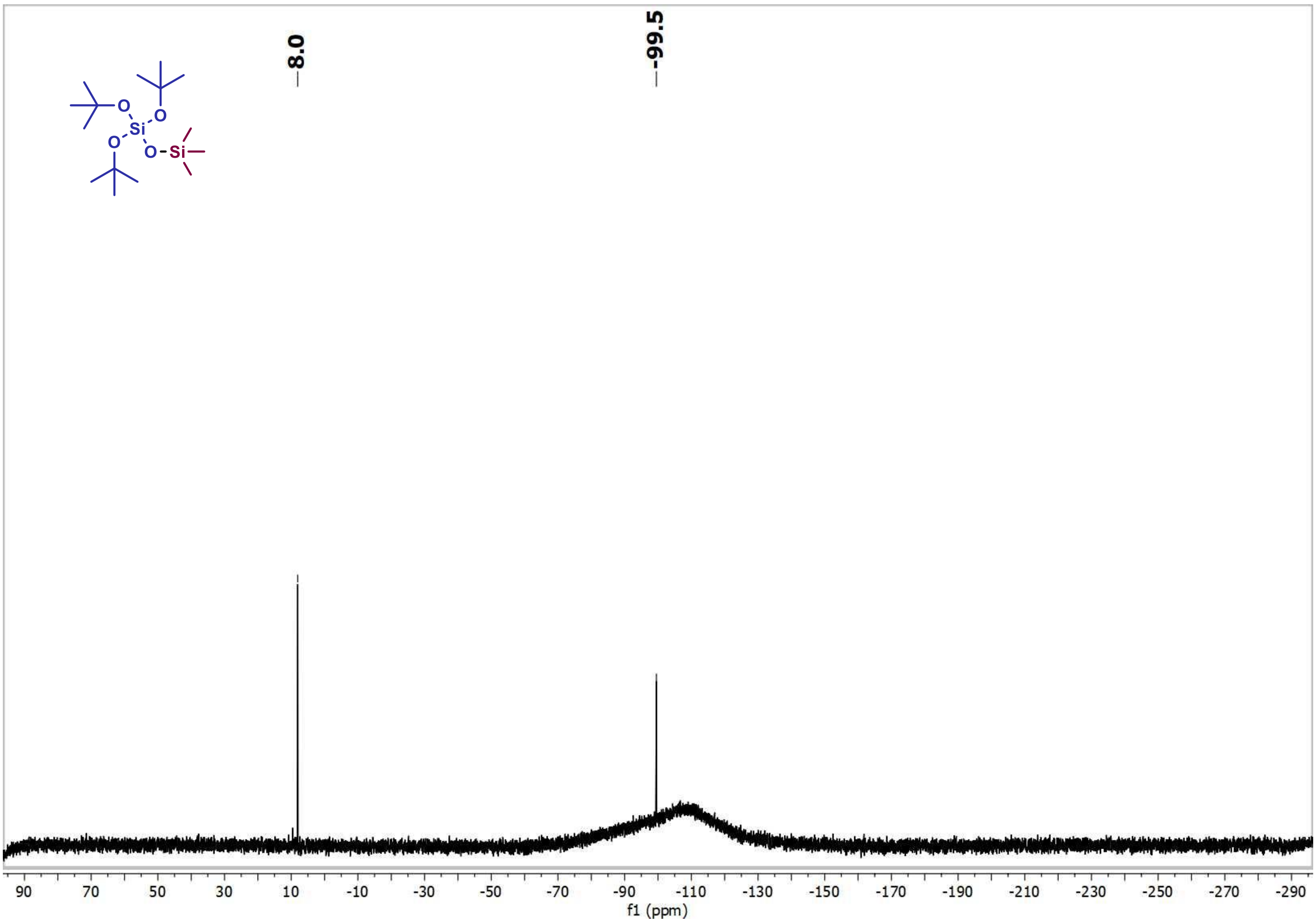

Figure S119. ^{1}H NMR (400 MHz, Chloroform-d, 25°C) of tri-tert-butyl (trimethylsilyl) silicate (**4d**).

Triisobutyl((4,4,5,5-tetramethyl-1,3,2-dioxaborolan-2-yl)oxy)silane (**5a**)

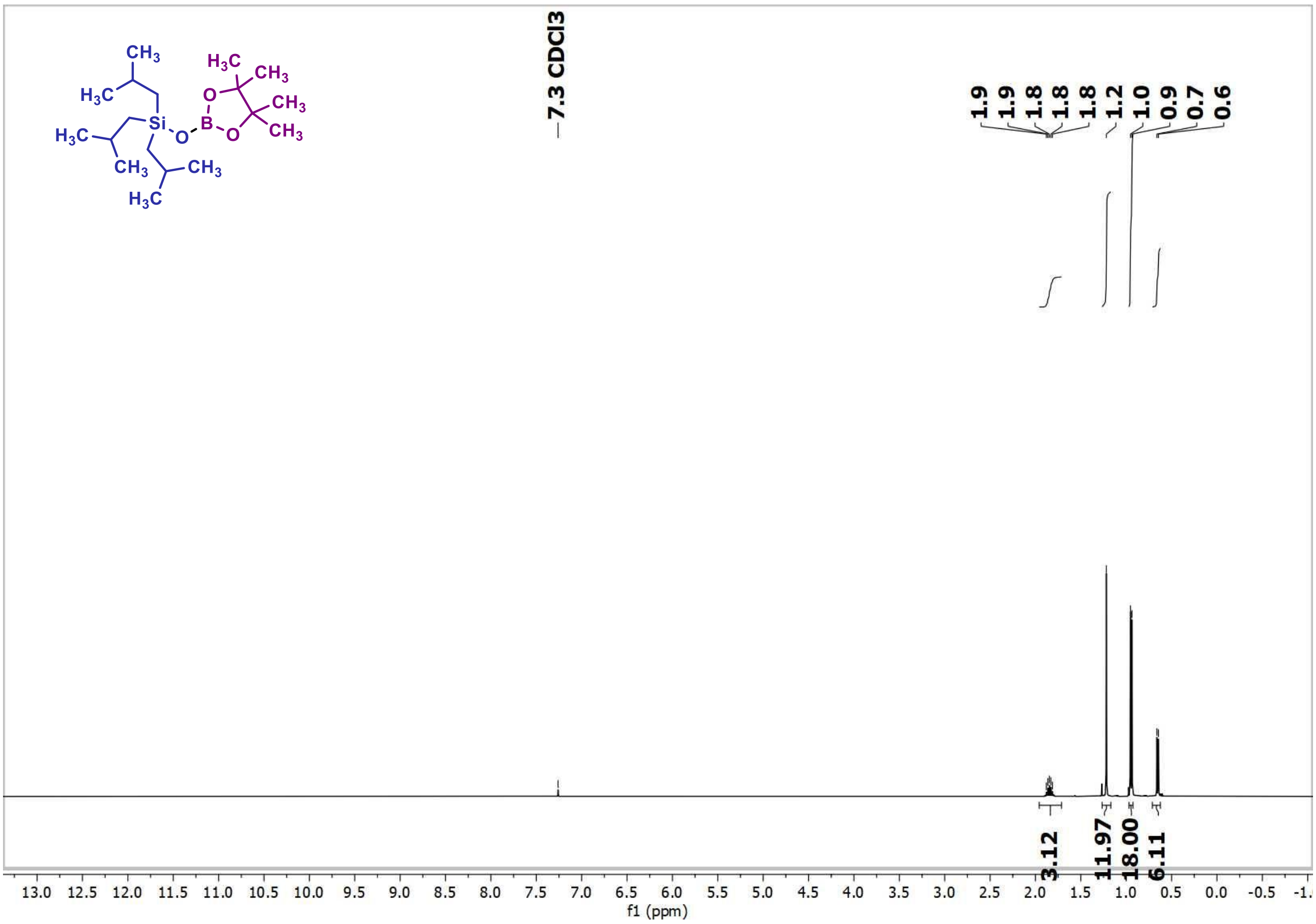

Figure S120. ¹H NMR (400 MHz, Chloroform-d, 25°C) of triisobutyl((4,4,5,5-tetramethyl-1,3,2-dioxaborolan-2-yl)oxy)silane (**5a**).

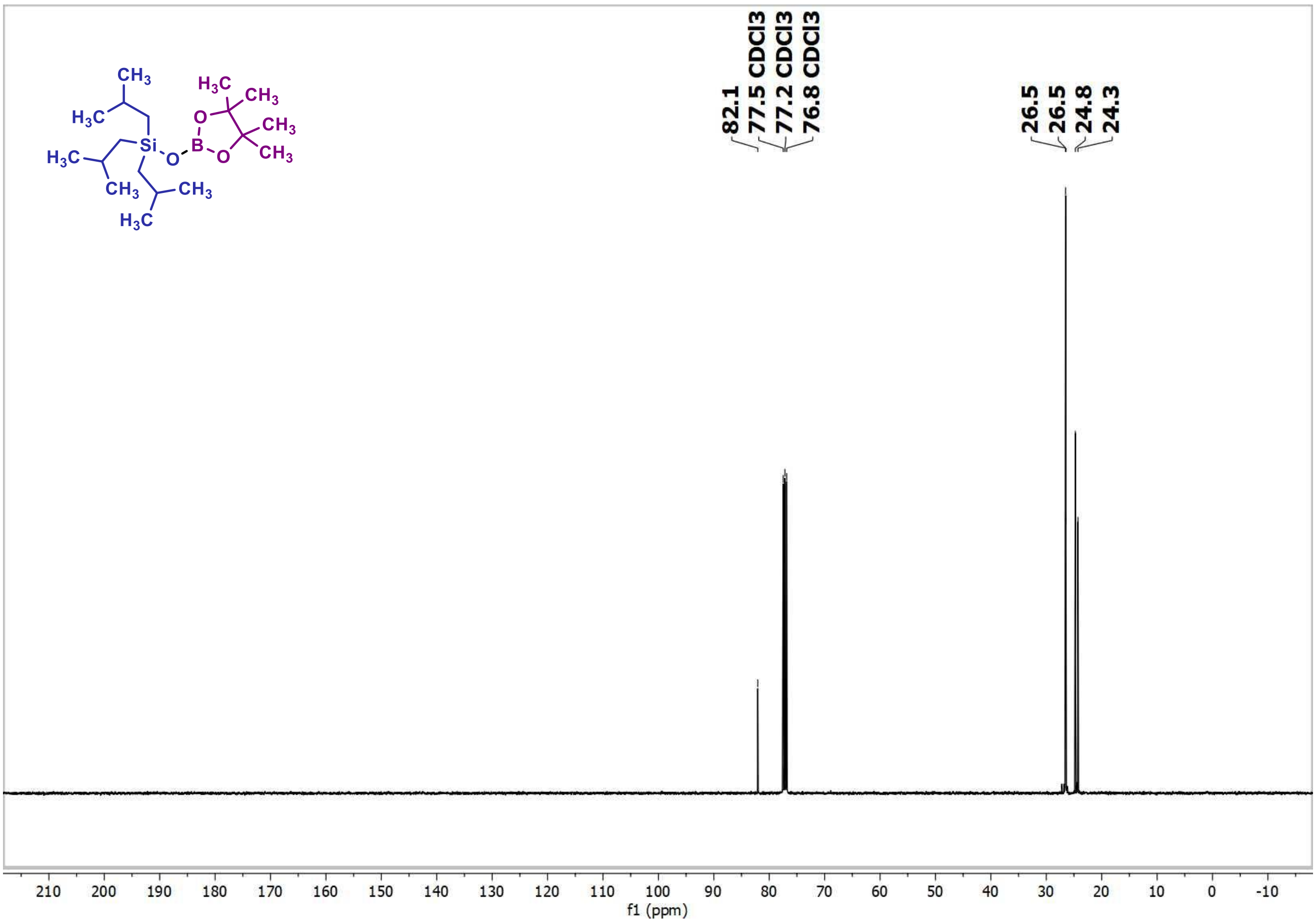

Figure S121. ¹³C NMR (101 MHz, Chloroform-d, 25°C) of triisobutyl((4,4,5,5-tetramethyl-1,3,2-dioxaborolan-2-yl)oxy)silane (**5a**).

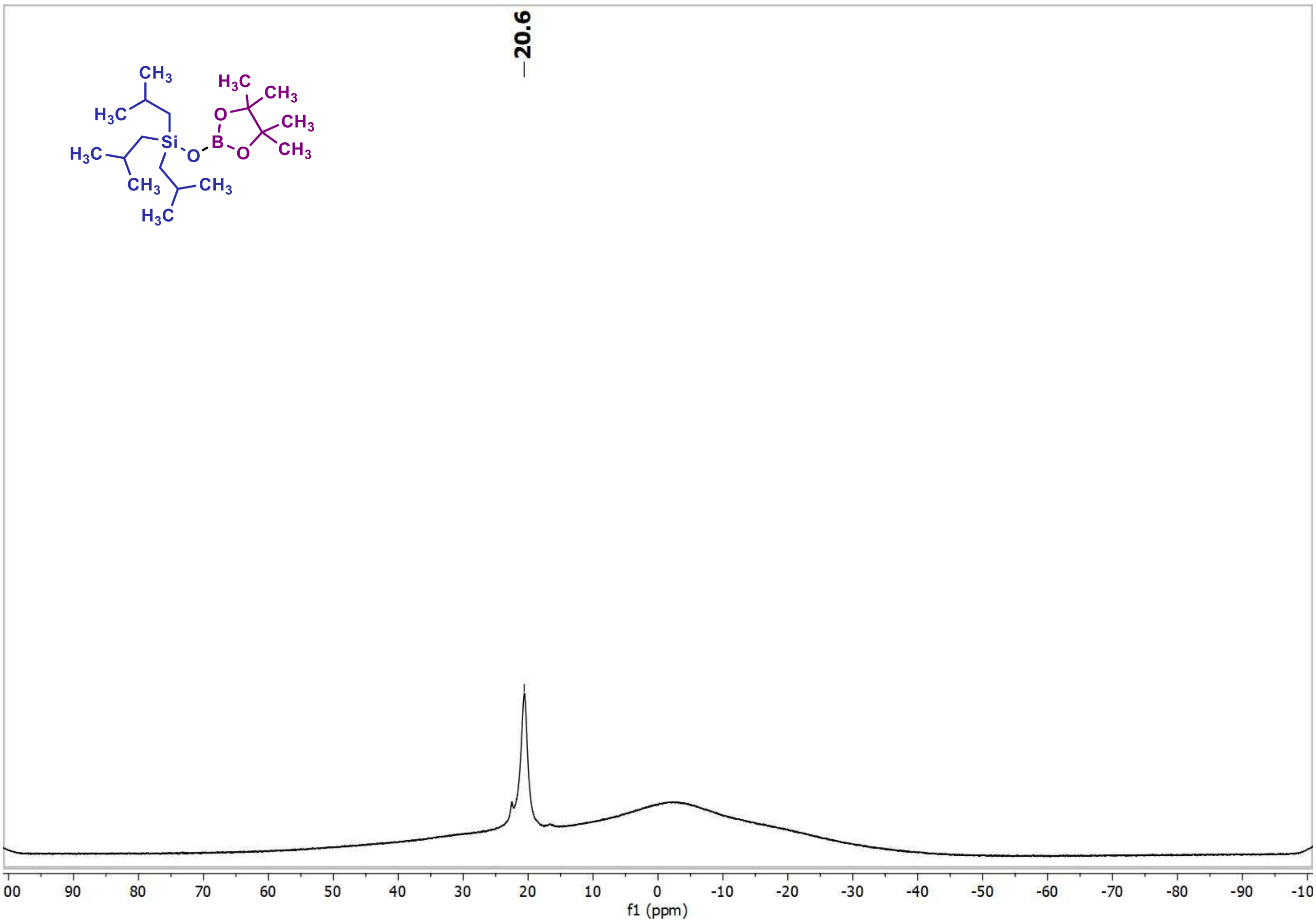

Figure S122. ^{11}B NMR (128 MHz, Chloroform-d, 25°C) of triisobutyl((4,4,5,5-tetramethyl-1,3,2-dioxaborolan-2-yl)oxy)silane (**5a**).

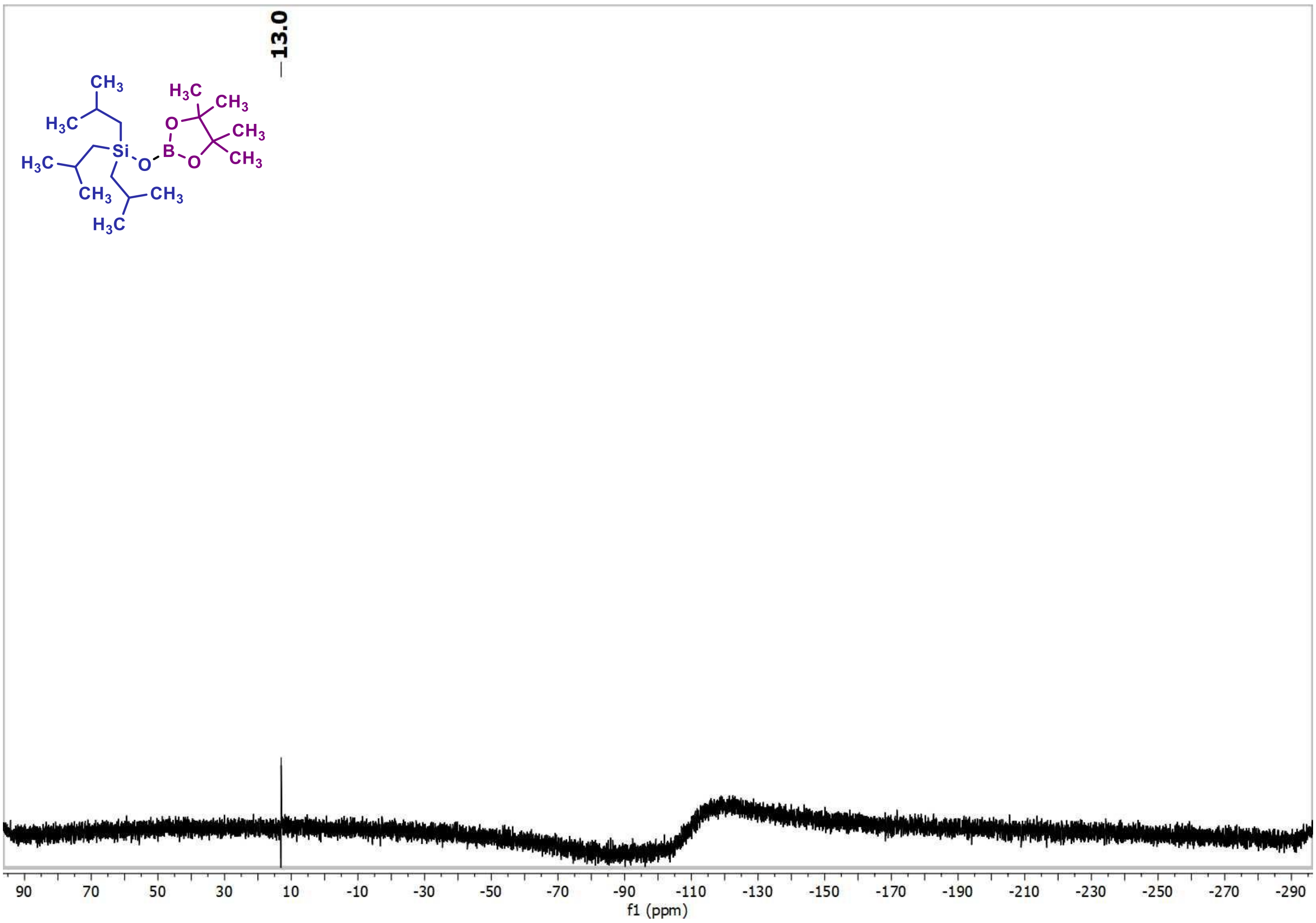

Figure S123. ^{29}Si NMR (79 MHz, Chloroform-d, 25°C) of triisobutyl((4,4,5,5-tetramethyl-1,3,2-dioxaborolan-2-yl)oxy)silane (**5a**).

tert-Butyldimethyl((4,4,5,5-tetramethyl-1,3,2-dioxaborolan-2-yl)oxy)silane (**5b**)

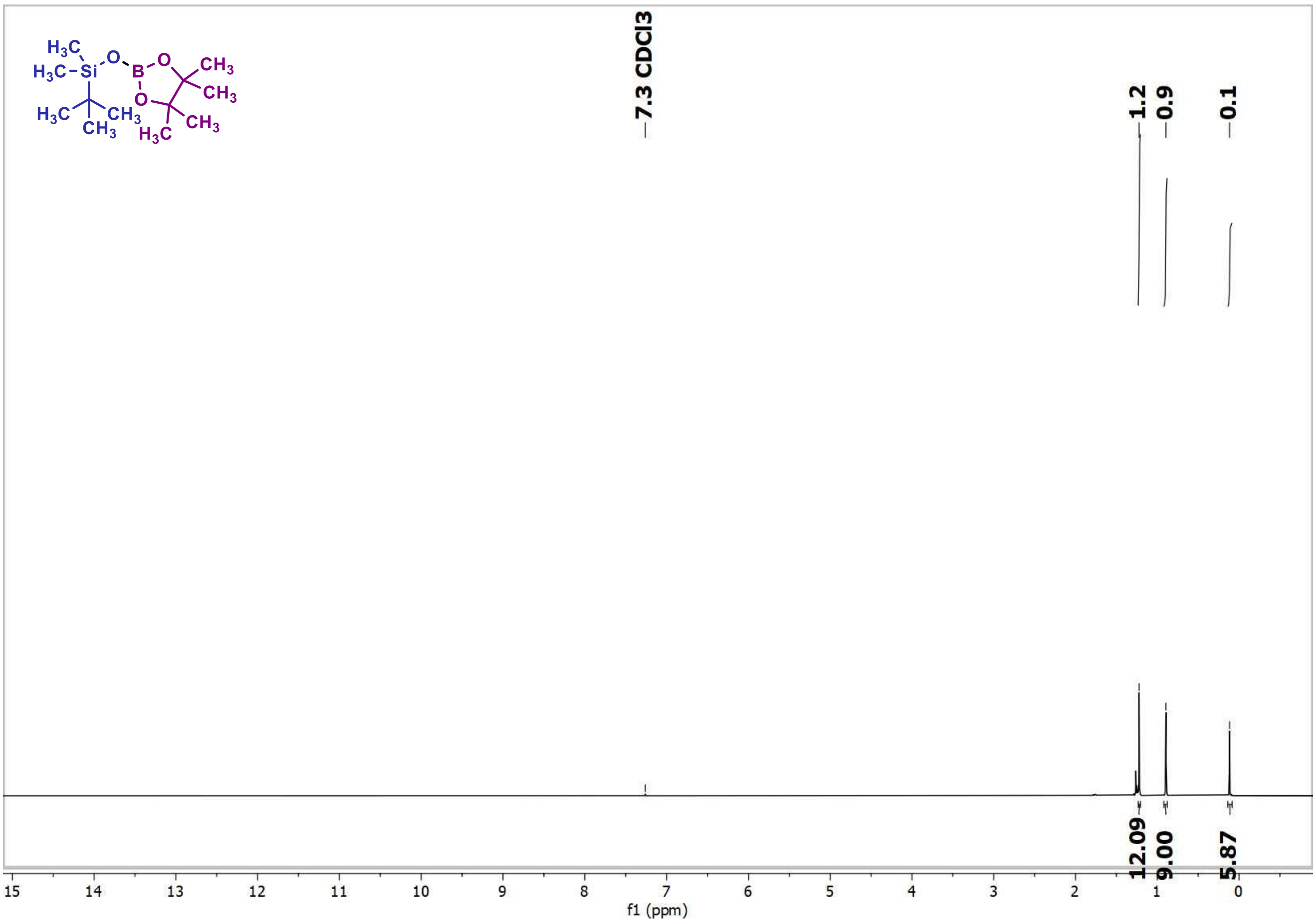

Figure S124. ^{1}H NMR (400 MHz, Chloroform-d, 25°C) of *tert*-butyldimethyl((4,4,5,5-tetramethyl-1,3,2-dioxaborolan-2-yl)oxy)silane (**5b**).

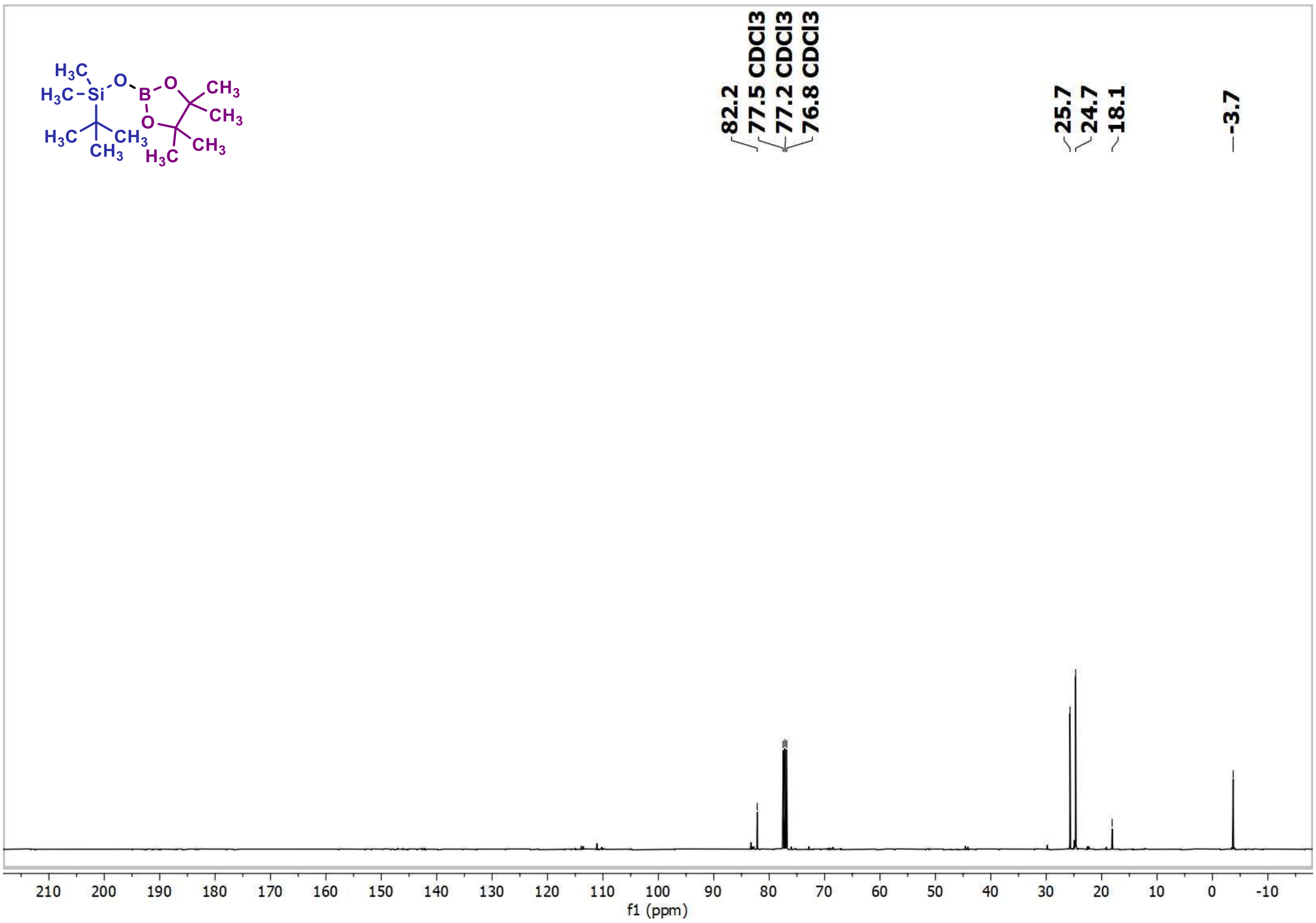

Figure S125. ^{13}C NMR (101 MHz, Chloroform-d, 25°C) of *tert*-butyldimethyl((4,4,5,5-tetramethyl-1,3,2-dioxaborolan-2-yl)oxy)silane (**5b**).

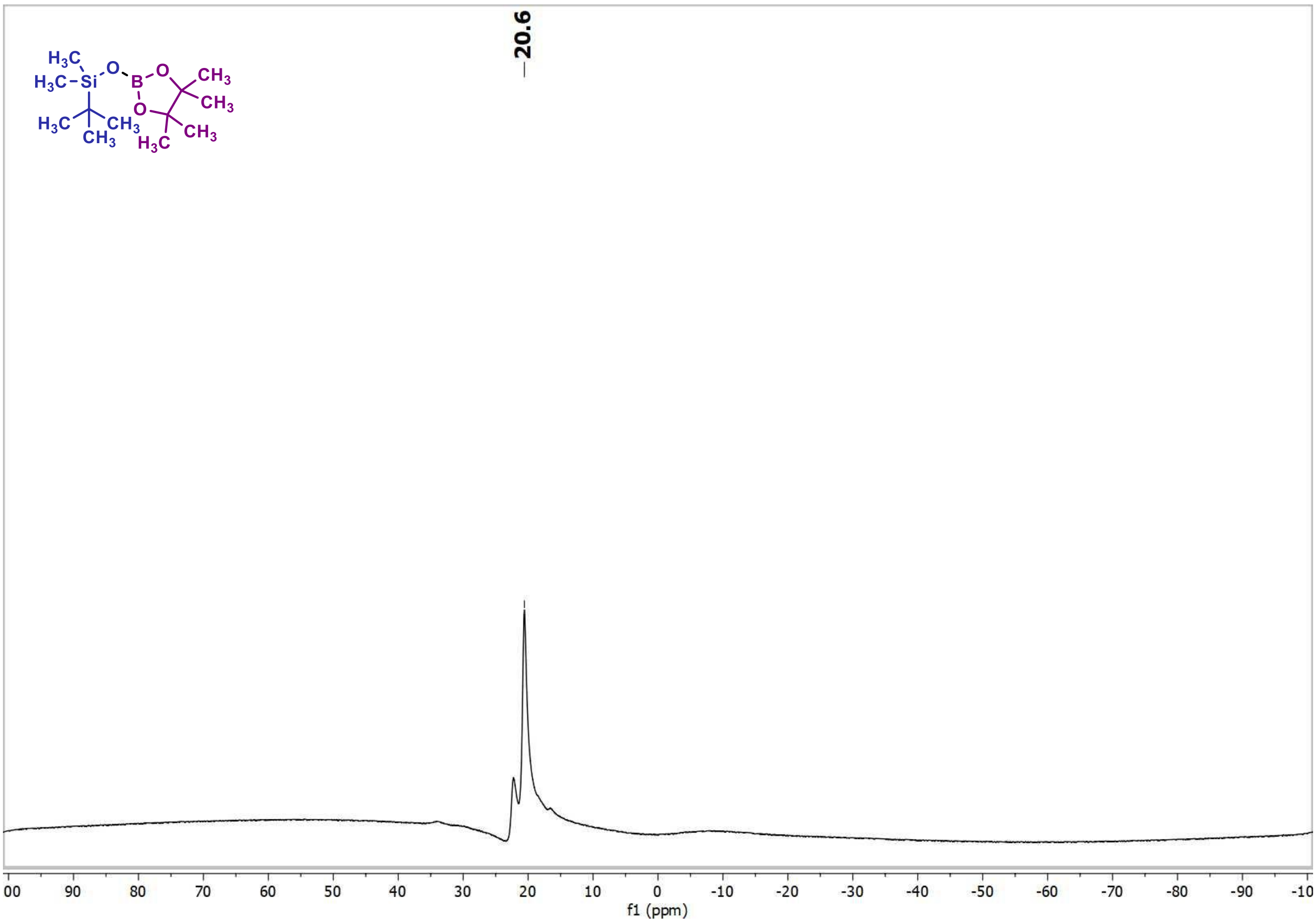

Figure S126. 11B NMR (128 MHz, Chloroform-d, 25°C) of *tert*-butyldimethyl((4,4,5,5-tetramethyl-1,3,2-dioxaborolan-2-yl)oxy)silane (**5b**).

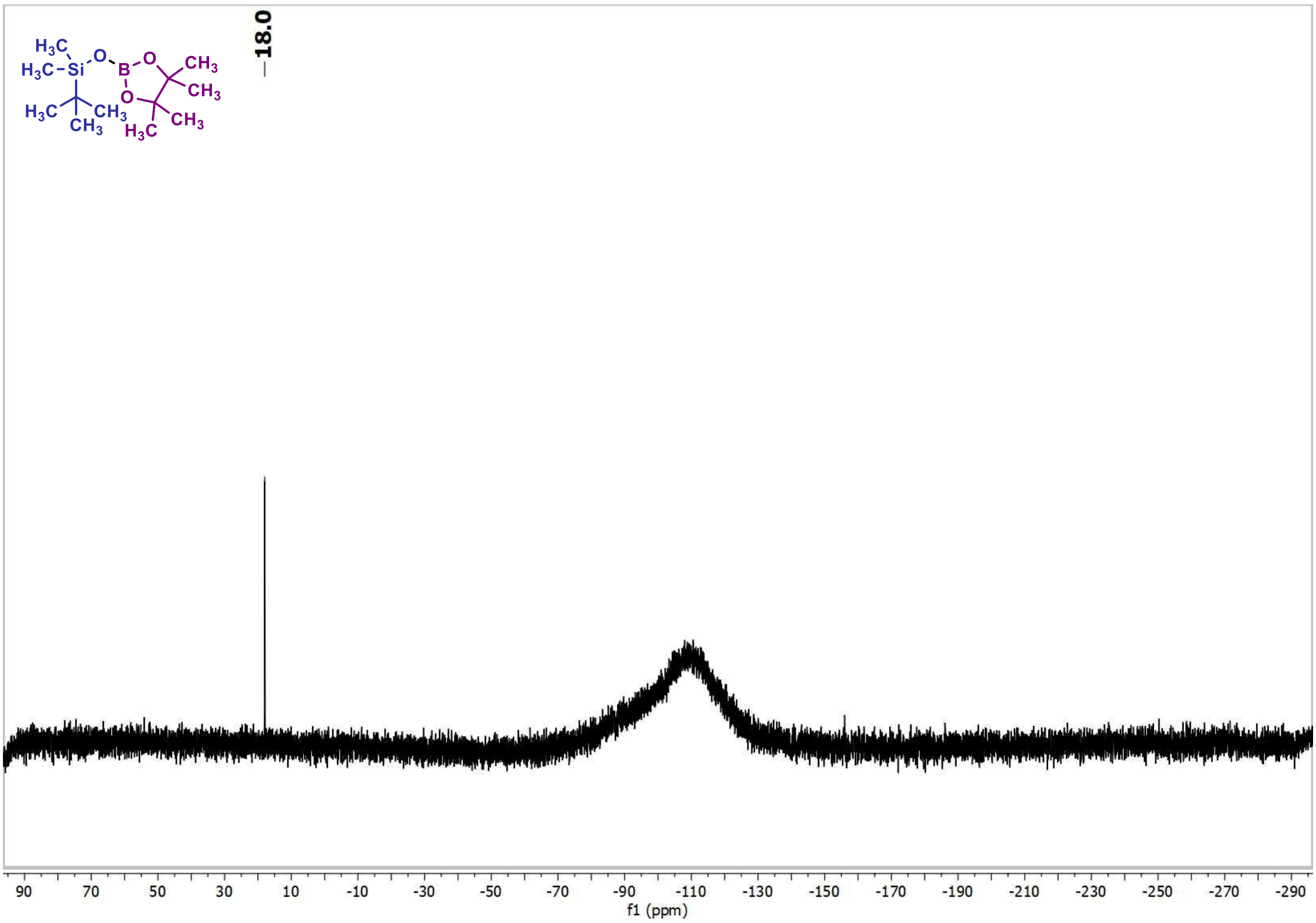

Figure S127. 29Si NMR (79 MHz, Chloroform-d, 25°C) of *tert*-butyldimethyl((4,4,5,5-tetramethyl-1,3,2-dioxaborolan-2-yl)oxy)silane (**5b**).

1-Methoxy-4-(4-methylpent-4-en-2-yl)benzene (**6a**)

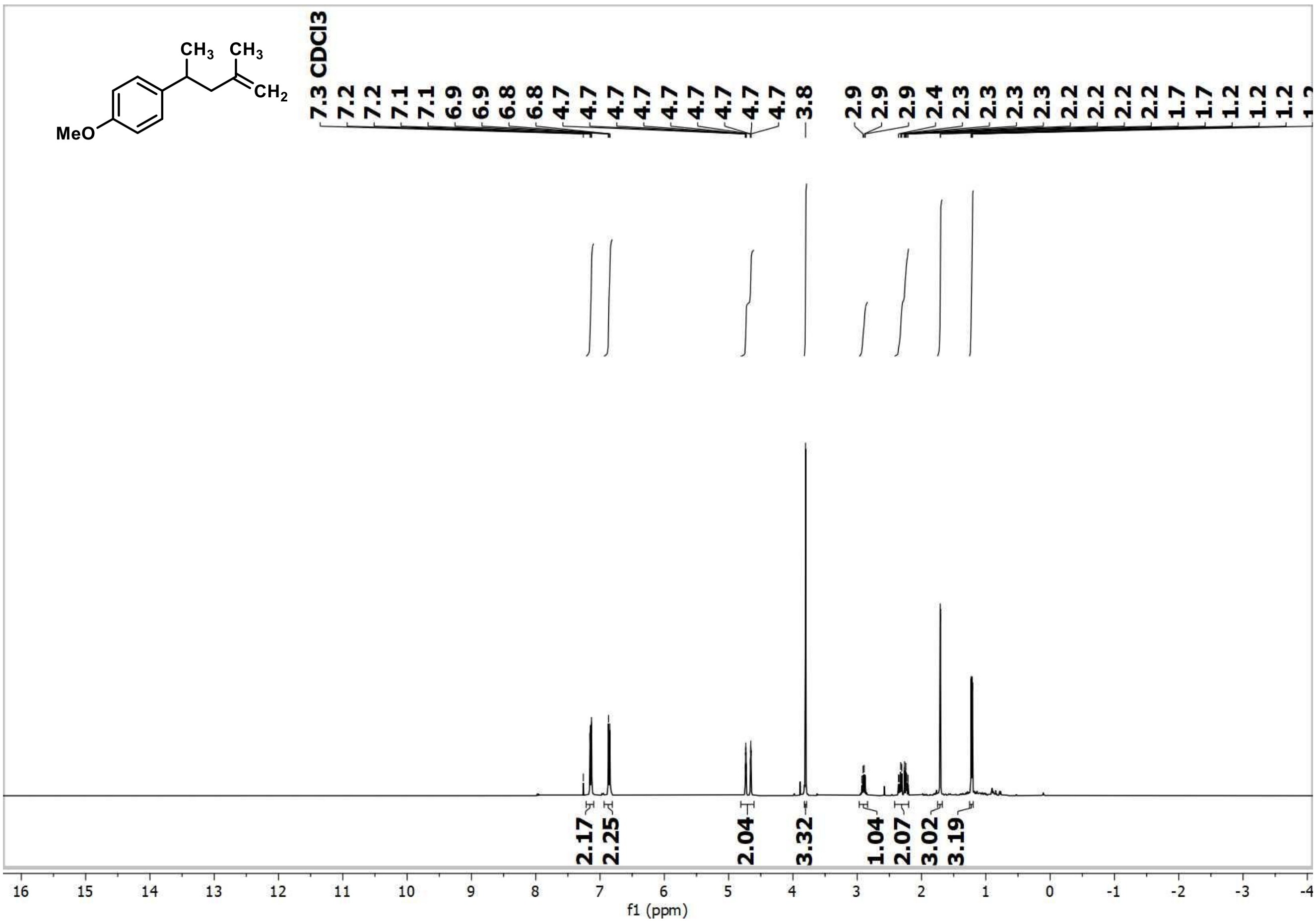

Figure S128. ^{1}H NMR (400 MHz, Chloroform-d, 25°C) of 1-methoxy-4-(4-methylpent-4-en-2-yl)benzene (**6a**).

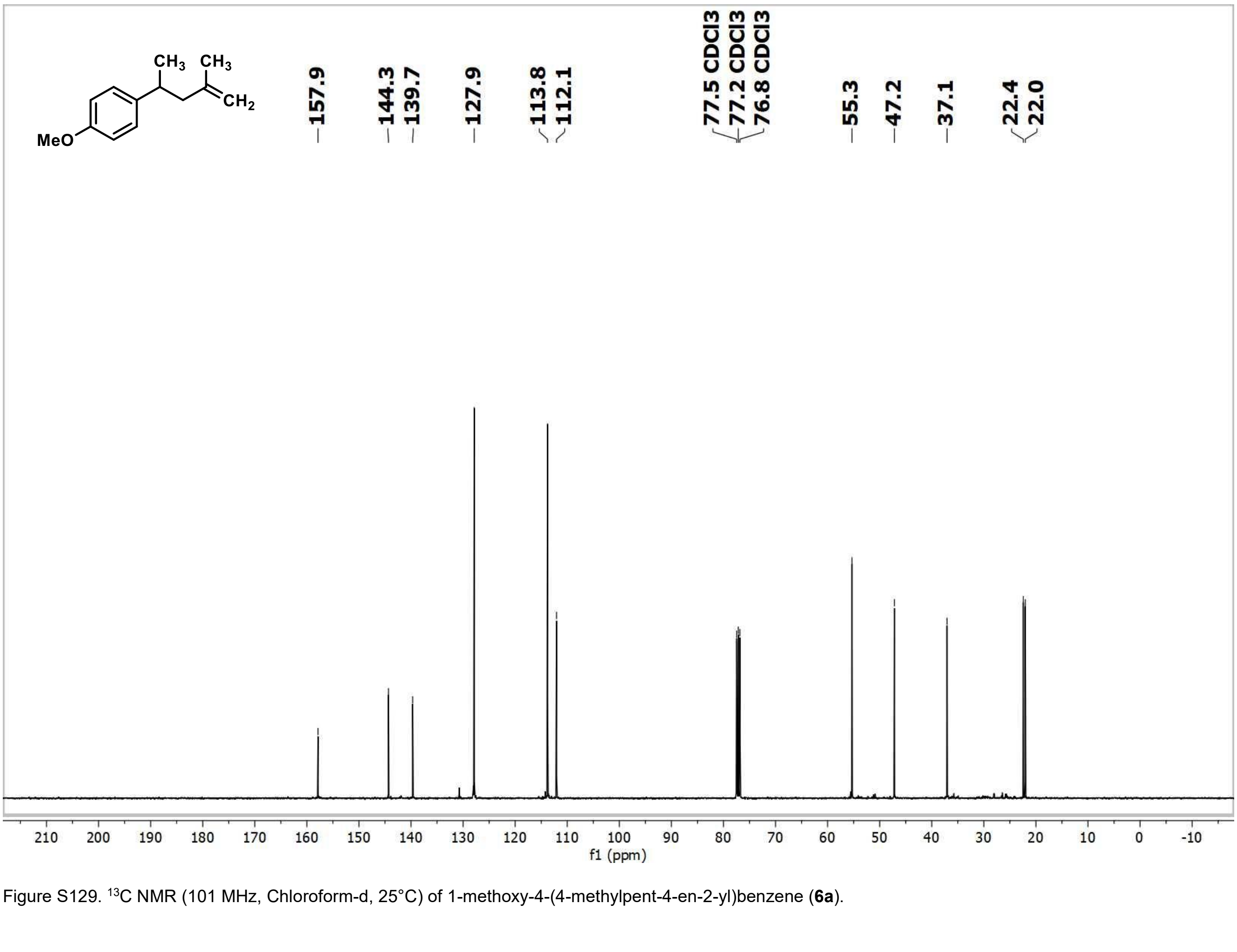

Figure S129. ^{13}C NMR (101 MHz, Chloroform-d, 25°C) of 1-methoxy-4-(4-methylpent-4-en-2-yl)benzene (**6a**).

(1-Cyclopropyl-3-methylbut-3-en-1-yl)benzene (**6b**)

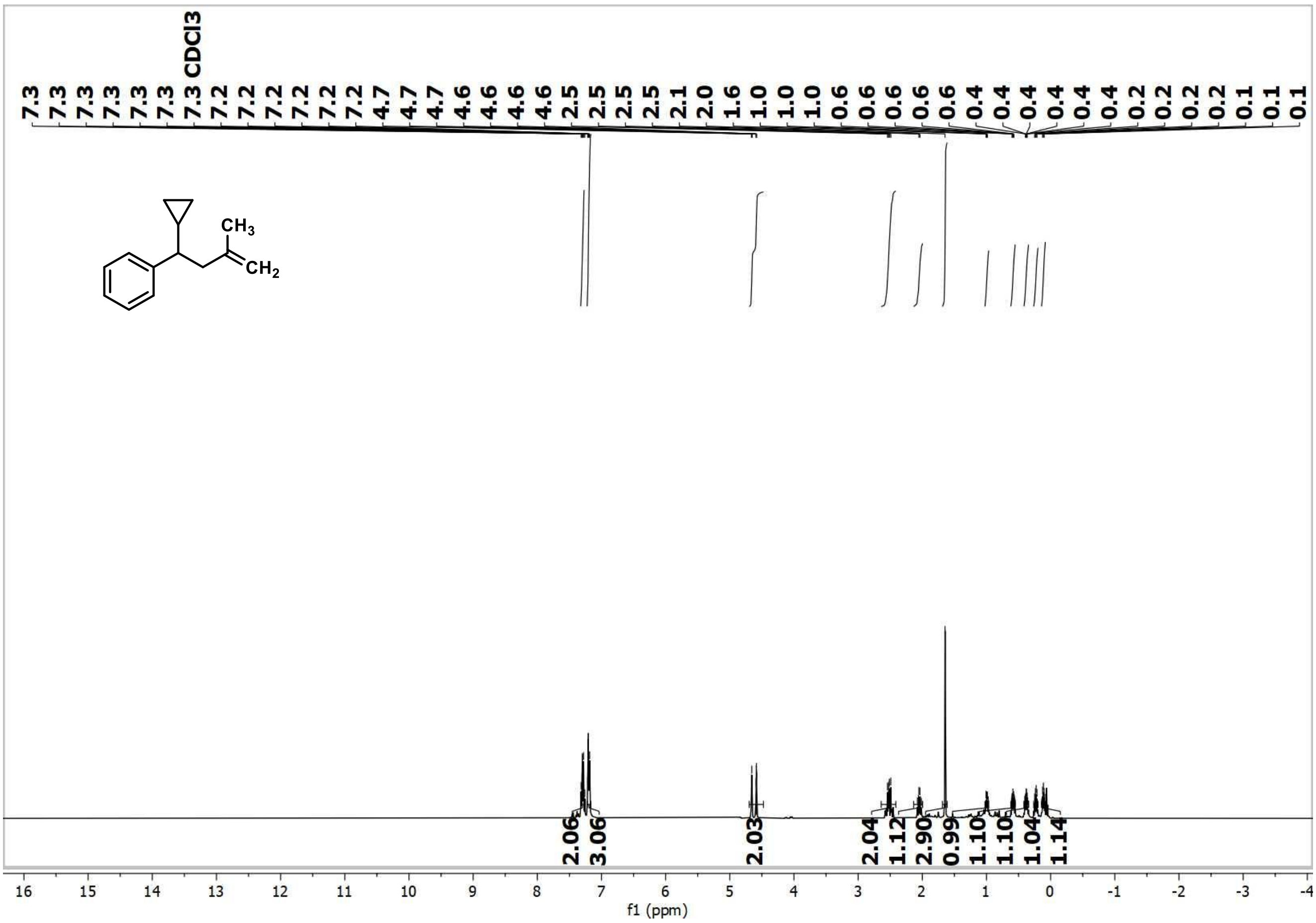

Figure S130. ^{1}H NMR (400 MHz, Chloroform-d, 25°C) of (1-cyclopropyl-3-methylbut-3-en-1-yl)benzene (**6b**).

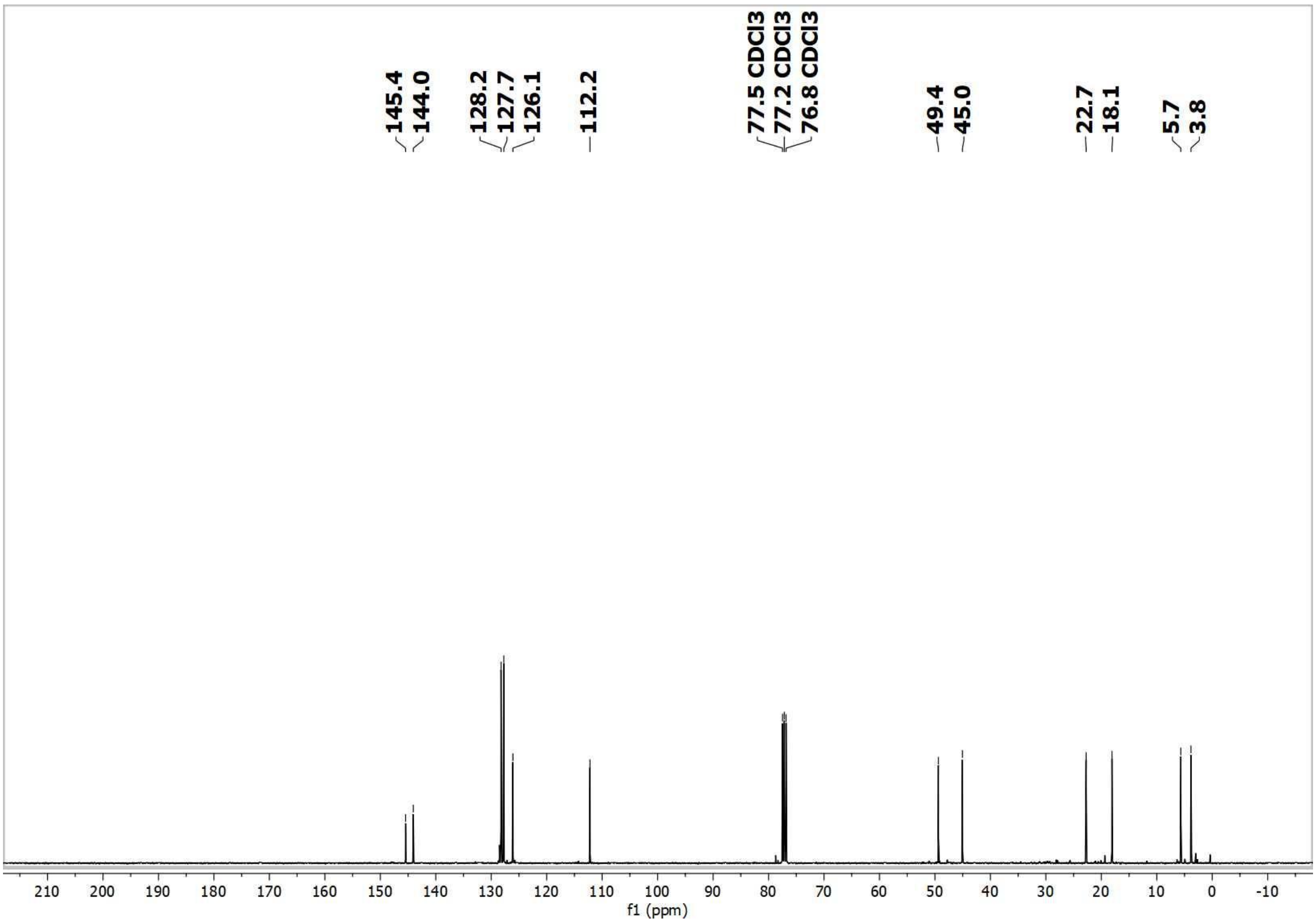

Figure S131. ^{13}C NMR (101 MHz, Chloroform-d, 25°C) of (1-cyclopropyl-3-methylbut-3-en-1-yl)benzene (**6b**).

Trimethyl(octylthio)silane (**7a**)

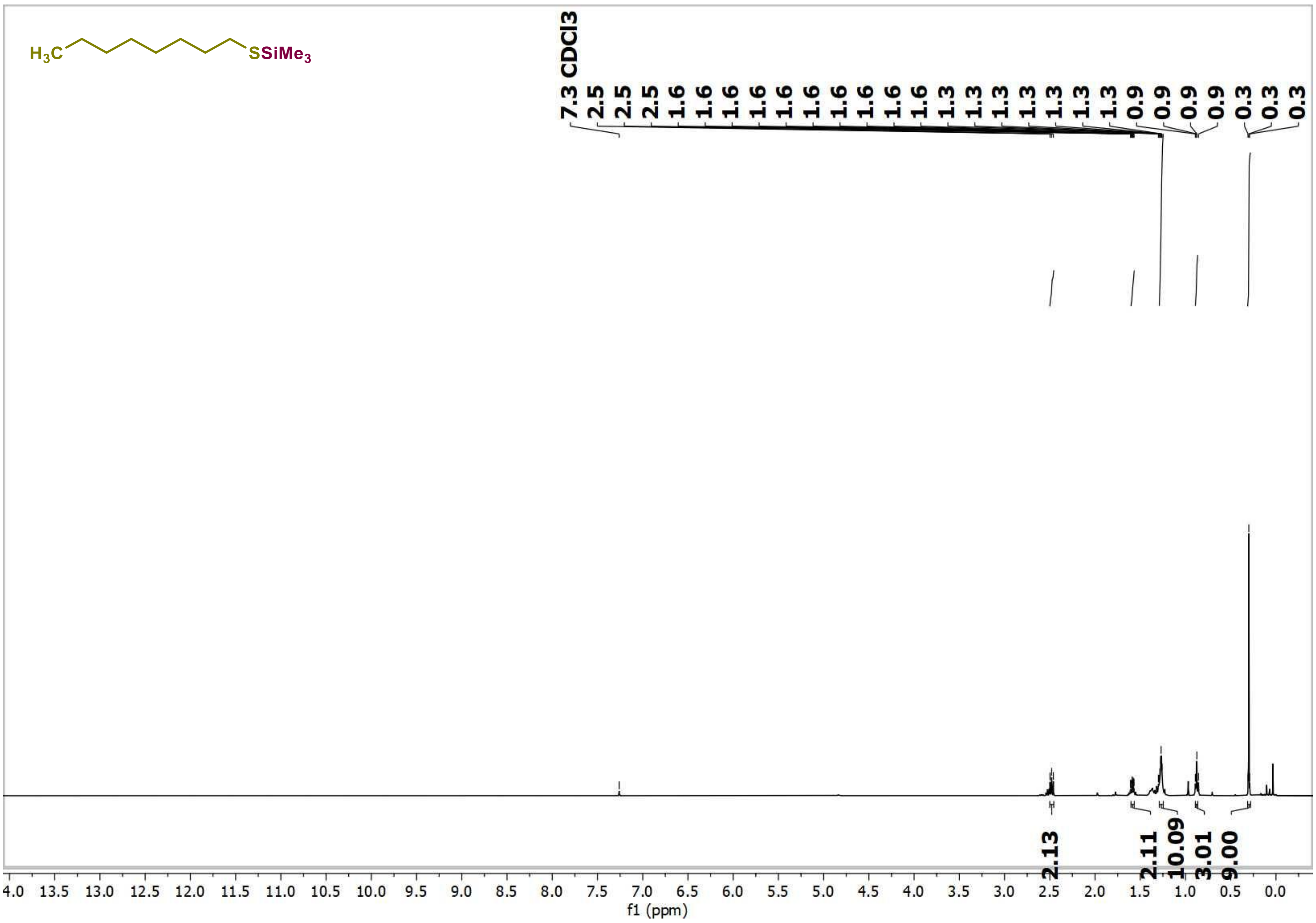

Figure S132. ^{1}H NMR (400 MHz, Chloroform-d, 25°C) of trimethyl(octylthio)silane (**7a**).

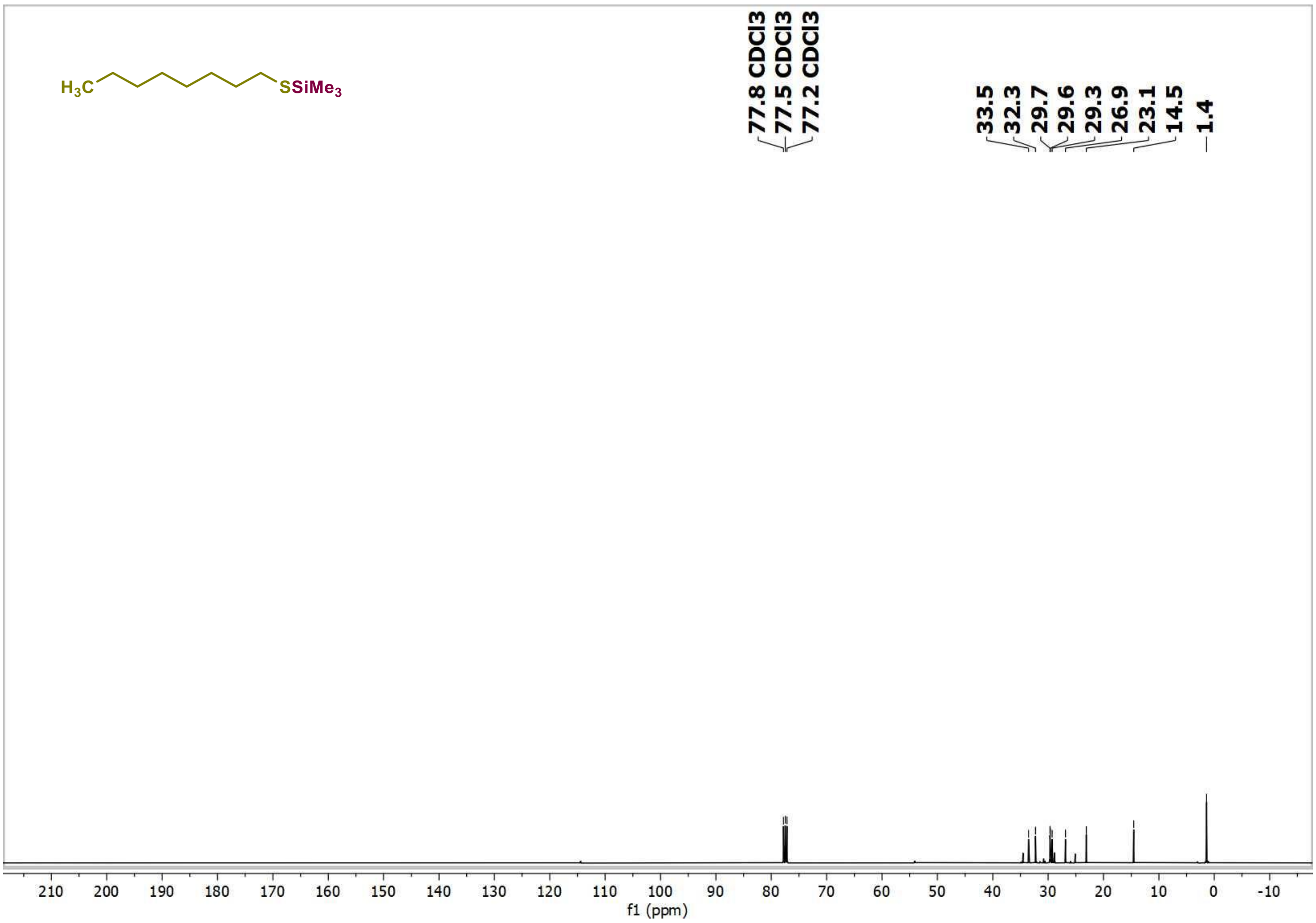

Figure S133. ^{13}C NMR (101 MHz, Chloroform-d, 25°C) of trimethyl(octylthio)silane (**7a**).

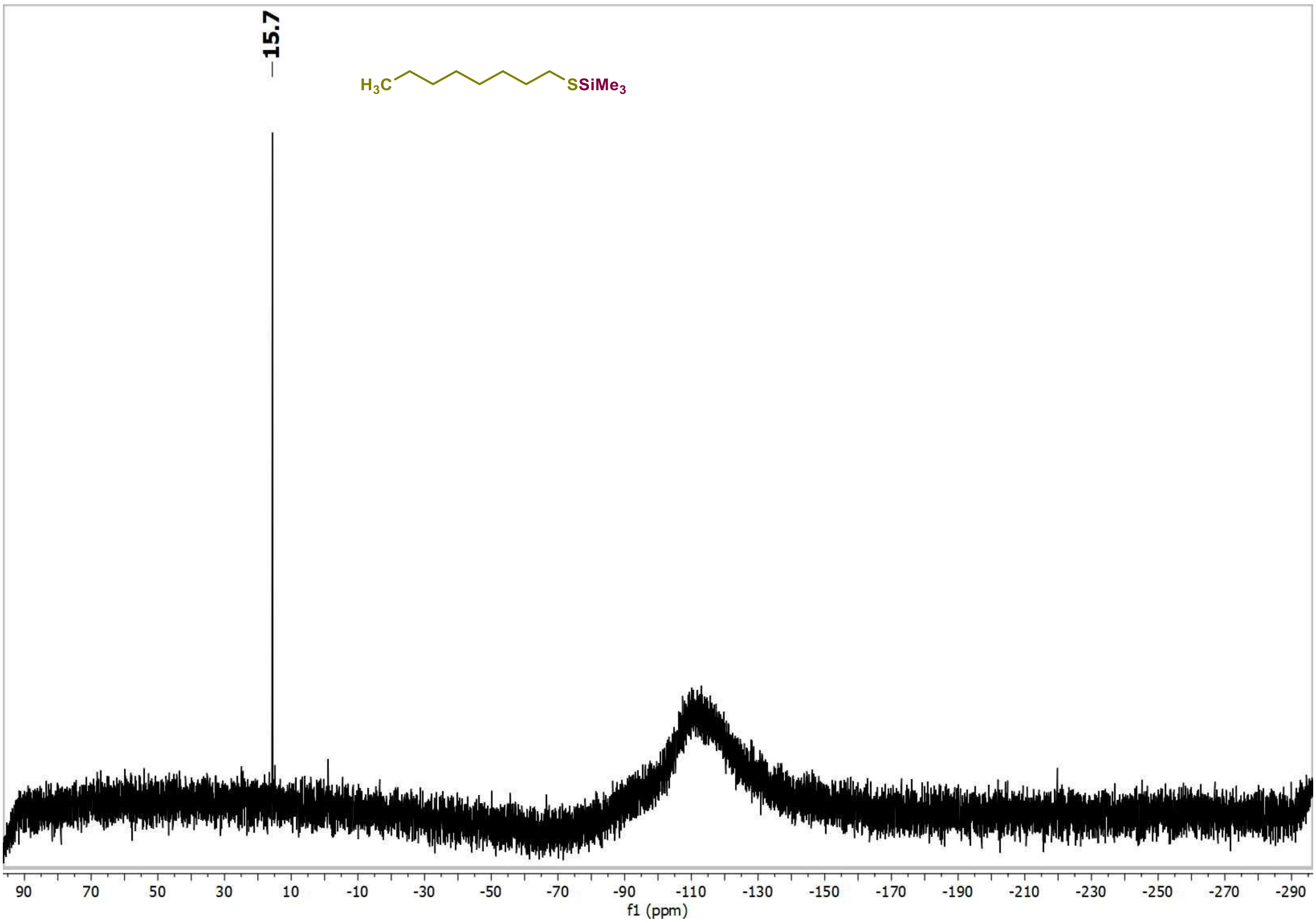

Figure S134. ^{29}Si NMR (79 MHz, Chloroform-d, 25°C) of trimethyl(octylthio)silane (**7a**).

1,1,1-Triisopropyl-3,3,3-trimethyldisilathiane (**7b**)

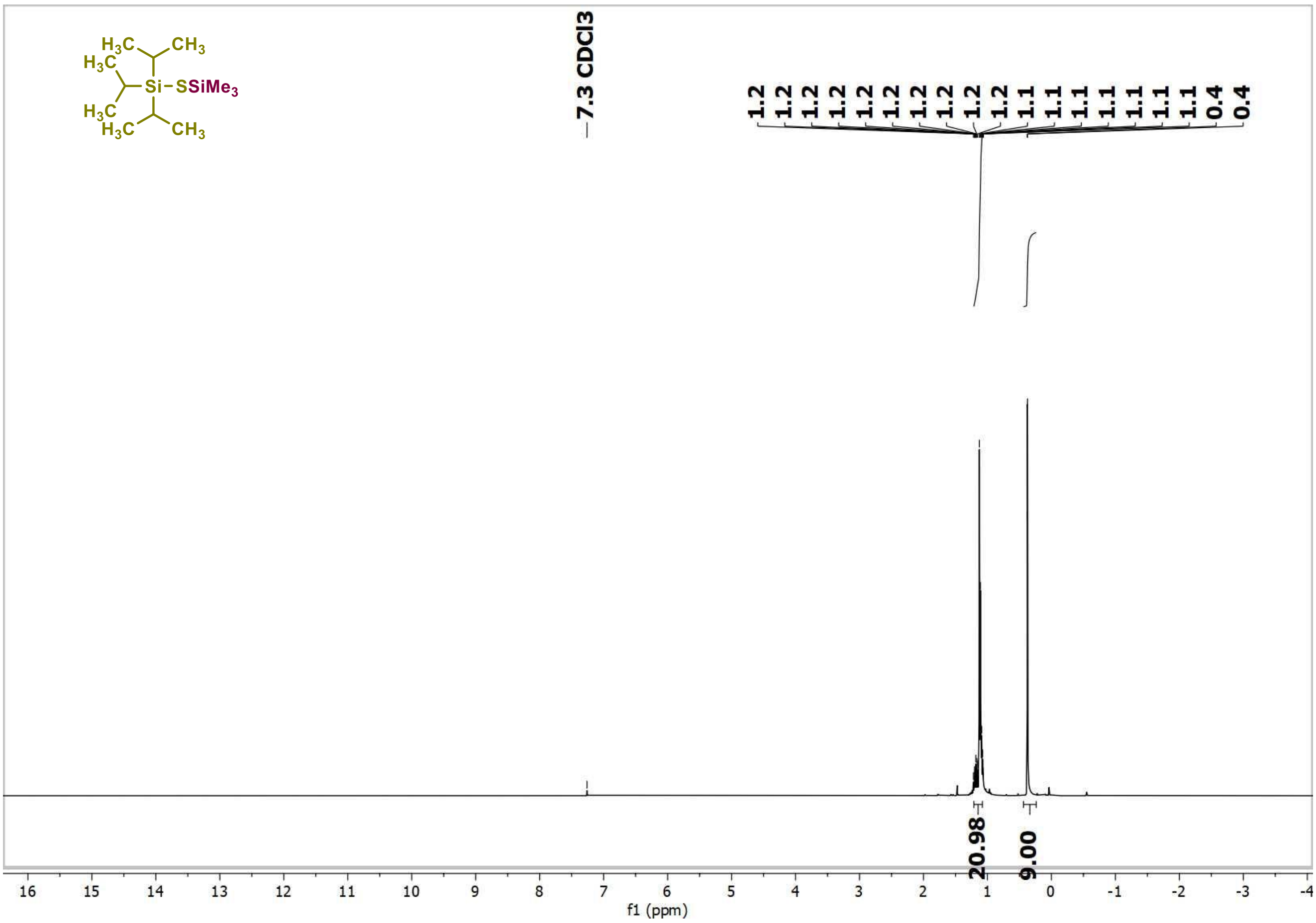

Figure S135. ^{1}H NMR (400 MHz, Chloroform-d, 25°C) of 1,1,1-triisopropyl-3,3,3-trimethyldisilathiane (**7b**).

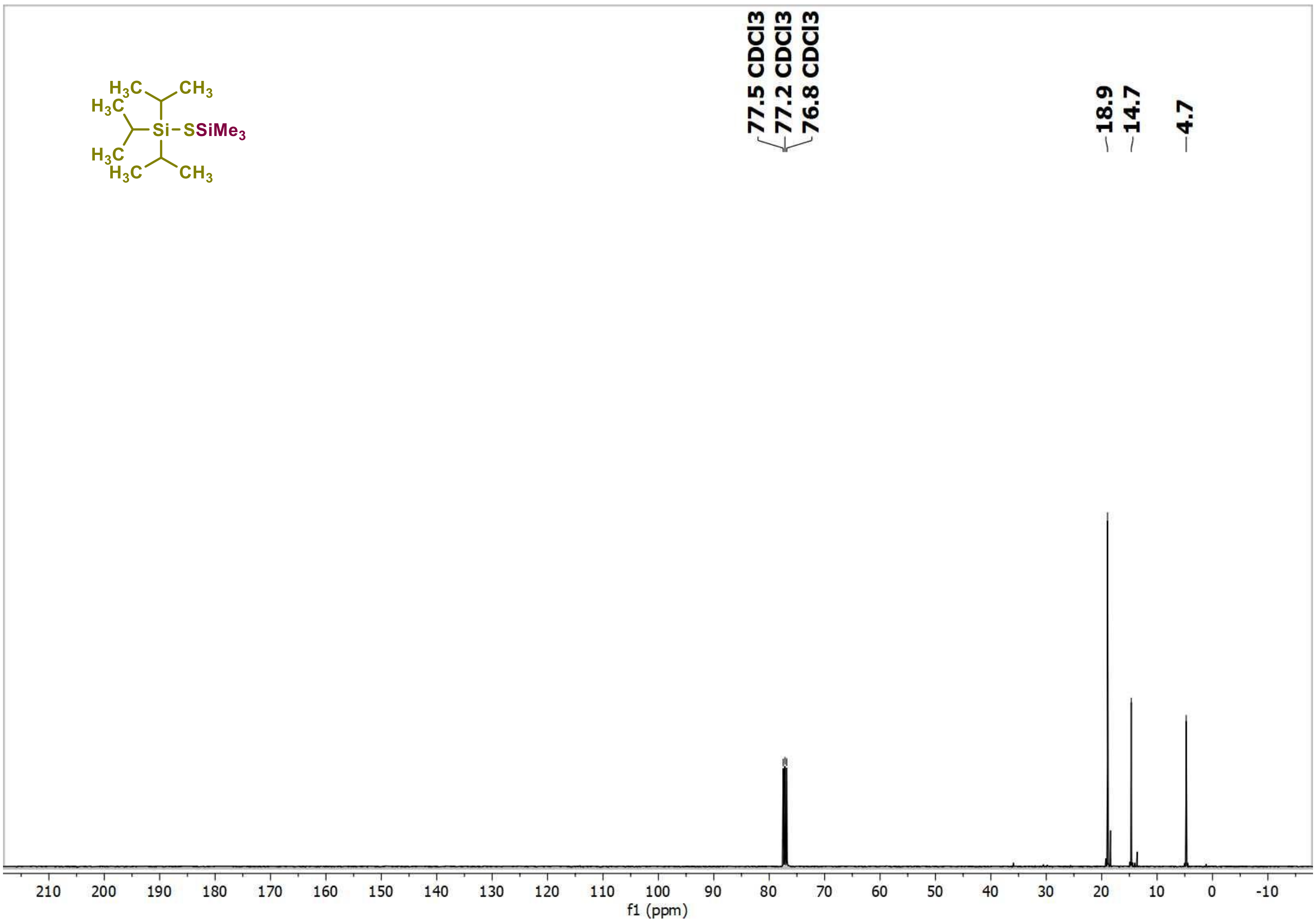

Figure S136. ^{1}H NMR (400 MHz, Chloroform-d, 25°C) of 1,1,1-triisopropyl-3,3,3-trimethyldisilathiane (**7b**).

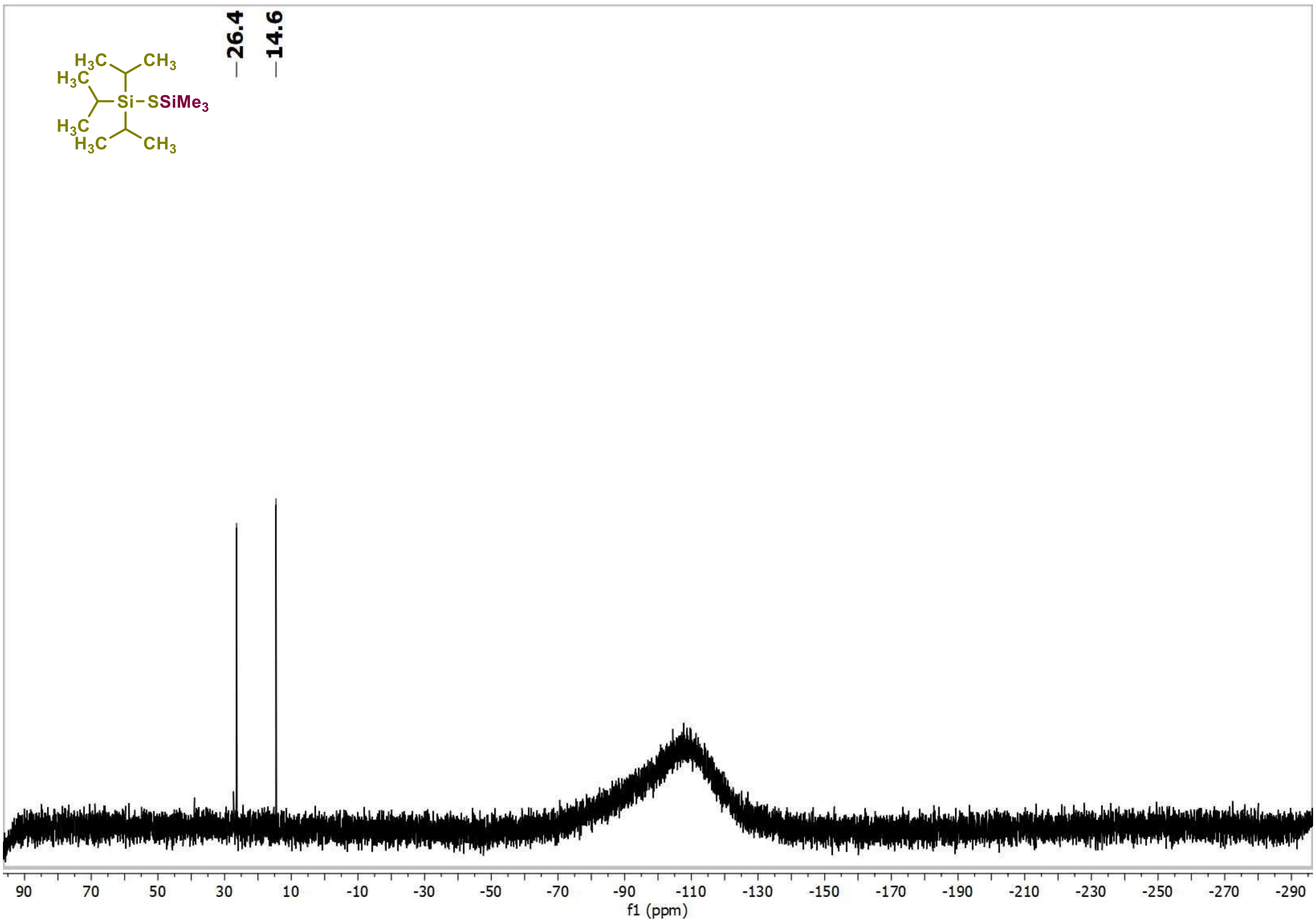

Figure S137. ^{29}Si NMR (79 MHz, Chloroform-d, 25°C) of 1,1,1-triisopropyl-3,3,3-trimethyldisilathiane (**7b**).

MECHANISTIC STUDIES NMR

Hexafluoroisopropanol

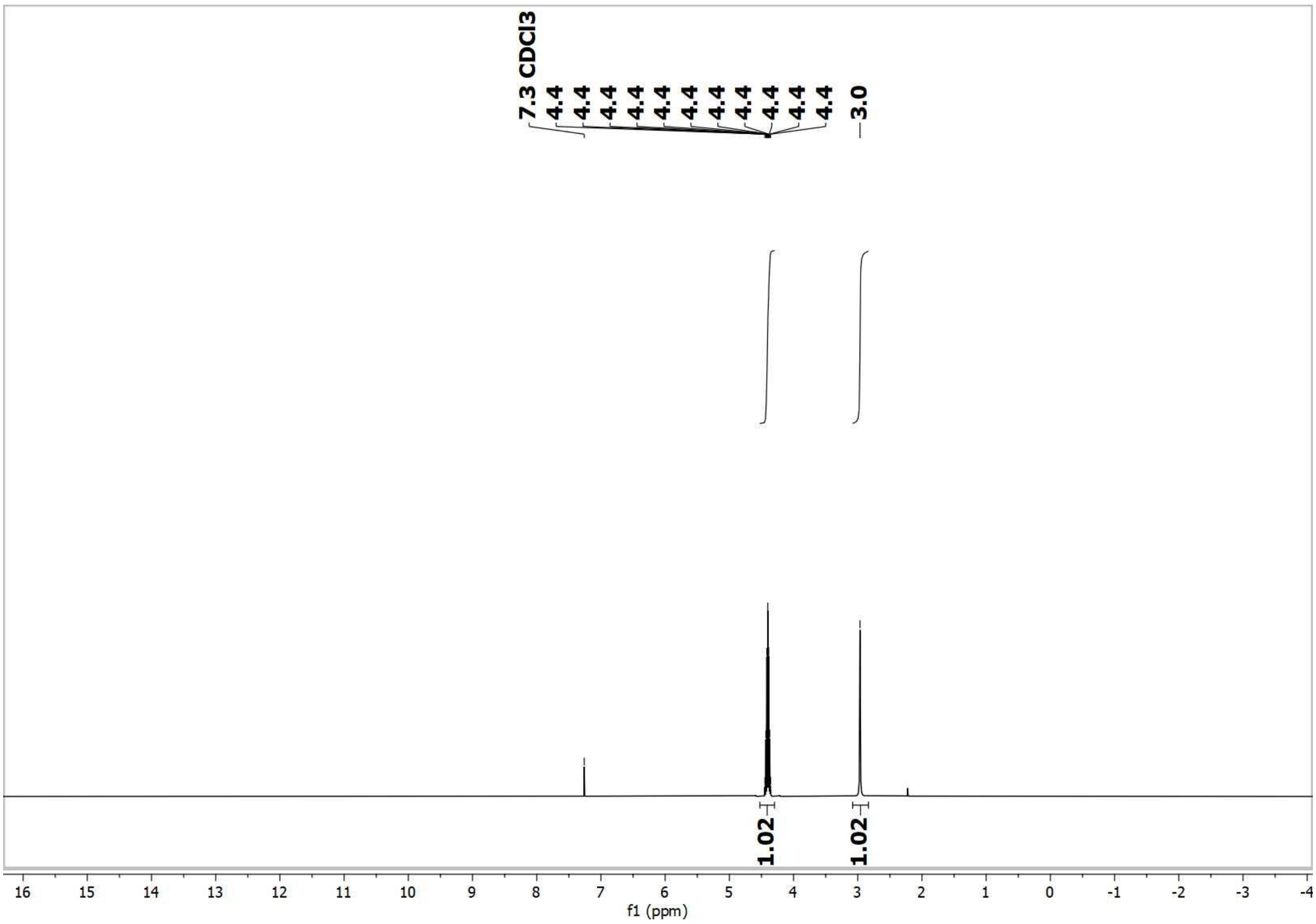

Figure S137. ^{1}H NMR (400 MHz, Chloroform-d, 25°C) of HFIP.

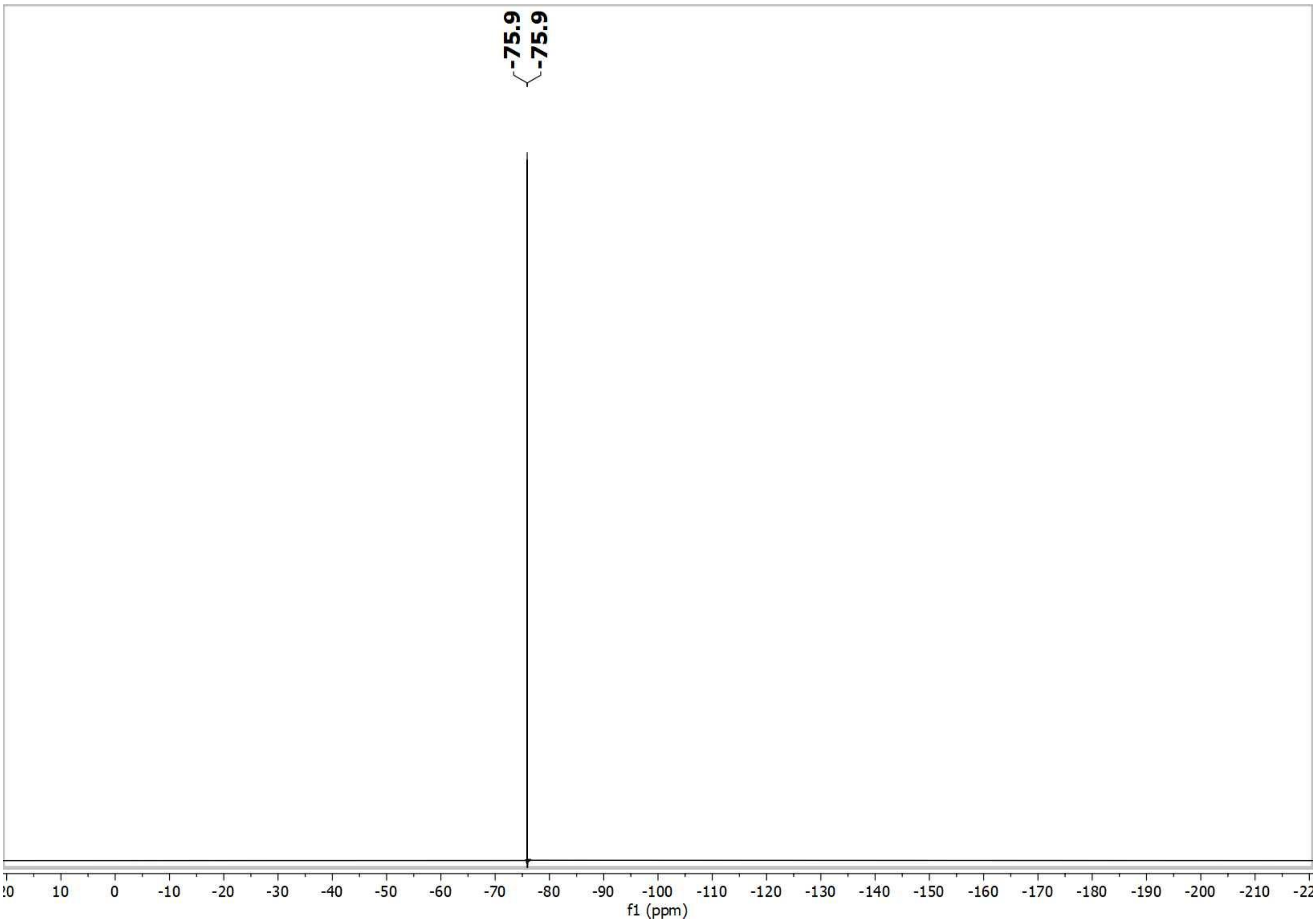

Figure S138. ^{19}F NMR (377 MHz, Chloroform-d, 25°C) of HFIP.

3-Fluorobenzyl alcohol (**1b**)

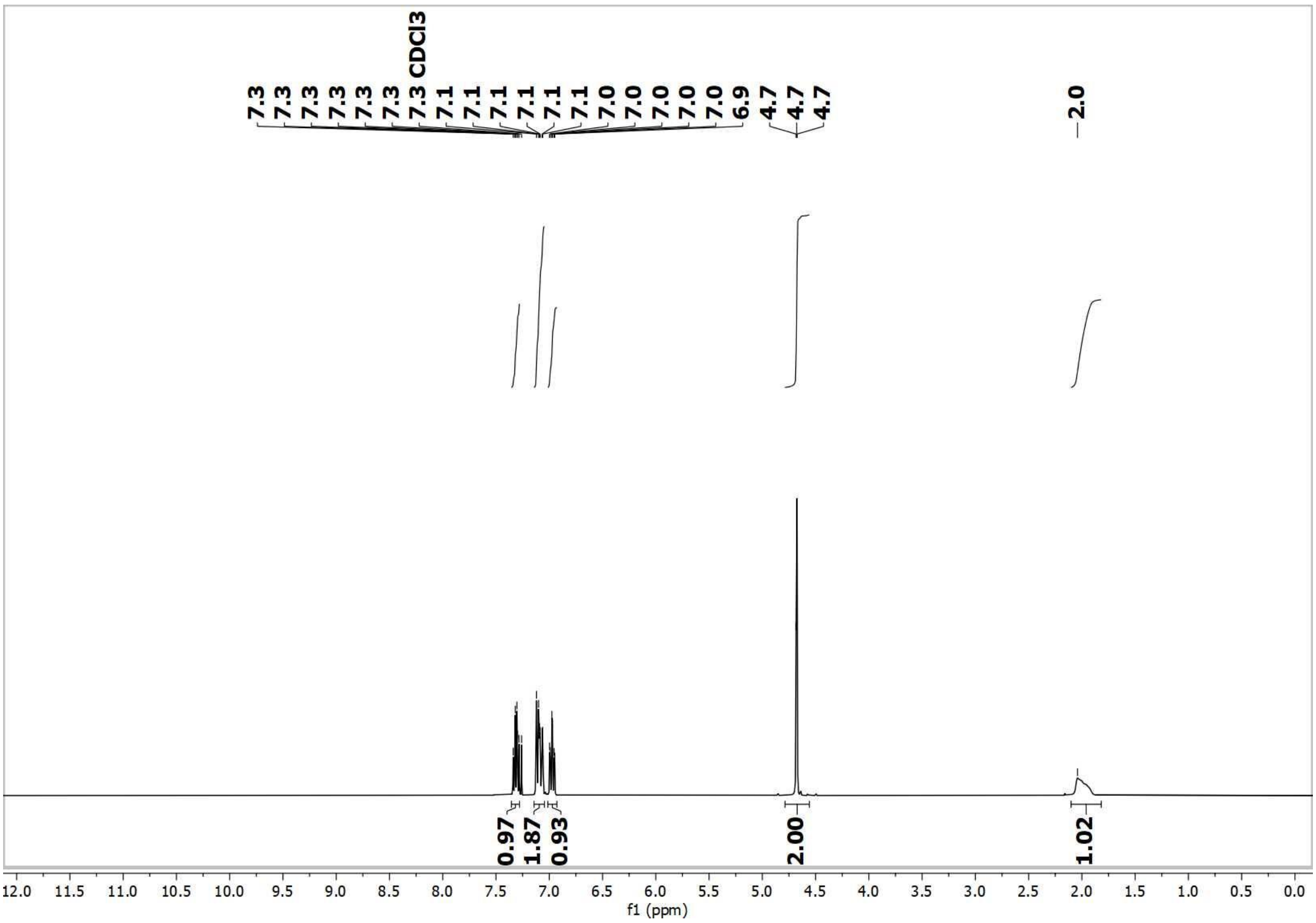

Figure S139. ^{1}H NMR (400 MHz, Chloroform-d, 25°C) of 3-fluorobenzyl alcohol (**1b**).

2-Methylallyltrimethylsilane (**2b**)

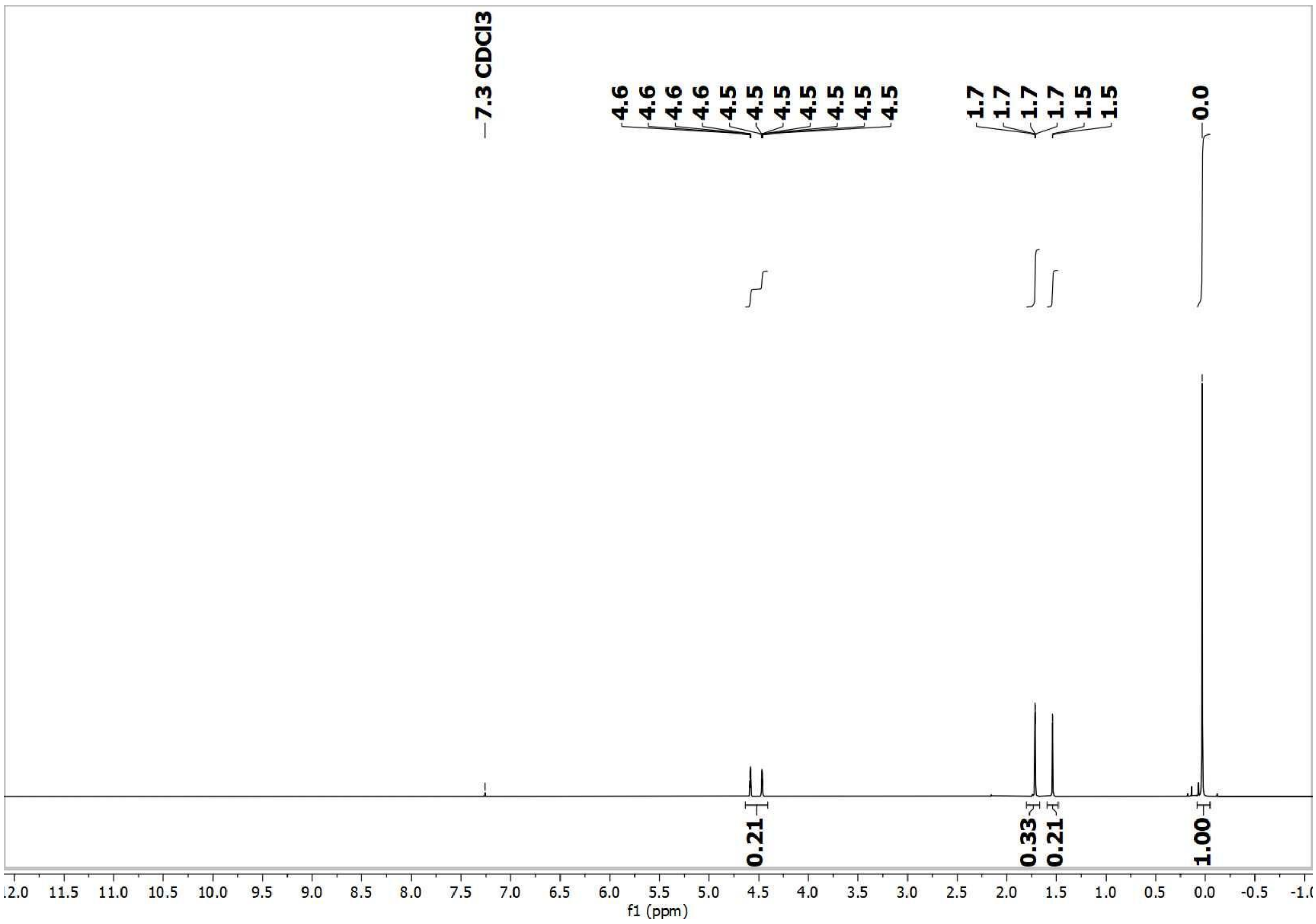

Figure S140. ^{1}H NMR (400 MHz, Chloroform-d, 25°C) of 2-methylallyltrimethylsilane (**2b**).

HFIP + **1b** (normal)

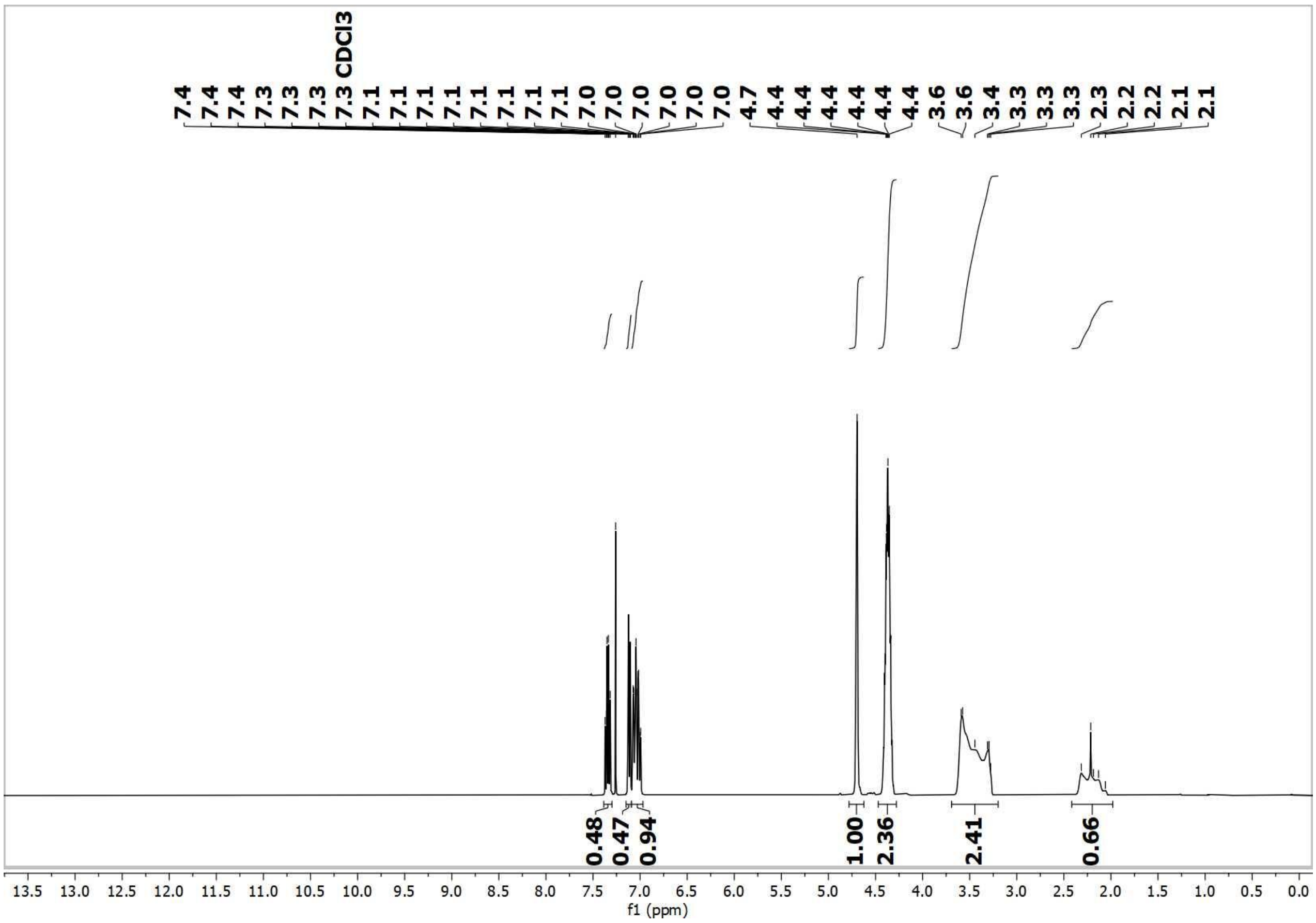

Figure S141. ^{1}H NMR (400 MHz, Chloroform-d, 25°C) of mixture of HFIP and 3-fluorobenzyl alcohol (**1b**).

HFIP + **2b** (normal)

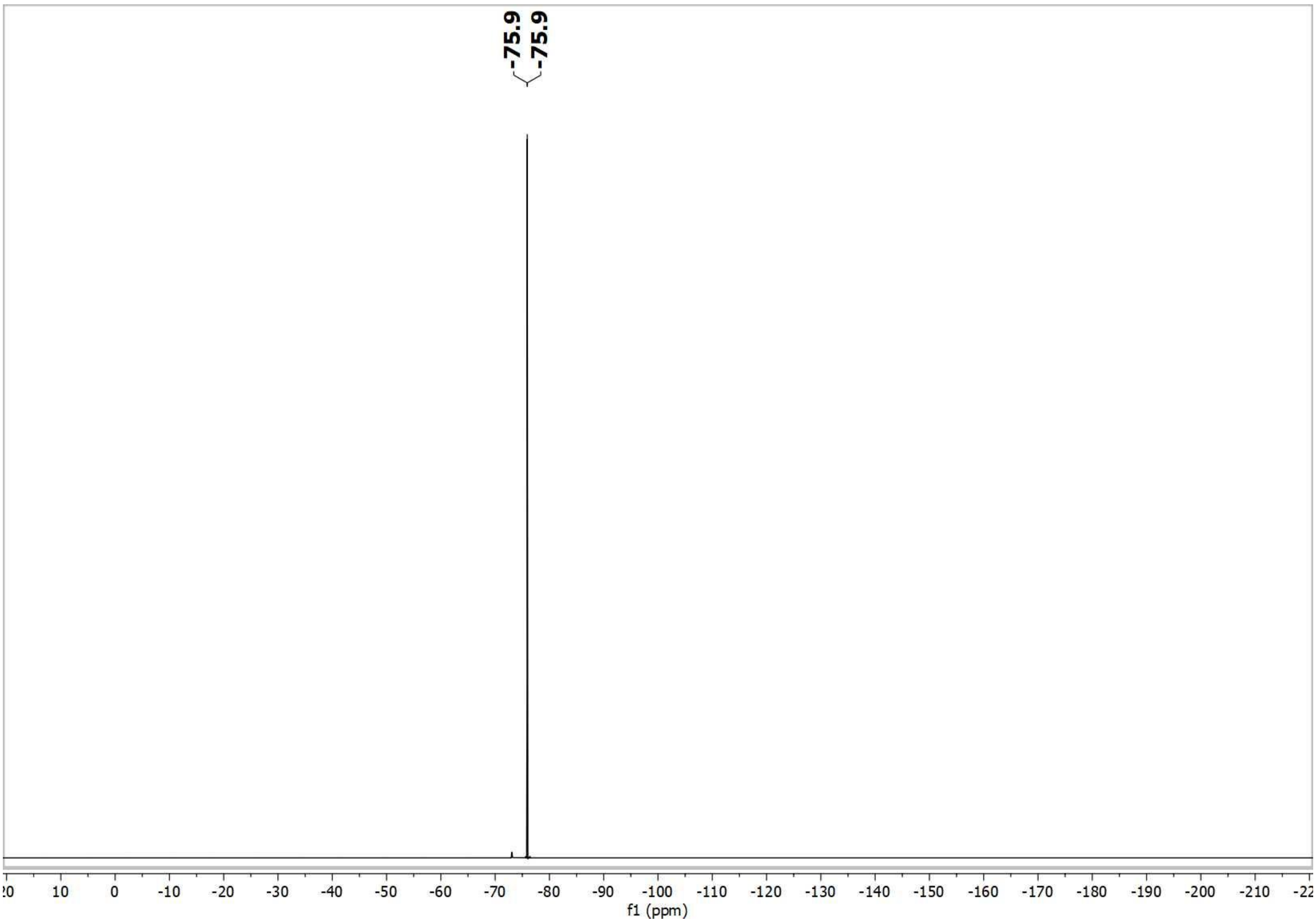

Figure S142. ^{19}F NMR (377 MHz, Chloroform-d, 25°C) of mixture of HFIP and 2-methylallyltrimethylsilane (**2b**).

HFIP + **1b** (stacked)

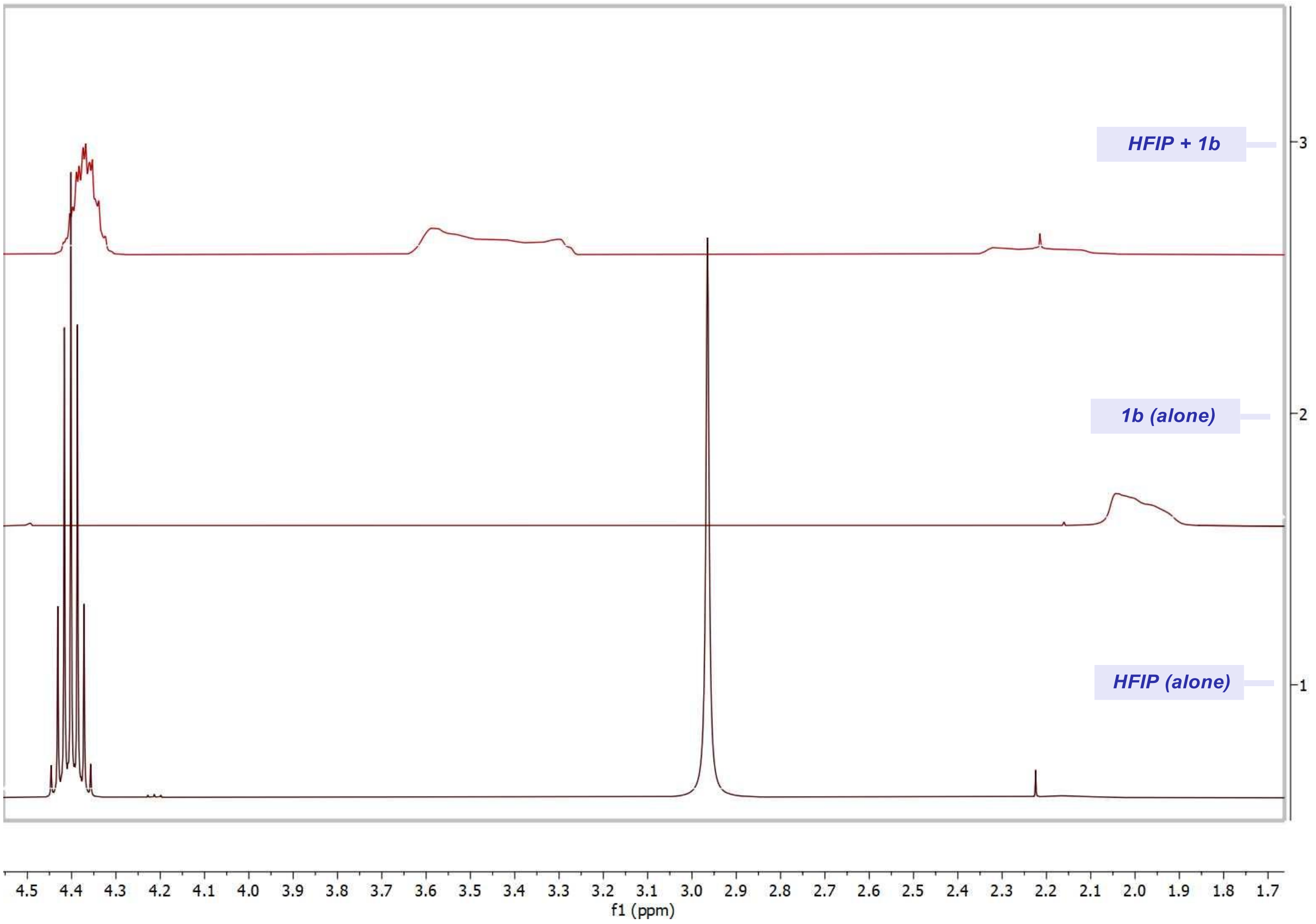

Figure S143. Stacked ^{1}H NMR (400 MHz, Chloroform-d, 25°C) of mixture of HFIP and 3-fluorobenzyl alcohol (**1b**).

HFIP + **2b** (stacked)

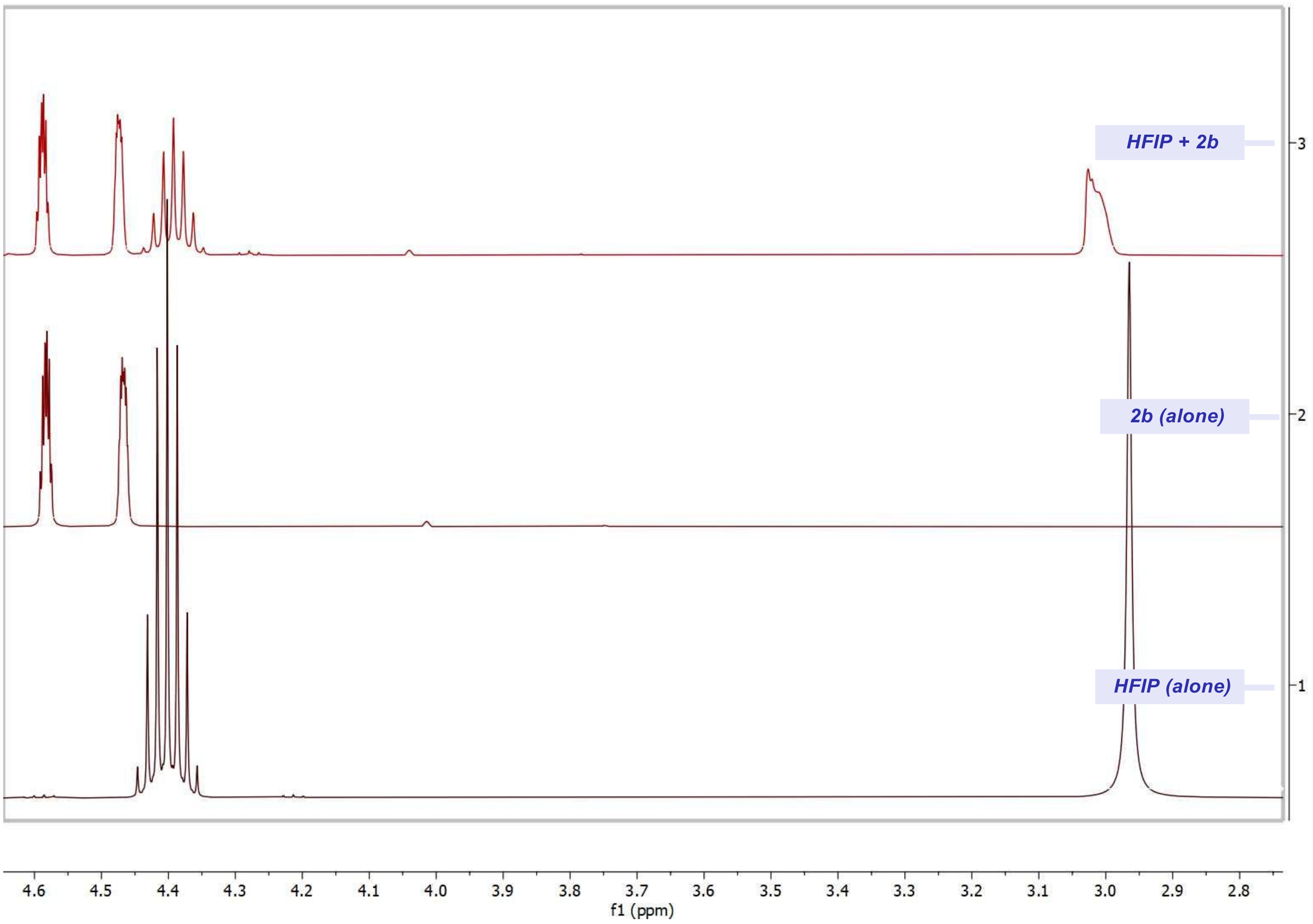

Figure S144. Stacked ^{1}H NMR (400 MHz, Chloroform-d, 25°C) of mixture of HFIP and 2-methylallyltrimethylsilane (**2b**).

Preliminary computational studies

Preliminary computational insight into the HFIP⋯π interaction.[27]

Atomistic models of HFIP and 2-methylallyltrimethylsilane were constructed using GaussView 6.1.1. The initial structure of the HFIP–2-methylallyltrimethylsilane complex was generated through a conformational search using the Macromodel[28] module implemented in the Schrödinger suite. The top-ranked conformer, based on the lowest energy obtained with the OPLS4 force field,[29] was selected for further investigation.

All quantum chemical calculations were performed using Gaussian 16.[30] Interaction energies were evaluated within the supermolecular approach. Density functional theory (DFT) calculations were carried out using the long-range corrected ωB97X-D functional. Geometry optimizations of obtained HFIP–2-methylallyltrimethylsilane complex in the first step of calculations were performed at the ωB97X-D/aug-cc-pVTZ level of theory, and single-point interaction energies were subsequently computed at the same level. All calculations were carried out in the gas phase. The basis set superposition error (BSSE) was corrected using the Boys–Bernardi counterpoise method. The interaction energy was calculated as the difference between the energy of the complex and the sum of the counterpoise-corrected energies of the monomers, computed in the geometry of the complex.

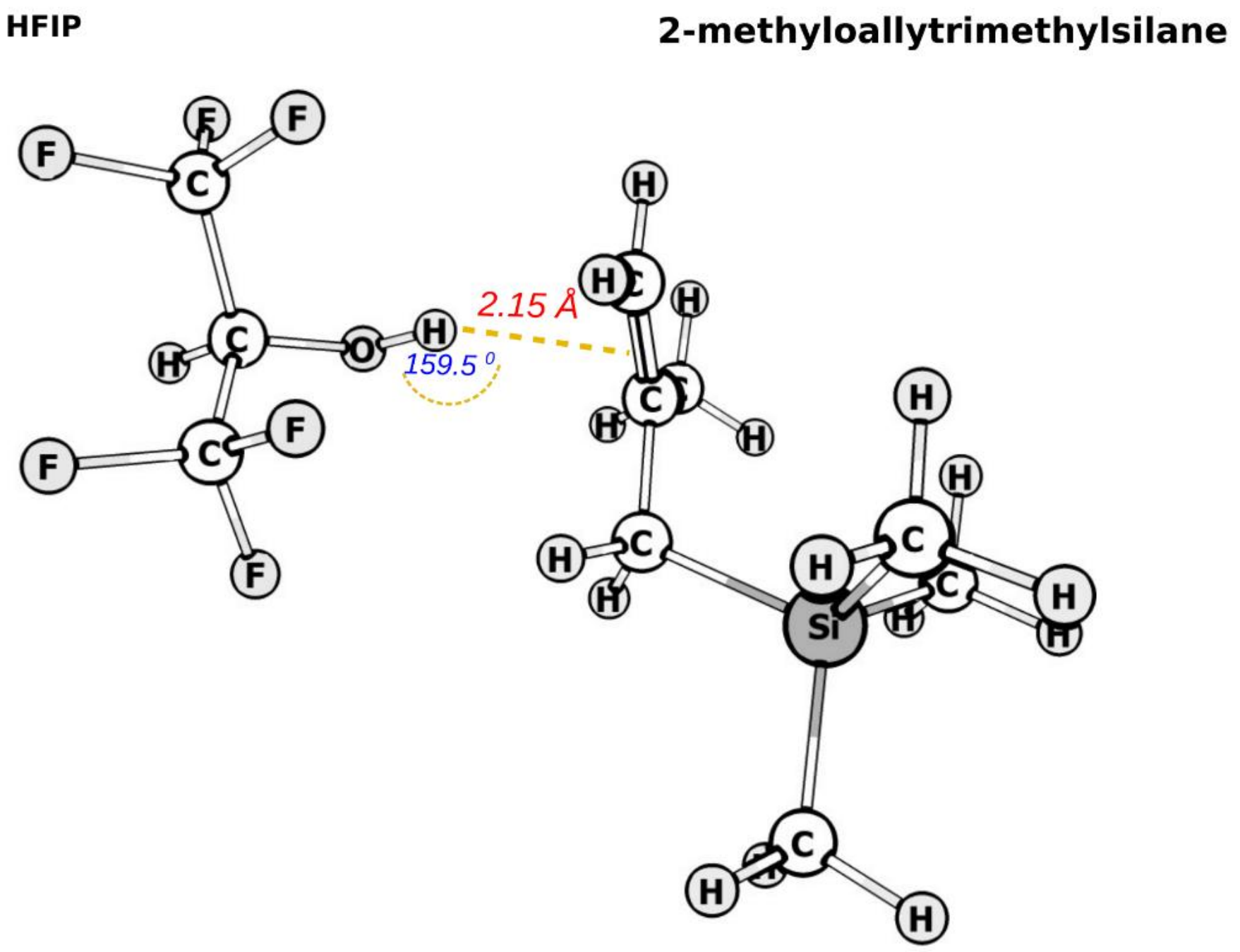

Fig. SX. Proposed structure model of HFIP–2-methylallyltrimethylsilane complex. The geometry was calculated at the ωB97X-D/aug-cc-pVTZ level of theory. OH⋯π interaction between HFIP and 2-methylallyltrimethylsilane was highlighted.

References

(1) Li, L.-H.; Wang, D.; Chan, T. H. Regioselective Alkylation of 1-Silyl-2-Methylallyl Carbanions. *Tetrahedron Lett.* **1991**, *32* (25), 2879–2882. https://doi.org/10.1016/0040-4039(91)80637-L.

(2) Kaźmierczak, J.; Kuciński, K.; Hreczycho, G. Highly Efficient Catalytic Route for the Synthesis of Functionalized Silsesquioxanes. *Inorg. Chem.* **2017**, *56* (15), 9337–9342. https://doi.org/10.1021/acs.inorgchem.7b01504.

(3) Suzuki, T.; Watahiki, T.; Oriyama, T. A Novel and Efficient Method for the Silylation of Alcohols with Methallylsilanes Catalyzed by $Sc(OTf)_3$. *Tetrahedron Lett.* **2000**, *41* (46), 8903–8906.

(4) Kaźmierczak, J.; Kuciński, K.; Stachowiak, H.; Hreczycho, G. Introduction of Boron Functionalities into Silsesquioxanes - Novel Independent Methodologies. *Chem. Eur. J.* **2018**, *24* (10), 2509–2514. https://doi.org/10.1002/chem.201705898.

(5) Kuciński, K.; Stachowiak, H.; Hreczycho, G. Silylation of Alcohols, Phenols, and Silanols with Alkynylsilanes - an Efficient Route to Silyl Ethers and Unsymmetrical Siloxanes. *Eur. J. Org. Chem.* **2020**, *2020* (26), 4042–4049. https://doi.org/10.1002/ejoc.202000573.

(6) Yadollahi, B.; Mirkhani, V.; Tangestaninejad, S.; Karimian, D. Rapid and Efficient Protection of Alcohols and Phenols, and Deprotection of Trimethylsilyl Ethers Catalyzed by a Cerium-Containing Polyoxometalate. *App. Organomet. Chem.* **2011**, *25* (2), 83–86. https://doi.org/10.1002/aoc.1715.

(7) Foumani, P. P.; Mousapour, M.; Shirini, F.; Tajik, H.; Hassanpoor, S. Protection of Alcohols in the Presence of a New Nano-Sized DABCO-Based Ionic Liquid Catalyst Containing Zinc Cation. *J. IRAN. CHEM. SOC.* **2024**, *21* (2), 359–371. https://doi.org/10.1007/s13738-023-02928-1.

(8) Zareyee, D.; Asghari, R.; Khalilzadeh, M. A. Silylation of Alcohols and Phenols with Hexamethyldisilazane over Highly Reusable Propyl Sulfonic Acid Functionalized Nanostructured SBA-15. *Chin. J. Catal.* **2011**, *32* (11), 1864–1868. https://doi.org/10.1016/S1872-2067(10)60300-2.

(9) Lange, M.; Barakat, A.; Vilotijevic, I. Silyl Ethers as Latent Pronucleophiles in Enantioselective Lewis Base Catalyzed Synthesis of Allylic Ethers from Allylic Fluorides. *Synlett* **2025**, *36* (07), 879–883. https://doi.org/10.1055/a-2447-4273.

(10) Broniarz, K.; Lewandowski, D.; Hreczycho, G. Cobalt-Driven Selective Hydrogermylation of Terminal and Internal Alkynes. *J. Catal.* **2025**, *452*, 116467. https://doi.org/10.1016/j.jcat.2025.116467.

(11) Nagase, Y.; Shirai, H.; Kaneko, M.; Shirakawa, E.; Tsuchimoto, T. Indium-Catalyzed Annulation of 3-Aryl- and 3-Heteroarylindoles with Propargyl Ethers: Synthesis and Photoluminescent Properties of Aryl- and Heteroaryl[c]Carbazoles. *Org. Biomol. Chem.* **2013**, *11* (9), 1456–1459. https://doi.org/10.1039/C3OB27407A.

(12) Sadrtdinova, G. I.; Ovchinnikova, V. I.; Komarov, P. D.; Ilyin, S. O.; Vinogradov, A. A.; Vinogradov, A. A.; Ivchenko, P. V.; Nifant'ev, I. E. Heterocene Catalysts for Copolymerization of Hex-1-Ene and Polar Vinyl Monomers. *Pet. Chem.* **2024**, *64* (11), 1253–1265. https://doi.org/10.1134/S0965544124060161.

(13) Deng, T.; Mazumdar, W.; Yoshinaga, Y.; Patel, P. B.; Malo, D.; Malo, T.; Wink, D. J.; Driver, T. G. Rh_2(II)-Catalyzed Intermolecular N-Aryl Aziridination of Olefins Using Nonactivated N Atom Precursors. *J. Am. Chem. Soc.* **2021**, *143* (45), 19149–19159. https://doi.org/10.1021/jacs.1c09229.

(14) Nishiyama, Y.; Kajimoto, H.; Kotani, K.; Nishida, T.; Sonoda, N. Reaction of Carbonyl Compounds with Trialkylsilyl Phenylselenide and Tributylstannyl Hydride under Radical Conditions. *J. Org. Chem.* **2002**, *67* (16), 5696–5700. https://doi.org/10.1021/jo025681q.

(15) Khazaei, A.; Rostami, A.; Mahboubifar, M. Efficient Trimethylsilylation of Alcohols and Phenols with HMDS in the Presence of a Catalytic Amount of 1,3-Dibromo-5,5-Dimethylhydantoin (DBDMH) as a Safe and Cheap Industrial Chemical. *J. Chin. Chem. Soc.* **2007**, *54* (2), 483–487. https://doi.org/10.1002/jccs.200700068.

(16) Nishiguchi, I.; Kita, Y.; Watanabe, M.; Ishino, Y.; Ohno, T.; Maekawa, H. Facile O-Silylation of Tertiary Alcohols in the Presence of Mg-Metal. *Synlett* **2000**, *2000* (7), 1025–1027. https://doi.org/10.1055/s-2000-6676.

(17) Kvíčalová, M.; Blechta, V.; Kobylczyk, K.; Piekos, R.; Schraml, J. Silicon-29 NMR Spectra of *Tert*-Butyldimethylsilyl and Trimethylsilyl Derivatives of Some Non-Rigid Diols. *Collect. Czech. Chem. Commun.* **1997**, *62* (5), 761–768. https://doi.org/10.1135/cccc19970761.

(18) Zeynizadeh, B.; Sorkhabi, S. Fast and Efficient Method for Silylation of Alcohols and Phenols with HMDS in the Presence of Bis-Thiourea Complexes of Cobalt, Nickel, Copper and Zinc Chlorides. *Phosp. Sulfur Silicon Rel. Elem.* **2018**, *193* (3), 127–135. https://doi.org/10.1080/10426507.2017.1417294.

(19) Brondani, P. B.; Mittersteiner, M.; Voigt, M. A.; Klinkowski, B. H.; Scharf, D. R.; Jesus, P. C. de. Synthetic Versatility of Lipases: Application for Si–O Bond Formation and Cleavage. *Synthesis* **2018**, *51*, 477–485. https://doi.org/10.1055/s-0037-1610281.

(20) Markwitz, M.; Łyczek, K.; Bellina, F.; Del Vecchio, A.; Kuciński, K. Potassium Hydroxide as a Cost-Effective Catalyst for Broad-Scope Silylation with $TMSCF_3$. *J. Org. Chem.* **2025**, *90* (37), 13040–13046. https://doi.org/10.1021/acs.joc.5c01625.

(21) Jereb, M. Highly Atom Economical Uncatalysed and I2-Catalysed Silylation of Phenols, Alcohols and Carbohydrates, Using HMDS under Solvent-Free Reaction Conditions (SFRC). *Tetrahedron* **2012**, *68* (20), 3861–3867. https://doi.org/10.1016/j.tet.2012.03.040.

(22) Wang, Q.; Chen, M.; Zou, C.; Chen, C. Direct Synthesis of Polar-Functionalized Polyolefin Elastomers. *Angew. Chem., Int. Ed.* **2025**, *64* (20), e202423814. https://doi.org/10.1002/anie.202423814.

(23) Chatterjee, B.; Gunanathan, C. Ruthenium-Catalysed Multicomponent Synthesis of Borasiloxanes. *Chem. Commun.* **2017**, *53* (16), 2515–2518. https://doi.org/10.1039/C7CC00787F.

(24) Ito, M.; Itazaki, M.; Nakazawa, H. Selective Boryl Silyl Ether Formation in the Photoreaction of Bisboryloxide/Boroxine with Hydrosilane Catalyzed by a Transition-Metal Carbonyl Complex. *J. Am. Chem. Soc.* **2014**, *136* (17), 6183–6186. https://doi.org/10.1021/ja500465x.
(25) Markwitz, M.; Labrzycki, K.; Łyczek, K.; Kuciński, K. Functionalization of Electron-Rich Secondary Benzyl Alcohols in HFIP: From Thioethers to Trisubstituted Methanes. *J. Org. Chem.* **2025**, *90* (27), 9513–9518. https://doi.org/10.1021/acs.joc.5c00900.
(26) Tarafder, K.; Surendranath, Y.; Olshansky, J. H.; Alivisatos, A. P.; Wang, L.-W. Hole Transfer Dynamics from a CdSe/CdS Quantum Rod to a Tethered Ferrocene Derivative. *J. Am. Chem. Soc.* **2014**, *136* (13), 5121–5131. https://doi.org/10.1021/ja500936n.
(27) GaussView, Version 6.1, Roy Dennington, Todd A. Keith, and John M. Millam, Semichem Inc., Shawnee Mission, KS, 2016.
(28) Mohamadi, F.; Richard, N. G.; Guida, W. C.; Liskamp, R.; Lipton, M.; Caufield, C.; Chang, G.; Hendrickson, T.; Still, W. C., “MacroModel – an integrated software system for modeling organic and bioorganic molecules using molecular mechanics. *J. Comput. Chem.* **1990**, *11*, 440–467.
(29) Lu, C.; Wu, C.; Ghoreishi, D.; Chen, W.; Wang, L.; Damm, W.; et al. OPLS4: improving force field accuracy on challenging regimes of chemical space. J. Chem. Theory Comput. 2021, 17, 4291–4300.
(30) Gaussian 16, Revision C.01, M. J. Frisch, G. W. Trucks, H. B. Schlegel, G. E. Scuseria, M. A. Robb, J. R. Cheeseman, G. Scalmani, V. Barone, G. A. Petersson, H. Nakatsuji, X. Li, M. Caricato, A. V. Marenich, J. Bloino, B. G. Janesko, R. Gomperts, B. Mennucci, H. P. Hratchian, J. V. Ortiz, A. F. Izmaylov, J. L. Sonnenberg, D. Williams-Young, F. Ding, F. Lipparini, F. Egidi, J. Goings, B. Peng, A. Petrone, T. Henderson, D. Ranasinghe, V. G. Zakrzewski, J. Gao, N. Rega, G. Zheng, W. Liang, M. Hada, M. Ehara, K. Toyota, R. Fukuda, J. Hasegawa, M. Ishida, T. Nakajima, Y. Honda, O. Kitao, H. Nakai, T. Vreven, K. Throssell, J. A. Montgomery, Jr., J. E. Peralta, F. Ogliaro, M. J. Bearpark, J. J. Heyd, E. N. Brothers, K. N. Kudin, V. N. Staroverov, T. A. Keith, R. Kobayashi, J. Normand, K. Raghavachari, A. P. Rendell, J. C. Burant, S. S. Iyengar, J. Tomasi, M. Cossi, J. M. Millam, M. Klene, C. Adamo, R. Cammi, J. W. Ochterski, R. L. Martin, K. Morokuma, O. Farkas, J. B. Foresman, and D. J. Fox, Gaussian, Inc., Wallingford CT, 2016.
