# Supplementary material for: Unlocking Lewis acid–free metalation of O–nucleophiles in HFIP
Source: Commun Chem. 2026 Jun 2;9:288. doi: 10.1038/s42004-026-02079-3 (PMC13526861; doi:10.1038/s42004-026-02079-3)
Supplement: Supplementary file 3 [file 42004_2026_2079_MOESM3_ESM.pdf]

Description of Additional Supplementary Files:

File name: Supplementary Data 1

Description: Supplementary Data 1 contains preliminary computational data in .XYZ format
